# Structural and Functional Neuroimaging Findings in Fibromyalgia: A Systematic Review

**DOI:** 10.1002/ejp.70331

**Published:** 2026-07-12

**Authors:** Flávio Antônio Duboc Flutt, João Pedro de Melo Cortez, Lucas Rego Ramos, Rayssa Paula Paz Furlanetto, Diogo Goulart Corrêa, Nivaldo Ribeiro Villela

**Affiliations:** ^1^ Pain Center, Rio de Janeiro State University Rio de Janeiro RJ Brazil; ^2^ Graduate Program in Clinical and Experimental Pathophysiology Rio de Janeiro State University Rio de Janeiro RJ Brazil; ^3^ Emotion Neurophysiology Laboratory Rio de Janeiro State University Rio de Janeiro RJ Brazil; ^4^ Programa de Pós‐Graduação Em Neurociências Translacional (PGNET) Universidade Federal do Rio de Janeiro Rio de Janeiro RJ Brazil; ^5^ Department of Radiology Rio de Janeiro State University Rio de Janeiro RJ Brazil; ^6^ Discipline of Neurosurgery, Faculty of Medical Sciences Rio de Janeiro State University Rio de Janeiro RJ Brazil

## Abstract

**Background and Objective:**

Fibromyalgia (FM) is a nociplastic chronic pain syndrome in which neuroimaging indicates central nervous system involvement, yet findings remain inconsistent across methods. We systematically reviewed structural and functional neuroimaging studies in adults with FM to identify the most reproducible cross‐modal alterations.

**Databases and Data Treatment:**

Following a PROSPERO‐registered protocol (CRD420251234980) and PRISMA 2020, we searched PubMed, EMBASE, Web of Science and VHL/BVS for English‐language studies published between 1 January 2010 and 16 October 2025. Eligible studies compared FM (any American College of Rheumatology criteria) with healthy controls and reported quantitative structural MRI, diffusion/structural connectivity or resting‐state/task‐based fMRI.

**Results:**

Ninety‐one studies were included (24 morphometry, 8 diffusion/structural connectivity, 32 resting‐state fMRI, 40 task‐based fMRI). Morphometry most consistently showed reduced grey matter volume or cortical thickness in anterior cingulate, insular and prefrontal/orbitofrontal cortices, with recurrent reductions in temporal cortex, posterior cingulate, amygdala and hippocampus; thalamic increases were less frequent. Diffusion findings were sparse and bidirectional, yielding no stable white‐matter alterations. Resting‐state fMRI was highly heterogeneous, with contradictory default‐mode, salience/insula and periaqueductal grey (PAG)‐related connectivity changes. Task‐based fMRI provided the clearest support for central sensitization, showing hyper‐responsivity of nociceptive networks with dysregulated prefrontal engagement and weakened rostral anterior cingulate cortex (rACC)–PAG/brainstem descending modulation.

**Conclusions:**

FM shows distributed brain alterations, with the most robust signals reflecting nociceptive amplification and impaired top‐down control. Harmonized longitudinal multimodal studies are needed to establish reliable biomarkers.

**Significance Statement:**

This systematic review shows that fibromyalgia neuroimaging findings are not best explained by a single biomarker, but by distributed alterations across pain‐processing, modulatory and affective‐cognitive systems. By comparing structural MRI, diffusion imaging, resting‐state fMRI and task‐based fMRI, the review identifies task‐evoked nociceptive amplification and impaired descending modulation as the most coherent cross‐study signal, while clarifying why resting‐state and diffusion findings remain difficult to translate clinically.

## 1 Introduction

Fibromyalgia (FM) is a chronic pain syndrome characterized by widespread musculoskeletal pain, along with highly frequent symptoms such as persistent fatigue, non‐restorative sleep and cognitive disturbances. It is frequently accompanied by psychiatric and affective comorbidities, with anxiety disorders showing a lifetime prevalence of up to 60% and depression occurring in 14% to 36% of patients (Sarzi‐Puttini et al. 2020), alongside alexithymia, post‐traumatic stress disorder (PTSD), personality disorders and suicidal ideation (Avni et al. 2025; Galvez‐Sánchez et al. 2019; Habibi Asgarabad et al. 2023; Rahangdale and Ferraro 2025). It is the third most prevalent musculoskeletal condition, affecting approximately 2%–3% of the global population, and is notably more common in women (Di Carlo et al. 2024; Iannuccelli et al. 2025; Sarzi‐Puttini et al. 2020). The genesis of FM is multifactorial and remains controversial, with no single established cause or specific diagnostic biomarker.

Importantly, the clinical burden of FM is not restricted to pain alone. Neuroimaging evidence from other symptom‐related conditions suggests that mood disturbance, cognitive complaints and sleep disruption may also involve abnormalities in cingulate, prefrontal, insular and large‐scale network systems. In major depressive disorder, structural MRI studies have shown cortical thinning in the orbitofrontal, cingulate, insular and temporal regions (Schmaal et al. 2017), while resting‐state studies have demonstrated altered interactions among frontoparietal control, default mode and salience/emotion‐related networks (Kaiser et al. 2015). Likewise, chronic insomnia has been associated with structural abnormalities in the orbitofrontal and precuneus regions, including orbitofrontal changes related to insomnia severity (Altena et al. 2010), while more recent meta‐analytic evidence has also identified convergent abnormalities in the subgenual anterior cingulate cortex (Reimann et al. 2023). These observations highlight that symptoms such as mood disturbance, cognitive complaints and sleep disruption should be considered when interpreting central nervous system alterations in FM.

Although several mechanisms have been implicated in FM, including chronic inflammation, central sensitization remains a key framework for understanding its symptoms. It is a pathological state characterized by heightened responsiveness to both nociceptive and non‐nociceptive stimuli, such that weak or normally innocuous inputs may be perceived as painful, while painful stimuli may evoke exaggerated responses. Thus, even in the absence of evident tissue damage, patients with FM may experience allodynia and hyperalgesia (Di Carlo et al. 2024; de la Coba et al. 2022).

Within this broader clinical picture, central sensitization represents one important mechanism, but not the entire explanatory model. Current nociplastic frameworks instead suggest that FM reflects the interplay of amplified nociceptive processing, impaired descending modulation and broader affective‐cognitive dysregulation (Nijs et al. 2021; Walitt et al. 2016). Neuroimaging offers a means to examine how these processes converge across structural and functional systems. Across multiple imaging modalities, studies have reported abnormal functional connectivity and structural or volumetric alterations in regions and large‐scale networks involved in sensory, affective, cognitive and pain‐modulatory processing. Collectively, this literature suggests that FM‐related brain alterations extend beyond amplified pain processing and also involve affective, cognitive and sleep‐related dimensions (Sarzi‐Puttini et al. 2020).

Neuroimaging research in FM has used multiple complementary approaches, including structural MRI, diffusion‐based methods and functional MRI, to investigate brain alterations across sensory, affective, cognitive and pain‐modulatory systems. While this multimodal literature has broadened current understanding of the condition, it has also introduced substantial methodological heterogeneity across studies.

In this systematic review, we evaluate and synthesize the existing neuroimaging evidence of functional and structural brain alterations in FM. Our primary aim is to identify recurrent and consistently reported structural and functional neuroimaging patterns across studies, while highlighting areas of inconsistency. We also sought to summarize the main methodological and clinical sources of heterogeneity that should be addressed in future harmonized studies before clinically useful biomarkers can be established.

## 2 Methods

### 2.1 Protocol and Registration

We conducted this systematic review according to the methodological recommendations of the Cochrane Handbook (2019). The study protocol was pre‐registered in the International Prospective Register of Systematic Reviews (PROSPERO) database (ID: CRD420251234980), and this report adheres to the Preferred Reporting Items for Systematic Reviews and Meta‐Analyses (PRISMA) 2020 guidelines (Page et al. 2021).

### 2.2 Eligibility Criteria

To be eligible for inclusion in this review, studies were required to investigate adult patients (aged 18 years or older) with a diagnosis of FM established according to any iteration of the American College of Rheumatology (ACR) criteria (e.g., 1990, 2010 or 2016). A mandatory criterion was the inclusion of a comparison group composed of healthy controls (HCs). The outcomes of interest were restricted to quantitative data from cerebral neuroimaging techniques, including structural MRI (such as VBM or SBM), Diffusion Tensor Imaging (DTI) and functional MRI (both resting‐state and task‐based).

We included observational case–control studies as well as baseline (pre‐intervention) data from clinical trials or longitudinal designs, provided they reported a direct comparison between the FM and HC groups. The search was limited to articles published in the English language between January 1, 2010, and October 16, 2025, in order to prioritize contemporary neuroimaging studies with more comparable MRI acquisition and analytic approaches. We excluded review articles, editorials, letters to the editor, case reports, preclinical (animal) studies, studies lacking a healthy control group, and those exclusively involving paediatric populations.

### 2.3 Neuroimaging Modalities and Analytical Measures

The included studies were grouped according to the primary neuroimaging modality and analytical approach reported by the original authors. Structural MRI analyses have applied voxel‐based morphometry (VBM), surface‐based morphometry (SBM) (Fischl 2012) and regional volumetry to reveal subtle grey‐matter alterations. Diffusion tensor imaging (DTI) and other diffusion‐based methods have been used to evaluate white‐matter microstructure using fractional anisotropy, mean diffusivity and tract‐based spatial statistics (TBSS) (Smith et al. 2006). Functional MRI investigations have employed seed‐based connectivity (Biswal et al. 1995; Fox and Raichle 2007), independent component analysis (ICA) (Beckmann and Smith 2004; Calhoun et al. 2001), ROI‐to‐ROI approaches (Whitfield‐Gabrieli and Nieto‐Castanon 2012) and graph‐theoretical metrics (Rubinov and Sporns 2010) to assess network‐level disturbances. To improve interpretability, key methodological terms and less conventional imaging measures reported across studies were defined during data extraction and are summarized in Table 1.

**TABLE 1:** Neuroimaging modalities, analytical methods and key measures included in this review.

Domain	Method/Measure	Brief description
Structural MRI	Voxel‐based morphometry (VBM)	Whole‐brain voxel‐wise analysis of grey‐ or white‐matter volume/density differences.
	Surface‐based morphometry (SBM)	Surface‐based analysis of cortical structure, usually cortical thickness and/or surface area.
	Regional volumetry	Volume quantification of predefined brain regions or subfields.
	Subcortical shape analysis	Assessment of localized shape abnormalities in subcortical structures beyond total volume.
	Structural covariance	Inter‐regional covariance in morphometric measures across participants, used to infer network‐level structural organization.
Diffusion/structural connectivity	Diffusion tensor imaging (DTI)	Diffusion‐based MRI method used to assess white‐matter microstructure.
	Tract‐based spatial statistics (TBSS)	Voxel‐wise analysis of diffusion‐derived metrics projected onto a white‐matter skeleton.
	Tractography/structural connectivity analyses	Reconstruction or quantification of white‐matter pathways and network organization based on diffusion data.
	Common diffusion‐derived measures	Frequently reported indices included fractional anisotropy (FA), mean diffusivity (MD), radial diffusivity (RD) and axial diffusivity (AD).
Resting‐state fMRI	Seed‐based connectivity	Correlation of spontaneous BOLD fluctuations between a predefined seed region and the rest of the brain or targets.
	ROI‐to‐ROI connectivity	Functional connectivity analysis between predefined regions of interest.
	ICA‐based analyses	Data‐driven decomposition of fMRI data into independent functional networks; some studies used dual regression for subject‐level network estimation.
	Graph‐theoretical analyses	Network topology analyses using measures such as centrality, modularity, efficiency, clustering or community organization.
	Local or frequency‐domain signal metrics	Measures of spontaneous signal properties, including ALFF, fALFF, ReHo, spectral power/FFT and BOLD variability (SDBOLD).
	Directed connectivity approaches	Analyses of directional statistical influence between time series, such as Granger causality.
Task‐based fMRI	GLM and ROI‐based activation analyses	Standard analyses of task‐evoked BOLD responses and condition contrasts, either whole‐brain or within predefined regions.
	Psychophysiological interaction (PPI/gPPI)	Analysis of task‐dependent changes in functional connectivity.
	Seed‐based task connectivity	Condition‐specific connectivity analysis from a predefined seed during task performance.
	ICA‐based analyses	Data‐driven identification of task‐related functional networks.
	Effective connectivity approaches	Model‐based analyses of directional relationships among brain regions, including structural equation modelling (SEM).
	Dynamic functional connectivity	Analysis of time‐varying connectivity during task performance.

*Note:* Methods and measures were grouped according to the terminology used in the included studies. Brief descriptions are provided to improve interpretability.

Abbreviations: ALFF, amplitude of low‐frequency fluctuations; BOLD, blood‐oxygen‐level‐dependent; FA, fractional anisotropy; fALFF, fractional amplitude of low‐frequency fluctuations; GLM, general linear model; ICA, independent component analysis; MD, mean diffusivity; PPI, psychophysiological interaction; ReHo, regional homogeneity; ROI, region of interest; SBM, surface‐based morphometry; SDBOLD, standard deviation of the BOLD signal; SEM, structural equation modelling; TBSS, tract‐based spatial statistics; VBM, voxel‐based morphometry.

### 2.4 Information Sources and Search Strategy

A comprehensive and systematic search of the literature was conducted to identify relevant studies. We performed searches in four principal electronic databases: PubMed, EMBASE, Web of Science and the Virtual Health Library (VHL or BVS). The search strategy was developed in accordance with the PICO (Population, Intervention/Outcome, Comparison) framework and tailored to the syntax of each database.

The search combined controlled vocabularies (such as MeSH and DeCS) with free‐text keywords to maximize sensitivity. The strategy was structured around three core concepts: (1) the population (‘Fibromyalgia’), (2) the outcomes (‘Neuroimaging’ and related terms) and (3) the comparison group (‘Healthy Controls’). The search was limited to articles published between January 1, 2010, and October 16, 2025.

Additionally, to ensure comprehensive retrieval and identify any studies missed by the electronic search, we manually screened the reference lists of relevant systematic reviews identified during the screening process.

The complete and detailed search strategies for all databases are available in Data S1.

### 2.5 Study Selection

The initial database search across the four electronic databases retrieved a total of 1350 citations (PubMed: 254; Embase: 623; Web of Science: 414; BVS: 59). All citations were subsequently imported into the Catchii software (Halman and Oshlack 2024). The software's built‐in deduplication feature identified and removed 541 duplicate records, leaving 809 unique citations for screening.

The screening was conducted in two distinct phases by two independent reviewers (F.F. and J.C.). In Phase 1, the two reviewers independently screened the titles and abstracts of all 809 citations against the pre‐defined eligibility criteria. This initial screening led to the exclusion of 664 records. Any disagreements at this stage were resolved through discussion to reach consensus or, if consensus could not be achieved, by a third screener (R.F.).

The remaining 145 articles were identified as potentially relevant and advanced to Phase 2 for full‐text assessment. The same two reviewers (F.F. and J.C.) independently assessed the full texts of these 145 articles to determine final eligibility. During this phase, 54 articles were excluded because they did not meet the inclusion criteria (e.g., the wrong population, no healthy control group or the wrong outcome) or because they consisted solely of conference abstracts reporting preliminary findings, with no corresponding full‐length article identified. Conflicts at the full‐text stage were again resolved by discussion or adjudication by the third screener (R.F.).

**FIGURE 1:**
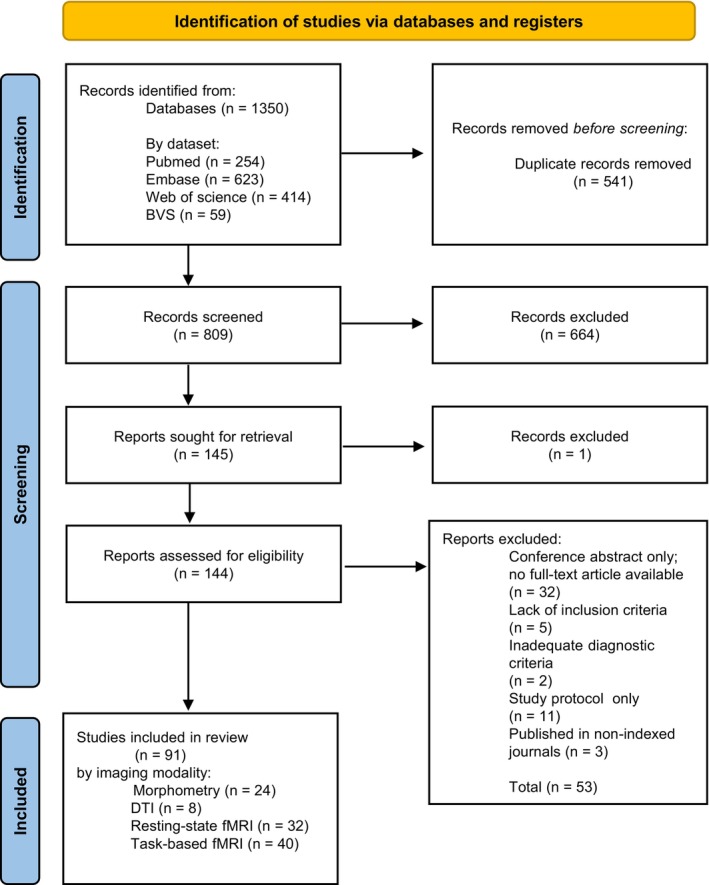
Study selection process. PRISMA 2020 flow diagram of study selection.

This final assessment process yielded 91 studies for inclusion in the systematic review. The complete study selection process is detailed in the PRISMA flow diagram (Figure 1).

### 2.6 Data Extraction

The data extraction for all 91 included studies was performed by one reviewer (F.F.) using a pre‐defined, standardized data charting form based on the study protocol. To ensure accuracy and minimize errors, a second reviewer (J.C.) independently verified all extracted data by checking the completed charting forms against the original full‐text articles. Any discrepancies, errors or ambiguities identified during the verification process were resolved through discussion and consensus between the two reviewers. If consensus could not be achieved, the third screener (R.F.) was consulted for adjudication.

The extracted information included: (1) Study identifiers (first author and publication year); (2) Study design (e.g., cross‐sectional, baseline data); (3) Participant characteristics (sample size for FM and HC groups, mean age and sex distribution); (4) Technical specifications (MRI scanner field strength in Tesla); (5) Methodological details (neuroimaging modality, primary analysis method and experimental paradigm for task‐fMRI studies); (6) Key findings (main reported differences between FM and HC groups); and (7) Clinical variables, whenever explicitly reported, including pain intensity, disease duration, FM impact/functional impairment, medication use, psychiatric and non‐psychiatric comorbidities, symptom‐scale data and correlations between neuroimaging findings and clinical measures.

For the visual synthesis of resting‐state fMRI connectivity findings, reported altered functional connectivity results were manually extracted from the included studies and organized as pairwise connections between the reported seed/ROI and target regions. Anatomical labels were harmonized into large‐scale cortical network categories based on the Yeo‐7 framework when applicable, with an additional category for subcortical/brainstem regions. These data were converted into an edge list and a network‐by‐network count matrix, in which each entry represented the number of reported altered connections between two categories. The chord diagram was generated using RAWGraphs (Mauri et al. 2017). Ribbon width reflects the frequency of reported altered connections between categories and should not be interpreted as effect size, statistical strength or direction of connectivity change.

### 2.7 Quality Assessment (Risk of Bias)

The methodological quality of the 91 included studies was assessed by a single reviewer (J.C.) using the modified Newcastle‐Ottawa Scale (NOS) for observational studies (Wells et al. 2011). The scale evaluates study quality based on three domains: Selection, Comparability and Outcome. For longitudinal or interventional studies, only the baseline pre‐intervention comparison was appraised, rather than the longitudinal or treatment‐related components of the parent study.

## 3 Results

### 3.1 Study Selection and Characteristics

The study selection process is summarized in the PRISMA flow diagram (Figure 1). From an initial 1350 citations identified, a total of 91 studies met the eligibility criteria after title/abstract screening and full‐text assessment and were included in the final synthesis.

These studies were categorized by primary neuroimaging modality; note that some studies contributed data to more than one category (e.g., reporting both structural and functional data). Of these, 24 studies reported on structural morphometry, 8 on Diffusion Tensor Imaging (DTI), 32 on resting‐state fMRI and 40 on task‐based fMRI.

Across the 91 included studies, FM diagnosis was most commonly based solely on the ACR 1990 criteria, followed by the later/modified ACR criteria only and combined ACR definitions. In a small number of studies, the diagnosis was reported in the extraction tables as ACR‐based, but the specific version was not fully specified.

The methodological quality of the 91 included studies was assessed using the modified Newcastle‐Ottawa Scale (NOS), as described in the methods. The overall quality of the evidence was variable: 74 studies were rated as high quality (7–9 stars), 16 as moderate quality (4–6 stars) and none as low quality (0–3 stars). Detailed scoring for each study is available as Table S1.

### 3.2 Synthesis of Findings

#### 3.2.1 Structural Brain Alterations: Morphometry

Morphometric studies most often reported reduced grey matter in FM, particularly in the cingulate, insular, temporal and prefrontal regions, although no single abnormality was uniformly replicated across studies. Across the 24 included studies, this pattern was chiefly reflected as grey matter volume (GMV) reduction in voxel‐based morphometry (VBM) studies and as cortical thinning or regional volume loss in surface‐based morphometry (SBM) and regional volumetry studies (Table 2).

**TABLE 2:** Structural morphometry findings in fibromyalgia.

Author/Year	Design	Sample size (FM/HC)	Mean age ± SD; sex ratio	Pain duration, years	Analysis method	MRI Scanner	Morphometry scope	Diagnostic criteria	Medication use reported	Key clinical values	Main findings	NOS Score
Robinson et al. (2011)	Cross‐sectional	14/11	FM: 43.1 ± 6.9/HC: 42.4 ± 9.8 (all female)	NR	VBM	3.0 T	Regional (pain‐related ROI)	ACR 1990	NR	Pain: VAS 2.9 (1.2) | Mood: BDI 13.2 (9.5), STAI‐S 33.9 (3.4), STAI‐T 43.1 (3.4)	↓ Insula, ↓ rACC, ↓ mACC	8/9 ★
Fallon et al. (2013)	Cross‐sectional	16/15	FM: 38.5 ± 8.5/HC: 39.4 ± 8.7 (all female)	9.1 ± 6.8	VBM + subcortical shape analysis	3.0 T	Both (whole‐brain + ROI)	ACR 1990	3–5‐day analgesic washout; stable meds allowed; acetaminophen allowed	FM: FIQ 63.37 ± 15.83 | Mood: BDI 19.50 ± 11.19	↓ Brainstem, ↓ Precuneus, ↑ SI	9/9 ★
Jensen et al. (2013)	Cross‐sectional	26/13	FM: 41.7 ± 8.9/HC: 41.2 ± 9.1 (all female)	11.0 ± 6.0	VBM + SBM	1.5 T	Both (whole‐brain + ROI)	ACR 1990	CNS‐active meds restricted	Pain: VAS 72 (14) mm | Mood: BDI 21 (11)	↓ rACC, ↓ lOFC, ↓ STG, ↓ MTG, ↓ SFG, ↓ Fusiform, ↑ SPC	9/9 ★
Ceko et al. (2013)	Cross‐sectional	28/28	FM: 46.1 ± 8.7/HC: 46.0 ± 8.6 (all female)	11.5 ± 8.7; younger: 8.8 ± 7.1; older: 12.1 ± 9.0	VBM + SBM	3.0 T	Whole‐brain	ACR 1990/2010	Opioids excluded; stable meds allowed; CNS‐active meds restricted	Pain: NRS 2.6 (2.7) | Mood: HADS‐A 10.3 (4.5), HADS‐D 5.7 (4.3)	↑ Insula, ↑ Putamen, ↑ GP, ↑ VLPFC; ↓ ACC, ↓ MPFC, ↓ DLPFC, ↓ OFC, ↓ PCC (age interaction)	9/9 ★
Robinson et al. (2015)	Cross‐sectional	14/12	FM: 44.1 (NR SD)/HC: 42.2 (NR SD) (all female)	NR	Volumetry	3.0 T	Regional (FreeSurfer ROI)	ACR NR	NR	NR	↓ Amygdala (L), ↓ Cerebellum (L), ↑ Thalamus (bilateral)	7/9 ★
McCrae et al. (2015)	Cross‐sectional	40/22	FM: 51.0 ± 13.5/HC: 43.4 ± 11.3 (all female)	11.9 ± 9.9	Volumetry	3.0 T	Regional (hippocampus)	ACR 1990	CNS‐active meds restricted	Mood: BDI 14.5 (10.2)	↓ Hippocampus (bilateral)	8/9 ★
Kutch et al. (2017)	Cross‐sectional	23/49	NR (all female)	NR	VBM	3.0 T	Whole‐brain	CMSI	NR	Mood: HADS‐A 7.1 ± 4.9, HADS‐D 6.7 ± 3.7	↑ SMA, ↑ MCC	6/9 ★
Fayed et al. (2017)	Cross‐sectional	12/10	FM: 41.7 ± 7.3 (2 M/10F)/HC: 39.5 ± 8.8 (sex NR)	NR	VBM	1.5 T	Whole‐brain	ACR NR; DSM‐IV	1‐week washout; no pharmacological treatment 1 week pre‐scan	Mood: HADS‐A 6.20 (2.8), HADS‐D 5.90 (3.8)	↑ ITG (bilateral)	8/9 ★
Pomares et al. (2017)	Cross‐sectional	26/25	FM: 61.0 ± 5.4/HC: 61.0 ± 7.6 (all female)	16.0 ± 9.0 (symptoms)	VBM	3.0 T	Both (whole‐brain + ROI)	ACR 2010	CNS‐active meds restricted	Pain: NRS 4.8 ± 2.2 | FM: FIQ 51 ± 18 | Mood: BDI 16 ± 10, HADS 16 ± 6	↓ PCC, ↓ Precuneus, ↓ ACC, ↓ Insula, ↓ MPFC, ↓ MTG, ↓ PreCG, ↓ Fusiform; ↑ Angular, ↑ Cuneus, ↑ PostCG	9/9 ★
Sundermann et al. (2019)	Baseline cross‐sectional	25/21	FM: 52.6 ± 11.1/HC: 52.2 ± 9.8 (all female)	15.8 ± 10.6	VBM	3.0 T	Both (whole‐brain + ROI)	ACR 2016	72‐h washout; CNS‐active meds restricted	Pain: NRS 3.80 ± 2.40	↑ PreCG (L), ↓ MTG (L), ↓ Angular/MOG (L)	8/9 ★
Feraco et al. (2020)	Cross‐sectional	12/12	FM: 43.2 (11F/1 M)/HC: 41.3 (11F/1 M)	NR	SBM + volumetry	3.0 T	Regional (pain‐related ROI)	ACR 1990	1‐week washout; acetaminophen allowed	NR	↓ ACC, ↓ PCC, ↓ Insula, ↓ IOG	6/9 ★
Leon‐Llamas et al. (2021)	Cross‐sectional	49/43	FM: 54.2 ± 10.1/HC: 53.4 ± 4.5 (all female)	19.4 ± 12.7 (symptoms)	Volumetry	3.0 T	Regional (hippocampus)	ACR 2010	NR	FM: FIQ 58.05 (17.85)	↓ Hippocampus subfields (bilateral)	8/9 ★
Kim et al. (2021)	Cross‐sectional	19/20	FM: 44.9 ± 8.3/HC: 45.0 ± 8.4 (all female)	3.0 ± 2.6	SBM + subcortical shape analysis	3.0 T	Both (whole‐brain + ROI)	ACR 1990	3‐day washout; CNS‐active meds restricted	Pain: VAS 52.8 (20.3) | Mood: BDI 19.0 (6.8)	↓ Thalamus (posterior), ↓ SFG (L)	8/9 ★
Tu et al. (2022a, 2022b)	Cross‐sectional	20/20	FM: 46.4 ± 12.4/HC: 42.1 ± 12.5 (all female)	5.2 ± 5.1	SBM	3.0 T	Whole‐brain	ACR 1990/2010	No analgesics on scan day	Pain: VAS 7.2 ± 1.6 | Mood: STAI 52.8 ± 20.1	↓ M1 (R), ↓ rACC (R), ↑ Precuneus (R)	7/9 ★
Mosch, Hagena, Herpertz, and Diers (2023); Mosch, Hagena, Herpertz, Ruttorf, and Diers (2023)	Cross‐sectional	23/21	FM: 50.5 ± 9.9/HC: 46.6 ± 13.1 (all female)	14.9 ± 11.8 (range 2–44)	VBM	3.0 T	Both (whole‐brain + ROI)	ACR 2016	Opioids withdrawn; no analgesics on scan day; 3‐day washout	FM: FIQ 60.2 ± 17.6	↓ MTG (bilateral), ↓ PHG (L), ↓ dACC (L), ↓ Putamen (R), ↓ Caudate (R), ↓ DLPFC (L); ↑ Cerebellum (bilateral), ↑ Thalamus (L)	7/9 ★
Liu et al. (2024)	Cross‐sectional	38/61	FM: 39.0 ± 11.7/HC: 31.4 ± 7.4 (majority female; ratio NR)	1.8 [0.5–5.0]	VBM	3.0 T	Whole‐brain	ACR NR	Stable meds allowed	Pain: VAS 4.5 [3–6] | Mood: HADS‐A 9.14 ± 3.56, HADS‐D 8.26 ± 3.12	↑ Cerebellum (L)	6/9 ★
de Oliveria Neto et al. (2024)	Cross‐sectional	33/33	FM: 41.7 ± 6.1/HC: 41.5 ± 6.0 (all female)	5.0 [Q1 2.0; Q3 8.0; range 0.4–48.0]	SBM	3.0 T	Whole‐brain	ACR 2016	Opioids excluded	NR	↑ PreCG, ↑ PostCG, ↑ Parietal (bilateral), ↑ Entorhinal; ↓ Anterior Insula (R)	8/9 ★
Agoalikum et al. (2025a)	Cross‐sectional	20/20	FM: 46.4 ± 12.5/HC: 42.1 ± 12.5 (all female)	5.2 ± 5.0	SBM	3.0 T	Whole‐brain	ACR 1990/2010	No painkillers on test day	Mood: STAI 52.8 ± 20.0	↑ Thalamus (bilateral), ↓ Putamen, ↓ Pallidum, ↓ Cerebellum (R), ↓ Calcarine, ↓ Amygdala (R), ↓ Insula (bilateral), ↓ Hippocampus (R)	6/9 ★
Agoalikum et al. (2025b)	Cross‐sectional	20/20	FM: 46.4 ± 12.5/HC: 42.1 ± 12.5 (all female)	5.2 ± 5.0	VBM	3.0 T	Whole‐brain	ACR 1990/2010	No painkillers on test day	Mood: STAI 52.8 ± 20.0	↑ Thalamus (L), ↓ Amygdala (R)	6/9 ★
Wu et al. (2025)	Baseline cross‐sectional	75/93	FM: 43.3 ± 10.5 (Subtype 1)/48.4 ± 10.1 (Subtype 2) (all female)	Subtype 1: 6.6 ± 5.8; Subtype 2: 4.3 ± 4.1	VBM + SBM	3.0 T	Both (whole‐brain + regional)	ACR 1990/2016	4‐week washout; no FM treatment ≥ 4 weeks	Pain: VAS 6.1 (1.3)/6.2 (1.8) | FM: FIQR 43.4 (17.2)/35.2 (20.1) | Mood: BDI‐II 9.6 (6.8)/7.9 (7.1)	Subtype 1: ↑ GMV (pain‐related); Subtype 2: no difference	7/9 ★
Kim, Kim, et al. (2015); Kim, Loggia, et al. (2015)	Cross‐sectional	42/63	FM: 45.3 ± 11.6/HC: 42.8 ± 13.7 (36F/6 M; 48F/15 M)	NR	VBM + structural covariance	3.0 T	Whole‐brain/network covariance	ACR 2010	NR	NR	↓ Frontal module covariance, ↑ Cerebellar covariance	8/9 ★
Harper et al. (2018)	Cross‐sectional	15/14	FM: 40.7 (NR SD) (all female)	NR	VBM	3.0 T	Both (whole‐brain + PAG)	ACR 1990	CNS‐active meds restricted	Pain: VAS 68.3 (13.4) | Mood: HADS‐D 4.9 (3.3), HADS‐A 6.5 (3.5)	↓ PAG	9/9 ★
Izuno et al. (2023)	Cross‐sectional	34/25	FM: 41.6 ± 7.4/HC: 39.5 ± 7.4 (all female)	NR	VBM	3.0 T	Both (whole‐brain + ROI)	ACR 2010	NR	Mood: HADS‐D 11.0 ± 4.4, HADS‐A 10.5 ± 4.2, STAI‐S 55.7 ± 10.8, STAI‐T 59.6 ± 12.8	↑ WMV R Temporal Pole; ↓ GMV Amygdala (trait anxiety); ↑ WMV Brainstem (depression); ↑ WMV Postcentral (magnification)	7/9 ★
Aster et al. (2022)	Cross‐sectional	43/40	FM: 53.5 ± 6.5/HC: 52.5 ± 6.7 (all female)	PNS: 16.8 ± 10.8; noPNS: 18.8 ± 12.7	SBM + volumetry	3.0 T	Both (whole‐brain + ROI)	ACR NR	3‐day washout; 4‐week washout; CNS‐active meds restricted	FM: FIQ 51.9 ± 12/42.3 ± 13.2 | Mood: STAI 47.1 ± 11.6/44.3 ± 13.3	↓ cortical volume in frontal, temporal, parietal cortex & insula; ↑ pericalcarine cortex	7/9 ★

*Note:* Cross‐sectional and baseline structural MRI studies comparing fibromyalgia (FM) and healthy controls (HC), reporting grey‐ and/or white‐matter morphometric differences. Direction of effects is shown as increases (↑) or decreases (↓) in FM relative to HC. Methodological quality is shown as NOS score★ (0–9), with higher scores indicating better methodological quality. Values are reported as mean ± SD, unless otherwise stated; median [IQR], range, subgroup‐specific values or symptom duration were retained as originally reported. References only in this table: (Fallon et al. 2013; Robinson et al. 2015; Fayed et al. 2017; Wu et al. 2025; Izuno et al. 2023).

Abbreviations: ACC, anterior cingulate cortex; AMYG, amygdala; ANG, angular gyrus; BDI, Beck Depression Inventory; bilat, bilateral; BS, brainstem; CAU, caudate; CER, cerebellum; dACC, dorsal ACC; DLPFC/dlPFC, dorsolateral PFC; F, female; FIQ/FIQR, Fibromyalgia Impact Questionnaire/Fibromyalgia Impact Questionnaire Revised; FM, fibromyalgia; FUS, fusiform gyrus; GMV, grey matter volume; GP/PAL, globus pallidus/pallidum; HADS, Hospital Anxiety and Depression Scale; HADS‐A/HADS‐D, Hospital Anxiety and Depression Scale anxiety/depression subscales; HC, healthy controls; HIP, hippocampus; IFG, inferior frontal gyrus; INS/aINS, insula/anterior insula; IOG, inferior occipital gyrus; ITG, inferior temporal gyrus; L, left; M, male; M1, primary motor cortex; mACC, mid‐ACC; MCC, mid‐cingulate cortex; MFG, middle frontal gyrus; MOG, middle occipital gyrus; MPFC/mPFC, medial PFC; MTG, middle temporal gyrus; NOS, Newcastle–Ottawa Scale; NR, not reported; NRS, numeric rating scale; OFC, orbitofrontal cortex; PAG, periaqueductal grey; PCC, posterior cingulate cortex; PFC, prefrontal cortex; pgACC, pregenual ACC; PHG, parahippocampal gyrus; PNS/noPNS, peripheral nerve involvement/no peripheral nerve involvement; PostCG, postcentral gyrus; PreCG, precentral gyrus; PUT, putamen; R, right; rACC, rostral ACC; ROI, region of interest; SBM, surface‐based morphometry; SD, standard deviation; SFG, superior frontal gyrus; SI/S1, primary somatosensory cortex; SII/S2, secondary somatosensory cortex; SMA, supplementary motor area; SPC/SPL, superior parietal cortex/lobule; STAI/STAI‐S/STAI‐T, State–Trait Anxiety Inventory/State/Trait subscales; STG, superior temporal gyrus; T, Tesla; THA, thalamus; VAS, visual analogue scale; VBM, voxel‐based morphometry; VLPFC/vlPFC, ventrolateral PFC; WMV, white matter volume.

Among the regions showing recurrent morphometric alterations, the anterior cingulate cortex (ACC) was the most frequently reported site of reduced GMV/cortical thickness, reported in seven studies (Ceko et al. 2013; Feraco et al. 2020; Jensen et al. 2013; Mosch, Hagena, Herpertz, and Diers 2023; Pomares et al. 2017; Robinson et al. 2011; Tu et al. 2022a). Recurrent reductions were also identified in the insula (Agoalikum et al. 2025a; Aster et al. 2022; Feraco et al. 2020; de Oliveria Neto et al. 2024; Robinson et al. 2011), temporal regions—particularly the superior and middle temporal gyri (STG and MTG) (Aster et al. 2022; Jensen et al. 2013; Mosch, Hagena, Herpertz, and Diers 2023; Pomares et al. 2017; Sundermann et al. 2019)—as well as in prefrontal (PFC) and orbitofrontal cortices (OFC) (Aster et al. 2022; Ceko et al. 2013; Jensen et al. 2013; Mosch, Hagena, Herpertz, and Diers 2023; Pomares et al. 2017). Additional recurrent decreases were reported in the posterior cingulate cortex (PCC) (Ceko et al. 2013; Feraco et al. 2020; Pomares et al. 2017), amygdala (Agoalikum et al. 2025a, 2025b; Robinson et al. 2011) and hippocampus (Agoalikum et al. 2025b; Leon‐Llamas et al. 2021; McCrae et al. 2015). Although insular abnormalities were repeatedly observed, subregional localization was inconsistently reported across studies: Robinson et al. (2011) described reduced grey matter in the mid‐insula; de Oliveria Neto et al. (2024) reported reduced cortical thickness in the right anterior insula.

These patterns varied somewhat according to analytic approach. VBM studies more often reported grey matter volume reductions in cingulate, insular, temporal and prefrontal regions (Mosch, Hagena, Herpertz, and Diers 2023; Pomares et al. 2017; Robinson et al. 2011; Sundermann et al. 2019), whereas SBM and regional volumetry studies more commonly described cortical thinning or regional volume loss in cingulate, insular, hippocampal and occipital regions (Agoalikum et al. 2025a; Leon‐Llamas et al. 2021; McCrae et al. 2015; de Oliveria Neto et al. 2024; Tu et al. 2022a). Studies using mixed morphometric approaches likewise identified alterations in cingulate, prefrontal, temporal, insular and subcortical regions (Aster et al. 2022; Ceko et al. 2013; Feraco et al. 2020; Jensen et al. 2013; Kim et al. 2021). However, because several of these reports did not clearly separate which findings derived from VBM versus SBM or volumetry, their results should be interpreted cautiously at the method‐specific level.

Findings of increased grey matter were less common, with the thalamus being the region most often described as enlarged (Agoalikum et al. 2025a, 2025b; Mosch, Hagena, Herpertz, and Diers 2023; Robinson et al. 2011). However, some regions showed bidirectional findings across studies, including the insula, thalamus and cerebellum. Specifically, insular reductions were reported in five studies (Agoalikum et al. 2025a; Feraco et al. 2020; de Oliveria Neto et al. 2024; Pomares et al. 2017; Robinson et al. 2011), whereas Ceko et al. (2013) described an increase; thalamic increases were reported in four studies (Agoalikum et al. 2025a, 2025b; Mosch, Hagena, Herpertz, and Diers 2023; Robinson et al. 2011), whereas Kim et al. (2021) reported a reduction; and the cerebellum showed both increases (Liu et al. 2024; Mosch, Hagena, Herpertz, and Diers 2023) and decreases (Agoalikum et al. 2025a; Robinson et al. 2011). Together, these findings indicate substantial variability in both the direction and anatomical distribution of morphometric effects.

This variability occurred alongside differences in morphometric scope and analytic strategy, as some studies used whole‐brain VBM/SBM approaches, whereas others combined whole‐brain and region‐specific analyses.

Clinical characterization was also uneven across morphometry studies. Psychiatric comorbidities were more often addressed through exclusion criteria or symptom scales than through systematic prevalence reporting. Several studies excluded current or severe psychiatric disorders, whereas others quantified depressive and/or anxiety burden using the Hospital Anxiety and Depression Scale (HADS), Beck Depression Inventory (BDI), State–Trait Anxiety Inventory (STAI), Center for Epidemiologic Studies Depression Scale (CES‐D), Patient Health Questionnaire‐9 (PHQ‐9), Geriatric Depression Scale‐15 (GDS‐15) or Hamilton scales (Izuno et al. 2023; McCrae et al. 2015; de Oliveria Neto et al. 2024; Pomares et al. 2017; Robinson et al. 2011; Tu, Wang, et al. 2023). Explicit prevalence reporting was uncommon, although Mosch, Hagena, Herpertz, and Diers (2023) described previous major depressive episodes and additional cases of generalized anxiety disorder and/or PTSD in the FM group. Overall, the available symptom‐scale data suggest that affective burden was not uniformly low across samples, supporting substantial clinical heterogeneity (Ceko et al. 2013; Fallon et al. 2013; Izuno et al. 2023; Jensen et al. 2013; Kim et al. 2021; Liu et al. 2024; McCrae et al. 2015; de Oliveria Neto et al. 2024; Pomares et al. 2017; Tu, Wang, et al. 2023).

Non‐psychiatric comorbidities, medication exposure and covariate handling were likewise inconsistently reported. Many studies excluded neurological disorders, other chronic pain conditions, autoimmune or inflammatory diseases, endocrine abnormalities or major medical illnesses (Aster et al. 2022; Ceko et al. 2013; Feraco et al. 2020; de Oliveria Neto et al. 2024), whereas medication‐related restrictions ranged from same‐day analgesic avoidance to temporary discontinuation of centrally acting drugs, antidepressants, anticonvulsants, benzodiazepines or opioids (Aster et al. 2022; Feraco et al. 2020; Jensen et al. 2013; Kim et al. 2021; Mosch, Hagena, Herpertz, and Diers 2023). Detailed reporting of ongoing treatment exposure remained variable.

Regarding clinical variables, morphometry studies variably considered disease severity using measures such as pain intensity, disease or symptom duration, FM impact/severity, WPI/SSS, experimental pain sensitivity, perceived stress and affective‐cognitive symptom scales. Overall, these variables did not yield a consistent clinical–morphometric pattern. Some studies reported associations between structural alterations and longer disease duration, experimental pain sensitivity, perceived stress or cognitive‐affective dimensions of pain, such as catastrophizing, rumination and helplessness (Agoalikum et al. 2025a; Ceko et al. 2013; Jensen et al. 2013). However, baseline pain intensity and global FM impact were not consistently associated with morphometric findings. Furthermore, only a subset of studies incorporated affective or clinical variables, such as depression, anxiety, pain severity or symptom duration, as covariates in morphometric analyses (Aster et al. 2022; McCrae et al. 2015; Tu, Wang, et al. 2023). Detailed study‐level information on comorbidities, medication use, symptom scales and disease severity is provided in (Table S2).

#### 3.2.2 Structural Brain Alterations: Diffusion MRI and Structural Connectivity

Diffusion MRI and structural connectivity studies were few and methodologically diverse, and did not support a stable white‐matter pattern in FM. Across the eight included studies, three focused primarily on diffusion tensor imaging (DTI)‐derived microstructural indices—fractional anisotropy (FA), mean diffusivity (MD), radial diffusivity (RD) and axial diffusivity (AD)—and yielded markedly inconsistent findings. Ceko et al. (2013) and Kim et al. (2014) reported patterns, including lower FA together with higher RD, mainly across the corpus callosum and other major white‐matter tracts, which may suggest altered white‐matter microstructure, although these scalar diffusion measures do not support a specific linear biological interpretation. In contrast, Tu et al. (2022b) reported the opposite pattern, with increased FA and reduced MD, RD and AD across multiple white‐matter tracts, including the corpus callosum, corona radiata, internal capsule, corticospinal tract, posterior thalamic radiation, cerebellar peduncle, sagittal stratum and superior fronto‐occipital fasciculus. Notably, after adjustment for anxiety and depression, most between‐group differences in that study were attenuated, except for residual AD reductions in selected tracts.

Structural connectivity findings were similarly heterogeneous and should not be interpreted as directly comparable, as the studies included relied on distinct analytic frameworks. One tractography‐based study reported reduced whole‐brain white matter fibre counts, suggesting reduced large‐scale structural connectivity. Tu, Li, et al. (2023), using graph‐theoretical analysis, found reduced local efficiency and clustering coefficient, indicating a less efficiently organized structural network with weaker local integration and segregation. By contrast, Mosch, Hagena, Herpertz, and Diers (2023) used a diffusion MRI connectometry approach based on tract‐specific FA mapping and reported regionally divergent effects, with reduced FA in the left corticospinal tract but increased FA in the right parolfactory cingulum. In contrast, Fayed et al. (2010) and Aster et al. (2022) did not identify global structural connectivity disruptions. Overall, diffusion microstructure and structural connectivity findings were sparse, methodologically diverse and directionally inconsistent, preventing identification of a stable white‐matter signature in FM.

Clinical characterization was also uneven across diffusion and structural connectivity studies, with variable reporting of psychiatric symptoms, medication exposure and covariate adjustment, which may have contributed to the inconsistency of the findings. Detailed characteristics and findings for these studies are available in Table 3.

**TABLE 3:** Diffusion MRI and structural connectivity findings in fibromyalgia.

Author/Year	Design	Sample size (FM/HC)	Mean age ± SD; sex ratio	Pain duration, years	Medication use reported	Diagnostic criteria	Scanner	DTI method	Analysis scope	Main findings	Clinical associations	NOS score
Fayed et al. (2010)	Cross‐sectional	10/10	FM: 40.0 ± 6.2 (8F/2M)/HC: 37.8 ± 8.7 (8F/2M)	1.6 ± 0.3	Pharmacologic treatment discontinued 1 week before scan; tramadol, pregabalin, paracetamol, duloxetine and benzodiazepine reported.	ACR primary FM (year NR)	1.5 T	DTI + DWI	Regional ROI‐based pain network	No significant between‐group differences in FA or ADC across predefined pain‐related ROIs.	None reported.	6/9 ★
Ceko et al. (2013)	Cross‐sectional	28/28	FM: 46.1 ± 8.7/HC: 46.0 ± 8.6 (all female)	11.5 ± 8.7	Stable medication allowed; NSAIDs, antidepressants, muscle relaxants, anticonvulsants, cannabinoids and triptans reported; opioids excluded.	ACR 1990	3.0 T	DTI (TBSS)	Whole‐brain TBSS	Older FM patients showed ↓ FA and ↑ RD in the posterior corpus callosum near the PCC; younger FM patients showed mild ↑ FA in the anterior internal capsule.	↓ FA in the callosal–PCC tract was associated with higher pain sensitivity and lower DMN connectivity.	9/9 ★
Kim et al. (2014)	Cross‐sectional	19/18	FM: 44.9 ± 8.3/HC: 44.7 ± 8.8 (all female)	3.0 ± 2.6	Analgesics, antidepressants and anticonvulsants stopped ≥ 3 days before the experiment.	ACR 1990	3.0 T	DTI with TBSS + probabilistic tractography	Whole‐brain TBSS + regional tractography	↓ FA in the left corpus callosum adjacent to the cingulum/ACC, with ↑ RD and a trend toward ↓ AD; no MD differences.	Lower FA correlated with higher sensory pain scores; not associated with VAS pain, FIQ or pain duration. Tractography linked the affected cluster to bilateral sensorimotor and premotor cortices.	7/9 ★
Kim, Kim, et al. (2015)	Cross‐sectional	42/63	FM: 45.3 ± 11.6 (36F/6M)/HC: 42.8 ± 13.7 (48F/15M)	NR	NR	ACR 2010	3.0 T	Probabilistic tractography	Whole‐brain structural network (ROI‐to‐ROI)	Reduced white‐matter fibre counts between frontal and parietal regions, with increased cerebellar–temporal/parietal connectivity in FM.	Greater cerebellar–temporal/parietal connectivity was associated with lower pain sensitivity, suggesting compensatory reorganization.	8/9 ★
Tu et al. (2022a, 2022b)	Cross‐sectional	20/20	FM: 46.4 ± 12.4/HC: 42.1 ± 12.5 (all female)	5.2 ± 5.1	Participants with FM were instructed not to use pain medication on the day of scan.	ACR 2010	3.0 T	DTI (TBSS)	Whole‐brain voxelwise TBSS	FM showed ↑ FA and ↓ MD/RD/AD across widespread white‐matter tracts; after adjusting for anxiety/depression, only ↓ AD in the internal capsule, corona radiata and cerebellar peduncle remained significant.	No significant associations with pain intensity, catastrophizing or disease duration after controlling for affective symptoms.	7/9 ★
Aster et al. (2022)	Cross‐sectional	43/40	FM: 53.5 ± 6.5/HC: 52.6 ± 6.7 (all female)	PNS: 16.8 ± 10.8; noPNS: 18.8 ± 12.7	Off pain medication for 3 days; anticonvulsants, antihistamines, muscle relaxants and benzodiazepines excluded within 4 weeks.	ACR 2010 (WPI ≥ 7; SSS ≥ 5)	3.0 T	DTI (ROI‐wise FA analysis)	Whole‐brain white‐matter integrity + ROI subgroup analysis	No significant between‐group differences in FA or ADC across ROIs; DTI metrics did not reveal measurable white‐matter abnormalities in FM.	None reported.	7/9 ★
Mosch, Hagena, Herpertz, and Diers (2023); Mosch, Hagena, Herpertz, Ruttorf, and Diers (2023)	Cross‐sectional	22/21	FM: 50.5 ± 9.9/HC: 46.6 ± 13.1 (all female)	14.9 ± 11.8	No pain medication on examination day; opioid use suspended ≤ 3 days before MRI; psychotropic medication excluded.	ACR 2016	3.0 T	Diffusion MRI connectometry	Whole‐brain connectometry + ROI‐wise correlations	↓ FA in corticospinal, fornix, thalamic radiation, medial lemniscus and cerebellar pathways, with ↑ FA in the parolfactory cingulum, cerebellum, forceps, tapetum and right IFOF.	FA alterations were associated with pain severity, pain duration, heat pain threshold, depression symptoms and general activity.	7/9 ★
Tu, Wang, et al. (2023); Tu, Li, et al. (2023)	Cross‐sectional	20/20	FM: 46.4 ± 12.4/HC: 42.1 ± 12.5 (all female)	5.2 ± 5.1	Individuals with FM were instructed not to use painkillers on the day of scan.	ACR 1990 + 2010	3.0 T	Probabilistic tractography	Whole‐brain structural network (graph theory + NBS)	FM showed ↓ clustering coefficient, local efficiency, hierarchy and synchronization, with ↑ normalized characteristic path length; NBS identified a reduced subnetwork linking DLPFC, SMA, M1, insula, thalamus, caudate and PCC.	No significant correlations with pain, anxiety, depression or catastrophizing.	6/9 ★

*Note:* Cross‐sectional and baseline diffusion MRI/structural connectivity studies comparing fibromyalgia (FM) and healthy controls (HC), reporting white‐matter microstructural and network‐level structural abnormalities. Direction of effects is shown as increases (↑) or decreases (↓) in FM relative to HC. Analysis scope indicates whether the study used regional/ROI‐based analyses, whole‐brain approaches or both. Methodological quality is shown as NOS score★ (0–9), with higher scores indicating better methodological quality. Values are reported as mean ± SD unless otherwise stated; median [IQR], range, subgroup‐specific values or symptom duration were retained as originally reported.

Abbreviations: ACC, anterior cingulate cortex; AD, axial diffusivity; ADC, apparent diffusion coefficient; DLPFC, dorsolateral prefrontal cortex; DMN, default mode network; DTI, diffusion tensor imaging; DWI, diffusion‐weighted imaging; F, female; FA, fractional anisotropy; FIQ, Fibromyalgia Impact Questionnaire; FM, fibromyalgia; HC, healthy controls; IFOF, inferior fronto‐occipital fasciculus; M, male; M1, primary motor cortex; MD, mean diffusivity; NBS, network‐based statistics; noPNS, subgroup without peripheral nerve involvement; NOS, Newcastle–Ottawa Scale; NR, not reported; PCC, posterior cingulate cortex; PNS, peripheral nerve involvement subgroup; RD, radial diffusivity; ROI, region of interest; SD, standard deviation; SMA, supplementary motor area; SSS, Symptom Severity Scale; TBSS, tract‐based spatial statistics; VAS, visual analogue scale; WPI, Widespread Pain Index.

#### 3.2.3 Functional Brain Alterations: Resting‐State fMRI

A total of 32 studies investigated functional brain alterations using resting‐state fMRI, employing methods such as functional connectivity (FC), local activity metrics, including amplitude of low‐frequency fluctuation (ALFF) and regional homogeneity (ReHo), and network‐level analyses (Table 4). The most frequently implicated systems were the default mode network (DMN; centred on medial prefrontal, posterior cingulate/precuneus and inferior parietal regions), the salience network (SN; mainly anterior insula and dorsal/anterior mid‐cingulate cortex), and connectivity involving subcortical and brainstem pain‐modulatory nodes such as the periaqueductal grey (PAG), thalamus and basal ganglia.

**TABLE 4:** Resting‐state fMRI findings in fibromyalgia.

Author/Year	Design	Sample size (FM/HC)	Mean age ± SD; sex ratio	Pain duration, years	Medication use reported	Diagnostic criteria	Scanner	Analysis method	Key clinical values	Main findings	Clinical associations	NOS score
Napadow et al. (2010)	Cross‐sectional	18/18	FM: 38.9 ± 10.8/HC: 36.1 ± 15.3 (all female)	≥ 1	Opioids/narcotics excluded; no new medications/treatments	ACR 1990	3.0 T	ICA (dual regression)	Pain: spontaneous pain at scan 4.8 ± 2.4	↑ DMN–INS; ↑ rEAN–INS.	Increased DMN–insula and rEAN–insula connectivity was associated with higher spontaneous pain intensity and FIQ scores.	8/9 ★
Cifre et al. (2012)	Cross‐sectional	9/11	FM: 52.3 ± 8.9/HC: 49.0 ± 12.1 (mostly female; FM 8F/1M, HC 9F/11M)	26.8 ± 17.4	Antidepressants (*n* = 8); analgesics/relaxants/NSAIDs (*n* = 4); anxiolytics (*n* = 8)	ACR 1990	3.0 T	Partial correlation	Pain: WHYMPI pain intensity 4.5 (0.9) | Mood: BDI 29.78 (12.07)	↑ ACC–INS, ACC–PUT, M1–SMA; ↓ ACC–PAG, THA–INS, SII–M1; excitatory/inhibitory imbalance.	Reduced PAG–thalamus and PAG–ACC connectivity correlated with higher depression scores, suggesting impaired descending modulation linked to affective symptoms.	8/9 ★
Jensen et al. (2013)	Cross‐sectional	26/13	FM: 38.0 ± 7/HC: 34.0 ± 9 (all female)	11.0 ± 6.0	CNS‐active and pain treatments restricted/discontinued	ACR 1990	1.5 T	ReHo	Pain: VAS 72 (14) mm | Mood: BDI 21 (11)	↓ ReHo in rACC/MPFC; overlap with structural atrophy.	Lower ReHo in the rACC was related to longer disease duration.	9/9 ★
Ceko et al. (2013)	Cross‐sectional	28/28	FM: 48.8 ± 7.7/HC: 48.8 ± 7.7 (all female)	11.5 ± 8.7	Stable meds allowed; opioids excluded; NSAIDs/antidepressants/muscle relaxants/anticonvulsants/cannabinoids/triptans reported	ACR 1990	3.0 T	Seed‐based	Pain: VAS/NRS 2.6 (2.7), range 0–9 | Mood: HADS‐A 10.3 (4.5), HADS‐D 5.7 (4.3)	↓ PCC–MPFC, PCC–INS; ↑ NACC–DLPFC; reward–modulatory shift.	Reduced PCC connectivity correlated with higher pain sensitivity in older FM, while increased insula–NAc coupling was associated with lower catastrophizing in younger FM.	9/9 ★
Ichesco et al. (2014)	Cross‐sectional	18/18	FM: 35.8 ± 12.0/HC: 32.3 ± 11.3 (all female)	3.9 ± 3.7	Opioids/narcotics excluded; no new treatments between consent and scan	ACR 1991	3.0 T	Seed‐based	Pain: SF‐MPQ VAS 4.4 ± 2.3	↑ INS–PCC, INS–STG; limbic–somatosensory hyperconnectivity.	Stronger insula–PCC and mid‐insula–cingulate connectivity were associated with lower pain thresholds and higher clinical pain intensity.	8/9 ★
Flodin et al. (2015)	Baseline cross‐sectional	14/11	FM: 48.4 ± NR/HC: 41.8 ± NR (all female; SD NR)	7.3 ± 4.0	NR	ACR 1990	3.0 T	Seed‐based	Pain: SF‐36 BP 37.00 ± 9.70 | FM: FIQ 60.8 ± 11.8	↓ INS–M1/S1, ↓ SMG–S1/M1; impaired salience–sensorimotor integration.	Lower insula–sensorimotor connectivity was linked to higher FIQ scores, indicating greater symptom severity.	8/9 ★
Kim, Kim, et al. (2015)	Cross‐sectional	35/14	FM: 44.9 ± 12.0 (32F/3M)/HC: 44.2 ± 14.3 (10F/4M)	9.76 ± 8.56 (from diagnosis)	Opioids and recreational drugs excluded; antidepressants 49%; muscle relaxants 16%; benzodiazepines 9%	ACR 2010	3.0 T	Seed‐based	Pain: clinical pain at MRI 29.9 ± 22.6 (0–100) | Mood: BDI 13.5 ± 8.2	↓ S1–S1; ↑ S1–INS during pain; salience hyperresponsivity.	Stronger S1–insula connectivity during pain was correlated with higher pain ratings, catastrophizing and reduced HRV.	8/9 ★
Truini et al. (2016)	Cross‐sectional	20/15	FM: 28–67/HC: 26–65 (FM 19F/1M; HC 13F/2M; SD NR)	NR	Antidepressants, opioids and antiepileptics excluded; rescue analgesics avoided 72 h pre‐scan	ACR 1990 and 2010	3.0 T	Seed‐based	NR	↑ PAG–ACC/INS/AMYG; ↓ PAG–RVM; descending modulation deficit.	Increased PAG connectivity with insula and IFG correlated with higher pain and tender point counts, while reduced PAG–frontal coupling was related to higher depression scores.	8/9 ★
Ichesco et al. (2016)	Cross‐sectional	16/15	FM: 38.5 ± 12.1/HC: 39.9 ± 13.0 (all female)	NR	Opioids/narcotics excluded; no new treatments; OTC analgesics withheld ≥ 8 h	ACR 1990	3.0 T	Seed‐based	Pain: VAS 52.6 ± 20.5 | Mood: HADS‐A 10.0 ± 4.1, HADS‐D 6.9 ± 3.9, PANAS positive 28.6 ± 8.9, PANAS negative 23.2 ± 7.9	↑ PAG–DMN (PCC/MPFC); ↓ PAG–RVM; reduced brainstem inhibition.	Increased PAG–DMN and PAG–amygdala connectivity correlated with higher pain and catastrophizing, whereas reduced PAG–RVM coupling was linked to longer pain duration.	8/9 ★
Fallon et al. (2016)	Cross‐sectional	16/15	FM: 38.5 ± 8.5/HC: 39.4 ± 8.7 (all female)	9.13 ± 6.80	5 medication‐free; 11 on permissible meds or after ≥ 3‐day washout	ACR 1990	3.0 T	Seed‐based	FM: FIQ 62.37 ± 15.84 | Mood: BDI 19.5 ± 11.19	↓ PCC–PHG; ↑ PCC–aMCC, IPL–HF; DMN reorganization.	Stronger PCC–aMCC connectivity correlated with higher tenderness and depression scores, whereas reduced PCC–parahippocampal coupling was related to longer symptom duration.	8/9 ★
Kutch et al. (2017)	Cross‐sectional	23/49	NR (all female)	NR	NR	ACR 1990	3.0 T	ICA + ROI‐to‐ROI	Pain: BPI severity 4.5 ± 2.0 | Mood: HADS‐A 7.1 ± 4.9, HADS‐D 6.7 ± 3.7	↑ SN–SMN; ↑ INS–frontal/temporal/limbic coupling.	No significant correlations between salience–sensorimotor connectivity and clinical symptoms were reported.	6/9 ★
Jarrahi et al. (2017)	Cross‐sectional	8/11	FM: 40.4 ± 9.9/HC: 43.1 ± 13.7 (all female)	NR	Opioids excluded	ACR 2011 modified criteria	3.0 T	Group ICA	NR	↑ spectral power in SMN/MPFC/SN/subcortical/cerebellar; ↓ posterior DMN high‐freq.	Pain ratings during the cold‐pressor condition modulated sensorimotor and prefrontal networks, reflecting altered autonomic–limbic integration.	7/9 ★
Coulombe et al. (2017)	Cross‐sectional	23/16	FM: 50.6 ± 8.1/HC: 49.8 ± 11.0 (all female)	NR	Antidepressants excluded; recent pain‐medication changes excluded	ACR 1990	3.0 T	Spectral power (FFT)	Pain: BPI 22.3 ± 7.5 | FM: FIQ 60.3 ± 15.8 | Mood: HADS‐A 10.4 ± 3.8, HADS‐D 7.3 ± 3.4	↑ PAG–LNG/RSC/HIP; ↓ PAG–PFC/SMA; limbic–VIS ↑, top‐down ↓.	Reduced PAG–PMC and PAG–PFC connectivity correlated with higher FIQ and catastrophizing, suggesting weaker descending control in more symptomatic patients.	8/9 ★
Jarrahi et al. (2018)	Cross‐sectional	8/11	FM: 40.4 ± 9.9/HC: 43.1 ± 13.7 (all female)	NR	NR	ACR 2011	3.0 T	Seed‐based	NR	Altered SOMATO–SMN coupling; ↑ DAN–brainstem; CEN under‐engagement.	No behavioural correlations were reported, though results support impaired descending inhibition.	8/9 ★
Kaplan et al. (2019)	Cross‐sectional	51/46	FM: 39.0 ± 11.0/HC: 38.8 ± 12.2 (all female)	≥ 6 months	Medication history collected; opioids/narcotics excluded in validation cohort; no new medications/treatments in validation cohort	ACR 1990	3.0 T	Group ICA + FNC	Pain: VAS 4.88 ± 2.24	↑ EC in INS/M1/S1/SMA/PCC; hub and rich‐club reorganization.	Increased eigenvector centrality in insula, M1, S1 and STG correlated with higher clinical pain; posterior insula centrality mediated the link between Glx and pain intensity.	8/9 ★
Kong et al. (2019)	Baseline cross‐sectional	21/20	FM: 53.1 ± 11.6 (20F/1M)/HC: 52.9 ± 11.1 (19F/1M)	NR	Regular medications allowed	ACR 1990 and 2010	3.0 T	Graph theory	FM: FIQR 45.1 ± 18.6 | Mood: BDI‐II 19.71 ± 11.12	↑ DLPFC–rACC/MPFC; enhanced cognitive–affective coupling (CEN).	No baseline correlations reported; post‐intervention findings were excluded.	9/9 ★
Pando‐Naude et al. (2019)	Cross‐sectional	20/20	FM: 46.4 ± 12.4/HC: 42.1 ± 12.5 (all female)	NR	No painkillers on scan day	ACR 1990 and 2010	3.0 T	Seed‐based	Mood: STAI 52.8 ± 20.1, CES‐D 31 ± 13.7	↑ pain‐matrix–DMN; ↓ pain‐matrix–INS/SMN; intrinsic coupling disruption.	Stronger pain matrix–DMN connectivity was linked to higher pain intensity, whereas reduced pain matrix–insula connectivity correlated with lower pain.	9/9 ★
van Ettinger‐Veenstra et al. (2020)	Cross‐sectional	31/28	FM: 39.23 ± 11.4/HC: 42.6 ± 10.2 (all female)	NR	NSAIDs, pain and sleep meds washed out 48 h; standard therapy continued	ACR 2010 criteria	3.0 T	ROI‐to‐ROI + Seed‐based	Pain: NRS 5.68 ± 1.83 | Mood: HADS‐D 6.00 ± 3.61, HADS‐A 7.81 ± 4.04	↑ rIPS–rINS; ↑ rIPS–preCG; anxiety/pain‐modulated CEN–salience coupling.	Stronger rIPS–insula connectivity correlated with higher pain intensity, while increased rIPS–precentral coupling was associated with higher anxiety scores (HADS‐A).	9/9 ★
Jung et al. (2020)	Cross‐sectional	38/16	FM: 42.1 ± 11.6/HC: 44.9 ± 16.3 (all female)	≥ 1	NSAIDs/pregabalin/duloxetine allowed; OTC pain meds on scan day and narcotics within 48 h excluded; daily narcotics, marijuana, stimulants excluded	ACR 2010 criteria	3.0 T	Seed‐based + fALFF	Pain: BPI 5.37 (1.50) | FM: FIQ 50.18 (18.55) | Mood: HADS‐D 7.26 (3.55), HADS‐A 7.68 (4.26)	↓ INS–PUT; ↓ fALFF; salience–basal ganglia decoupling.	Lower insula–putamen connectivity was associated with higher FIQ and pain interference, while reduced spontaneous activity in these regions correlated with greater depression.	8/9 ★
Kong et al. (2021)	Baseline cross‐sectional	20/19	FM: 51.6 ± 11.6/HC: 52.3 ± 10.4 (mostly female)	NR	Regular medications allowed; 3/20 maintained pharmacological treatment	ACR 1990 and 2010	3.0 T	Seed‐based	FM: FIQR 45.9 ± 17.6 | Mood: BDI‐II 17.7 ± 9.3	↓ HYP–AMYG/THA; ↑ HYP–SUBCG; hypothalamic dysregulation.	No baseline correlations reported between connectivity and clinical variables.	8/9 ★
Larkin et al. (2021)	Cross‐sectional	70/37	NR (all female; 18–75 years; age‐matched ±2 years)	Discovery: ≥ 6 months; replication: ≥ 1	No new FM treatments/medications in replication cohort; opioids/narcotics excluded	ACR 1990	3.0 T	Graph theory	Pain: VAS tertiles 31.04 ± 12.92/58.48 ± 5.99/76.87 ± 7.32 (overall mean NR)	↓ NMI; unstable community topology; ↓ DMN–SN coupling.	Reduced network stability (NMI) correlated with higher pain intensity, indicating disrupted DMN–salience coupling in more symptomatic patients.	7/9 ★
Kim et al. (2021)	Cross‐sectional	19/20	FM: 44.9 ± 8.3/HC: 45.0 ± 8.4 (all female)	35.6 ± 31.1 months	Analgesics, antidepressants and anticonvulsants stopped ≥ 3 days; analgesics/muscle relaxants/NSAIDs, antidepressants and anticonvulsants reported	1990 ACR criteria	3.0 T	Seed‐based	Pain: VAS 52.8 (20.3) | Mood: BDI 19.0 (6.8)	↑ THA–IPL; ↓ THA–PAG/rACC; thalamocortical control change.	Stronger thalamocortical coupling correlated with lower pain thresholds, reflecting heightened pain sensitivity.	8/9 ★
Balducci et al. (2022)	Cross‐sectional	33/33	FM: 41.7 ± 6.1/HC: 41.5 ± 6.0 (all female)	NR	Opioids excluded; rescue analgesics/benzodiazepines stopped ≥ 24 h; current medication recorded	ACR 1990 + 2016 ACR criteria	3.0 T	Seed‐based	NR	Descriptive ICA dataset; DMN/SN/CEN identified.	No correlations reported (open resource dataset).	7/9 ★
Park, Baker, et al. (2022); Park et al. (2022a, 2022b)	Cross‐sectional (multi‐site)	32/37	FM: 48.1 ± 7.5/HC: 45.6 ± 8.9 (Stanford); FM: 34.9 ± 11.7/HC: 41.1 ± 9.3 (Duke) — all female	9.08 ± 7.35 (range 9 months‐28 years)	Opioid‐naive; no opioids within 90 days; other pain/mood medications reported	ACR 2011	3.0 T	Seed‐based	NR	↓ NACC–MPFC; ↓ NACC–PUT/THA/VP; mesolimbic hypoconnectivity.	Reduced NAcc–mPFC connectivity was associated with higher anxiety but not with pain or fatigue.	9/9 ★
Liu et al. (2024a)	Cross‐sectional	38/61	FM: 39.0 ± 11.7/HC: 31.4 ± 7.4 (majority female; ratio NR)	1.75 [0.5–5]	Stable or as‐needed NSAIDs allowed; NSAIDs, duloxetine, pregabalin and sedatives reported	ACR 2016	3.0 T	ICA	Pain: VAS 4.5 [3–6] | FM: WPI 9.17 ± 2.12, SSS 7.31 ± 1.69 | Mood: HADS‐A 9.14 ± 3.56, HADS‐D 8.26 ± 3.12	↓ DDMN–CAU (vs. AS); no FM–HC difference.	No significant correlations with pain or affective measures; reduced FPN connectivity was associated with higher age.	6/9 ★
Elkana and Beheshti (2025)	Cross‐sectional	33/33	FM: 43 ± NR/HC: 43 ± NR (all female)	2.00 [1.00–6.00]	Daily/crisis medication count recorded; no washout reported	ACR 2010	3.0 T	ROI‐to‐ROI + ML	Pain: 47.00 [38.00–66.00] | FM: FIQ 35.47 [29.99–39.97] | Mood: HAM‐D 16.00 [11.00–21.00], HAM‐A 23.00 [17.00–27.00]	Altered DMN/SMN/VIS/DAN; ML AUC 0.65; predictive DMN–VIS–DAN links.	Functional connectivity in DMN, VIS and DAN networks correlated with higher symptom severity, anxiety and depression.	6/9 ★
Agoalikum et al. (2025a, 2025b)	Cross‐sectional	20/20	FM: 46.4 ± 12.5/HC: 42.1 ± 12.5 (all female)	5.2 ± 5.0	No painkillers on test day	ACR 1990 and 2010	3.0 T	Granger causality	Pain: PI 7.2 ± 1.6 | Mood: STAI 52.8 ± 20.0, CES‐D 30.6 ± 13.7	↓ THA → CAU/FUS/HIP; ↑ inflow to THA; thalamocortical control shift.	Greater thalamic inflow correlated with higher pain intensity, catastrophizing, rumination and anxiety.	6/9 ★
Flodin et al. (2014)	Cross‐sectional	16/22	FM: 48.3 ± NR/HC: 45.7 ± NR (all female; SD NR)	7.6 ± 3.8	Analgesics/NSAIDs/sedatives‐hypnotics restricted pre‐scan; anticonvulsants (*n* = 1); antidepressants (*n* = 11)	ACR 1990	3.0 T	Seed‐based + ICA + fALFF	FM: FIQ 61.2 ± 13.3	↓ INS–S1/M1, ↓ THA–PMC; ↑ SMG–CER; mixed pattern.	Stronger insula–PCC, thalamus–mPFC and operculum–parahippocampal connectivity correlated with higher pain sensitivity.	6/9 ★
Harper et al. (2018)	Cross‐sectional	15/14	FM: 40.7 ± 10.2/HC: 40.7 ± 11.5 (all female)	≥ 6 months	CNS‐active antidepressants/stimulants/anorectics/anticonvulsants withdrawn; OTC analgesics withheld ≥ 8 h	1990 ACR criteria	3.0 T	Seed‐based	Pain: VAS 68.3 (13.4) | Mood: HADS‐D 4.9 (3.3), HADS‐A 6.5 (3.5)	↑ PAG–INS/pgACC; descending inhibition loss.	Increased PAG–insula connectivity was associated with higher pain and altered PAG–pons coupling predicted abnormal conditioned pain modulation.	9/9 ★
Lim et al. (2023)	Baseline cross‐sectional	12/15	FM: 49.3 ± 9.0/HC: 43.4 ± 8.7 (all female)	≥ 1	No new medication during study; current opiates excluded	ACR 1990	3.0 T	SDBOLD	Pain: VAS 5.1 ± 2.3	↓ SDBOLD in PFC/aINS; ↑ SDBOLD in pINS; reduced cortical flexibility.	Lower vmPFC BOLD variability correlated with higher pain and reduced inhibitory control, suggesting impaired cortical flexibility.	7/9 ★
Gurevitch et al. (2024)	Baseline cross‐sectional	19/30	NR (majority female)	NR	NR	ACR 2016	3.0 T	ROI‐based	NR	↓ AMYG baseline reactivity; NF target characterization.	Lower baseline amygdala activity predicted greater neurofeedback modulation success and reduced emotional arousal.	5/9 ★
Aster et al. (2022)	Cross‐sectional	43/40	FM: 53.5 ± 6.5/HC: 52.5 ± 6.7 (all female)	PNS 16.8 ± 10.8; noPNS 18.8 ± 12.7	Off pain medication 3 days; anticonvulsants, antihistamines, muscle relaxants and benzodiazepines excluded within 4 weeks	ACR 2016	3.0 T	ROI‐to‐ROI	Pain: GCPS 73.6 ± 10.8 (PNS), 64.0 ± 15.1 (noPNS) | FM: FIQ 51.9 ± 12.0 (PNS), 42.3 ± 13.2 (noPNS) | Mood: ADS 27.8 ± 11.8 (PNS), 21.2 ± 11.4 (noPNS); STAI 47.1 ± 11.6 (PNS), 44.3 ± 13.3 (noPNS)	↑ DMN–INS; ↑ rEAN–INS.	Reduced functional connectivity was associated with higher pain intensity and greater symptom severity.	7/9 ★

*Note:* Cross‐sectional and baseline resting‐state functional MRI (rs‐fMRI) studies comparing fibromyalgia (FM) and healthy controls (HC) summarize alterations in functional connectivity, local activity metrics and network topology. Direction of effects is shown as increases (↑) or decreases (↓) in FM relative to HC. Methodological quality is shown as NOS score★ (0–9), with higher scores indicating better methodological quality. Values are reported as mean ± SD, unless otherwise stated; median [IQR], range, subgroup‐specific values or symptom duration were retained as originally reported. References only in this table: Resting‐state fMRI (Cifre et al. 2012; Kim, Loggia, et al. 2015; Kong et al. 2019; Kong et al. 2021; Lim et al. 2023; Gurevitch et al. 2024; Elkana and Beheshti 2025; van Ettinger‐Veenstra et al. 2020; Park, Baker, et al. 2022).

Abbreviations: ACC/rACC/pgACC/MCC/PCC, cingulate cortex subregions; ALFF, amplitude of low‐frequency fluctuations; AMYG, amygdala; AUC, area under the receiver operating characteristic curve; CAU, caudate; CEN/CCN, central/cognitive executive network; DAN, dorsal attention network; dDMN/vDMN, dorsal/ventral DMN; dFC, dynamic functional connectivity; DMN, default mode network; EAN, executive attention network; fALFF, fractional ALFF; FC, functional connectivity; FPN, frontoparietal network; GC, Granger causality; HIP, hippocampus; ICA, independent component analysis; IENFD, intraepidermal nerve fibre density; INS/aINS/pINS, insula/anterior insula/posterior insula; L/R/bilat, left/right/bilateral; LC, locus coeruleus; ML, machine learning; NAcc, nucleus accumbens; noPNS, no peripheral nerve involvement; NOS, Newcastle–Ottawa Scale; NRM, nucleus raphe magnus; PAG, periaqueductal grey; PMC, premotor cortex; PNS, peripheral nerve involvement; PUT, putamen; ReHo, regional homogeneity; ROI, region of interest; ROI‐to‐ROI, connectivity between ROIs; rs‐fMRI, resting‐state functional MRI; RVM, rostral ventromedial medulla; S1/S2/M1, primary somatosensory cortex/secondary somatosensory cortex/primary motor cortex; SDBOLD, standard deviation of BOLD signal; seed‐based FC, seed‐to‐voxel connectivity; SMN, sensorimotor network; SN, salience network; THA, thalamus; VAN, ventral attention network; VIS, visual network; VTA, ventral tegmental area.

The DMN showed particularly inconsistent findings. Six studies reported increased connectivity or activity within the DMN (Fallon et al. 2016; Ichesco et al. 2014, 2016; Kaplan et al. 2019; Napadow et al. 2010; Pando‐Naude et al. 2019), whereas five reported decreased connectivity or activity in the same network (Ceko et al. 2013; Fallon et al. 2016; Jarrahi et al. 2018; Larkin et al. 2021; Liu et al. 2024). These findings indicate that DMN abnormalities were recurrent, but not directionally consistent across studies.

Connectivity involving the salience network and its principal hub, the insula, also showed mixed results. Some studies described increased connectivity (Jarrahi et al. 2017; Kutch et al. 2017), whereas others reported decreased connectivity (Flodin et al. 2014, 2015; Jung et al. 2020). Thus, although salience‐related alterations were repeatedly implicated, their direction likewise varied across samples and analytic approaches.

**FIGURE 2:**
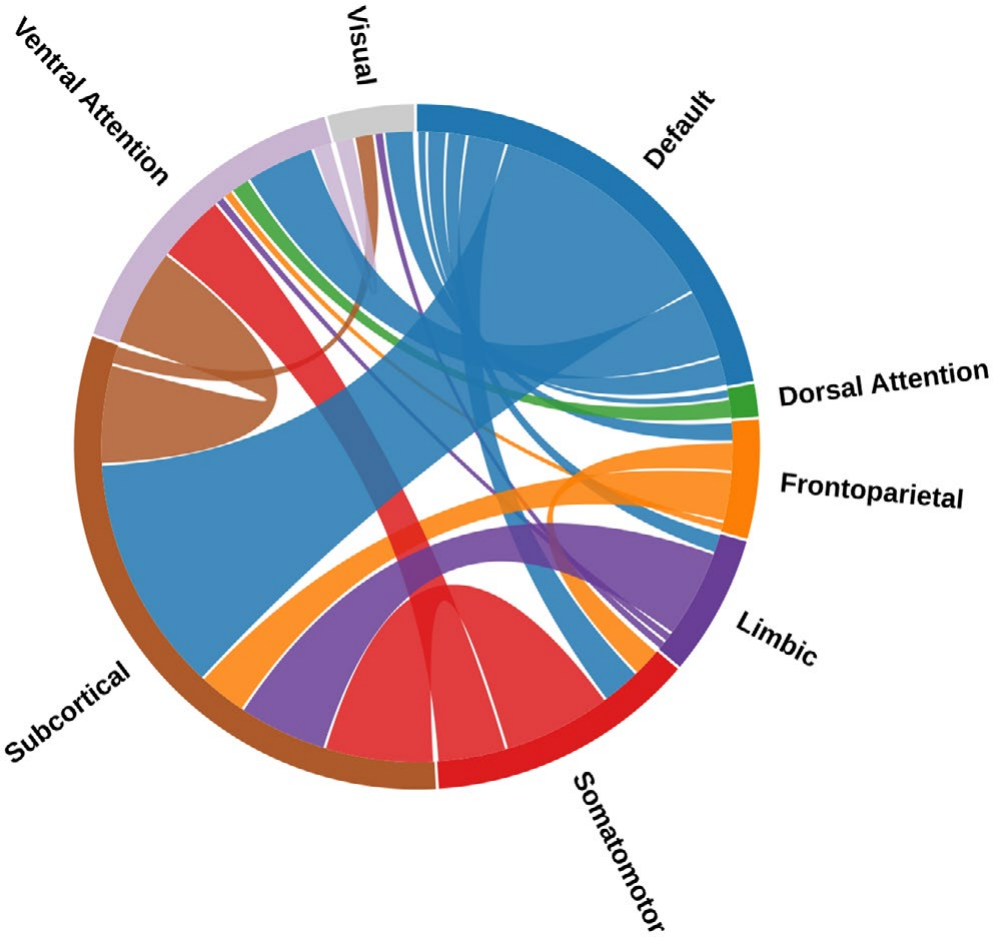
Network‐level alterations in resting‐state functional connectivity in fibromyalgia. Chord diagram summarizing reported altered resting‐state functional connectivity findings in fibromyalgia (FM) versus healthy controls (HC), after mapping ROI‐level findings to harmonized large‐scale Yeo‐7 network categories. Ribbon width reflects the number of reported altered ROI‐to‐ROI findings mapped to each network pair. Within‐network connections were excluded.

Connectivity involving the PAG or PAG‐adjacent brainstem regions was also non‐convergent, with reports of both decreased and increased coupling with regions involved in descending pain modulation, including the insula, rostral/perigenual anterior cingulate cortex, dorsal pons, caudal pons and rostral ventromedial medulla (Coulombe et al. 2017; Flodin et al. 2015; Harper et al. 2018; Truini et al. 2016). More broadly, when ROI‐to‐ROI and seed‐based findings were aggregated in the chord diagram (Figure 2), subcortical and brainstem nodes such as the thalamus, basal ganglia, amygdala and PAG emerged as frequent endpoints, although their coupling patterns remained directionally inconsistent. Overall, the resting‐state literature was characterized by recurrent involvement of the DMN, salience/insula regions and PAG‐centred descending pain‐modulatory circuitry, but without a single reproducible network‐level connectivity profile.

In addition to methodological diversity, resting‐state studies showed substantial heterogeneity in sample characterization. Psychiatric symptoms were handled through variable exclusion criteria, structured assessments or symptom scales and medication handling ranged from washout or exclusion of centrally acting drugs to allowance of stable ongoing treatment or descriptive reporting without formal modelling (Aster et al. 2022; Ceko et al. 2013; Coulombe et al. 2017; van Ettinger‐Veenstra et al. 2020; Harper et al. 2018; Ichesco et al. 2014, 2016; Kim et al. 2021; Larkin et al. 2021). Overall, uneven reporting and control of mood symptoms, comorbidities and medication exposure likely contributed to between‐study heterogeneity. Detailed study‐level information is provided in Table S2.

#### 3.2.4 Functional Brain Alterations: Task‐Based fMRI

**FIGURE 3:**
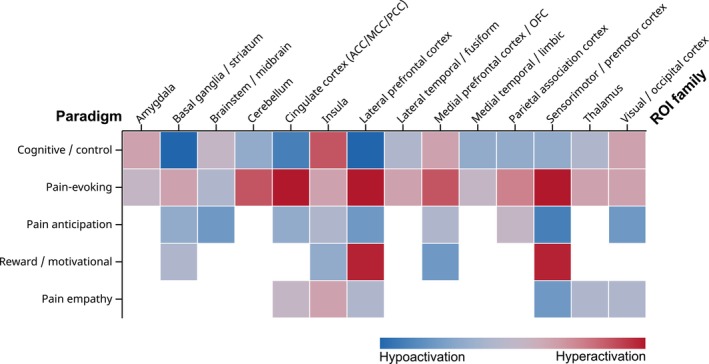
Task‐evoked activation across paradigms and macro‐anatomical brain regions in fibromyalgia. Paradigm‐by‐region family heat map of task‐evoked BOLD activation in fibromyalgia (FM) versus healthy controls (HC). Colours represent a normalized balance of reports of increased (red) versus decreased (blue) activation for each region–paradigm cell. The underlying study counts contributing to each cell are provided in Table S3.

A total of 40 studies used task‐based fMRI to probe evoked brain responses in FM across pain‐related, anticipation/expectancy, reward and cognitive paradigms (Table 5). Among the reviewed modalities, task‐based fMRI provided the clearest mechanistic signal in FM. Across paradigms, the predominant pattern was altered responsivity within insular, somatosensory and cingulate pain‐processing regions, together with reduced or dysregulated recruitment of prefrontal control systems. To summarize the distribution of these findings across experimental paradigms and major region families, we constructed a paradigm‐by‐region family heat map (Figure 3), in which colour encodes a normalized index based on the balance between reports of increased versus decreased task‐evoked activation in each cell. The underlying paradigm‐by‐region family counts used to construct Figure 3 are provided in Table S3.

**TABLE 5:** Task‐based fMRI findings in fibromyalgia.

Author/Year	Design	Sample size (FM/HC)	Mean age ± SD; sex ratio	Pain duration, years	Scanner	Experimental paradigm	Analysis method	Diagnostic criteria	Medication use reported	Key clinical values	Main findings	NOS score
Burgmer et al. (2010)	Cross‐sectional	17/17	FM: 52.6 ± 7.9/HC: 49.5 ± 8.9 (all female)	NR	3.0 T	Tonic pain	GLM + ROI	ACR 1990	Medication that may alter pain perception or brain activation (e.g., pain medication, anxiolytics, antidepressants) discontinued 48 h before scan	Pain: Clinical pain pre (VAS) 31.2 ± 24.4 | FM: PDI score 26.4 ± 13.4; FFbH (% of functioning) 70.2 ± 17.3 | Mood: HADS 21.2 ± 7.9	↓ middle frontal gyrus (temporal deactivation unique to FM); negative pre‐stimulus correlations in MCC, SMA, precentral (FM<HC/RA) during tonic pain	9/9 ★
Glass et al. (2011)	Cross‐sectional	18/14	FM: 43.6 ± 9.8/HC: 41.1 ± 11.9 (all female)	9.1	3.0 T	Cognitive	Seed‐based FC	ACR NR	NR	Pain: Average Pain Intensity (PED) 55.4 (15.5) | Mood: Depressive Symptoms (CES‐D) 16.28 (9.31); Anxiety (STPI) 20.1 (5.9)	↓ right premotor, SMA, MCC, left DLPFC (BA9), right IPL, right insula, left putamen; ↑ right inferior temporal/fusiform + ↑ FC with medial frontal/preSMA (inhibitory control)	8/9 ★
Diers et al. (2011)	Cross‐sectional	6/6	FM: 54.7 ± 4.5/HC: 50.3 ± 8.8 (all female)	21.67 (15.49) (range 6–45)	3.0 T	Chemical pain	GLM + ROI	ACR 1990/2010	No opioid medication; one NSAID; three antidepressants (1 SSRI, 1 tricyclic, 1 tetracyclic); patients asked not to take pain medication the day before and if possible 3 days before measurement; antidepressants not interrupted	Pain: Habitual pain intensity 3.8 (2.8) on 0–10 NRS before measurement | FM: FIQ total 44.43 (5.87) | Mood: CESD 20.5 (11.48)	↑ ACC/MCC/SI + ↑ bilateral insula, thalamus, left BG, OFC, cerebellum (chemically induced pain); ↑ left anterior insula (FM>HC)	6/9 ★
Burgmer et al. (2011)	Cross‐sectional	12/14	FM: 50.1 ± 7.3/HC: 46.9 ± 6.8 (all female)	10.50 (6.26) (range 3–26)	3.0 T	Anticipation	GLM + ROI	ACR 1990	Wide range of medication including steroids, opioids, nonsteroidal antirheumatics, tricyclic antidepressants and SSRIs; medication altering pain perception or brain activation discontinued 48 h before scanning	FM: Pain Disability Index 47.7 (18.1); Hannover Functional Capacity Questionnaire 72.0 (15.0) | Mood: HADS total score 18.1 (7.1)	↑ DLPFC (BA9), PAG, PPC (anticipation of known pain); ↑ recruitment of attentional/descending networks	8/9 ★
Seo et al. (2012)	Cross‐sectional	19/22	FM: 38.7 ± 7.6/HC: 38.3 ± 8.5 (all female)	39.41 ± 43.90 months	3.0 T	Cognitive	GLM + ROI	ACR 1990	Allowed; not controlled; 7 patients took antidepressants; 6 took pregabalin (75 mg) once daily; 1 took pregabalin (75 mg) plus milnacipran (25 mg) once daily; medication considered as possible confound and subgroup analyses found no activation differences at *p* < 0.01 uncorrected	FM: FIQ 59.37 ± 19.89 | Mood: BDI 23.21 ± 10.59; BAI 29.79 ± 8.45	↓ left DLPFC, right VLPFC, right inferior parietal (2‐back); ↓ global BOLD in frontoparietal network	9/9 ★
Jensen, Loitoile, et al. (2012); Jensen, Kosek, et al. (2012)	Cross‐sectional	28/14	FM: 37.8 ± 6.8/HC: 33.6 ± 8.6 (all female)	123.8 (76.8) months	1.5 T	Pressure pain	Seed‐based FC	ACR 1990	CNS‐acting therapies washed out; antidepressants, anticonvulsants, mood stabilizers, opioids, narcotic patches, TENS, biofeedback, tender/trigger point injections, acupuncture and anaesthetics withdrawn; analgesics prohibited except paracetamol, dipyrone and NSAIDs as rescue; analgesic/narcotic drugs stopped 48 h; zolpidem allowed	Pain: Average clinical pain during previous week 72.3 (13.3) VAS mm | FM: FIQ 71.0 (12.6)	↓ FC rACC–amygdala/hippocampus/PAG/RVM and ↓ thalamus–OFC; no increases; ↓ DPMS	8/9 ★
Craggs et al. (2012)	Cross‐sectional	13/11	NR (all female)	NR	3.0 T	Thermal pain	SEM	ACR 1990	NR	NR	Reversal S1 ↔ S2; ↑ posterior insula → aMCC (directionality)	8/9 ★
Burgmer et al. (2012)	Cross‐sectional	17/17	FM: 52.6 ± 8.0/HC: 49.5 ± 8.9 (all female)	≥ 2	3.0 T	Tonic pain	GLM + ROI	ACR 1990	10 patients used no pain medication; remaining patients used steroids, non‐steroid anti‐rheumatics and tricyclic antidepressants; medications that may alter pain perception or brain activation were discontinued 48 h before scanning	Pain: NRS 1 4.71 ± 9.43 (pre‐incision) | FM: PDI 26.35 ± 13.45; FFbH 70.23 ± 17.29 | Mood: HADS 21.24 ± 7.87; Preanxiety 32.47 ± 25.47	HC: ↑ bilateral SMC with hyperalgesia and ↓ left DLPFC; FM: ↑ left DLPFC and ↓ SMC (between‐group reversal)	9/9 ★
Kamping et al. (2013)	Cross‐sectional	16/16	FM: 53.0 ± 7.1/HC: 51.3 ± 8.5 (all female)	23.21 (15.20) (range 5–60)	3.0 T	Laser pain	GLM + ROI	ACR 1990/2010	All FM patients except 2 discontinued usual pain medication and were at least 3 days without medication; 2 continued duloxetine or citalopram	FM: FIQ sum score 43.82 (SD 12.91, range 21.55 to 64.34) | Mood: CESD 16.75 (SD 8.46, range 4.00–30.00)	↓ ventral ACC, right posterior insula, left anterior insula, right SII, left OFC (positive picture + pain)	9/9 ★
Lee et al. (2013)	Cross‐sectional	23/24	FM: 38.0 ± 7.3/HC: 37.3 ± 8.1 (all female)	27.7 ± 36.7 months (median 12.0 months)	3.0 T	Empathy	GLM + ROI	ACR 1990	Patients were on stable doses of medication and instructed not to take any medication on the test day; controls took no psychoactive medication	FM: KFIQ 56.4 ± 19.5 | Mood: BDI 21.6 ± 9.9; BAI 29.4 ± 8.1	↓ ACC, DLPFC, thalamus, insula, pre/postcentral, IPL, SMA (pain‐image observation) with restricted activation in FM	8/9 ★
Kim et al. (2013)	Baseline cross‐sectional	21/11	FM: 51.3 ± 8.4/HC: 46.5 ± 12.0 (all female)	NR	3.0 T	Pressure pain	GLM	ACR 1990	Pregabalin pharmacological therapy; other medication restrictions NR	FM: FIQ 65.98 ± 18.11 | Mood: BDI 41.10 ± 12.17; STAI1 44.10 ± 8.46; STAI2 47.05 ± 7.58	Baseline FM: ↑ SII, insula, IFG, thalamus, MTG, IPL, cerebellum; post‐pregabalin: ↓ thalamus, SI, IPL, calcarine, MFG, MCC, precuneus, insula	5/9 ★
Schmidt‐Wilcke et al. (2014)	Baseline cross‐sectional	17/12	FM: 44.1 ± 9.9/HC: 42.7 ± 12.2 (all female)	NR	3.0 T	Cognitive	GLM	ACR 1990	NR	Pain: PED 54.4 (16.4) (0–100) | Mood: CES‐D 15.9 (9.4); STPI 18.6 (5.8)	↓ dACC/MCC, ↓ SMA, ↓ right premotor cortex during inhibition (FM<HC)	6/9 ★
Loggia et al. (2014)	Cross‐sectional	31/14	FM: 44.0 ± 11.9/HC: 44.2 ± 14.3 (FM 27F/4M, HC 10F/4M)	12.5 ± 12.2	3.0 T	Anticipation	GLM + ROI	ACR 2010	opioids excluded	Pain: Clinical pain intensity (0–100) 34.3 ± 25.19 | FM: BPI, Pain Interference (0–10) 5.5 ± 2.0 | Mood: BDI (0–63) 17.0 ± 13.6	HC: ↑ VTA/ACC/MCC/PAG/SMA/S2/insula (pain anticipation/stimulation) + VTA ↓ during relief anticipation; FM: reduced/absent responses	6/9 ★
Martinsen et al. (2014)	Cross‐sectional	23/28	FM: 49.8 (25–64)/HC: 46.3 (20–63) (all female; age range only)	8.9 (0.5–19)	3.0 T	Cognitive	GLM	ACR 1990	One patient on anticonvulsants; 11 on antidepressants (4 TCA, 4 SSRI, 3 SNRI); daily NSAIDs 2, acetaminophen 4, tramadol 1; refrained from hypnotics, NSAIDs, acetaminophen and tramadol/other analgesics 48 h before study participation and 72 h before fMRI	Pain: Pain VAS (mm) 45.3 (5–92) | FM: FIQ 63.1 (42.5–85.0) | Mood: HADS‐D 7.3 (3.0–16.3); HADS‐A 8.8 (0–18)	↓ caudate, hippocampus, lingual gyrus, MTG (FM<HC) with no ACC/PFC differences	8/9 ★
Rahm et al. (2015)	Cross‐sectional	11/11	FM: 48.5 ± 6.5/HC: 46.4 ± 8.4 (all female)	6.7 (6.9)	3.0 T	Empathy	ROI	ACR 1990	Concurrent analgesic or psychotropic medication allowed; participants asked not to take medication in the morning of scanning; tramadol (3), hydromorphone (1), antidepressants (6: mirtazapine, sertraline, doxepin, amitriptyline, clomipramine, duloxetine)	Mood: BDI 20.0 (6.8)	↑ anterior insula, ACC, SMA/pre‐SMA, S1/S2 (pain‐related image observation; self‐perspective)	7/9 ★
Harte et al. (2016)	Baseline cross‐sectional	28/19	FM: 39.7 ± 11.2/HC: 41.1 ± 12.2 (all female)	> 6 months (disease duration criterion)	3.0 T	Cognitive	GLM + ROI	ACR 1990	Current use/history of opioid or narcotic analgesics, sedatives, hypnotics, unstable doses of antidepressants, NSAIDs or muscle relaxants excluded; pregabalin nonresponders to ≥ 300 mg/d excluded for pharmacologic subset	Pain: 48.2 ± 23.3 (VAS at baseline)	↑ right anterior insula (aversive checkerboard)	9/9 ★
Ellerbrock et al. (2021)	Cross‐sectional	70/35	FM: 47.4 ± 7.9/HC: 47.9 ± 7.9 (all female)	AA: 184 (± 112, 24, 492) mo; */G: 189 (± 89.2, 60, 408) mo	3.0 T	Pressure pain	GLM + PPI	ACR 1990/2011	Antidepressants/anticonvulsants excluded; analgesics/NSAIDs/hypnotics restricted before visits; no strong opioids	FM: FIQ – AA: 63.6 (± 16.2); */G: 61.5 (± 18.3) | Mood: BDI—AA: 16 (± 8); */G: 15.1 (± 7.8); STAI‐S—AA: 43.3 (± 11.8); */G: 44.5 (± 12.3)	OPRM1 G‐allele: ↑ PCC/precentral during painful pressure; ↓ FC PCC cluster–DLPFC/precentral/angular	9/9 ★
Schreiber et al. (2017)	Cross‐sectional	38/15	FM: 46.3 ± 11.4/HC: 44.1 ± 14.8 (FM 33F/5M; HC 11F/4M)	NR	3.0 T	Tonic pain	GLM	ACR 2010	Opioids excluded; gabapentin, antidepressants, nonsteroidal anti‐inflammatory drugs and acetaminophen allowed/continued	Pain: 5.4 ± 2.1 (Pain severity (BPI; NRS 0–10)) | FM: Widespread pain index 10.9 ± 2.6; Symptom severity sum 9.3 ± 1.9 | Mood: Depression (BDI) 15.2 ± 8.2	↓ deactivation of medial temporal lobe (amygdala/hippocampus/PHG/entorhinal) during pain and after‐sensations	8/9 ★
Derbyshire et al. (2017)	Cross‐sectional	13/15	FM: 51.4 (all female)/HC: 25.3 (7 M/8F)	NR	3.0 T	Cognitive	GLM + ROI	ACR 1990	NR	Pain: 4.1 (SE 0.6) prescan pain | Mood: HADS‐A 9.5 (1.1); HADS‐D 7.7 (1.3)	↑ PAG, thalamus, aMCC, anterior insula, S1/S2, BA9/46 with ↑ suggested pain (hypnosis)	5/9 ★
Ellingson et al. (2018)	Cross‐sectional	20/18	FM: 42.3 ± 11.3/HC: 40.7 ± 9.3 (all female)	NR	3.0 T	Cognitive	ROI	ACR 2016	Regular opioids, cardiovascular medications, anticonvulsants and high‐dose antidepressants excluded; low‐dose antidepressants permitted (4 FM patients) and maintained; abstain from pain medications for 24 h before testing	Pain: SF‐MPQ VAS: 38.3 (17.0) | FM: FIQ: 51.2 (15.2) | Mood: BDI: 9.2 (8.1); STAI Trait Anxiety: 38.2 (10.6); POMS Total Mood Disturbance: 133.7 (30.4)	Distraction reduces pain in both groups; FM: ↑ DLPFC associated with PCS (reduced top‐down modulation)	9/9 ★
Martinsen et al. (2018)	Baseline cross‐sectional	19/20	FM: 49 ± 6/HC: 47 ± 2 (all female)	8 (range 1–16)	3.0 T	Cognitive	GLM	ACR 1990	Analgesics/NSAIDs/hypnotics restricted; patients refrained from hypnotics, NSAIDs, acetaminophen and tramadol/other analgesics at least 48 h before study participation and 72 h before fMRI	Pain: 44.6 ± 17.5 (Pain VAS) | FM: 59.9 ± 16.0 (FIQ total) | Mood: HAD‐D 11.3 ± 11.1; HAD‐A 8.63 ± 4.4	↓ caudate and ↓ temporal areas in Stroop incongruent>congruent (FM<HC)	7/9 ★
Martucci et al. (2019)	Cross‐sectional	34/15 (FM subgrouped: 17 opioid users/17 non‐opioid)	FM: 48.1 ± 9.6 (no opioids)/FM: 52.8 ± 6.9 (opioids)/HC: 48.1 ± 10.2 (all female)	Non‐opioid FM: 11.5 ± 7.7; opioid FM: 10.0 ± 7.0	3.0 T	Reward	ROI	ACR 2011	Allowed: normal medication use continued; opioid group required opioid treatment ≥ 3 months; non‐opioid group no opioids in prior 90 days and never > 30 days; classes reported included opioids, NSAIDs, acetaminophen, SNRIs, SSRIs, tricyclic antidepressants, other anxiolytics, antiepileptics, triptans, benzodiazepines, benzodiazepine‐like drugs, muscle relaxants, GABA analogues, low‐dose naltrexone, medical cannabis, SARI, NDRI, ondansetron	Pain: Pain Severity (BPI): non‐opioid FM 5.7 ± 2.1; opioid FM 6.0 ± 1.5 | FM: Fibromyalgia Assessment Form/Number of Pain Areas (FAF): non‐opioid FM 13.9 ± 3.9; opioid FM 12.6 ± 3.7 | Mood: Trait Anxiety (STAI): 49.7 ± 8.5/51.9 ± 12.4; State Anxiety (STAI): 41.4 ± 7.0/41.9 ± 12.8; Depression (BDI): 15.8 ± 8.9/15.2 ± 9.3	FM without opioids: ↓ MPFC (reward anticipation) + ↑ MPFC (non‐loss); opioid users = normalized	8/9 ★
Hubbard et al. (2020)	Cross‐sectional	28/28	FM 45.3 ± 8.7/HC 44.6 ± 9.1 (gender NR)	NR	3.0 T	Tonic pain	GLM	ACR 2010	Patients continued usual medications, including antidepressants, gabapentin, NSAIDs and acetaminophen	Pain: NRS ratings: 44.84 ± 7.70	FM: ↑ DLPFC (pain offset) ∝ PCS; ↑ VLPFC (pain onset) ∝ unpleasantness; ↑ OFC → lower pressure thresholds	8/9 ★
Sandström et al. (2020)	Cross‐sectional	67/34	FM: 47 ± 8/HC: 48 ± 8 (all female)	119 (87, 11–408) months	3.0 T	Anticipation	GLM + PPI	ACR 1990/2011	Antidepressants or anticonvulsants excluded; had to refrain from NSAIDs, analgesics or hypnotics before participation; HC without regular NSAIDs, analgesics or sleep medication	Pain: VAS pain current: 52 (22, 6–99) | FM: FIQ: 63 (17, 13–95) | Mood: BDI: 16 (8, 1–36); STAI‐Trait: 43 (12, 24–75); STAI‐State: 48 (8, 34–70)	P30green > P30red: ↑ M1, anterior insula, IPL, cerebellum, lingual; HC shows opposite pattern in S2/mid‐insula	9/9 ★
López‐Solà et al. (2017)	Cross‐sectional	37/35	FM 48.0 ± 8.9/HC 47.6 ± 7.4 (all female)	80.41 ± 52.05 months	3.0 T	Pressure pain	GLM + ROI	ACR 1990	Stable medical treatment allowed; rescue analgesic drugs withheld 72 h before scanning; antidepressants, anxiolytics, hypnotics, gabapentin, ibuprofen, paracetamol, tramadol reported	Pain: Clinical Pain (0–100 NRS) 72.03 ± 14.82 | FM: FIQ (Total Score) 66.86 ± 15.79 | Mood: HADS‐Depression 8.89 ± 4.72; HADS‐Anxiety 11.54 ± 4.15	FM: ↑ NPS activation and ↑ FM‐pain/multisensory pattern expression in the insula/operculum (↑), mPFC (↑), PCC (↑) and ACC (↑) compared with controls	9/9 ★
Kim et al. (2020)	Cross‐sectional	13/18	FM: 48.1 ± 9.6/HC: 47.8 ± 8.9 (all female)	NR	3.0 T	Reward	GLM + ROI	ACR 2011	Restricted: routine moderate‐to‐high opioid use (> 60 mg morphine equivalents); benzodiazepines excluded except alprazolam, lorazepam and diazepam	NR	↓ striatum (caudate/NAcc/putamen) during reward/loss anticipation (FM<HC)	8/9 ★
Park, Baker, et al. (2022); Park et al. (2022a, 2022b)	Cross‐sectional	20/20	FM: 35.9 ± 12.3/HC: 44.3 ± 12.1 (all female)	6.03 (5.33) (range 9 months–20 years)	3.0 T	Reward	ROI	Modified ACR 2016	Patients were opioid‐naïve/no opioids before study > 90 days; non‐opioid and mood‐altering medications allowed and listed (NSAIDs, acetaminophen, SNRIs, SSRIs, TCAs, anxiolytics, triptans, trazodone, antiepileptic, muscle relaxants, GABA analogues, benzodiazepine, NDRI); controls took no pain or mood‐altering medications	Pain: 4.5 ± 1.9 (Pain Severity [BPI]) | FM: 12.05 ± 3.8 (Number of Pain Areas [FAF]) | Mood: BDI 16.7 ± 8.7; STAI‐Trait 48.2 ± 10.4; STAI‐State 38.7 ± 9.3	↓ MPFC (reward anticipation) + ↑ MPFC (no‐loss outcome); ↓ anterior insula (exploratory)	9/9 ★
Balducci et al. (2022)	Cross‐sectional	33/33	FM: 41.7 ± 6.1/HC: 41.5 ± 6.0 (all female)	NR	3.0 T	Cognitive	ICA	ACR 1990/2016	Opioids excluded; participants needed to stop rescue‐dose analgesic or benzodiazepine for at least 24 h before MRI; current medication assessed but not reported in detail	NR	Both activate PFC/ACC/insula/amygdala (emotion regulation); FM shows altered descending connectivity	7/9 ★
Ioachim et al. (2022)	Cross‐sectional	15/15	FM: 46.0 ± 13.0/HC: 39.0 ± 10.0 (all female)	NR	3.0 T	Anticipation	SEM	ACR 1990/2016	Not taking centrally‐acting medications; other medications allowed if taken for at least 3 months prior; participants were not asked to stop ongoing non‐centrally‐acting medication	Pain: Initial pain score 33.9 (23.7) (controls 2.3 (5.62)) | FM: FIQR total 50.26 (3.66) | Mood: BDI 16.26 (2.77)	↑ PAG–NRM and ↓ LC–C6; anticipation: ↑ LC/PBN + ↓ NRM–C6	7/9 ★
Oliva et al. (2022)	Cross‐sectional	20/20	FM: 43 ± NR/HC: 35 ± NR (18F each group)	NR	3.0 T	Anticipation	GLM + ROI	ACR 2010	Regular medications not altered; included non‐opioid analgesics (*n* = 13), opioids (*n* = 9), tricyclic antidepressants/serotonin and noradrenaline reuptake inhibitors (*n* = 11), and gabapentinoids (*n* = 7); medications taken in the previous 72 h were recorded	Pain: BPI pain on average 6.4 ± 1.7; BPI pain now 5.3 ± 1.6 | FM: Widespread Pain Index 13.5 ± 2.6; Symptom Severity 10 ± 1.5 | Mood: Hospital Anxiety (HADS) 12.2 ± 3.6; Hospital Depression (HADS) 10.5 ± 4.7	Attentional analgesia present; HC>FM in frontopolar (BA10) and left LC	8/9 ★
Cheng et al. (2022)	Cross‐sectional	84/38	FM: 39.8 ± 12.3/HC: 38.8 ± 12.9 (all female)	NR	3.0 T	Tonic pain	Dynamic FC	ACR NR	NR	NR	↑ S1‐leg–salience (insula/MCC/TPJ/IFG) and ↑ dynamic S1‐leg–S2/TPJ (high TSP)	8/9 ★
Park, Baker, et al. (2022); Park et al. (2022a, 2022b)	Cross‐sectional	20/20	FM: 35.9 ± 12.3/HC: 44.2 ± 12.1 (all female)	6.03 (5.33) (range 9 months–20 years)	3.0 T	Reward	GLM + ROI	Modified ACR 2016	Patients were opioid‐naïve/no opioids before study > 90 days; non‐opioid and mood‐altering medications allowed and listed (NSAIDs, acetaminophen, SNRIs, SSRIs, TCAs, anxiolytics, triptans, trazodone, antiepileptic, muscle relaxants, GABA analogues, benzodiazepine, NDRI); controls took no pain or mood‐altering medications	Pain: 4.5 ± 1.9 (Pain Severity [BPI]) | FM: 12.05 ± 3.8 (Number of Pain Areas [FAF]) | Mood: BDI 16.7 ± 8.7; STAI‐Trait 48.2 ± 10.4; STAI‐State 38.7 ± 9.3	↓ MPFC (reward anticipation) + ↑ MPFC (no‐loss); ↑ within‐DMN FC	8/9 ★
Sandström et al. 2024	Cross‐sectional	65/33	FM: 48 ± 8/HC: 48 ± 8 (all female)	119 (90, 11, 408) months	3.0 T	Anticipation	GLM + PPI	ACR 1990/2011	Ongoing medication with antidepressants or anticonvulsants excluded; inability to refrain from NSAIDs, analgesics or hypnotics for at least 48 h before participation (72 h before fMRI) excluded; healthy controls had no regular NSAIDs, analgesics, sleep medication, antidepressants or anticonvulsants.	Pain: VAS pain current: 52 (23, 6, 99) | FM: FIQ: 62 (16, 12, 92) | Mood: BDI: 15 (8, 1, 34); STAI‐State: 42 (12, 24, 75)	↓ left/right dlPFC (pain anticipation); HC shows dlPFC–SMA/S1 coupling absent in FM	7/9 ★
Mosch, Hagena, Herpertz, and Diers (2023); Mosch, Hagena, Herpertz, Ruttorf, and Diers (2023)	Cross‐sectional	22/21	FM: 50.5 ± 9.9/HC: 46.6 ± 13.1 (all female)	14.88 (11.82) (range 2–44)	3.0 T	Thermal pain	Seed‐based FC	ACR 2016	No pain medication on scan day; opioid use suspended no later than 3 days before MRI (1× fentanyl patches, 1× tramadol); psychotropic medication users excluded	NR	↓ VLPFC/DLPFC/dACC (self‐controlled pain) + absent OFC; FM: ↑ amygdala/hypothalamus/PHG; ↓ FC PFC–pain regions	7/9 ★
Löfgren et al. (2023)	Baseline cross‐sectional	18/19	FM: 50 (44–57)/HC: 55 (40–58) (all female; median [range])	9 (median; EIH cohort); fMRI subsample 8 (median)	3.0 T	Intervention	GLM + PPI	ACR 1990	Needed to refrain from analgesics/NSAIDs/hypnotics; declared refraining from hypnotics, NSAIDs, acetaminophen and tramadol/other analgesics 48 h before EIH and 72 h before fMRI	Pain: VAS pain 54.0 (33.7–70.2) (EIH cohort); fMRI subsample 38.0 (28.5–54.8) | FM: FIQ total 59.8 (50.1–73.6) (EIH cohort); fMRI subsample 63.9 (52.8–73.8) | Mood: HADS‐D 7.0 (5.0–8.2); HADS‐A 8.0 (4.0–11.0) (EIH cohort); fMRI subsample HADS‐D 6.5 (5.0–10.2), HADS‐A 9.0 (6.2–10.0)	↑ insula, ACC, caudate; ↓ dlPFC/OFC in baseline pain	6/9 ★
Balducci et al. (2024)	Cross‐sectional	30/31	FM: 41.9 ± 6.3/HC: 41.2 ± 6.1 (all female)	NR	3.0 T	Cognitive	GLM + PPI	ACR 2016	56.67% used medication daily; pregabalin was the most prescribed	Pain: VAS for pain during the interview: 45.7 (19.8) (HC: 1.2 [3.5]) | FM: Widespread pain index: 11.8 (4.3); Symptom severity scale: 8.3 (2.5) | Mood: HAMD total score: 15.2 (6.5); HAMA total score: 21.2 (7.0)	↑ left superior lateral occipital (emotional processing); gPPI: FM ↑ pACC–sensorimotor (positive) and ↓ (negative)	8/9 ★
Fanton et al. (2022)	Cross‐sectional	83/43	FM: 47.3 ± 7.8/HC: 48.1 ± 7.6 (all female)	FM duration 121.3 (87.6, 11, 408)	3.0 T	Anticipation	GLM + ROI	ACR 1990/2011	Anticonvulsants or antidepressants excluded; inability to refrain from hypnotics, NSAIDs or analgesics before participation excluded; 48 h before first visit and 72 h before neuroimaging	Pain: VAS current 53.5 (22.1, 6, 99) | FM: FIQ 63.5 (16.4, 13, 95) | Mood: HAD‐A 7.8 (4.3, 0, 21); HAD‐D 7.4 (4.1, 0, 18)	↓ rACC activation and ↓ expectation‐induced analgesia; TSPO effect	8/9 ★
Renz et al. (2025)	Cross‐sectional	48/35	FM: 44.6 ± 8.9/HC: 42.3 ± 8.2 (all female)	NR	3.0 T	Intervention	ROI + GLM	ACR NR	NR	Pain: 61.67 ± 16.23 (Pain intensity [CPG]) | Mood: BDI‐II 21.64 ± 8.89; HADS‐A 9.52 ± 3.47	↓ amygdala down‐regulation and ↓ amygdala–vmPFC coupling (emotion regulation)	6/9 ★
Park et al. (2024)	Cross‐sectional	24/24	FM: 37.9 ± 13.9/HC: 41.8 ± 12.4 (all female)	8.58 (8.50) (range 9 months–20 years)	3.0 T	Reward	ICA	ACR 2016	Regular medications allowed/continued; no opioid use at time of study, none in prior 90 days, and no prior use > 1 month lifetime; medication classes included NSAIDs, acetaminophen, other pain medicine, SNRIs, SSRIs, other anxiolytics, triptans, trazodone, antiepileptic, muscle relaxants, GABA analogues, benzodiazepines and NDRI	Pain: Pain Severity (BPI): 5.3 ± 1.3 | Mood: Trait Anxiety (STAI): 45.9 ± 10.5; State Anxiety (STAI): 42.0 ± 9.4; Depression (BDI): 19.0 ± 8.9	↑ left motor network engagement (gain anticipation) (FM>HC); engagement positively related to anxiety and negatively to reward responsiveness	6/9 ★
Walitt et al. (2016)	Cross‐sectional	16/13	FM: 44.9 ± 10.2/HC: 44.2 ± 11.2 (all female)	NR	3.0 T	Reward	GLM	ACR 1990	FM medication use NR	Pain: BPI severity 4.59 ± 2.26 | FM: FIQ impact 53.5 ± 12.8	No group differences; HC: ↑ performance–aINS/putamen/DLPFC coupling; FM: decoupling; subjective dyscognition → ↓ activation (HC: aINS/putamen; FM: S1/SMG/INS).	6/9 ★

*Note:* Task‐based functional MRI (tb‐fMRI) studies comparing fibromyalgia (FM) and healthy controls (HC), grouped by paradigm (pain‐evoked, anticipation/expectancy, reward and cognitive tasks) and summarizing blood‐oxygen‐level‐dependent (BOLD) activation and/or task‐modulated connectivity differences. Direction of effects is shown as increases (↑) or decreases (↓) in FM relative to HC. Methodological quality is shown as NOS score★ (0–9), with higher scores indicating better methodological quality. Values are reported as mean ± SD, unless otherwise stated; median [IQR], range, subgroup‐specific values or symptom duration were retained as originally reported. References only in this table: Task‐based fMRI (Schmidt‐Wilcke et al. 2014; Martinsen et al. 2014; Cheng et al. 2022; Löfgren et al. 2023; Renz et al. 2025).

Abbreviations: ACC/rACC/pgACC/dACC/MCC/aMCC, anterior cingulate cortex and subregions (rostral/pregenual/dorsal/mid/anterior mid‐cingulate); ACR, American College of Rheumatology; AMYG, amygdala; BA, Brodmann area; BDI, Beck Depression Inventory; BG, basal ganglia; BOLD, blood‐oxygen‐level‐dependent signal; BPI, Brief Pain Inventory; CAU, caudate; DLPFC/dlPFC, dorsolateral prefrontal cortex; FIQ/FIQR, Fibromyalgia Impact Questionnaire/Fibromyalgia Impact Questionnaire Revised; FM, fibromyalgia; FUS, fusiform gyrus; GLM, general linear model; HADS, Hospital Anxiety and Depression Scale; HADS‐A/HADS‐D, Hospital Anxiety and Depression Scale anxiety/depression subscales; HC, healthy controls; HIP, hippocampus; ICA, independent component analysis; INS/aINS/pINS, insula/anterior insula/posterior insula; IPL, inferior parietal lobule; L/R/bilat, left/right/bilateral; MPFC/mPFC, medial prefrontal cortex; NAcc, nucleus accumbens; NOS, Newcastle–Ottawa Scale; NR, not reported; NRS, numeric rating scale; OFC, orbitofrontal cortex; OPRM1, μ‐opioid receptor gene; PAG, periaqueductal grey; PCC, posterior cingulate cortex; PCS, Pain Catastrophizing Scale; PFC, prefrontal cortex; PHG, parahippocampal gyrus; PPI, psychophysiological interaction; PUT, putamen; ROI, region of interest; RVM, rostral ventromedial medulla; S1/S2/SI/SII/M1, primary somatosensory cortex/secondary somatosensory cortex/primary motor cortex; SD, standard deviation; SEM, structural equation modelling; SMA/pre‐SMA, supplementary motor area/pre‐supplementary motor area; STAI/STAI‐S/STAI‐T, State–Trait Anxiety Inventory/State/Trait subscales; STG/MTG/ITG, superior/middle/inferior temporal gyri; tb‐fMRI, task‐based functional MRI; THA, thalamus; VAS, visual analogue scale; VLPFC/vlPFC, ventrolateral prefrontal cortex; VTA, ventral tegmental area.

Because task‐based fMRI depends on experimentally evoked responses, differences in sample characterization may be especially consequential in this literature. Psychiatric burden was handled inconsistently across studies: several excluded current or severe psychiatric disorders, including depression, anxiety or psychiatric treatment (Burgmer et al. 2012; Ellingson et al. 2018; Fanton et al. 2022; Ioachim et al. 2022), whereas others documented substantial mood‐related burden or psychiatric comorbidity within the FM sample, including current psychiatric disorder, major depressive disorder, previous depressive episodes, generalized anxiety disorder and PTSD (Balducci et al. 2024; Mosch, Hagena, Herpertz, Ruttorf, and Diers 2023; Rahm et al. 2015). Medication exposure was likewise heterogeneous, ranging from washout or temporary restriction of antidepressants, anticonvulsants, hypnotics, non‐steroidal anti‐inflammatory drugs, analgesics or opioids (Fanton et al. 2022; Harte et al. 2016; Jensen, Loitoile, et al. 2012) to the inclusion of participants under stable ongoing treatment, including non‐opioid analgesics, antidepressants, gabapentinoids, and, in some samples, chronic opioid therapy (Martucci et al. 2019; Oliva et al. 2022; Schreiber et al. 2017). Several studies also incorporated pain‐sensitivity and modulatory measures that are particularly relevant to evoked paradigms, such as pressure pain thresholds, pain tolerance, temporal summation of pain or conditioned pain modulation (Burgmer et al. 2012; Cheng et al. 2022; Fanton et al. 2022; Harte et al. 2016; Oliva et al. 2022), whereas others did not report these variables in comparable detail. Overall, between‐study variability in task‐based findings likely reflects not only differences in paradigm design but also inconsistent characterization of clinically relevant modifiers of evoked neural responses. Detailed study‐level information on comorbidities, medication use and symptom‐related variables for task‐based studies is provided in Data S3.

Task‐based fMRI findings were interpreted within two broad mechanistic domains: paradigms primarily supporting amplified nociceptive responsivity and paradigms probing impaired top‐down or descending pain modulation.

##### 3.2.4.1 Amplified Nociceptive Responsivity/Sensitization‐Related Paradigms

###### 3.2.4.1.1 Pain‐Evoked Paradigms

Pain‐evoked tasks included tonic, pressure, thermal, laser, chemical pain and pain‐observation/empathy paradigms. During tonic pain, two studies reported reduced activation in medial frontal and motor control regions in FM compared with controls, particularly within the medial frontal gyri, mid‐cingulate cortex (MCC), supplementary motor area (SMA) and precentral cortex (Burgmer et al. 2010, 2012). In contrast, other tonic/offset designs showed enhanced recruitment of lateral/ventral prefrontal areas during specific pain phases, with FM demonstrating greater dorsolateral, ventrolateral and orbitofrontal prefrontal cortices (DLPFC/VLPFC/OFC) responses during onset/offset or low‐intensity stimulation (Hubbard et al. 2020; Schreiber et al. 2017).

Pressure pain studies most consistently showed hyperactivation of classic pain‐processing regions in FM. Increased responses in insula, SII, thalamus, inferior frontal gyrus (IFG), inferior parietal lobule (IPL), cerebellum and dorsal anterior cingulate cortex (dACC) were reported at baseline or in multivariate pain‐signature analyses (Kim et al. 2013; López‐Solà et al. 2017). Connectivity‐focused pressure paradigms also indicated reduced coupling within descending pain modulatory system nodes (e.g., rACC–amygdala/hippocampus/PAG/RVM and thalamus–OFC) in FM (Jensen, Loitoile, et al. 2012). Genetic subgroup analyses suggested pain‐evoked PCC/precentral responses and altered PCC–DLPFC/precentral/angular connectivity in FM carriers of specific μ‐opioid receptor gene (OPRM1) variants (Ellerbrock et al. 2021).

Thermal pain and picture‐pain interaction tasks tended to show diminished prefrontal and salience‐network engagement in FM relative to controls. FM demonstrated reduced DLPFC/anterior insula/PAG activation or reduced PFC‐to‐pain‐region coupling during thermal pain and cognitive‐affective pain modulation (Craggs et al. 2012; Mosch, Hagena, Herpertz, Ruttorf, and Diers 2023). Laser pain with affective context revealed lower activity in ventral ACC, insula, SII and OFC in FM (Kamping et al. 2013). Chemically induced pain produced a predominantly hyperresponsive pattern in FM, with increased activation in insula, thalamus, basal ganglia, OFC and cerebellum, including stronger anterior insula activity versus controls (Diers et al. 2011).

Empathy/pain‐observation paradigms showed mixed effects. One study found reduced activation across ACC, DLPFC, thalamus, insula, sensorimotor cortex, IPL and SMA when observing pain images (Lee et al. 2013), whereas another reported increased anterior insula, ACC, SMA/pre‐SMA and primary and secondary somatosensory cortices (S1/S2) responses during self‐perspective pain‐image viewing in FM (Rahm et al. 2015).

##### 3.2.4.2 Impaired Top‐Down/Descending Modulation

In addition to pain‐evoked paradigms, several studies examined anticipatory, cognitive, affective and reward‐related responses, reporting differences in prefrontal, cingulate, brainstem and striatal regions in FM.

###### 3.2.4.2.1 Anticipation Paradigms

Anticipation/expectancy tasks consistently implicated altered prefrontal–brainstem and attentional systems. FM showed increased activation of DLPFC (Brodmann area 9), PAG and posterior parietal cortex during anticipation of known pain in some paradigms (Burgmer et al. 2011). However, other studies observed blunted or absent anticipatory responses within reward/relief‐related and pain‐control regions (e.g., ventral tegmental area (VTA)/ACC/MCC/PAG/SMA/insula) compared with controls (Loggia et al. 2014).

Expectation‐manipulation designs indicated that cue‐driven modulation recruited sensorimotor and salience regions in FM (e.g., primary motor cortex, anterior insula, IPL, cerebellum, lingual gyrus), with patterns differing from controls (Sandström et al. 2020). Brainstem‐focused anticipation work showed altered locus coeruleus (LC)/nucleus raphe magnus (NRM) connectivity during expectancy and attentional analgesia contexts (Ioachim et al. 2022; Oliva et al. 2022). More recent anticipation studies reported reduced rACC and DLPFC engagement in FM during expectation‐induced analgesia or pain prediction (Fanton et al. 2022; Sandström et al. 2023).

###### 3.2.4.2.2 Reward Paradigms

Reward tasks (mostly monetary incentive‐type paradigms) suggested altered mesolimbic and medial prefrontal processing. Multiple studies found reduced medial prefrontal cortex (MPFC) activity during reward anticipation in FM, paired with increased MPFC responses during no‐loss paradigm (Martucci et al. 2019; Park et al. 2022a, 2022b). Striatal hyporesponsivity was also reported, with decreased activation in caudate/nucleus accumbens/putamen during reward/loss anticipation (Kim et al. 2020). One reward study using independent component analysis (ICA) reported increased activation within a left motor network during gain anticipation in FM (Park et al. 2024).

###### 3.2.4.2.3 Cognitive Paradigms

Cognitive tasks covered inhibitory control, working memory, attentional distraction, Stroop interference, emotional regulation/processing and cognitive modulation of pain. In executive/inhibitory paradigms, FM showed reduced activation in premotor/SMA/MCC, DLPFC (BA9), IPL, insula and putamen, alongside increased inferior temporal/fusiform activation and strengthened coupling with medial frontal/pre‐SMA regions (Glass et al. 2011). Working‐memory designs (2‐back) reported globally reduced frontoparietal BOLD responses, particularly in left DLPFC and right VLPFC/inferior parietal cortex (Seo et al. 2012). Cognitive conflict tasks similarly showed decreased caudate and temporal cortex activity during Stroop incongruent processing (Martinsen et al. 2018).

Attentional distraction paradigms reduced pain in both groups but linked FM pain modulation to greater DLPFC engagement, particularly in individuals with higher catastrophizing (Ellingson et al. 2018). Aversive or emotionally salient visual tasks showed increased anterior insula activation in FM (Harte et al. 2016). Hypnosis/suggestion paradigms produced enhanced activation of PAG, thalamus, anterior mid‐cingulate cortex (aMCC), anterior insula, S1/S2 and lateral PFC regions in FM during suggested pain (Derbyshire et al. 2017). Emotion regulation/processing studies reported broadly similar core activation patterns across groups, but with altered prefrontal–cingulate connectivity or increased lateral occipital engagement in FM (Balducci et al. 2022, 2024). Across cognitive paradigms more broadly, behavioural findings were mixed: some studies showed preserved task performance despite altered neural recruitment, whereas others reported slower responses and/or lower accuracy in FM (Ellingson et al. 2018; Glass et al. 2011; Seo et al. 2012).

## 4 Discussion

This systematic review provides a comprehensive synthesis of neuroimaging findings in FM, aiming to identify robust cross‐modal alterations and to contextualize divergent results within methodological and clinical constraints. Across modalities, the literature supports a distributed pattern of brain alterations in FM rather than a single reproducible neuroimaging marker. The most coherent signal emerges from the combination of amplified nociceptive responsivity and reduced engagement of modulatory and affective‐cognitive control systems. This overall profile is broadly compatible with central sensitization, but also fits more comprehensive nociplastic models that incorporate impaired descending modulation and non‐pain symptom dimensions.

### 4.1 Structural Brain Alterations

Structural MRI studies converge on grey matter volume and cortical thickness reductions in FM, particularly in the ACC, insula and prefrontal, notably the orbitofrontal region. Although the ACC emerged as the most frequently reported site of grey‐matter reduction across morphometry studies, this should not be interpreted as a universally replicated abnormality. Rather, it reflects the most recurrent regional finding within methodologically diverse literature, in which studies differed in analytic scope, morphometric approach and anatomical granularity of reporting. These grey‐matter losses may reflect maladaptive neuroplasticity and contribute to impaired pain regulation, as well as to the affective and cognitive burden of the syndrome. (Nakamura et al. 2025; Ong et al. 2019). While volume loss predominates, thalamic increases and other mixed findings may reflect sensory‐relay overload, where the thalamus amplifies nociceptive signals or neuroinflammatory mechanisms such as microglial activation and altered neurotransmitter dynamics (Chang et al. 2025; Chen et al. 2017). Conversely, studies in chronic pain conditions also report thalamic volume reductions linked to pain intensity and duration, suggesting possible neurodegenerative processes driven by excitotoxicity or chronic inflammation (Henderson et al. 2013; Li et al. 2020; Nakamura et al. 2025). These discrepancies likely reflect both methodological diversity and clinical variability across studies.

Methodologically, SBM offers a more cortex‐specific readout than VBM by separating cortical thickness and surface area (and related folding metrics), rather than collapsing them into a single GMV estimate. This added granularity may reveal subtler cortical patterns and partly explain method‐dependent variability across studies (Goto et al. 2022).

### 4.2 White Matter Microstructural and Structural Connectivity Alterations (DTI/SC)

Evidence from diffusion imaging and structural connectivity studies remains limited and heterogeneous. Across the small set of DTI studies, findings diverge substantially, with some reporting patterns such as lower FA together with higher RD, which may suggest altered white‐matter microstructure, although these scalar diffusion measures do not support a specific linear biological interpretation. Other studies reported increased FA with reduced diffusivity in overlapping pathways, further underscoring the uncertainty of the current DTI literature. Given this bidirectional profile and the lack of tract‐specific replication, the literature does not yet support a stable white‐matter microstructural alteration in FM.

At the network level, tractography‐based structural connectivity results are similarly mixed. Several studies suggest a less integrated structural architecture, reflected by reduced fibre counts and lower local/global efficiency or clustering, whereas others detect no global disruption or report only regionally circumscribed effects (Aster et al. 2022; Fayed et al. 2010; Kim, Kim, et al. 2015; Mosch, Hagena, Herpertz, and Diers 2023; Tu, Wang, et al. 2023). This disorganization may contribute to the cognitive symptoms and poor functional synchronization observed in FM. Overall, potential white‐matter and connectome alterations in FM remain unresolved, likely reflecting genuine biological heterogeneity coupled with significant methodological variability in acquisition and analytic pipeline.

### 4.3 Functional Brain Alterations: Resting‐State fMRI (Rs‐fMRI)

Resting‐state fMRI was one of the most extensively utilized modalities, with 32 studies contributing data. However, this domain is characterized by substantial methodological variability, which precludes the identification of a single reproducible resting‐state profile. Much of this uncertainty reflects the fact that reported results are often directionally inconsistent. The complexity stems from the fact that key findings are often contradictory. For instance, in the widely studied Default Mode Network (DMN), six studies reported increased connectivity or activity (Fallon et al. 2016; Ichesco et al. 2014, 2016; Kaplan et al. 2019; Napadow et al. 2010; Pando‐Naude et al. 2019) while five reported decreased activity (Ceko et al. 2013; Fallon et al. 2016; Jarrahi et al. 2018; Larkin et al. 2021; Liu et al. 2024). Similarly, the Salience Network (SN) and the Periaqueductal Grey (PAG) yielded mixed findings of both increased and decreased coupling. Taken together, these observations suggest a functional imbalance between internal focus and threat detection, where chronic pain becomes a salient, intrusive experience. Similar DMN–salience abnormalities (often with mixed directionality) have been consistently reported across chronic pain and affective/trauma‐related disorders, supporting a transdiagnostic interpretation of impaired network switching between internally oriented and salience‐driven processing rather than a FM‐specific rs‐fMRI signature (Fiúza‐Fernandes et al. 2025; Kaiser et al. 2015; Menon 2023; Miller et al. 2017). Rather than diminishing its relevance, this framing highlights FM as a key clinical model to examine how persistent pain, affective symptoms and hypervigilance interact at the network level—while also helping explain why rs‐fMRI results in FM differ across patient phenotypes and analytical pipelines.

The ambiguity is further amplified by the sheer methodological diversity in analytical strategies, including variations in seed placement, the use of Independent Component Analysis (ICA) versus local activity metrics (ALFF/ReHo), and the application of graph theoretical approaches. This limited convergence across studies highlights the need for harmonized protocols and meta‐analyses to synthesize the rs‐fMRI evidence more effectively.

### 4.4 Functional Brain Alterations: Task‐Based fMRI (Tb‐fMRI)

Task‐based fMRI provides the most direct and consistent empirical evidence supporting the hypothesis of central sensitization in FM, moving beyond the inconsistencies observed in structural and resting‐state studies.

#### 4.4.1 Hyper‐Responsivity to Nociceptive Stimulus

Despite the use of diverse painful stimuli (pressure, chemical, thermal) across 22 task‐based studies, the key finding is the hyper‐responsivity of the classic pain matrix. Specifically, paradigms involving pressure pain and chemical pain consistently showed increased activation across core nociceptive processing regions, including the Insula (particularly the anterior insula), the Thalamus and the Anterior Cingulate Cortex (ACC). This amplified BOLD response during noxious stimulation is the clearest functional signature of central sensitization—the heightened responsiveness of central nervous system neurons to normal or subthreshold afferent input.

#### 4.4.2 Failure of Top‐Down Modulation and Affective Control

**FIGURE 4:**
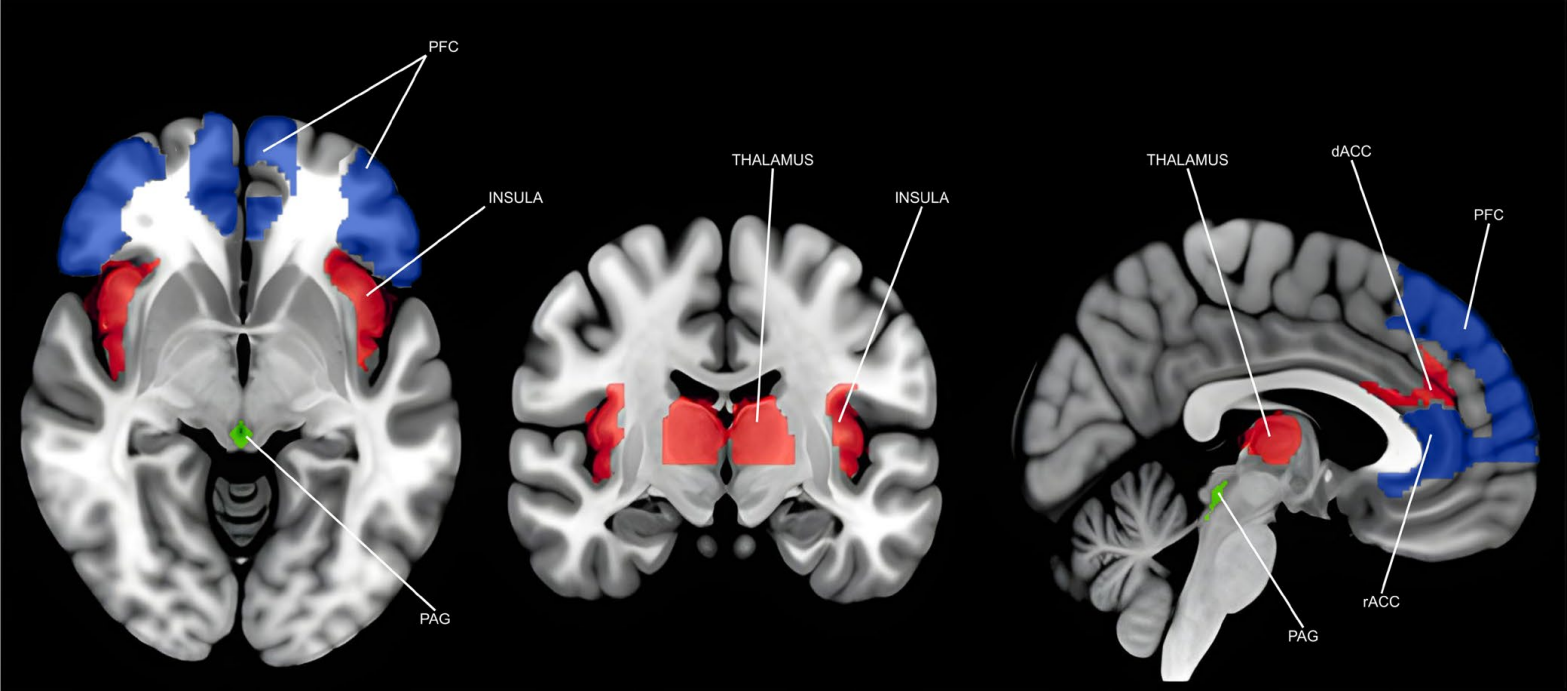
Schematic summary of the principal task‐based fMRI findings in fibromyalgia. Coloured regions summarize recurrent patterns reported across task‐based fMRI studies and should not be interpreted as a voxelwise statistical map or as findings from a single analysis. Red indicates regions commonly implicated in increased nociceptive responsivity, blue indicates prefrontal/cingulate regions involved in altered modulatory control, and green indicates the periaqueductal grey (PAG) as a key node of descending pain modulation. Arrows represent the authors' conceptual interpretation of these findings rather than direct connectivity observed in every study. ACC, anterior cingulate cortex; FM, fibromyalgia; PAG, periaqueductal grey; PFC, prefrontal cortex.

In parallel with this sensory amplification, task‐based fMRI highlights a functional impairment in the brain's regulatory mechanisms. Studies using cognitive and anticipation paradigms consistently suggest a dysregulated or decreased engagement of the prefrontal networks. This failure of *top‐down* control is crucial:

1. Impaired Descending Modulation: Decreased functional coupling involving the Periaqueductal Grey (PAG) and the rostral ACC (rACC) suggests a structural and functional compromise of the descending pain modulatory system (Figure 4).
2. Reward and Anticipation Deficits: Tasks involving reward anticipation consistently show reduced activity in the medial Prefrontal Cortex (mPFC) and the striatum (Nucleus Accumbens/Caudate). This pattern is compatible with altered mesolimbic dopaminergic processing, which has been proposed in chronic pain (Taylor et al. 2016; Wood et al. 2007) and is consistent with evidence of abnormal dopaminergic responses to pain in FM, potentially contributing to fatigue and a reduced ability to experience reward or relief.

This interpretation is also broadly consistent with complementary fMRI studies of the brainstem and spinal cord in FM. In particular, these studies have reported altered pain‐related neural signalling and connectivity during temporal summation, pain anticipation and pain stimulation (Bosma et al. 2016; Hassanpour et al. 2024; Ioachim et al. 2022), including in circuits involving the PAG, locus coeruleus (LC), hypothalamus and parabrachial nuclei (PBN), which supports the view that FM involves impaired descending pain modulation beyond cortical pain‐processing networks. Along the same lines, intervention‐based neuroimaging studies suggest that at least some of these abnormalities may be state‐dependent rather than entirely fixed, and may partially normalize with clinical improvement (Flodin et al. 2015; Jensen, Kosek, et al. 2012; Kim et al. 2013).

Importantly, the broader heterogeneity of the neuroimaging literature in FM does not preclude the possibility that specific task‐based paradigms may yield strong discriminative signals. In particular, López‐Solà et al. (2017) showed that a combined pain‐evoked and multisensory fMRI framework could distinguish FM patients from healthy controls with high cross‐validated accuracy, indicating that machine‐learning approaches applied to mechanistically grounded paradigms may represent a particularly promising avenue for future biomarker development in FM. At the same time, such findings derive from specific experimental designs and internal validation frameworks and should not yet be interpreted as evidence that the broader neuroimaging literature has established a universally robust and generalizable biomarker across modalities and clinical contexts. In this regard, the contrast with other classification‐oriented neuroimaging work, such as Robinson et al. (2015), further suggests that discriminative performance may vary substantially according to modality and study design.

Although the present review focused on MRI‐based modalities, complementary molecular imaging findings help contextualize these functional abnormalities at the neurochemical level. Positron emission tomography (PET) data suggest that FM is associated with an abnormal dopaminergic response to pain, with evidence of absent or disrupted basal ganglia dopamine release during sustained experimental pain (Wood et al. 2007). In parallel, proton magnetic resonance spectroscopy (^1^H‐MRS) studies suggest a broader glutamatergic imbalance in FM, with elevated glutamatergic metabolites reported not only in the posterior insula, where higher glutamate levels were associated with lower pressure pain thresholds, but also in other pain‐related and modulatory regions (Fayed et al. 2010; Feraco et al. 2011; Harris et al. 2009; Valdés et al. 2010). Collectively, these observations are consistent with the possibility that altered pain processing in FM may occur in the context of broader neurochemical disturbances affecting modulatory systems relevant to pain sensitivity and salience.

This neurochemical perspective also extends to systems involved in pain modulation and affective regulation. PET studies have reported reduced μ‐opioid receptor availability in FM, linked to lower pain‐evoked neural activity in antinociceptive regions such as the dorsolateral prefrontal cortex and anterior cingulate cortex, as well as to greater affective pain (Schrepf et al. 2016). Additional work has also suggested increased cortical GABA_A receptor concentration associated with pain symptoms and impaired functioning (Pomares et al. 2019), together with increased translocator protein (TSPO) binding consistent with neuroinflammatory processes in several cortical regions (Mueller et al. 2023). Overall, these findings support the view that the structural and functional abnormalities observed in FM may occur in parallel with broader neurochemical disturbances in systems relevant to pain modulation, salience and affective regulation.

Task‐based fMRI findings may represent the clearest functional expression of nociplastic pain in FM, as they integrate amplified pain‐related responsivity with impaired modulation and broader affective‐cognitive dysregulation, thereby linking the core neuroimaging findings to the clinical presentation of augmented pain and affective disturbances.

### 4.5 Implications for Future Neuroimaging Research

The findings of this review highlight a persistent gap between mechanistic neuroimaging evidence and clinical translation in FM. Although structural MRI, resting‐state fMRI, task‐based fMRI and diffusion studies have repeatedly implicated regions involved in pain processing and modulation, the specific anatomical patterns, direction of effects and analytical approaches varied substantially across studies. As a result, the current literature supports neuroimaging as a tool for investigating candidate mechanisms of nociplastic pain in FM, but not yet as a basis for diagnostic, prognostic or treatment‐response biomarkers.

A key methodological implication of this review is the need for more complete and standardized reporting of neuroimaging results. Several studies reported altered regions or networks descriptively, but did not consistently provide peak coordinates, coordinate space, cluster size, statistical thresholds, correction procedures, effect direction or contrast/task details. This limits comparability across studies and restricts the feasibility of coordinate‐based meta‐analyses, such as activation likelihood estimation or related approaches. Future FM neuroimaging studies should therefore report whole‐brain results with standardized anatomical labels, MNI or Talairach coordinates, cluster‐level statistics, direction of effects and sufficient methodological detail regarding acquisition, preprocessing and statistical modelling. Whenever possible, sharing unthresholded statistical maps and extracted datasets would further improve reproducibility and facilitate quantitative evidence synthesis.

### 4.6 Limitations

Interpretation of this review is constrained by several limitations of the primary literature. The major issue is pervasive methodological and clinical variability, which drives contradictory structural and functional findings and precluded formal quantitative meta‐analysis, limiting the ability to derive definitive pooled effects. An additional source of heterogeneity is the incomplete and inconsistent characterization of clinically relevant pain‐sensitivity phenotypes across studies. Although some task‐based studies linked brain responses to hyperalgesia, lower pain thresholds, high temporal summation of pain or reduced endogenous analgesia, allodynia and hyperalgesia were not assessed or reported in a sufficiently standardized manner to determine how strongly these features influenced the overall neuroimaging profile of FM. Clinical heterogeneity was further compounded by variable reporting of comorbidities, medication use and symptom severity across studies. Because the included studies compared FM patients with healthy controls, it is not possible to determine from the present review whether the reported neuroimaging alterations are specific to FM or reflect broader mechanisms related to chronic pain, central sensitization or common comorbidities. In addition, the scarcity of published null results suggests publication bias toward significant findings, potentially inflating observed effects. Moreover, the modified Newcastle–Ottawa Scale (NOS) is a generic tool for observational studies and does not adequately capture neuroimaging‐specific methodological risks, such as acquisition quality control, motion and physiological noise handling, preprocessing variability and multiple‐comparison correction. As a result, NOS ratings may have overestimated the true methodological rigour of several included neuroimaging studies. Uncertainty was greatest in DTI and structural connectivity, where few studies and sharply divergent results prevented identification of a stable white‐matter signature. Finally, because several findings originated from prolific research groups, partial sample overlap across publications cannot be ruled out, which may have artificially amplified the apparent consistency of specific findings.

## 5 Conclusion

This review suggests that FM involves distributed brain dysfunction, with the most consistent evidence showing grey‐matter reductions in cingulo‐insular/prefrontal regions and task‐based hyperreactivity of pain networks with impaired top‐down control, supporting nociplastic pain mechanisms. Resting‐state and diffusion results remain heterogeneous, highlighting the need for larger longitudinal, harmonized multimodal studies to establish reliable network‐level biomarkers.

## Author Contributions

F.A.D.F. and N.R.V. contributed to conceptualization and methodology. F.A.D.F. and J.P.M.C. performed formal analysis. F.A.D.F., J.P.M.C. and R.P.P.F. were responsible for investigation, data curation and writing the original draft. F.A.D.F., L.R.R., D.G.C. and N.R.V. contributed to writing – review and editing. N.R.V., L.R.R. and D.G.C. provided supervision. N.R.V. was responsible for project administration.

## Funding

The authors have nothing to report.

## Disclosure

Public data depository: https://doi.org/10.5281/zenodo.18264051.

## Conflicts of Interest

The authors declare no conflicts of interest.

## Supporting information


**Data S1:** Screening process_compressed.

**Data S2:** Search strategy.

**Data S3:** Supplemental digital content.

**Table S1:** Methodological quality assessment of included studies using the modified Newcastle–Ottawa Scale (NOS).
**Table S2:** comorbidities and clinical characteristics.
**Table S3:** Paradigm‐by‐region family counts underlying Figure 3. For each task paradigm and region‐of‐interest (ROI) family, the number of reports showing increased (hyperactivation) versus decreased (hypoactivation) task‐evoked activation in fibromyalgia versus healthy controls. Dominant direction reflects the more frequent direction (Tie/Mixed when equal). Normalized balance = (n hyperactivation − *n* hypoactivation)/7, where 7 is the largest absolute net difference across cells (range −1 to +1). Figure 3 score rescales this to 0–1 as (normalized balance + 1)/2 for the colour mapping (0 = decreased/blue, 1 = increased/red). ACC, anterior cingulate cortex; MCC, mid‐cingulate cortex; OFC, orbitofrontal cortex; PCC, posterior cingulate cortex.

## Data Availability

The datasets and Supporting Information generated and analysed during the current study are publicly available in the Zenodo repository at https://doi.org/10.5281/zenodo.20692808.
