# Supplementary material for: Structural and Functional Neuroimaging Findings in Fibromyalgia: A Systematic Review
Source: Eur J Pain. 2026 Jul 12;30(6):e70331. doi: 10.1002/ejp.70331 (PMC13356883; doi:10.1002/ejp.70331)
Supplement: Supplementary file 1 — Data S1: Screening process_compressed. [file EJP-30-0-s002.pdf]

Database	Title	Year	Authors	Duplicate	Screened	Confirmed	Eligible	Accepted	ExclusionReasons
Pubmed	Brain structural differences between fibromyalgia patients and healthy control subjects: a source-based morphometric study	2025	Agoalikum E Wu H Klugah-Brown B Maes M		YY	Y	Y	Y	
Pubmed	Chronic pain is associated with greater brain entropy in the prefrontal cortex	2025	Del Mauro G Li Y Yu J Kochunov P Sevel LS Boissoneault J Chen S Wang Z		NN				
Pubmed	Combined Functional and Structural Imaging of White Matter Reveals Brain Connectivity Alterations in Fibromyalgia Patients	2025	Gao Z Xie X Liu F Xu T Zhang N Zhang X Li Y Kong Y Lv D Wu T		NN				
Pubmed	Elevated posterior insula glutamate in patients with sickle cell disease	2025	Zhou X Ichesco E Pucka AQ Liu Z O'Brien AR Harte SE Harris RE Wang Y		NN				
Pubmed	Functional brain changes in Mexican women with fibromyalgia	2025	Elkana O Beheshti I		YY	Y	Y	Y	
Pubmed	Investigating the neural correlates of the left thalamus in women with fibromyalgia: A Granger causality and voxel-based morphometry approach	2025	Agoalikum E Wu H Klugah-Brown B Maes M		YY	Y	Y	Y	
Pubmed	Muscle and cerebral oxygenation during exercise in fibromyalgia: a near-infrared spectroscopy study	2025	Lehto T Zetterman T Gagnon D Markkula R Arokoski J Kalso E Peltonen JE		NN				
Pubmed	Neural, psychological, and daily life evidence for a transdiagnostic process of affective dysregulation in depression and chronic widespread pain	2025	Renz MP Schmidt H Drusko A Berhe O Zidda F Sebald C Andoh J Wieland S Tesarz J Treede RD Meyer-Lindenberg A Tost H		YN	Y	Y	Y	
Pubmed	Patient subtyping in juvenile fibromyalgia: the role of multisensory hypersensitivity and neurophysiological correlates	2025	Martín-Herrero L Suñol M Pascual-Diaz S Ting TV Dudley JA Jackson C Kashikar-Zuck S Coghill RC López-Solà M		YY	Y	N		Pediatric subjects.
Pubmed	Serum Interleukin-8 Levels and Their Association with Anxiety and Functional Disability in Military Personnel with Chronic Low Back Pain	2025	Dhahri R Ben Ayed H Dergaa I Ceylan Hİ Tazaghdanti A Kochkar R Ghazouani E Fenniche I Ben Ammar L Jebri R Dorgham I Slouma M Muntean RI Gharsallah I		NN				
Pubmed	Temporal interaction information and laser evoked responses: preliminary results in fibromyalgia	2025	Clemente L La Rocca M Stramaglia S Marinazzo D		NN				

	patients with small fibers pathology		Lombardi R Lauria G de Tommaso M				
Pubmed	Two Neuroanatomical Subtypes in Fibromyalgia Patients: Distinct Morphological Patterns and Treatment Outcomes	2025	Wu S Jing B Wang Y Long M Li Y Li Z Jiao J	YY	Y	Y	Y
Pubmed	Abnormal functional neurocircuitry underpinning emotional processing in fibromyalgia	2024	Balducci T Garza-Villarreal EA Valencia A Aleman A van Tol MJ	YY	Y	Y	Y
Pubmed	Alterations of the resting-state brain network connectivity and gray matter volume in patients with fibromyalgia in comparison to ankylosing spondylitis	2024	Liu D Zhang Y Zhao J Liu B Lin C Yang M Gu J Jin O	YY	Y	Y	Y
Pubmed	Amygdala self-neuromodulation capacity as a window for process-related network recruitment	2024	Gurevitch G Lubianiker N Markovits T Or-Borichev A Sharon H Fine NB Fruchtman-Steinbok T Keynan JN Shahar M Friedman A Singer N Hendler T	YN	Y	Y	Y
Pubmed	Behavioral Changes and Long-Term Cortical Thickness Alterations in Women with Fibromyalgia	2024	Oliveria Neto PG Rego Ramos L DosSantos MF	YY	Y	Y	Y
Pubmed	Cervical and Ocular Vestibular Evoked Myogenic Potentials in Fibromyalgia Syndrome Patients	2024	Dabbous AO Abdel Baki NM Hassanein MM Sheta SM	NN			
Pubmed	Effects of Different Transcranial Direct Current Stimulation Intensities over Dorsolateral Prefrontal Cortex on Brain Electrical Activity and Heart Rate Variability in Healthy and Fibromyalgia Women: A Randomized Crossover Trial	2024	Gomez-Alvaro MC Gusi N Cano-Plasencia R Leon-Llamas JL Murillo-Garcia A Melo-Alonso M Villafaina S	NN			
Pubmed	Enhanced motor network engagement during reward gain anticipation in fibromyalgia	2024	Park SH Michael AM Baker AK Lei C Martucci KT	YY	Y	Y	Y
Pubmed	Identification of texture MRI brain abnormalities on Fibromyalgia syndrome using interpretable machine learning models	2024	Jiang H Liu A Ying Z	YN	N		

Pubmed	Investigating Descending Pain Regulation in Fibromyalgia and the Link to Altered Autonomic Regulation by Means of Functional MRI Data	2024	Hassanpour S Algitami H Umraw M Merletti J Keast B Stroman PW	YY	Y	N		Wrong outcome/anatomical scope: dedicated fMRI study of the **brainstem and cervical spinal cord**, whereas the review included only **cerebral neuroimaging** studies.
Pubmed	Abnormal immune system response in the brain of women with Fibromyalgia after experimental endotoxin challenge	2023	Mueller C Jordan I Jones C Lawson P Younger JW	NN				
Pubmed	Altered Functional Networks during Gain Anticipation in Fibromyalgia	2023	Park SH Michael AM Baker AK Lei C Martucci KT	YY	Y	Y	Y	
Pubmed	Anti-satellite glia cell IgG antibodies in fibromyalgia patients are related to symptom severity and to metabolite concentrations in thalamus and rostral anterior cingulate cortex	2023	Fanton S Menezes J Krock E Sandström A Tour J Sandor K Jurczak A Hunt M Baharpoor A Kadetoff D Jensen KB Fransson P Ellerbrock I Sitnikov R Svensson CI Kosek E	NN				
Pubmed	Brain mediators of negative affect-induced physical symptom reporting in patients with functional somatic syndromes	2023	Bogaerts K Van Den Houte M Jongen D Ly HG Coppens E Schruers K Van Diest I Jan T Van Wambeke P Petre B Kragel PA Lindquist MA Wager TD Van Oudenhove L Van den Bergh O	NN				
Pubmed	Brain morphometric changes in fibromyalgia and the impact of psychometric and clinical factors: a volumetric and diffusion-tensor imaging study	2023	Mosch B Hagena V Herpertz S Diers M	YY	Y	Y	Y	
Pubmed	Characteristic oscillatory brain networks for predicting patients with chronic migraine	2023	Hsiao FJ Chen WT Wu YT Pan LH Wang YF Chen SP Lai KL Coppola G Wang SJ	NN				
Pubmed	Decreased DTI-ALPS and choroid plexus enlargement in fibromyalgia: a preliminary multimodal MRI study	2023	Tu Y Li Z Xiong F Gao F	YY	Y	Y	Y	

Pubmed	Distinct neural signaling characteristics between fibromyalgia and provoked vestibulodynia revealed by means of functional magnetic resonance imaging in the brainstem and spinal cord	2023	Ioachim G Warren HJM Powers JM Staud R Pukall CF Stroman PW	YN	N		
Pubmed	Dysfunctional Activation of the Dorsolateral Prefrontal Cortex During Pain Anticipation Is Associated With Altered Subsequent Pain Experience in Fibromyalgia Patients	2023	Sandström A Ellerbrock I Tour J Kadetoff D Jensen K Kosek E	YY	Y	Y	Y
Pubmed	Evidence of neuroinflammation in fibromyalgia syndrome: a [18 F]DPA-714 positron emission tomography study	2023	Mueller C Fang YD Jones C McConathy JE Raman F Lapi SE Younger JW	NN			
Pubmed	Functional Magnetic Resonance Imaging Signal Variability Is Associated With Neuromodulation in Fibromyalgia	2023	Lim M Kim DJ Nascimento TD Ichesco E Kaplan C Harris RE DaSilva AF	YN	Y	Y	Y
Pubmed	Microbial Composition and Stool Short Chain Fatty Acid Levels in Fibromyalgia	2023	Kim Y Kim GT Kang J	NN			
Pubmed	Neural correlates of control over pain in fibromyalgia patients	2023	Mosch B Hagena V Herpertz S Ruttorf M Diers M	YY	Y	Y	Y
Pubmed	Neuroimaging in Breast Implant Illness: An fMRI Pilot Study	2023	Miseré RML Rutten S van den Hurk J Colaris MJL van der Hulst RRWJ	NN			
Pubmed	Psychological characteristics associated with the brain volume of patients with fibromyalgia	2023	Izuno S Yoshihara K Hosoi M Eto S Hirabayashi N Todani T Gondo M Hayaki C Anno K Hiwatashi A Sudo N	YY	Y	Y	Y
Pubmed	Reduced Spinal Cord Gray Matter in Patients with Fibromyalgia Using Opioids Long-term	2023	Baker AK Park SH Weber KA 2nd Martucci KT	NN			
Pubmed	The effects of a 15-week physical exercise intervention on pain modulation in fibromyalgia: Increased pain-related processing within the cortico-striatal- occipital networks, but no improvement of exercise-induced hypoalgesia	2023	Löfgren M Sandström A Bileviciute-Ljungar I Mannerkorpi K Gerdle B Ernberg M Fransson P Kosek E	YY	Y	Y	Y

Pubmed	Topological alterations in white matter structural networks in fibromyalgia	2023	Tu Y Wang J Li Z Xiong F Gao F	YN	Y	Y	Y	
Pubmed	A behavioral and brain imaging dataset with focus on emotion regulation of women with fibromyalgia	2022	Balducci T Rasgado-Toledo J Valencia A van Tol MJ Aleman A Garza-Villarreal EA	YY	Y	Y	Y	
Pubmed	Abnormal Visual Evoked Responses to Emotional Cues Correspond to Diagnosis and Disease Severity in Fibromyalgia	2022	Goldway N Petro NM Ablin J Keil A Ben Simon E Zamir Y Weizman L Greental A Hendler T Sharon H	NN				
Pubmed	Altered Pain in the Brainstem and Spinal Cord of Fibromyalgia Patients During the Anticipation and Experience of Experimental Pain	2022	Ioachim G Warren HJM Powers JM Staud R Pukall CF Stroman PW	YY	Y	Y	Y	
Pubmed	Altered Subprocesses of Working Memory in Patients with Fibromyalgia: An Event-Related Potential Study Using N-Back Task	2022	Mercado F Ferrera D Fernandes-Magalhaes R Peláez I Barjola P	NN				
Pubmed	Altered resting-state functional connectivity within corticostriatal and subcortical-striatal circuits in chronic pain	2022	Park SH Baker AK Krishna V Mackey SC Martucci KT	YY	Y	Y	Y	
Pubmed	Assessment of retinal nerve fiber thickness and optic nerve head blood flow in female patients diagnosed with fibromyalgia syndrome	2022	Urfalıoglu S Berk E	NN				
Pubmed	Association between descending pain modulatory system and cognitive impairment in fibromyalgia: A cross-sectional exploratory study	2022	Serrano PV Zortea M Alves RL Beltran G Deliberali CB Maule A Torres ILS Fregni F Caumo W	NN				
Pubmed	Brain morphometric changes in patients with fibromyalgia	2022	Karayol KC Karayol SS	YY	Y	N		Full text cannot be retrieved
Pubmed	CNS imaging characteristics in fibromyalgia patients with and without peripheral nerve involvement	2022	Aster HC Evdokimov D Braun A Üçeyler N Kampf T Pham M Homola GA Sommer C	YY	Y	Y	Y	
Pubmed	Central pain modulatory mechanisms of attentional analgesia are preserved in fibromyalgia	2022	Oliva V Gregory R Brooks JCW Pickering AE	YY	Y	Y	Y	

Pubmed	Cortical Abnormalities in Patients with Fibromyalgia: A Pilot Study of Surface-Based Morphometry Analysis	2022	Tu Y Wang J Xiong F Gao F	YY	Y	Y	Y
Pubmed	Disrupted White Matter Microstructure in Patients With Fibromyalgia Owing Predominantly to Psychological Factors: A Diffusion Tensor Imaging Study	2022	Tu Y Wang J Xiong F Gao F	YN	Y	Y	Y
Pubmed	Distinct aberrations in cerebral pain processing differentiating patients with fibromyalgia from patients with rheumatoid arthritis	2022	Sandström A Ellerbrock I Löfgren M Altawil R Bileviciute-Ljungar I Lampa J Kosek E	NN			
Pubmed	Dynamic Functional Brain Connectivity Underlying Temporal Summation of Pain in Fibromyalgia	2022	Cheng JC Anzolin A Berry M Honari H Paschali M Lazaridou A Lee J Ellingsen DM Loggia ML Grahl A Lindquist MA Edwards RR Napadow V	YY	Y	Y	Y
Pubmed	Effect of Single Session of Anodal M1 Transcranial Direct Current Stimulation-TDCS-On Cortical Hemodynamic Activity: A Pilot Study in Fibromyalgia	2022	La Rocca M Clemente L Gentile E Ricci K Delussi M de Tommaso M	NN			
Pubmed	Effects of Transcranial Direct Current Stimulation on Brain Electrical Activity, Heart Rate Variability, and Dual-Task Performance in Healthy and Fibromyalgia Women: A Study Protocol	2022	Gomez-Alvaro MC Villafaina S Leon-Llamas JL Murillo-Garcia A Melo-Alonso M Sánchez-Gómez J Molero P Cano-Plasencia R Gusi N	NN			
Pubmed	Electrophysiological indices of pain expectation abnormalities in fibromyalgia patients	2022	Barjola P Peláez I Ferrera D González-Gutiérrez JL Velasco L Peñacoba-Puente C López-López A Fernandes-Magalhaes R Mercado F	NN			
Pubmed	Fibromyalgia: Associations Between Fat Infiltration, Physical Capacity, and Clinical Variables	2022	Gerdle B Dahlqvist Leinhard O Lund E Bengtsson A Lundberg P Ghafouri B Forsgren MF	YN	N		
Pubmed	Functional connectivity modulations during offset analgesia in chronic pain patients: an fMRI study	2022	Li T Zhang S Ikeda E Kobinata H	NN			
Pubmed	Gender influence on clinical manifestations, depressive	2022	Iannuccelli C Lucchino B Gioia C Dolcini G Rabasco J Venditto	NN			

	symptoms and brain-derived neurotrophic factor (BDNF) serum levels in patients affected by fibromyalgia		T Ioppolo F Santilli V Conti F Di Franco M	
Pubmed	Hair cortisol levels in women with medically unexplained symptoms	2022	Fischer S Skoluda N Ali N Nater UM Mewes R	NN
Pubmed	Identification of Resting-State Network Functional Connectivity and Brain Structural Signatures in Fibromyalgia Using a Machine Learning Approach	2022	Thanh Nhu N Chen DY Kang JH	N
Pubmed	Laser evoked potentials in fibromyalgia with peripheral small fiber involvement	2022	Vecchio E Quitadamo SG Ricci K Libro G Delussi M Lombardi R Lauria G de Tommaso M	NN
Pubmed	Microstructural Evidence of Neuroinflammation for Psychological Symptoms and Pain in Patients With Fibromyalgia	2022	Lo YC Li TJT Lin TC Chen YY Kang JH	NN
Pubmed	Modification of Alpha Brain Oscillatory Activity in Fibromyalgia After Very Low Intensity Transcranial Magnetic Stimulation	2022	Gomez-Arguelles JM Lopez I Rodriguez-Rojo IC Romero V Sabater C Corral M Bruna R Maestu C	NN
Pubmed	Motor cortex inhibition as a fibromyalgia biomarker: a meta-analysis of transcranial magnetic stimulation studies	2022	Pacheco-Barrios K Lima D Pimenta D Slawka E Navarro-Flores A Parente J Rebello-Sanchez I Cardenas-Rojas A Gonzalez-Mego P Castelo-Branco L Fregni F	NN
Pubmed	Movement observation activates motor cortex in fibromyalgia patients: a fNIRS study	2022	Gentile E Brunetti A Ricci K Bevilacqua V Craighero L de Tommaso M	NN
Pubmed	Multimodal MRI of myalgic encephalomyelitis/chronic fatigue syndrome: A cross-sectional neuroimaging study toward its neuropathophysiology and diagnosis	2022	Shan ZY Mohamed AZ Andersen T Rendall S Kwiatek RA Fante PD Calhoun VD Bhuta S Lagopoulos J	N
Pubmed	Neural correlates of the attentional bias towards pain-related faces in fibromyalgia patients: An ERP study using a dot-probe task	2022	Fernandes-Magalhaes R Ferrera D Peláez I Martín-Buro MC Carpio A De Lahoz ME Barjola P Mercado F	NN

Pubmed	Reduced midbrain raphe echogenicity in patients with fibromyalgia syndrome	2022	Üçeyler N Schließer M Evdokimov D Radziwon J Feulner B Unterecker S Rimmele F Walter U	NN			
Pubmed	Replication of neural responses to monetary incentives and exploration of reward-influenced network connectivity in fibromyalgia	2022	Park SH Deng EZ Baker AK MacNiven KH Knutson B Martucci KT	YY	Y	Y	Y
Pubmed	Resting-state magnetoencephalographic oscillatory connectivity to identify patients with chronic migraine using machine learning	2022	Hsiao FJ Chen WT Pan LH Liu HY Wang YF Chen SP Lai KL Coppola G Wang SJ	NN			
Pubmed	Sound-Induced Flash Illusions Support Cortex Hyperexcitability in Fibromyalgia	2022	Di Stefano V Iacono S Gagliardo A Maggio B Guggino G Gangitano M Monastero R Maggio VR Bolognini N Brighina F	NN			
Pubmed	The Impact of Micro RNA-320a Serum Level on Severity of Symptoms and Cerebral Processing of Pain in Patients with Fibromyalgia	2022	Hussein M Fathy W Abdelaleem EA Nasser M Yehia A Elanwar R	NN			
Pubmed	The translocator protein gene is associated with endogenous pain modulation and the balance between glutamate and γ-aminobutyric acid in fibromyalgia and healthy subjects: a multimodal neuroimaging study	2022	Fanton S Sandström A Tour J Kadetoff D Schalling M Jensen KB Sitnikov R Ellerbrock I Kosek E	YN	Y	Y	Y
Pubmed	A Controlled Thermoalgesic Stimulation Device for Exploring Novel Pain Perception Biomarkers	2021	Nunez-Ibero M Camino-Pontes B Diez I Erramuzpe A Martinez-Gutierrez E Stramaglia S Alvarez-Cienfuegos JO Cortes JM	NN			
Pubmed	Abnormal neuroinflammation in fibromyalgia and CRPS using [11C]-(R)-PK11195 PET	2021	Seo S Jung YH Lee D Lee WJ Jang JH Lee JY Choi SH Moon JY Lee JS Cheon GJ Kang DH	NN			
Pubmed	Abnormal neurometabolites in fibromyalgia patients: Magnetic resonance spectroscopy study	2021	Jung YH Kim H Lee D Lee JY Lee WJ Moon JY Choi SH Kang DH	NN			
Pubmed	Altered functional connectivity between hypothalamus and limbic system in fibromyalgia	2021	Kong J Huang Y Liu J Yu S Ming C Chen H Wilson G Harvey WF Li W Wang C	YY	Y	Y	Y

Pubmed	Altered network architecture of functional brain communities in chronic nociplastic pain	2021	Larkin TE Kaplan CM Schrepf A Ichesco E Mawla I Harte SE Mashour GA Clauw DJ Harris RE	YN	Y	Y	Y	
Pubmed	Central nervous activity during implicit processing of emotional face expressions in fibromyalgia syndrome	2021	Fischer-Jbali LR Montoro CI Montoya P Halder W Duschek S	NN				
Pubmed	Cerebral Perfusion and Sensory Testing Results Differ in Interstitial Cystitis/Bladder Pain Syndrome Patients with and without Fibromyalgia: A Site-Specific MAPP Network Study	2021	Deutsch G Deshpande H Lai HH Kutch JJ Ness TJ	NN				
Pubmed	Dysfunctional eating behavior in fibromyalgia and its association with serum biomarkers of brain plasticity (BDNF and S100B): an exploratory study	2021	Elkfury JL Antunes LC Dal Moro Angoleri L Sipmann RB de Souza A da Silva Torres IL Caumo W	NN				
Pubmed	Dysfunctional energy metabolisms in fibromyalgia compared with healthy subjects	2021	Jung YH Kim H Lee D Lee JY Moon JY Choi SH Kang DH	NN				
Pubmed	Fibromyalgia Patients Are Not Only Hypersensitive to Painful Stimuli But Also to Acoustic Stimuli	2021	Staud R Godfrey MM Robinson ME	NN				
Pubmed	How fMRI Analysis Using Structural Equation Modeling Techniques Can Improve Our Understanding of Pain Processing in Fibromyalgia	2021	Warren HJM Ioachim G Powers JM Stroman PW	YN	Y	Y	Y	
Pubmed	Impact of Fibromyalgia in the Hippocampal Subfields Volumes of Women-An MRI Study	2021	Leon-Llamas JL Villafaina S Murillo-Garcia A Gusi N	YN	Y	Y	Y	
Pubmed	Polymorphisms of the μ-opioid receptor gene influence cerebral pain processing in fibromyalgia	2021	Ellerbrock I Sandström A Tour J Kadetoff D Schalling M Jensen KB Kosek E	YY	Y	Y	Y	
Pubmed	Relationship between pineal gland, sleep and melatonin in fibromyalgia women: a magnetic resonance imaging study	2021	Leon-Llamas JL Villafaina S Murillo-Garcia A Rohlfs Domínguez P Gusi N	YY	Y	N		only parenchyma pineal volume/pineal gland evaluated, with not other brain measure eligible to the review

Pubmed	Serotonergic gene-to-gene interaction is associated with mood and GABA concentrations but not with pain-related cerebral processing in fibromyalgia subjects and healthy controls	2021	Ellerbrock I Sandström A Tour J Fanton S Kadetoff D Schalling M Jensen KB Sitnikov R Kosek E	NN			
Pubmed	Spinal Cord Resting State Activity in Individuals With Fibromyalgia Who Take Opioids	2021	Martucci KT Weber KA 2nd Mackey SC	YN	N		
Pubmed	Spinal cord neural activity of patients with fibromyalgia and healthy controls during temporal summation of pain: an fMRI study	2021	Staud R Boissoneault J Lai S Mejia MS Ramanlal R Godfrey MM Stroman PW	YN	N		
Pubmed	Structural and functional thalamocortical connectivity study in female fibromyalgia	2021	Kim DJ Lim M Kim JS Chung CK	YY	Y	Y	Y
Pubmed	5'UTR polymorphism in the serotonergic receptor HTR3A gene is differently associated with striatal Dopamine D2/D3 receptor availability in the right putamen in Fibromyalgia patients and healthy controls-Preliminary evidence	2020	Ledermann K Hasler G Jenewein J Sprott H Schnyder U Martin-Soelch C	NN			
Pubmed	Aberrant Salience? Brain Hyperactivation in Response to Pain Onset and Offset in Fibromyalgia	2020	Hubbard CS Lazaridou A Cahalan CM Kim J Edwards RR Napadow V Loggia ML	YY	Y	Y	Y
Pubmed	An fMRI-based neural marker for migraine without aura	2020	Tu Y Zeng F Lan L Li Z Maleki N Liu B Chen J Wang C Park J Lang C Yujie G Liu M Fu Z Zhang Z Liang F Kong J	NN			
Pubmed	Cortical Binding Potential of Opioid Receptors in Patients With Fibromyalgia Syndrome and Reduced Systemic Interleukin-4 Levels - A Pilot Study	2020	Üçeyler N Buchholz HG Kewenig S Ament SJ Birklein F Schreckenberger M Sommer C	NN			
Pubmed	DNA Methylation and Brain-Derived Neurotrophic Factor Expression Account for Symptoms and Widespread Hyperalgesia in Patients With Chronic Fatigue Syndrome and Comorbid Fibromyalgia	2020	Polli A Ghosh M Bakusic J Ickmans K Monteyne D Velkeniers B Bekaert B Godderis L Nijs J	NN			

Pubmed	Default mode network changes in fibromyalgia patients are largely dependent on current clinical pain	2020	Čeko M Frangos E Gracely J Richards E Wang B Schweinhardt P Catherine Bushnell M	YY	Y	N		Inadequate diagnose criteria
Pubmed	Exploration of Functional Connectivity Changes Previously Reported in Fibromyalgia and Their Relation to Psychological Distress and Pain Measures	2020	van Ettinger-Veenstra H Boehme R Ghafouri B Olausson H Wicksell RK Gerdle B	YY	Y	Y	Y	
Pubmed	Fibromyalgia syndrome-A laser-evoked potentials study unsupportive of small nerve fibre involvement	2020	Van Assche DCF Plaghki L Masquelier E Hatem SM	NN				
Pubmed	Hypothalamic-pituitary axis response to a 0.25-MG dexamethasone test in women with fibromyalgia	2020	González-Vives S Díaz-Marsá M De la Vega I Palomares N Vázquez S López-Villatoro JM Palomo T Carrasco JL	NN				
Pubmed	Magnetic resonance imaging of neuroinflammation in chronic pain: a role for astrogliosis?	2020	Jung C Ichesco E Ratai EM Gonzalez RG Burdo T Loggia ML Harris RE Napadow V	YN	Y	Y	Y	
Pubmed	Mutual interaction between motor cortex activation and pain in fibromyalgia: EEG-fNIRS study	2020	Gentile E Brunetti A Ricci K Delussi M Bevilacqua V de Tommaso M	NN				
Pubmed	Neural correlates of conditioned pain responses in fibromyalgia subjects indicate preferential formation of new pain associations rather than extinction of irrelevant ones	2020	Sandström A Ellerbrock I Tour J Kadetoff D Jensen KB Kosek E	YY	Y	Y	Y	
Pubmed	Neurochemical Correlates of Brain Atrophy in Fibromyalgia Syndrome: A Magnetic Resonance Spectroscopy and Cortical Thickness Study	2020	Feraco P Nigro S Passamonti L Grecucci A Caligiuri ME Gagliardo C Bacci A	YY	Y	Y	Y	
Pubmed	Neuromagnetic Amygdala Response to Pain-Related Fear as a Brain Signature of Fibromyalgia	2020	Hsiao FJ Chen WT Ko YC Liu HY Wang YF Chen SP Lai KL Lin HY Coppola G Wang SJ	NN				
Pubmed	Neurophysiological Differences Between Women With Fibromyalgia and Healthy Controls During Dual Task: A Pilot Study	2020	Villafaina S Fuentes-García JP Cano-Plasencia R Gusi N	NN				
Pubmed	Pain catastrophizing is associated with the Val66Met	2020	da Silveira Alves CF Caumo W Silvestri JM Zortea M Dos	NN				

	polymorphism of the brain-derived neurotrophic factor in fibromyalgia		Santos VS Cardoso DF Regner A de Souza AH Simon D				
Pubmed	Patients with fibromyalgia show increased beta connectivity across distant networks and microstates alterations in resting-state electroencephalogram	2020	González-Villar AJ Triñanes Y Gómez-Perretta C Carrillo-de-la-Peña MT	NN			
Pubmed	Reductions in Cerebral Blood Flow Can Be Provoked by Sitting in Severe Myalgic Encephalomyelitis/Chronic Fatigue Syndrome Patients	2020	Campen CLMV Rowe PC Visser FC	NN			
Pubmed	Striatal hypofunction as a neural correlate of mood alterations in chronic pain patients	2020	Kim M Mawla I Albrecht DS Admon R Torrado-Carvajal A Bergan C Protsenko E Kumar P Edwards RR Saha A Napadow V Pizzagalli DA Loggia ML	YY	Y	Y	Y
Pubmed	The Comparison of the Biological Rhythms of Patients with Fibromyalgia Syndrome with Biological Rhythms of Healthy Controls	2020	Bulbul F Koca I Savas E Dokuyucu R	NN			
Pubmed	Altered Cervical Spinal Cord Resting-State Activity in Fibromyalgia	2019	Martucci KT Weber KA 2nd Mackey SC	YN	N		
Pubmed	Altered resting state functional connectivity of the cognitive control network in fibromyalgia and the modulation effect of mind-body intervention	2019	Kong J Wolcott E Wang Z Jorgenson K Harvey WF Tao J Rones R Wang C	YY	Y	Y	Y
Pubmed	Apparent Effects of Opioid Use on Neural Responses to Reward in Chronic Pain	2019	Martucci KT MacNiven KH Borg N Knutson B Mackey SC	YY	Y	Y	Y
Pubmed	BDNF and serum S100B levels according the spectrum of structural pathology in chronic pain patients	2019	Stefani LC Leite FM da Graça L Tarragó M Zanette SA de Souza A Castro SM Caumo W	NN			
Pubmed	Brain Electrical Activity Associated With Visual Attention and Reactive Motor Inhibition in Patients With Fibromyalgia	2019	González-Villar AJ Arias M Carrillo-de-la-Peña MT	NN			
Pubmed	Brain glial activation in fibromyalgia - A multi-site	2019	Albrecht DS Forsberg A Sandström A Bergan C Kadetoff D Protsenko E Lampa	NN			

	positron emission tomography investigation		J Lee YC Höglund CO Catana C Cervenka S Akeju O Lekander M Cohen G Halldin C Taylor N Kim M Hooker JM Edwards RR Napadow V Kosek E Loggia ML				
Pubmed	Brain responses to other people's pain in fibromyalgia: a magnetoencephalography study	2019	Goldstein A Zeev-Wolf M Herz N Ablin JN	NN			
Pubmed	Differential Neuroplastic Changes in Fibromyalgia and Depression Indexed by Up-Regulation of Motor Cortex Inhibition and Disinhibition of the Descending Pain System: An Exploratory Study	2019	Cardinal TM Antunes LC Brietzke AP Parizotti CS Carvalho F De Souza A da Silva Torres IL Fregni F Caumo W	NN			
Pubmed	Functional connectivity of music-induced analgesia in fibromyalgia	2019	Pando-Naude V Barrios FA Alcauter S Pasaye EH Vase L Brattico E Vuust P Garza-Villarreal EA	YY	Y	Y	Y
Pubmed	Impact of Fibromyalgia on Alpha-2 EEG Power Spectrum in the Resting Condition: A Descriptive Correlational Study	2019	Villafaina S Collado-Mateo D Fuentes-García JP Cano-Plasencia R Gusi N	NN			
Pubmed	Influence of pain anticipation on brain activity and pain perception in Gulf War Veterans with chronic musculoskeletal pain	2019	Lindheimer JB Stegner AJ Ellingson-Sayen LD Van Riper SM Dougherty RJ Falvo MJ Cook DB	NN			
Pubmed	Motor Cortex Function in Fibromyalgia: A Study by Functional Near-Infrared Spectroscopy	2019	Gentile E Ricci K Delussi M Brighina F de Tommaso M	YN	N		
Pubmed	Motor cortex function in fibromyalgia: a pilot study involving near-infrared spectroscopy and co-recording of laser-evoked potentials	2019	Gentile E Ricci K Delussi M de Tommaso M	NN			
Pubmed	NYX-2925, A Novel N-methyl-D-aspartate Receptor Modulator: A First-in-Human, Randomized, Double-blind Study of Safety and Pharmacokinetics in Adults	2019	Houck DR Sindelar L Sanabria CR Stanworth SH Krueger M Suh M Madsen TM	NN			
Pubmed	Neural correlates of cognitive dysfunction in fibromyalgia patients: Reduced brain electrical	2019	Samartin-Veiga N González-Villar AJ Carrillo-de-la-Peña MT	NN			

	activity during the execution of a cognitive control task						
Pubmed	No evidence for altered plasma NGF and BDNF levels in fibromyalgia patients	2019	Baumeister D Eich W Saft S Geisel O Hellweg R Finn A Svensson CI Tesarz J	NN			
Pubmed	Nocturnal Gamma-Hydroxybutyrate Reduces Cortisol-Awakening Response and Morning Kynurenine Pathway Metabolites in Healthy Volunteers	2019	Dornbierer DA Boxler M Voegel CD Stucky B Steuer AE Binz TM Baumgartner MR Baur DM Quednow BB Kraemer T Seifritz E Landolt HP Bosch OG	NN			
Pubmed	Potency of descending pain modulatory system is linked with peripheral sensory dysfunction in fibromyalgia: An exploratory study	2019	Brietzke AP Antunes LC Carvalho F Elkifury J Gasparin A Sanches PRS da Silva Junior DP Dussán-Sarria JA Souza A da Silva Torres IL Fregni F Md WC	NN			
Pubmed	Subliminal emotional pictures are capable of modulating early cerebral responses to pain in fibromyalgia	2019	Peláez I Ferrera D Barjola P Fernandes R Mercado F	NN			
Pubmed	Subtle changes of gray matter volume in fibromyalgia reflect chronic musculoskeletal pain rather than disease-specific effects	2019	Sundermann B Dehghan Nayyeri M Pfleiderer B Stahlberg K Jünke L Baie L Dieckmann R Liem D Happe T Burgmer M	YY	Y	Y	Y
Pubmed	Unaltered low nerve growth factor and high brain-derived neurotrophic factor levels in plasma from patients with fibromyalgia after a 15-week progressive resistance exercise	2019	Jablochkova A Bäckryd E Kosek E Mannerkorpi K Ernberg M Gerdle B Ghafouri B	NN			
Pubmed	Altered theta oscillations in resting EEG of fibromyalgia syndrome patients	2018	Fallon N Chiu Y Nurmikko T Stancak A	NN			
Pubmed	Association between brain-derived neurotrophic factor gene polymorphisms and fibromyalgia in a Korean population: a multicenter study	2018	Park DJ Kim SH Nah SS Lee JH Kim SK Lee YA Hong SJ Kim HS Lee HS Kim HA Joung CI Kim SH Lee SS	NN			
Pubmed	Association of Fine Motor Loss and Allodynia in Fibromyalgia: An fNIRS Study	2018	Eken A Gökçay D Yılmaz C Baskak B Baltacı A Kara M	NN			
Pubmed	Catastrophizing Interferes with Cognitive Modulation of Pain in Women with Fibromyalgia	2018	Ellingson LD Stegner AJ Schwabacher IJ Lindheimer JB Cook DB	YY	Y	Y	Y

Pubmed	Cerebral Blood Flow Volume Using Color Duplex Sonography in Patients With Fibromyalgia Syndrome	2018	Kaya A Akgöl G Gülkesen A Poyraz AK Yildirim T Atmaca M	NN			
Pubmed	Cold Water Pressor Test Differentially Modulates Functional Network Connectivity in Fibromyalgia Patients Compared with Healthy Controls	2018	Jarrahi B Martucci KT Nilakantan AS Mackey S	YY	Y	Y	Y
Pubmed	Design and Validation of an FPGA-Based Configurable Transcranial Doppler Neurofeedback System for Chronic Pain Patients	2018	Rey B Rodríguez A Lloréns-Bufort E Tembl J Muñoz MÁ Montoya P Herrero-Bosch V Monzo JM	NN			
Pubmed	Differential efficiency of transcutaneous electrical nerve stimulation in dominant versus nondominant hands in fibromyalgia: placebo-controlled functional near-infrared spectroscopy study	2018	Eken A Kara M Baskak B Baltacı A Gökçay D	NN			
Pubmed	Disrupted Resting State Network of Fibromyalgia in Theta frequency	2018	Choe MK Lim M Kim JS Lee DS Chung CK	NN			
Pubmed	Effect of distress on transient network dynamics and topological equilibrium in phantom sound perception	2018	Mohan A Alexandra SJ Johnson CV De Ridder D Vanneste S	NN			
Pubmed	Home-Based Transcranial Direct Current Stimulation Device Development: An Updated Protocol Used at Home in Healthy Subjects and Fibromyalgia Patients	2018	Carvalho F Brietzke AP Gasparin A Dos Santos FP Vercelino R Ballester RF Sanches PRS da Silva DP Jr Torres ILS Fregni F Caumo W	NN			
Pubmed	Morphology of subcortical brain nuclei is associated with autonomic function in healthy humans	2018	Ruffle JK Coen SJ Giampietro V Williams SCR Apkarian AV Farmer AD Aziz Q	NN			
Pubmed	Novel Insights of Effects of Pregabalin on Neural Mechanisms of Intracortical Disinhibition in Physiopathology of Fibromyalgia: An Explanatory, Randomized, Double-Blind Crossover Study	2018	Deitos A Soldatelli MD Dussán-Sarria JA Souza A da Silva Torres IL Fregni F Caumo W	NN			

Pubmed	Pain Expressions and Inhibitory Control in Patients With Fibromyalgia: Behavioral and Neural Correlates	2018	Pidal-Miranda M González-Villar AJ Carrillo-de-la-Peña MT	NN			
Pubmed	Resting Functional Connectivity of the Periaqueductal Gray Is Associated With Normal Inhibition and Pathological Facilitation in Conditioned Pain Modulation	2018	Harper DE Ichesco E Schrepf A Hampson JP Clauw DJ Schmidt-Wilcke T Harris RE Harte SE	YN	Y	Y	Y
Pubmed	Salivary glutamate is elevated in individuals with chronic migraine	2018	Nam JH Lee HS Kim J Kim J Chu MK	NN			
Pubmed	The role of long-term physical exercise on performance and brain activation during the Stroop colour word task in fibromyalgia patients	2018	Martinsen S Flodin P Berrebi J Löfgren M Bileviciute-Ljungar I Mannerkorpi K Ingvar M Fransson P Kosek E	YY	Y	Y	Y
Pubmed	A study of brain metabolism in fibromyalgia by positron emission tomography	2017	Usui C Soma T Hatta K Aratani S Fujita H Nishioka K Machida Y Kuroiwa Y Nakajima T Nishioka K	NN			
Pubmed	Altered cerebral blood flow velocity features in fibromyalgia patients in resting-state conditions	2017	Rodríguez A Tembl J Mesa-Gresa P Muñoz MÁ Montoya P Rey B	NN			
Pubmed	Brain responses to vestibular pain and its anticipation in women with Genito-Pelvic Pain/Penetration Disorder	2017	Pazmany E Ly HG Aerts L Kano M Bergeron S Verhaeghe J Peeters R Tack J Dupont P Enzlin P Van Oudenhove L	NN			
Pubmed	Brain signature and functional impact of centralized pain: a multidisciplinary approach to the study of chronic pelvic pain (MAPP) network study	2017	Kutch JJ Ichesco E Hampson JP Labus JS Farmer MA Martucci KT Ness TJ Deutsch G Apkarian AV Mackey SC Klumpp DJ Schaeffer AJ Rodriguez LV Kreder KJ Buchwald D Andriole GL Lai HH Mullins C Kusek JW Landis JR Mayer EA Clemens JQ Clauw DJ Harris RE	YN	Y	Y	Y
Pubmed	Difference in Regional Brain Volume between Fibromyalgia Patients and Long-Term Meditators	2017	Fayed N García-Martí G Sanz-Requena R Marti-Bonmatí L Garcia-Campayo J	YN	Y	Y	Y
Pubmed	Electroencephalographic Evidence of Altered Top-Down Attentional Modulation in	2017	González-Villar AJ Pidal-Miranda M Arias M Rodríguez-	NN			

	Fibromyalgia Patients During a Working Memory Task		Salgado D Carrillo-de-la-Peña MT				
Pubmed	Elevations of Ventricular Lactate Levels Occur in Both Chronic Fatigue Syndrome and Fibromyalgia	2017	Natelson BH Vu D Coplan JD Mao X Blate M Kang G Soto E Kapusuz T Shungu DC	NN			
Pubmed	Epigenetics insights into chronic pain: DNA hypomethylation in fibromyalgia-a controlled pilot-study	2017	Ciampi de Andrade D Maschietto M Galhardoni R Gouveia G Chile T Victorino Krepischi AC Dale CS Brunoni AR Parravano DC Cueva Moscoso AS Raicher I Kaziyama HHS Teixeira MJ Brentani HP	NN			
Pubmed	Functional MRI of the Reserpine-Induced Putative Rat Model of Fibromyalgia Reveals Discriminatory Patterns of Functional Augmentation to Acute Nociceptive Stimuli	2017	Wells JA Shibata S Fujikawa A Takahashi M Saga T Aoki I	YN	N		
Pubmed	Histological Underpinnings of Grey Matter Changes in Fibromyalgia Investigated Using Multimodal Brain Imaging	2017	Pomares FB Funck T Feier NA Roy S Daigle-Martel A Ceko M Narayanan S Araujo D Thiel A Stikov N Fitzcharles MA Schweinhardt P	YY	Y	Y	Y
Pubmed	Investigating the BOLD spectral power of the intrinsic connectivity networks in fibromyalgia patients: A resting-state fMRI study	2017	Jarrahi B Martucci KT Nilakantan AS Mackey S	YY	Y	Y	Y
Pubmed	Lower Functional Connectivity of the Periaqueductal Gray Is Related to Negative Affect and Clinical Manifestations of Fibromyalgia	2017	Coulombe MA Lawrence KS Moulin DE Morley-Forster P Shokouhi M Nielson WR Davis KD	YY	Y	Y	Y
Pubmed	Occipital Nerve Field Transcranial Direct Current Stimulation Normalizes Imbalance Between Pain Detecting and Pain Inhibitory Pathways in Fibromyalgia	2017	De Ridder D Vanneste S	NN			
Pubmed	Painful After-Sensations in Fibromyalgia are Linked to Catastrophizing and Differences in Brain Response in the Medial Temporal Lobe	2017	Schreiber KL Loggia ML Kim J Cahalan CM Napadow V Edwards RR	YY	Y	Y	Y

Pubmed	Reduced laser-evoked potential habituation detects abnormal central pain processing in painful radiculopathy patients	2017	Hüllemann P von der Brelie C Manthey G Düsterhöft J Helmers AK Synowitz M Baron R	NN			
Pubmed	Resting state electrical brain activity and connectivity in fibromyalgia	2017	Vanneste S Ost J Van Havenbergh T De Ridder D	NN			
Pubmed	Suggestions to Reduce Clinical Fibromyalgia Pain and Experimentally Induced Pain Produce Parallel Effects on Perceived Pain but Divergent Functional MRI-Based Brain Activity	2017	Derbyshire SW Whalley MG Seah ST Oakley DA	YY	Y	Y	Y
Pubmed	Towards a neurophysiological signature for fibromyalgia	2017	López-Solà M Woo CW Pujol J Deus J Harrison BJ Monfort J Wager TD	YY	Y	Y	Y
Pubmed	rTMS of the prefrontal cortex has analgesic effects on neuropathic pain in subjects with spinal cord injury	2017	Nardone R Höller Y Langthaler PB Lochner P Golaszewski S Schwenker K Brigo F Trinka E	NN			
Pubmed	A neurometabolite study of chronic daily headache in patients with systemic lupus erythematosus using magnetic resonance spectroscopy: comparison with fibromyalgia patients and healthy controls	2016	Son CN Kim SH Chang HW Kim JM	NN			
Pubmed	A possible neural mechanism for photosensitivity in chronic pain	2016	Martenson ME Halawa OI Tonsfeldt KJ Maxwell CA Hammack N Mist SD Pennesi ME Bennett RM Mauer KM Jones KD Heinricher MM	NN			
Pubmed	Abnormal resting state functional connectivity of the periaqueductal grey in patients with fibromyalgia	2016	Truini A Tinelli E Gerardi MC Calistri V Iannuccelli C La Cesa S Tarsitani L Mainero C Sarzi-Puttini P Cruccu G Caramia F Di Franco M	YY	Y	Y	Y
Pubmed	Altered fMRI resting-state connectivity in individuals with fibromyalgia on acute pain stimulation	2016	Ichesco E Puiu T Hampson JP Kairys AE Clauw DJ Harte SE Peltier SJ Harris RE Schmidt-Wilcke T	YY	Y	Y	Y
Pubmed	Augmented Pain Processing in Primary and Secondary Somatosensory Cortex in	2016	Lim M Roosink M Kim JS Kim HW Lee EB Son KM Kim HA Chung CK	NN			

	Fibromyalgia: A Magnetoencephalography Study Using Intra-Epidermal Electrical Stimulation						
Pubmed	Cerebral vasomotor reactivity in fibromyalgia patients and its relationship to central neuropathic pain	2016	Sibel G Kurtoglu HS Kehaya S Pamuk N Celik Y	NN			
Pubmed	Characterizing "fibrofog": Subjective appraisal, objective performance, and task-related brain activity during a working memory task	2016	Walitt B Čeko M Khatiwada M Gracely JL Rayhan R VanMeter JW Gracely RH	YY	Y	Y	Y
Pubmed	Controllability and hippocampal activation during pain expectation in fibromyalgia syndrome	2016	González-Roldán AM Bomba IC Diesch E Montoya P Flor H Kamping S	YN	N		
Pubmed	Discordant Dry Eye Disease (An American Ophthalmological Society Thesis)	2016	Shtein RM Harper DE Pallazola V Harte SE Hussain M Sugar A Williams DA Clauw DJ	NN			
Pubmed	Endogenous opioidergic dysregulation of pain in fibromyalgia: a PET and fMRI study	2016	Schrepf A Harper DE Harte SE Wang H Ichesco E Hampson JP Zubieta JK Clauw DJ Harris RE	NN			
Pubmed	Evaluation of cytokines, oxidative stress markers and brain-derived neurotrophic factor in patients with fibromyalgia - A controlled cross-sectional study	2016	Ranzolin A Duarte AL Bredemeier M da Costa Neto CA Ascoli BM Wollenhaupt-Aguiar B Kapczinski F Xavier RM	NN			
Pubmed	Fibromyalgia Is Correlated with Retinal Nerve Fiber Layer Thinning	2016	Garcia-Martin E Garcia-Campayo J Puebla-Guedea M Ascaso FJ Roca M Gutierrez-Ruiz F Vilades E Polo V Larrosa JM Pablo LE Satue M	NN			
Pubmed	Functional Connectivity with the Default Mode Network Is Altered in Fibromyalgia Patients	2016	Fallon N Chiu Y Nurmikko T Stancak A	YY	Y	Y	Y
Pubmed	Habituation deficit of auditory N100m in patients with fibromyalgia	2016	Choi W Lim M Kim JS Chung CK	NN			
Pubmed	Increased Low- and High-Frequency Oscillatory Activity in the Prefrontal Cortex of Fibromyalgia Patients	2016	Lim M Kim JS Kim DJ Chung CK	NN			
Pubmed	Motor Cortex Excitability and BDNF Levels in Chronic	2016	Caumo W Deitos A Carvalho S Leite J Carvalho F Dussán-	NN			

	Musculoskeletal Pain According to Structural Pathology		Sarria JA Lopes Tarragó Mda G Souza A Torres IL Fregni F				
Pubmed	Pharmacologic attenuation of cross-modal sensory augmentation within the chronic pain insula	2016	Harte SE Ichesco E Hampson JP Peltier SJ Schmidt-Wilcke T Clauw DJ Harris RE	YY	Y	Y	Y
Pubmed	Proceedings of the 3rd IPLeiria's International Health Congress : Leiria, Portugal. 6-7 May 2016	2016	Tomás CC Oliveira E Sousa D Uba-Chupel M Furtado G Rocha C Teixeira A Ferreira P Alves C Gisin S Catarino E Carvalho N Coucelo T Bonfim L Silva C Franco D González JA Jardim HG Silva R Baixinho CL Presado MªH Marques MªF Cardoso ME Cunha M Mendes J Xavier A Galhardo A Couto M Frade JG Nunes C Mesquita JR Nascimento MS Gonçalves G Castro C Mártires A Monteiro MªJ Rainho C Caballero FP Monago FM Guerrero JT Monago RM Trigo AP Gutierrez ML Milanés GM Reina MG Villanueva AG Piñero AS Aliseda IR Ramirez FB Ribeiro A Quelhas A Manso C Caballero FP Guerrero JT Monago FM Santos RB Jimenez NR Nuñez CG Gomez IR Fernandez MªJL Marquez LA Moreno AL Huertas MªJT Ramirez FB Seabra D Salvador MªC Braga L Parreira P Salgueiro-Oliveira A Arreguy-Sena C Oliveira BF Henriques MªA Santos J Lebre S Marques A Festas C Rodrigues S Ribeiro A Lumini J Figueiredo AG Hernandez-Martinez FJ Campi L Quintana-Montesdeoca MªP Jimenez-Diaz JF Rodriguez-De-Vera BC Parente A Mata MªA Pereira AMª Fernand... ### LONG-TEXT-TRUNCATED-BY-CATCHII ###	NN			
Pubmed	Relation of dopamine receptor 2 binding to pain perception in female fibromyalgia patients with and without depression--A $[^{11}C]$ raclopride PET-study	2016	Ledermann K Jenewein J Sprott H Hasler G Schnyder U Warnock G Johayem A Kollias S Buck A Martin-Soelch C	NN			
Pubmed	Aberrant cerebral blood flow responses during cognition: Implications for the understanding of cognitive deficits in fibromyalgia	2015	Montoro CI Duschek S Muñoz Ladrón de Guevara C Fernández-Serrano MJ Reyes del Paso GA	NN			

Pubmed	Affective Modulation of Brain and Autonomic Responses in Patients With Fibromyalgia	2015	Rosselló F Muñoz MA Duschek S Montoya P	NN			
Pubmed	Altered cortical processing of observed pain in patients with fibromyalgia syndrome	2015	Fallon N Li X Chiu Y Nurmikko T Stancak A	NN			
Pubmed	Comparison of machine classification algorithms for fibromyalgia: neuroimages versus self-report	2015	Robinson ME O'Shea AM Craggs JG Price DD Letzen JE Staud R	YY	Y	Y	Y
Pubmed	Disinhibition of the primary somatosensory cortex in patients with fibromyalgia	2015	Lim M Roosink M Kim JS Kim DJ Kim HW Lee EB Kim HA Chung CK	NN			
Pubmed	Effect of Milnacipran Treatment on Ventricular Lactate in Fibromyalgia: A Randomized, Double-Blind, Placebo-Controlled Trial	2015	Natelson BH Vu D Mao X Weiduschat N Togo F Lange G Blate M Kang G Coplan JD Shungu DC	YN	N		
Pubmed	Fibromyalgia is characterized by altered frontal and cerebellar structural covariance brain networks	2015	Kim H Kim J Loggia ML Cahalan C Garcia RG Vangel MG Wasan AD Edwards RR Napadow V	YN	Y	Y	Y
Pubmed	Fibromyalgia patients have reduced hippocampal volume compared with healthy controls	2015	McCrae CS O'Shea AM Boissoneault J Vatthauer KE Robinson ME Staud R Perlstein WM Craggs JG	YY	Y	Y	Y
Pubmed	Hyperexcitability in pain matrices in patients with fibromyalgia	2015	Truini A Gerardi MC Di Stefano G La Cesa S Iannuccelli C Pepe A Sarzi-Puttini P Cruccu G Di Franco M	NN			
Pubmed	Impaired pre-attentive auditory processing in fibromyalgia: A mismatch negativity (MMN) study	2015	Choi W Lim M Kim JS Kim DJ Chung CK	NN			
Pubmed	Increased cortical activation upon painful stimulation in fibromyalgia syndrome	2015	Üçeyler N Zeller J Kewenig S Kittel-Schneider S Fallgatter AJ Sommer C	NN			
Pubmed	Normalization of aberrant resting state functional connectivity in fibromyalgia patients following a three month physical exercise therapy	2015	Flodin P Martinsen S Mannerkorpi K Löfgren M Bileviciute-Ljungar I Kosek E Fransson P	YY	Y	Y	Y
Pubmed	Reaction time, cerebral blood flow, and heart rate responses in fibromyalgia: Evidence of alterations in attentional control	2015	Reyes Del Paso GA Montoro CI Duschek S	NN			

Pubmed	Self-perspective leads to increased activation of pain processing brain regions in fibromyalgia	2015	Rahm B Lacour M Decety J Müller J Scheidt CE Bauer J König R Wirsching M Glauche V Ohlendorf S Unterbrink T Hartmann A Joos AA	YY	Y	Y	Y
Pubmed	The somatosensory link in fibromyalgia: functional connectivity of the primary somatosensory cortex is altered by sustained pain and is associated with clinical/autonomic dysfunction	2015	Kim J Loggia ML Cahalan CM Harris RE Beissner F Dr Phil Nat Garcia RG Kim H Wasan AD Edwards RR Napadow V	YY	Y	Y	Y
Pubmed	Alterations in excitatory and inhibitory brainstem interneuronal circuits in fibromyalgia: evidence of brainstem dysfunction	2014	Kofler M Halder W	NN			
Pubmed	Altered resting state connectivity of the insular cortex in individuals with fibromyalgia	2014	Ichesco E Schmidt-Wilcke T Bhavsar R Clauw DJ Peltier SJ Kim J Napadow V Hampson JP Kairys AE Williams DA Harris RE	YY	Y	Y	Y
Pubmed	Altered white matter integrity in the corpus callosum in fibromyalgia patients identified by tract-based spatial statistical analysis	2014	Kim DJ Lim M Kim JS Son KM Kim HA Chung CK	YN	Y	Y	Y
Pubmed	Changes in clinical pain in fibromyalgia patients correlate with changes in brain activation in the cingulate cortex in a response inhibition task	2014	Schmidt-Wilcke T Kairys A Ichesco E Fernandez-Sanchez ML Barjola P Heitzeg M Harris RE Clauw DJ Glass J Williams DA	YY	Y	Y	Y
Pubmed	Disrupted brain circuitry for pain-related reward/punishment in fibromyalgia	2014	Loggia ML Berna C Kim J Cahalan CM Gollub RL Wasan AD Harris RE Edwards RR Napadow V	YY	Y	Y	Y
Pubmed	Fibromyalgia is associated with decreased connectivity between pain- and sensorimotor brain areas	2014	Flodin P Martinsen S Löfgren M Bileviciute-Ljungar I Kosek E Fransson P	YN	Y	Y	Y
Pubmed	Fibromyalgia patients had normal distraction related pain inhibition but cognitive impairment reflected in caudate nucleus and hippocampus during the Stroop Color Word Test	2014	Martinsen S Flodin P Berrebi J Löfgren M Bileviciute-Ljungar I Ingvar M Fransson P Kosek E	YY	Y	Y	Y

Pubmed	Higher glutamate+glutamine and reduction of N-acetylaspartate in posterior cingulate according to age range in patients with cognitive impairment and/or pain	2014	Fayed N Andrés E Viguera L Modrego PJ Garcia-Campayo J	NN				
Pubmed	Repetitive transcranial magnetic stimulation of the left premotor/dorsolateral prefrontal cortex does not have analgesic effect on central poststroke pain	2014	de Oliveira RA de Andrade DC Mendonça M Barros R Luvisoto T Myczkowski ML Marcolin MA Teixeira MJ	NN				
Pubmed	The MAPP research network: design, patient characterization and operations	2014	Landis JR Williams DA Lucia MS Clauw DJ Naliboff BD Robinson NA van Bokhoven A Sutcliffe S Schaeffer AJ Rodriguez LV Mayer EA Lai HH Krieger JN Kreder KJ Afari N Andriole GL Bradley CS Griffith JW Klumpp DJ Hong BA Lutgendorf SK Buchwald D Yang CC Mackey S Pontari MA Hanno P Kusek JW Mullins C Clemens JQ	NN				
Pubmed	Update on laser-evoked potential findings in fibromyalgia patients in light of clinical and skin biopsy features	2014	de Tommaso M Nolano M Iannone F Vecchio E Ricci K Lorenzo M Delussi M Girolamo F Lavolpe V Provitera V Stancanelli A Lapadula G Livrea P	NN				
Pubmed	Alterations in endogenous opioid functional measures in chronic back pain	2013	Martikainen IK Peciña M Love TM Nuechterlein EB Cummiford CM Green CR Harris RE Stohler CS Zubieta JK	NN				
Pubmed	Augmented central pain processing in vulvodynia	2013	Hampson JP Reed BD Clauw DJ Bhavsar R Gracely RH Haefner HK Harris RE	NN				
Pubmed	Brain correlates of cognitive inhibition in fibromyalgia: emotional intrusion of symptom-related words	2013	Mercado F González JL Barjola P Fernández-Sánchez M López-López A Alonso M Gómez-Esquer F	NN				
Pubmed	Decreased muscle concentrations of ATP and PCR in the quadriceps muscle of fibromyalgia patients--a 31P-MRS study	2013	Gerdle B Forsgren MF Bengtsson A Leinhard OD Sören B Karlsson A Brandejsky V Lund E Lundberg P	NN				
Pubmed	Deficient modulation of pain by a positive emotional context in fibromyalgia patients	2013	Kamping S Bomba IC Kanske P Diesch E Flor H	YY	Y	Y	Y	

Pubmed	Do patients with fibromyalgia show abnormal neural responses to the observation of pain in others?	2013	Lee SJ Song HJ Decety J Seo J Kim SH Kim SH Nam EJ Kim SK Han SW Lee HJ Do Y Chang Y	YY	Y	Y	Y
Pubmed	Evaluation of the effectiveness of pregabalin in alleviating pain associated with fibromyalgia: using functional magnetic resonance imaging study	2013	Kim SH Lee Y Lee S Mun CW	YY	Y	Y	Y
Pubmed	Fibromyalgia interacts with age to change the brain	2013	Ceko M Bushnell MC Fitzcharles MA Schweinhardt P	YY	Y	Y	Y
Pubmed	Ipsilateral cortical activation in fibromyalgia patients during brushing correlates with symptom severity	2013	Fallon N Chiu YH Li X Nurmikko TJ Stancak A	NN			
Pubmed	Muscle fatigue in fibromyalgia is in the brain, not in the muscles: a case-control study of perceived versus objective muscle fatigue	2013	Bandak E Amris K Bliddal H Danneskiold-Samsøe B Henriksen M	NN			
Pubmed	Overlapping structural and functional brain changes in patients with long-term exposure to fibromyalgia pain	2013	Jensen KB Srinivasan P Spaeth R Tan Y Kosek E Petzke F Carville S Fransson P Marcus H Williams SC Choy E Vitton O Gracely R Ingvar M Kong J	YY	Y	Y	Y
Pubmed	Structural alterations in brainstem of fibromyalgia syndrome patients correlate with sensitivity to mechanical pressure	2013	Fallon N Alghamdi J Chiu Y Sluming V Nurmikko T Stancak A	YY	Y	Y	Y
Pubmed	Behavioral and neuronal investigations of hypervigilance in patients with fibromyalgia syndrome	2012	Tiemann L Schulz E Winkelmann A Ronel J Henningsen P Ploner M	NN			
Pubmed	Brain dysfunction in fibromyalgia and somatization disorder using proton magnetic resonance spectroscopy: a controlled study	2012	Fayed N Andres E Rojas G Moreno S Serrano-Blanco A Roca M Garcia-Campayo J	YN	N		
Pubmed	Cerebral mechanisms of experimental hyperalgesia in fibromyalgia	2012	Burgmer M Pfleiderer B Maihöfner C Gaubitz M Wessolleck E Heuft G Pogatzki-Zahn E	YY	Y	Y	Y
Pubmed	Comparison of the cortisol awakening response in women with shoulder and neck pain and women with fibromyalgia	2012	Riva R Mork PJ Westgaard RH Lundberg U	NN			

Pubmed	Disrupted functional connectivity of the pain network in fibromyalgia	2012	Cifre I Sitges C Fraiman D Muñoz MÁ Balenzuela P González-Roldán A Martínez-Jauand M Birbaumer N Chialvo DR Montoya P	YY	Y	Y	Y
Pubmed	Effective connectivity among brain regions associated with slow temporal summation of C-fiber-evoked pain in fibromyalgia patients and healthy controls	2012	Craggs JG Staud R Robinson ME Perlstein WM Price DD	YY	Y	Y	Y
Pubmed	Linking disease symptoms and subtypes with personalized systems-based phenotypes: a proof of concept study	2012	Aschbacher K Adam EK Crofford LJ Kemeny ME Demitrack MA Ben-Zvi A	NN			
Pubmed	Patients with fibromyalgia display less functional connectivity in the brain's pain inhibitory network	2012	Jensen KB Loitoile R Kosek E Petzke F Carville S Fransson P Marcus H Williams SC Choy E Mainguy Y Vitton O Gracely RH Gollub R Ingvar M Kong J	YY	Y	Y	Y
Pubmed	Reduced insular γ-aminobutyric acid in fibromyalgia	2012	Foerster BR Petrou M Edden RA Sundgren PC Schmidt-Wilcke T Lowe SE Harte SE Clauw DJ Harris RE	YN	N		
Pubmed	Self-ratings of higher olfactory acuity contrast with reduced olfactory test results of fibromyalgia patients	2012	Lötsch J Kraetsch HG Wendler J Hummel T	NN			
Pubmed	Working memory impairment in fibromyalgia patients associated with altered frontoparietal memory network	2012	Seo J Kim SH Kim YT Song HJ Lee JJ Kim SH Han SW Nam EJ Kim SK Lee HJ Lee SJ Chang Y	YY	Y	Y	Y
Pubmed	Central mechanisms during fatiguing muscle exercise in muscular dystrophy and fibromyalgia syndrome: a study with transcranial magnetic stimulation	2011	Schwenkreis P Voigt M Hasenbring M Tegenthoff M Vorgerd M Kley RA	NN			
Pubmed	Cerebral activation and catastrophizing during pain anticipation in patients with fibromyalgia	2011	Burgmer M Petzke F Giesecke T Gaubitz M Heuft G Pfleiderer B	YY	Y	Y	Y
Pubmed	Cerebral blood flow alterations in pain-processing regions of patients with fibromyalgia using perfusion MR imaging	2011	Foerster BR Petrou M Harris RE Barker PB Hoeffner EG Clauw DJ Sundgren PC	YN	N		

Pubmed	Differential central pain processing following repetitive intramuscular proton/prostaglandin E_2 injections in female fibromyalgia patients and healthy controls	2011	Diers M Schley MT Rance M Yilmaz P Lauer L Rukwied R Schmelz M Flor H	YN	Y	Y	Y
Pubmed	Executive function in chronic pain patients and healthy controls: different cortical activation during response inhibition in fibromyalgia	2011	Glass JM Williams DA Fernandez-Sanchez ML Kairys A Barjola P Heitzeg MM Clauw DJ Schmidt-Wilcke T	YY	Y	Y	Y
Pubmed	Gray matter volumes of pain-related brain areas are decreased in fibromyalgia syndrome	2011	Robinson ME Craggs JG Price DD Perlstein WM Staud R	YY	Y	Y	Y
Pubmed	Sleep architecture in patients with fibromyalgia	2011	Besteiro González JL Suárez Fernández TV Arboleya Rodríguez L Muñiz J Lemos Giráldez S Alvarez Fernández A	NN			
Pubmed	Alteration of delay and trace eyeblink conditioning in fibromyalgia patients	2010	Nees F Rüddel H Mussgay L Kuehl LK Römer S Schächinger H	NN			
Pubmed	Depression, anxiety, health-related quality of life and pain in patients with chronic fibromyalgia and neuropathic pain	2010	Gormsen L Rosenberg R Bach FW Jensen TS	NN			
Pubmed	Differential effects of painful and non-painful stimulation on tactile processing in fibromyalgia syndrome and subjects with masochistic behaviour	2010	Pollok B Krause V Legrain V Ploner M Freynhagen R Melchior I Schnitzler A	NN			
Pubmed	Evidence of reduced sympatho-adrenal and hypothalamic-pituitary activity during static muscular work in patients with fibromyalgia	2010	Kadetoff D Kosek E	NN			
Pubmed	Fibromyalgia unique temporal brain activation during experimental pain: a controlled fMRI Study	2010	Burgmer M Pogatzki-Zahn E Gaubitz M Stüber C Wessoleck E Heuft G Pfleiderer B	YY	Y	Y	Y
Pubmed	Increased glutamate/glutamine compounds in the brains of patients with fibromyalgia: a magnetic resonance spectroscopy study	2010	Valdés M Collado A Bargalló N Vázquez M Rami L Gómez E Salamero M	YN	N		

Pubmed	Intrinsic brain connectivity in fibromyalgia is associated with chronic pain intensity	2010	Napadow V LaCount L Park K As-Sanie S Clauw DJ Harris RE	YY	Y	Y	Y
Pubmed	Localized 1H-NMR spectroscopy in patients with fibromyalgia: a controlled study of changes in cerebral glutamate/glutamine, inositol, choline, and N-acetylaspartate	2010	Fayed N Garcia-Campayo J Magallón R Andrés-Bergareche H Luciano JV Andres E Beltrán J	YY	Y	Y	Y
Pubmed	Quantitative electroencephalographic abnormalities in fibromyalgia patients	2010	Hargrove JB Bennett RM Simons DG Smith SJ Nagpal S Deering DE	NN			
Pubmed	Salivary cortisol release and hypothalamic pituitary adrenal axis feedback sensitivity in fibromyalgia is associated with depression but not with pain	2010	Wingenfeld K Nutzinger D Kauth J Hellhammer DH Lautenbacher S	NN			
Embase	Aberrant Resting-State Effective Connectivity Between the Insula and Other Regions of the Whole Brain in Children With Obstructive Sleep Apnea	2025	Ji, T., Li, X., Xu, Z., Zhao, J., Wang, G., Li, Y., Zhang, X., Liu, Q., Sun, N., Mei, L., Wang, S., Ni, X.	NN			
Embase	Altered blood and keratinocyte microRNA/transfer RNA fragment profiles related to fibromyalgia syndrome and its severity	2025	Erbacher, C., Vaknine-Treidel, S., Madrer, N., Weinbender, S., Evdokimov, D., Unterecker, S., Moshitzky, G., Sommer, C., Greenberg, D.S., Soreq, H., Üçeyler, N.	NN			
Embase	An ALE meta-analysis of pain processing alterations in fibromyalgia: Toward an evidence-based process model	2025	Cavicchioli, M., Caruso, A., Scalabrini, A., Torelli, A., Bottiroli, S., Pichiecchio, A., Prodi, E., Cangelosi, M., Lai, C., Vitali, P., Sconfienza, L.M., Sarzi-Puttini, P., Galli, F.	NN			
Embase	Analysis of Brain Responses and Habituation to Multimodal Sensory Stimuli in Patients With Fibromyalgia	2025		NN			
Embase	Assessment of the cortical processing of pain in patients with migraine using middle latency somatosensory evoked potentials: a case–control study	2025	Essam, A.M., Mossad, M., Hussein, M., Mohammed, Z., Elanwar, R.	NN			

Embase	Brain and Spinal Cord Correlates of Deficient Endogenous Pain Modulation in Fibromyalgia Identified by Corticospinal Functional Magnetic Resonance Imaging	2025	Pfyffer, D., Kaptan, M., Law, C.S., Weber II, K.A., Oliva, V., Bédard, S., Indriolo, T., Maronesy, T., Glover, G.H., Mackey, S.		YN	N
Embase	Brain structural differences between fibromyalgia patients and healthy control subjects: a source-based morphometric study	2025	Agoalikum, E., Wu, H., Klugah-Brown, B., Maes, M.	Y		
Embase	Chronic pain is associated with greater brain entropy in the prefrontal cortex	2025	Del Mauro, G., Li, Y., Yu, J., Kochunov, P., Sevel, L.S., Boissoneault, J., Chen, S., Wang, Z.	Y		
Embase	Combined Functional and Structural Imaging of White Matter Reveals Brain Connectivity Alterations in Fibromyalgia Patients	2025	Gao, Z., Xie, X., Liu, F., Xu, T., Kong, Y., Lv, D., Wu, T., Zhang, N., Zhang, X., Li, Y.	Y		
Embase	Comparing autonomic nervous system function in patients with functional somatic syndromes, stress-related syndromes and healthy controls	2025	Van Den Houte, M., Ramakers, I., Van Oudenhove, L., Van den Bergh, O., Bogaerts, K.		NN	
Embase	Effect of Primal Reflex Release Technique on Pain and Function in Plantar Fasciitis Patients	2025			NN	
Embase	Effects of Spirulina Supplementation on Mental Health in Healthy Adults	2025			NN	
Embase	Elevated posterior insula glutamate in patients with sickle cell disease	2025	Zhou, X., Ichesco, E., Pucka, A.Q., Liu, Z., O'Brien, A.R., Harte, S.E., Harris, R.E., Wang, Y.	Y		
Embase	Expectations related to the use of theta burst stimulation protocols for pain relief. A systematic review	2025	Mussigmann, T., Bardel, B., Lefaucheur, J.-P.		NN	
Embase	Exploration of Sub-Regions of the Insula Contributions to Sensory-gating of Noxious Stimuli Using Low-intensity Focused Ultrasound	2025	Legon, W.		NN	

Embase	Exploring Sesnsorimotor Dysfunction in Fibromyalgia: Effect on Pain Sensitivity, Flexibility and Cortical Excitability	2025	Kumar, A., Kumar, U., Singh, A., Venkataraman, S., Bhatia, R.		NN
Embase	Fibromyalgia and the painful self: A meta-analysis of resting-state fMRI data	2025	Cavicchioli, M., Scalabrini, A., Nimbi, F., Torelli, A., Bottiroli, S., Pichiecchio, A., Prodi, E., Trentini, C., Sarzi-Puttini, P., Galli, F.		NN
Embase	Fibromyalgia and the painful self: A meta-analysis of restingstate fMRI data	2025	Cavicchioli, M., Scalabrini, A., Nimbi, F., Torelli, A., Bottiroli, S., Pichiecchio, A., Prodi, E., Trentini, C., Sarzi-Puttini, P., Galli, F.	Y	
Embase	Fibromyalgia in the Era of Brain PET/CT Imaging	2025	Abenavoli, E., Berti, V., Nerattini, M., Sarzi-Puttini, P., Filippou, G., Lucia, A., Pari, G., Pallanti, S., Salaffi, F., Carotti, M., Sirotti, S., Porta, F.		NN
Embase	Functional Connectivity of the Interoceptive Network in Restless Legs Syndrome (RLS) : an Anatomical-clinical Prospective Study Based on Daily-life Assessment	2025			NN
Embase	Functional brain changes in Mexican women with fibromyalgia	2025	Elkana, O., Beheshti, I.	Y	
Embase	Investigating the neural correlates of the left thalamus in women with fibromyalgia: A Granger causality and voxel-based morphometry approach	2025	Agoalikum, E., Wu, H., Klugah-Brown, B., Maes, M.	Y	
Embase	Is Cortical Motor Neuron Dysfunction the Underlying Mechanism of Fatigue in Fibromyalgia?	2025			NN
Embase	Is the brain stem a significant part of the pain network? Long-term effects of neuromodulation by SET in FM patients	2025	Thieme, K., Krahe, B.		NN
Embase	Multivariate pattern analysis reveals resting-state EEG biomarkers in fibromyalgia	2025	Soldic, D., Martín-Buro, M.C., López-García, D., del Pino, A.B., Fernandes-Magalhaes, R., Ferrera, D., Peláez, I., Carretié, L., Mercado, F.		NN

Embase	Muscle and cerebral oxygenation during exercise in fibromyalgia: a near-infrared spectroscopy study	2025	Lehto, T., Zetterman, T., Gagnon, D., Markkula, R., Arokoski, J., Kalso, E., Peltonen, J.E.	Y		
Embase	Neural, psychological, and daily life evidence for a transdiagnostic process of affective dysregulation in depression and chronic widespread pain	2025	Renz, M.P., Schmidt, H., Drusko, A., Berhe, O., Zidda, F., Sebald, C., Andoh, J., Wieland, S., Tesarz, J., Treede, R.-D., Meyer-Lindenberg, A., Tost, H.	Y		
Embase	OP0276 GUT DYSBIOSIS AND VAGUS NERVE: A PATHWAY TO CHRONIC PAIN IN RHEUMATOID ARTHRITIS	2025	Pous, A., Audo, R., Galoppin, M., Morel, J., Rivat, C., Thireau, J., Bourinet, E., Immediato Daien, C.		NN	
Embase	Pain processing and its alterations in fibromyalgia: An ALE metaanalysis of fMRI studies of multisensory task-evoked painful experiences	2025	Cavicchioli, M., Caruso, A., Scalabrini, A., Nimbi, F., Torelli, A., Bottiroli, S., Pichiecchio, A., Prodi, E., Trentini, C., Sarzi-Puttini, P., Galli, F.		YN	N
Embase	Patient subtyping in juvenile fibromyalgia: the role of multisensory hypersensitivity and neurophysiological correlates	2025	Martín-Herrero, L., Suñol, M., Pascual-Diaz, S., Ting, T.V., Dudley, J.A., Jackson, C., Kashikar-Zuck, S., Coghill, R.C., López-Solà, M.	Y		
Embase	Prevalence of the anti-CASPR2 autoantibody in patients with somatic symptom disorder accompanied by medically unexplained pain	2025	Katayama, S., Nayanar, G., Suga, T., Watanabe, M., Takao, C., Umezaki, Y., Takahashi, H., Toyofuku, A., Shiwaku, H.		NN	
Embase	Seed-based resting-state connectivity as a neurosignature in fibromyalgia and depression: a narrative systematic review	2025	Tocchetto, B.F., Moreira, A.C.J., de Oliveira Franco, Á., Torres, I.L.S., Fregni, F., Caumo, W.		NN	
Embase	Serum Interleukin-8 Levels and Their Association with Anxiety and Functional Disability in Military Personnel with Chronic Low Back Pain	2025	Dhahri, R., Ben Ayed, H., Dergaa, I., Ceylan, H.İ., Tazaghdanti, A., Kochkar, R., Ghazouani, E., Fenniche, I., Ben Ammar, L., Jebri, R., Dorgham, I., Slouma, M., Muntean, R.-I., Gharsallah, I.	Y		
Embase	Sustained Enlargement in Vagus and Sural Nerve Cross-Sectional Areas in Fibromyalgia: A Longitudinal Study	2025	Bianchi, B., Cipolletta, E., Farah, S., Salaffi, F., Di Carlo, M.		NN	

Embase	The Effects of Action Observation Speed on Motor Function in Patients with Chronic Low Back Pain: From Observation to Execution	2025	Grande-Alonso, M., Estradera-Bel, M., Forner-Álvarez, C., Cuenca-Martínez, F., Vidal-Quevedo, C., Paris-Alemany, A., La Touche, R.		NN			
Embase	The gut microbiota promotes pain in fibromyalgia	2025	Cai, W., Haddad, M., Haddad, R., Kesten, I., Hoffman, T., Laan, R., Westfall, S., Defaye, M., Abdullah, N.S., Wong, C., Brown, N., Tansley, S., Lister, K.C., Hooshmandi, M., Wang, F., Lorenzo, L.-E., Hovhannisyan, V., Ho-Tieng, D., Kumar, V., Sharif, B., Thurairajah, B., Fan, J., Sahar, T., Clayton, C., Wu, N., Zhang, J., Bar-Yoseph, H., Pitashny, M., Krock, E., Mogil, J.S., Prager-Khoutorsky, M., Séguéla, P., Altier, C., King, I.L., De Koninck, Y., Brereton, N.J.B., Gonzalez, E., Shir, Y., Minerbi, A., Khoutorsky, A.		NN			
Embase	The molecular patterns in blood, saliva and muscle and their correlation with objective CNS alterations and clinical characteristics	2025	Ghafouri, B., Berglund, T., Forsgren, M., Leinhard, O.D., Lund, E., Lundberg, P., Simon, R.		YN	Y	N	Full text cannot be retrieved
Embase	Two Neuroanatomical Subtypes in Fibromyalgia Patients: Distinct Morphological Patterns and Treatment Outcomes	2025	Wu, S., Jing, B., Wang, Y., Long, M., Li, Y., Li, Z., Jiao, J.	Y				
Embase	A systematic review of quantitative EEG findings in Fibromyalgia, Chronic Fatigue Syndrome and Long COVID	2024	Silva-Passadouro, B., Tamasauskas, A., Khoja, O., Casson, A.J., Delis, I., Brown, C., Sivan, M.		NN			
Embase	Abnormal functional neurocircuitry underpinning emotional processing in fibromyalgia	2024	Balducci, T., Garza-Villarreal, E.A., Valencia, A., Aleman, A., van Tol, M.-J.	Y				
Embase	Alterations of the resting-state brain network connectivity and gray matter volume in patients with fibromyalgia in comparison to ankylosing spondylitis	2024	Liu, D., Zhang, Y., Zhao, J., Liu, B., Lin, C., Yang, M., Gu, J., Jin, O.	Y				
Embase	Amygdala self-neuromodulation capacity as a window for process-related network recruitment	2024	Gurevitch, G., Lubianiker, N., Markovits, T., Or-Borichev, A., Sharon, H., Fine, N.B., Fruchtman-Steinbok, T., Keynan, J.N., Shahar, M.,	Y				

			Friedman, A., Singer, N., Hendler, T.					
Embase	Association between the white matter microstructure and psychological factors in patients with fibromyalgia	2024	Izuno, S., Yoshihara, K., Hosoi, M., Eto, S., Hirabayashi, N., Todani, T., Gondo, M., Hayaki, C., Anno, K., Sudo, N.		YY	Y	N	conference abstract only – no full text available.
Embase	Atrophy patterns in hippocampal subregions and their relationship with cognitive function in fibromyalgia patients with mild cognitive impairment	2024	Long, Y., Xie, X., Wang, Y., Xu, J., Gao, Z., Fang, X., Xu, T., Zhang, N., Lv, D., Wu, T.		NN			
Embase	Behavioral Changes and Long-Term Cortical Thickness Alterations in Women with Fibromyalgia	2024	Oliveria Neto, P.G.D., Rego Ramos, L., DosSantos, M.F.	Y				
Embase	Blunted sudomotor reactivity in fibromyalgia is associated with levels of depression	2024	Garcia-Hernandez, A., de la Coba, P., Reyes del Paso, G.A.		NN			
Embase	CPM deficiency in fibromyalgia: key findings and novel treatment directions	2024	Rabany, L.		NN			
Embase	Cervical and Ocular Vestibular Evoked Myogenic Potentials in Fibromyalgia Syndrome Patients	2024	Dabbous, A.O., Abdel Baki, N.M., Hassanein, M.M., Sheta, S.M.	Y				
Embase	Cervical impairments in subjects with migraine or tension type headache: an observational study	2024	del Blanco Muñiz, J.Á., Sánchez Sierra, A., Ladriñán Maestro, A., Ucero Lozano, R., Sosa-Reina, M.D., Martín Vera, D.		NN			
Embase	Clinical and Neurophysiological Effects of Simultaneous Application of tDCS to Visual and Motor Cortex in Migraine; Double Blind Randomized Controlled Study	2024			NN			
Embase	Diagnostic Test Accuracy of Histological Muscle and Skin Biopsies of Rheumatoid Arthritis Patients Revealing Objective Chronic Widespread Pain Phenomena Related to Fibromyalgia	2024			NN			
Embase	Effects of Different Transcranial Direct Current Stimulation Intensities over Dorsolateral	2024	Gomez-Alvaro, M.C., Gusi, N., Cano-Plasencia, R., Leon-	Y				

	Prefrontal Cortex on Brain Electrical Activity and Heart Rate Variability in Healthy and Fibromyalgia Women: A Randomized Crossover Trial		Llamas, J.L., Murillo-Garcia, A., Melo-Alonso, M., Villafaina, S.			
Embase	Enhanced motor network engagement during reward gain anticipation in fibromyalgia	2024	Park, S.H., Michael, A.M., Baker, A.K., Lei, C., Martucci, K.T.	Y		
Embase	Exploring Neuronal Underpinnings of Emotional Regulation of Pain in Fibromyalgia Patients	2024	Kaptan, M., Pfyffer, D., Law, C.S.W., Oliva, V., Weber, K.A., Glover, G., Mackey, S.		YN	N
Embase	Fibromyalgia and the nested hierarchical model of self: theoretical considerations based on a meta-analysis of resting-state functional connectivity studies	2024	Cavicchioli, M.		NN	
Embase	Frequency of irritable bowel syndrome in spondyloarthritis: A multicentric cross-sectional study and meta-analysis	2024	Bernard, J., Barnetche, T., Amory, C., Despres, J., Vandersmissen, M., Landrin, J., Gaujoux-Viala, C., Lukas, C., Ruyssen-Witrand, A., Truchetet, M.-E., Vergne-Salle, P., Mathieu, S., Tournadre, A.		NN	
Embase	Health conditions of first-degree relatives of children with familial Mediterranean fever	2024	Yıldırım, S., Haşlak, F., Yıldız, M., Adrovic, A., Aliyeva, A., Günalp, A., Gül, Ü., Şahin, S., Barut, K., Kasapçopur, Ö.		NN	
Embase	Hemodynamics in chronic pain: A pathway to multi-modal health risks	2024	Davydov, D.M., Galvez-Sánchez, C.M., Reyes del Paso, G.A.		NN	
Embase	Identification of texture MRI brain abnormalities on Fibromyalgia syndrome using interpretable machine learning models	2024	Jiang, H., Liu, A., Ying, Z.	Y		
Embase	Insulin Resistance, Temperament and Personality Traits Are Associated with Anhedonia in a Transdiagnostic Sample	2024	Siwek, M., Chrobak, A.A., Sołtys, Z., Dudek, D., Krupa, A.J.		NN	
Embase	Interoception in patients with fibromyalgia compared to healthy controls	2024	Ramakers, I., Houte, M.V.D., Oudenhove, L.V., Bogaerts, K.		NN	

Embase	Investigating Descending Pain Regulation in Fibromyalgia and the Link to Altered Autonomic Regulation by Means of Functional MRI Data	2024	Hassanpour, S., Algitami, H., Umraw, M., Merletti, J., Keast, B., Stroman, P.W.	Y	
Embase	Investigating Neurometabolites and Pain in Fibromyalgia	2024	Jordan, I.A., Fox, S., McDaniel, M., Younger, J.		NN
Embase	Is There a Difference in EEG Characteristics in Acute, Chronic, and Experimentally Induced Musculoskeletal Pain States? a Systematic Review	2024	Mathew, J., Perez, T.M., Adhia, D.B., De Ridder, D., Mani, R.		NN
Embase	LONG NON-CODING RNA (H19) IN AXIAL SPONDYLARTHRITIS PATIENTS: INSIGHTS INTO DIAGNOSTIC POTENTIAL AND DISEASE PARAMETERS	2024	Elzawawy, A., Soliman, E., Tayea Elsayed, E., Morsy, M.		NN
Embase	Longitudinal course of circulating miRNAs in a patient with hypophosphatasia and asfotase alfa treatment: a case report	2024	Hadzimuratovic, B., Haschka, J., Hackl, M., Diendorfer, A.B., Mittelbach, A., Feurstein, J., Zwerina, J., Resch, H., Kocijan, R.		NN
Embase	Neuroinflammation and aromatase inhibitor associated musculoskeletal syndrome (AIMSS) in breast cancer: Initial insights from [11C]PBR28 PET/MR imaging	2024	Zhu, Y., Swanson, N., Murphy, J., Kim, M., Partridge, A., Edwards, R., Schreiber, K., Loggia, M.		NN
Embase	PATIENTS WITH RHEUMATOID ARTHRITIS HAVE ACCELERATED AGE-RELATED STRUCTURAL CHANGES IN THE BRAIN	2024	Wasén, C., Dai, Z., Erlandsson, M., Andersson, K.M.E., Silfverswärd, S.T., Bokarewa, M.I., Heckemann, R.A.		NN
Embase	Pooled safety evaluation for a new single-shot live-attenuated chikungunya vaccine	2024	Maurer, G., Buerger, V., Larcher-Senn, J., Erlsbacher, F., Dubischar, K., Eder-Lingelbach, S., Jaramillo, J.C.		NN
Embase	Relieving Chronic Pain: Psychosomatic Mechanisms and Psychological Interventions in Fibromyalgia and Chronic Headache	2024			NN
Embase	The Effect of Transcutaneous Vagal Nerve Stimulation on the Processing of Visceral Pain	2024			NN

	Signals: a High Resolution FMRI Study in Healthy Volunteers							
Embase	The role of the progesterone receptor PROGINS variant in the development of fibromyalgia syndrome and its psychological findings	2024	Nursal, A.F., Cagliyan Turk, A., Kuruca, N., Yigit, S.		NN			
Embase	Visual Quantitative Sensory Testing Provides Transdiagnostic Window into Behavioral and Functional Neural Correlates of Nociplastic Pain	2024	Waller, N., Kaplan, C., Schrepf, A., Ichesco, E., Harris, R., Clauw, D., Harte, S.		YN	N		
Embase	Women with fibromyalgia: Insights into behavioral and brain imaging	2024	Elkana, O., Beheshti, I.		YY	Y	Y	Y
Embase	Yellow nail syndrome in anti-SSA and anti-SSB positive primary Sjögren’s syndrome	2024	D’Adamo, L.J., Oh, Y., Young, L.		NN			
Embase	A Pilot Study of Auricular Microstimulation to Determine if it Improves Vagal Modulation	2023			NN			
Embase	A systematic review of quantitative EEG findings in Long COVID, Fibromyalgia and Chronic Fatigue Syndrome	2023	Silva-Passadouro, B., Tamasauskas, A., Khoja, O., Casson, A.J., Delis, I., Brown, C., Sivan, M.		NN			
Embase	ASSESSMENT OF OPTIC NERVE AND MACULA FINDINGS WITH OPTIC COHERENCE ANGIOGRAPHY IN FEMALE PATIENTS WITH FIBROMYALGIA AND ITS RELATION WITH DISEASE SEVERITY	2023	Koldaş Doğan, Ş., Bulut, M., Çelik, G., Büber, H., Durmaz, D.		NN			
Embase	Abnormal immune system response in the brain of women with Fibromyalgia after experimental endotoxin challenge	2023	Mueller, C., Jordan, I., Jones, C., Lawson, P., Younger, J.W.	Y				
Embase	Alexithymia and psychological distress in fibromyalgia and chronic migraine: Clues for nociplastic pain conditions?	2023	Galli, F., Bottiroli, S., Ghiggia, A., Tassorelli, C., Lingiardi, V., Castelli, L.		NN			
Embase	Altered Dorsal And Ventral Horn Gray Matter Volumes In Long-Term Opioid-Using Fibromyalgia Patients	2023	Baker, A., Park, S.H., Weber, K.A., Martucci, K.T.		NN			

Embase	Altered Functional Networks during Gain Anticipation in Fibromyalgia	2023	Park, S.H., Michael, A.M., Baker, A.K., Lei, C., Martucci, K.T.	Y	
Embase	Altered interictal serum histamine and immunoglobulin E but unchanged tryptase levels in individuals with episodic and chronic migraine	2023	Park, C.G., Na, H.Y., Park, D.E., Kim, H.Y., Chu, M.K.		NN
Embase	Anti-satellite glia cell IgG antibodies in fibromyalgia patients are related to symptom severity and to metabolite concentrations in thalamus and rostral anterior cingulate cortex	2023	Fanton, S., Menezes, J., Krock, E., Sandström, A., Tour, J., Sandor, K., Jurczak, A., Hunt, M., Baharpoor, A., Kadetoff, D., Jensen, K.B., Fransson, P., Ellerbrock, I., Sitnikov, R., Svensson, C.I., Kosek, E.	Y	
Embase	Autoantibodies Against Surface Molecules are Altered in Fibromyalgia Syndrome	2023	Luebber, F., Strobach, J., Paulus, F.M., Heidecke, H., Graßhoff, H., Lange, T., Riemekasten, G.		NN
Embase	Brain mediators of negative affect-induced physical symptom reporting in patients with functional somatic syndromes	2023	Bogaerts, K., Van Den Houte, M., Jongen, D., Ly, H.G., Coppens, E., Schruers, K., Van Diest, I., Jan, T., Van Wambeke, P., Petre, B., Kragel, P.A., Lindquist, M.A., Wager, T.D., Van Oudenhove, L., Van den Bergh, O.	Y	
Embase	Brain morphometric changes in fibromyalgia and the impact of psychometric and clinical factors: a volumetric and diffusion-tensor imaging study	2023	Mosch, B., Hagena, V., Herpertz, S., Diers, M.	Y	
Embase	Central metabolites and peripheral parameters associated neuroinflammation in fibromyalgia patients: A preliminary study	2023	Jung, Y.-H., Kim, H., Seo, S., Lee, D., Lee, J.-Y., Moon, J.Y., Cheon, G.J., Choi, S.-H., Kang, D.-H.		NN
Embase	Characteristic oscillatory brain networks for predicting patients with chronic migraine	2023	Hsiao, F.-J., Chen, W.-T., Wu, Y.-T., Pan, L.-L.H., Wang, Y.-F., Chen, S.-P., Lai, K.-L., Coppola, G., Wang, S.-J.	Y	
Embase	Correlation Between Emotional and Psychological Symptoms and Brain Glucose Metabolism on PET/CT in Patients with Long Covid	2023	Wichert-Ana, L., Ferreira, D.L., Lopes-Santos, L.E., Trevisan, A.C., Angelis, G.D., Sakamoto, J.S., Coretti, B.G., Alexandre-Santos, L., Foss, M.P.,		NN

			Fukumori, O.Y., Kato, M., Pitella, F.A., Tumas, V., Rodrigues, F.B.					
Embase	Current Opinions about the Use of Duloxetine: Results from a Survey Aimed at Psychiatrists	2023	Alvarez-Mon, M.A., García-Montero, C., Fraile-Martinez, O., Quintero, J., Fernandez-Rojo, S., Mora, F., Gutiérrez-Rojas, L., Molina-Ruiz, R.M., Lahera, G., Álvarez-Mon, M., Ortega, M.A.		NN			
Embase	Decreased DTI-ALPS and choroid plexus enlargement in fibromyalgia: a preliminary multimodal MRI study	2023	Tu, Y., Li, Z., Xiong, F., Gao, F.	Y				
Embase	Diffusion tensor imaging of white matter microstructure in fibromyalgia: A pilot study	2023	Grazzini, I., Cuneo, G.L., La Grua, M., Paci, V., Pari, G., Spossati, G., Caccialupi, C., Malatesti, L., Venezia, D.		YY	Y	N	conference abstract only – no full text available.
Embase	Does Temporomandibular Joint Pain have a Predictive Value for Temporomandibular Joint Internal Derangement in Fibromyalgia Patients? Magnetic Resonance Imaging Role in Diagnosis	2023	Koca, C.G., Paken, G., Kösehasanoğulları, M.		NN			
Embase	Dysfunctional Activation of the Dorsolateral Prefrontal Cortex During Pain Anticipation Is Associated With Altered Subsequent Pain Experience in Fibromyalgia Patients	2023	Sandström, A., Ellerbrock, I., Tour, J., Kadetoff, D., Jensen, K., Kosek, E.	Y				
Embase	Efficacy of Unihemispheric Concurrent Dual-Site Anodal Transcranial Direct Current Stimulation Combined With Therapeutic Exercise on Pain in Fibromyalgia	2023			NN			
Embase	End-Tidal CO2 in Patients with Panic Disorder, Stress-Related or Functional Syndromes, Versus Healthy Controls	2023	Ramakers, I., Van Den Houte, M., Van Oudenhove, L., Van den Bergh, O., Bogaerts, K.		NN			
Embase	Evidence of neuroinflammation in fibromyalgia syndrome: a [18F]DPA-714 positron emission tomography study	2023	Mueller, C., Fang, Y.-H.D., Jones, C., McConathy, J.E., Raman, F., Lapi, S.E., Younger, J.W.	Y				

Embase	Fibromyalgia, mood disorders, cognitive test results, cognitive symptoms and quality of life in systemic lupus erythematosus	2023	Raghunath, S., Guymer, E.K., Glikmann-Johnston, Y., Golder, V., Kandane Rathnayake, R., Morand, E.F., Stout, J.C., Hoi, A.		NN
Embase	Functional Magnetic Resonance Imaging Signal Variability Is Associated With Neuromodulation in Fibromyalgia	2023	Lim, M., Kim, D.J., Nascimento, T.D., Ichesco, E., Kaplan, C., Harris, R.E., DaSilva, A.F.	Y	
Embase	Gut Microbiota: a Player in the Patients Pain's Sensitization With Rheumatoid Arthritis?	2023			NN
Embase	Gut microbiota promotes pain in fibromyalgia	2023	Cai, W., Haddad, M., Haddad, R., Kesten, I., Hoffman, T., Laan, R., Wong, C., Brown, N., Tansley, S., Lister, K.C., Hooshmandi, M., Wang, F., Sharif, B., Westfall, S., Sahar, T., Clayton, C., Wu, N., Zhang, J., Bar-Yoseph, H., Pitashny, M., Mogil, J.S., Prager-Khoutorsky, M., Séguéla, P., King, I.L., De Koninck, Y., Brereton, N.J.B., Gonzalez, E., Shir, Y., Minerbi, A., Khoutorsky, A.		NN
Embase	IMAGING OF THE PERIPHERAL NERVOUS SYSTEM IN NOCIPLASTIC PAIN: AN ULTRASOUND STUDY IN PATIENTS WITH FIBROMYALGIA	2023	Di Carlo, M., Bianchi, B., Cipolletta, E., Farah, S., Filippucci, E., Salaffi, F.		NN
Embase	Illness-Promoting Psychological Processes in Children and Adolescents with Functional Neurological Disorder	2023	Kozlowska, K., Schollar-Root, O., Savage, B., Hawkes, C., Chudleigh, C., Raghunandan, J., Scher, S., Helgeland, H.		NN
Embase	Imaging of the peripheral nervous system in nociplastic pain: An ultrasound study in patients with fibromyalgia	2023	Di Carlo, M., Bianchi, B., Cipolletta, E., Farah, S., Filippucci, E., Salaffi, F.	Y	
Embase	In Schizophrenia, Chronic Fatigue Syndrome-and Fibromyalgia-Like Symptoms are Driven by Breakdown of the Paracellular Pathway with Increased Zonulin and Immune Activation-Associated Neurotoxicity	2023	Maes, M., Andrés-Rodríguez, L., Vojdani, A., Sirivichayakul, S., Barbosa, D.S., Kanchanatawan, B.		NN
Embase	Insights into the field of sleep	2023	Parrino, L.		NN

Embase	Interoception in Patients With Medically Unexplained Symptoms	2023			NN			
Embase	Investigating the Neurobiological Contributions to Pain in Patients With Fibromyalgia	2023			NN			
Embase	Microbial Composition and Stool Short Chain Fatty Acid Levels in Fibromyalgia	2023	Kim, Y., Kim, G.-T., Kang, J.	Y				
Embase	Neural Correlates Of Altered Reward-Driven Attention In Chronic Pain And Opioid Use	2023	Park, S.H., Baker, A., Martucci, K.T.		YY	Y	N	conference abstract only – no full text available.
Embase	Neural correlates of control over pain in fibromyalgia patients	2023	Mosch, B., Hagena, V., Herpertz, S., Ruttorf, M., Diers, M.	Y				
Embase	Neurofeedback for Nociplastic Pain in Rheumatoid Arthritis	2023			NN			
Embase	Neuroimaging in Breast Implant Illness: An fMRI Pilot Study	2023	Miseré, R.M.L., Rutten, S., van den Hurk, J., Colaris, M.J.L., van der Hulst, R.R.W.J.	Y				
Embase	Nocebo Hyperalgesia in Patients With Fibromyalgia and Healthy Controls: An Experimental Investigation of Conditioning and Extinction Processes at Baseline and 1-Month Follow-up	2023	Karacaoglu, M., Peerdeman, K.J., Numans, M.E., Stolk, M.R., Meijer, S., Klinger, R., Veldhuijzen, D.S., van Middendorp, H., Evers, A.W.M.		NN			
Embase	PAINWAVES: THE POTENTIAL OF MACHINE LEARNING TO DIFFERENTIATE CHRONIC PAIN COHORTS USING ELECTROENCEPHALOGRAPH Y	2023	Tsigarides, J., Rushbrooke, A., Bagnall, A.		NN			
Embase	PREPULSE INHIBITION OF THE BLINK REFLEX-A PUTATIVE MARKER OF FIBROMYALGIANESS-IS ALTERED IN PTS WITH RHEUMATOID ARTHRITIS COMPARED TO HEALTHY CONTROLS, BUT NOT PREDICTIVE FOR PAIN LEVEL OR THERAPEUTIC RESPONSE TO BDMARDS	2023	Zavada, J., Forejtová, Z., Nováková, L., Serranová, T.		NN			
Embase	PREVALENCE OF INFLAMMATORY BACK PAIN IN PRIMARY SJOGREN'S	2023	Abacar, K., Colakoglu Ozkaya, S., Biyikli, E., Bugdayci, O., Atagunduz, P., Selcuk, Z.D.		NN			

	SYNDROME IS INCREASED AND ASSOCIATED WITH ACUTE AND STRUCTURAL CHANGES OF THE SACROILIAC JOINT				
Embase	Reduced Spinal Cord Gray Matter in Patients with Fibromyalgia Using Opioids Long-term	2023	Baker, A.K., Park, S.H., Weber, K.A., Martucci, K.T.	Y	
Embase	Serotonin Transporter mRNA Expression Is Reduced in the Peripheral Blood Mononuclear Cells of Subjects with Major Depression but Normal in Fibromyalgia	2023	Villanueva-Charbonneau, G., Potvin, S., Marchand, S., McIntyre, A., McIntosh, D., Bissonnette, A., Gendron, A., Giguère, C.-É., Koué, M.-È., Kouassi, É.		NN
Embase	Structural imaging studies of patients with chronic pain: An anatomical likelihood estimate meta-analysis	2023	Henn, A.T., Larsen, B., Frahm, L., Xu, A., Adebimpe, A., Scott, J.C., Linguiti, S., Sharma, V., Basbaum, A.I., Corder, G., Dworkin, R.H., Edwards, R.R., Woolf, C.J., Habel, U., Eickhoff, S.B., Eickhoff, C.R., Wagels, L., Satterthwaite, T.D.		NN
Embase	Structural integrity of corpus callosum in patients with migraine: a diffusion tensor imaging study	2023	Tantik Pak, A., Nacar Dogan, S., Sengul, Y.		NN
Embase	Subacute changes in brain functional network connectivity after nocturnal sodium oxybate intake are associated with anterior cingulate GABA	2023	Bavato, F., Esposito, F., Dornbierer, D.A., Zölch, N., Quednow, B.B., Staempfli, P., Landolt, H.-P., Seifritz, E., Bosch, O.G.		NN
Embase	The effects of a 15-week physical exercise intervention on pain modulation in fibromyalgia: Increased pain-related processing within the cortico-striatal- occipital networks, but no improvement of exercise-induced hypoalgesia	2023	Löfgren, M., Sandström, A., Bileviciute-Ljungar, I., Mannerkorpi, K., Gerdle, B., Ernberg, M., Fransson, P., Kosek, E.	Y	
Embase	The role of the brainstem in sleep disturbances and chronic pain of Gulf War and Iraq/Afghanistan veterans	2023	Zhang, Y., Moore, M., Jennings, J.S., Clark, J.D., Bayley, P.J., Ashford, J.W., Furst, A.J.		NN

Embase	The temporal expression of circulating microRNAs after acute experimental pain in humans	2023	Giordano, R., Gerra, M.C., Okutani, H., Lo Vecchio, S., Stensballe, A., Petersen, K.K.-S., Arendt-Nielsen, L.		NN				
Embase	Topological alterations in white matter structural networks in fibromyalgia	2023	Tu, Y., Wang, J., Li, Z., Xiong, F., Gao, F.	Y					
Embase	A Randomized Controlled Trial of Adjunctive D-Cycloserine to Intermittent Theta-burst Stimulation Transcranial Magnetic Stimulation in Fibromyalgia	2022			YY	Y	N		Study protocol only
Embase	A behavioral and brain imaging dataset with focus on emotion regulation of women with fibromyalgia	2022	Balducci, T., Rasgado-Toledo, J., Valencia, A., van Tol, M.-J., Aleman, A., Garza-Villarreal, E.A.	Y					
Embase	Abnormal Visual Evoked Responses to Emotional Cues Correspond to Diagnosis and Disease Severity in Fibromyalgia	2022	Goldway, N., Petro, N.M., Ablin, J., Keil, A., Ben Simon, E., Zamir, Y., Weizman, L., Greental, A., Hendler, T., Sharon, H.	Y					
Embase	Alexithymia and psychological distress in fibromyalgia and chronic migraine: A cross-sectional study	2022	Ghiggia, A., Bottiroli, S., Lingiardi, V., Tassorelli, C., Galli, F., Castelli, L.		NN				
Embase	Altered Pain Processing Associated with Administration of Dopamine Agonist and Antagonist in Healthy Volunteers	2022	Martin, S.L., Jones, A.K.P., Brown, C.A., Kobylecki, C., Whitaker, G.A., El-deredy, W., Silverdale, M.A.		NN				
Embase	Altered Pain in the Brainstem and Spinal Cord of Fibromyalgia Patients During the Anticipation and Experience of Experimental Pain	2022	Ioachim, G., Warren, H.J.M., Powers, J.M., Staud, R., Pukall, C.F., Stroman, P.W.	Y					
Embase	Altered Subprocesses of Working Memory in Patients with Fibromyalgia: An Event-Related PotentialStudy Using N-Back Task	2022	Mercado, F., Ferrera, D., Fernandes-Magalhaes, R., Peláez, I., Barjola, P.	Y					
Embase	Altered brain reward response to monetary incentives in fibromyalgia: A replication study	2022	Park, S.H., Deng, E.Z., Baker, A.K., MacNiven, K.H., Knutson, B., Martucci, K.T.		YY	Y	Y	Y	

Embase	Altered resting-state functional connectivity within corticostriatal and subcortical-striatal circuits in chronic pain	2022	Park, S.H., Baker, A.K., Krishna, V., Mackey, S.C., Martucci, K.T.	Y					
Embase	An artificial intelligence platform for movement analysis and rehabilitation: Clinical applications of stepsense to complex pain and long covid	2022	Pelah, A., Sarangi, V., de Villiers, E., Shenker, N., Stone, T., Estibeiro, P., Barenholtz, E., Levy, X., Fields, G.		NN				
Embase	Assessment of retinal nerve fiber thickness and optic nerve head blood flow in female patients diagnosed with fibromyalgia syndrome	2022	Urfalıoglu, S., Berk, E.	Y					
Embase	Assessment of retinal nerve fiber thickness and optic nerve head blood flow in female patients diagnosed with fibromyalgia syndrome	2022	Urfalıoglu, S., Berk, E.	Y					
Embase	Association between descending pain modulatory system and cognitive impairment in fibromyalgia: A cross-sectional exploratory study	2022	Serrano, P.V., Zortea, M., Alves, R.L., Beltran, G., Deliberali, C.B., Maule, A., Torres, I.L.S., Fregni, F., Caumo, W.	Y					
Embase	Attempt to replicate voxel-based morphometry analysis in fibromyalgia: Detection of below threshold differences framed by contributions of variable clinical presentation to low reproducibility	2022	Baker, A.K., Nanda, M., Park, S.H., Martucci, K.T.		YY	Y	Y	Y	
Embase	Autoimmune Mechanisms in Fibromyalgia	2022			YY	Y	N		Study protocol only
Embase	BLADDER ULTRASONOGRAPHY AS FIRST STEP NONINVASIVE APPROACH IN FEMALE PATIENTS WITH IRRITABLE BOWEL SYNDROME AND OVERACTIVE BLADDER	2022	Georgescu, D., Petre, I., Schiller, A., Georgescu, L.-A.		NN				
Embase	BRAIN MECHANISMS UNDERLYING NEGATIVE AFFECT INDUCED SOMATIC SYMPTOM REPORTING IN FUNCTIONAL SOMATIC SYNDROME PATIENTS	2022	Van Den Houte, M., Bogaerts, K., Jongen, D., Tack, J., Van Wambeke, P., Wager, T., Van Den Bergh, O., Van Oudenhove, L.		NN				

Embase	Brain morphometric changes in patients with fibromyalgia	2022	Karayol, K.C., Karayol, S.S.	Y		
Embase	CNS imaging characteristics in fibromyalgia patients with and without peripheral nerve involvement	2022	Aster, H.-C., Evdokimov, D., Braun, A., Üçeyler, N., Kampf, T., Pham, M., Homola, G.A., Sommer, C.	Y		
Embase	Central pain modulatory mechanisms of attentional analgesia are preserved in fibromyalgia	2022	Oliva, V., Gregory, R., Brooks, J.C.W., Pickering, A.E.	Y		
Embase	Cerebral hemodynamic changes to transcranial Doppler sonography in celiac disease: A pilot study	2022	Fisicaro, F., Lanza, G., D'Agate, C.C., Pennisi, M., Cantone, M., Pennisi, G., Hadjivassiliou, M., Bella, R.		NN	
Embase	Comparison of gamma-aminobutyric acid, glutamate, and N-acetylaspartate concentrations in the insular cortex between patients with fibromyalgia, rheumatoid arthritis, and healthy controls - A magnetic resonance spectroscopy study	2022	Aster, H.-C., Hahn, V., Schmalzing, M., Homola, G.A., Kampf, T., Pham, M., Üçeyler, N., Sommer, C.		YN	N
Embase	Comprehensive Investigation of Pain and Inflammation in Rheumatoid Arthritis from a Multidisciplinary Approach	2022	Tóth, L., Orsi, G., Csókási, K., Suto, G., Kumánovics, G., Császár-Nagy, N., Takács, S., Szigedi, E., Nagy, Z., Hodovány, Z., Duzsik, L., Vidnyánszky, Z., zsefKun, J., Urbán, P., Nagy, G., Helyes, Z.		NN	
Embase	Cortical Abnormalities in Patients with Fibromyalgia: A Pilot Study of Surface-Based Morphometry Analysis	2022	Tu, Y., Wang, J., Xiong, F., Gao, F.	Y		
Embase	Disrupted White Matter Microstructure in Patients With Fibromyalgia Owing Predominantly to Psychological Factors: A Diffusion Tensor Imaging Study	2022	Tu, Y., Wang, J., Xiong, F., Gao, F.	Y		
Embase	Distinct CholinomiR Blood Cell Signature as a Potential Modulator of the Cholinergic System in Women with Fibromyalgia Syndrome	2022	Erbacher, C., Vaknine, S., Moshitzky, G., Lobentanzer, S., Eisenberg, L., Evdokimov, D., Sommer, C., Greenberg, D.S., Soreq, H., Üçeyler, N.		NN	

Embase	Distinct aberrations in cerebral pain processing differentiating patients with fibromyalgia from patients with rheumatoid arthritis	2022	Sandström, A., Ellerbrock, I., Löfgren, M., Altawil, R., Bileviciute-Ljungar, I., Lampa, J., Kosek, E.	Y					
Embase	Distinctive Alterations in the Functional Anatomy of the Cerebral Cortex in Pain-sensitized Osteoarthritis and Fibromyalgia Patients	2022	Pujol, J., Ojeda, F., Blanco-Hinojo, L., Doreste, A., Martinez-Vilavella, G., Pérez-Solá, V., Deus, J., Monfort, J.		YY	Y	N		conference abstract only – no full text available.
Embase	Do biologics work in chronic pain?	2022	Choy, E.		NN				
Embase	Dynamic Functional Brain Connectivity Underlying Temporal Summation of Pain in Fibromyalgia	2022	Cheng, J.C., Anzolin, A., Berry, M., Honari, H., Paschali, M., Lazaridou, A., Lee, J., Ellingsen, D.-M., Loggia, M.L., Grahl, A., Lindquist, M.A., Edwards, R.R., Napadow, V.	Y					
Embase	EEG-heart rate connectivity changes after sensorimotor rhythm neurofeedback training: Ancillary study	2022	Alba, G., Terrasa, J.L., Vila, J., Montoya, P., Muñoz, M.A.		NN				
Embase	Effect of Single Session of Anodal M1 Transcranial Direct Current Stimulation—TDCS—On Cortical Hemodynamic Activity: A Pilot Study in Fibromyalgia	2022	Rocca, M.L., Clemente, L., Gentile, E., Ricci, K., Delussi, M., de Tommaso, M.	Y					
Embase	Effects of Transcranial Direct Current Stimulation on Brain Electrical Activity, Heart Rate Variability, and Dual-Task Performance in Healthy and Fibromyalgia Women	2022		Y					
Embase	Electrophysiological indices of pain expectation abnormalities in fibromyalgia patients	2022	Barjola, P., Peláez, I., Ferrera, D., González-Gutiérrez, J.L., Velasco, L., Peñacoba-Puente, C., López-López, A., Fernandes-Magalhaes, R., Mercado, F.	Y					
Embase	Evidence of Intact Corticostriatal and Altered Subcortical-striatal Resting-state Functional Connectivity in Chronic Pain	2022	Park, S.H., Baker, A.K., Krishna, V., Mackey, S.C., Martucci, K.T.		YY	Y	N		conference abstract only – no full text available.
Embase	Fatigue in Chronic Pain - Cognitive Functions, Emotional Aspects, Biomarkers and	2022			NN				

	Neuronal Correlates - a Descriptive Comparative Study							
Embase	Fibromyalgia: Associations Between Fat Infiltration, Physical Capacity, and Clinical Variables	2022	Gerdle, B., Leinhard, O.D., Lund, E., Bengtsson, A., Lundberg, P., Ghafouri, B., Forsgren, M.F.	Y				
Embase	Functional connectivity modulations during offset analgesia in chronic pain patients: an fMRI study	2022	Li, T., Zhang, S., Ikeda, E., Kobinata, H.	Y				
Embase	Gender influence on clinical manifestations, depressive symptoms and brain-derived neurotrophic factor (BDNF) serum levels in patients affected by fibromyalgia	2022	Iannuccelli, C., Lucchino, B., Gioia, C., Dolcini, G., Rabasco, J., Venditto, T., Ioppolo, F., Santilli, V., Conti, F., Di Franco, M.	Y				
Embase	Gray Matter Volume Abnormality in Chronic Pain Patients With Depressive Symptoms: A Systemic Review and Meta-Analysis of Voxel-Based Morphometry Studies	2022	Ma, T., Ji, Y.-Y., Yan, L.-F., Lin, J.-J., Li, Z.-Y., Wang, W., Li, J.-L., Cui, G.-B.		NN			
Embase	INVESTIGATING THE NEURAL DIFFERENCES IN INDIVIDUALS WITH CHRONIC WIDESPREAD PAIN OR FIBROMYALGIA AND THE EFFECT OF SLEEP: A CROSSSECTIONAL STUDY USING DATA FROM UK BIOBANK	2022	Silva, N.W.I.L., Soni, A., Wanigasekera, V., Zhang, S., Wall, A.J.W., Tracey, I.		YY	Y	N	conference abstract only – no full text available.
Embase	INVESTIGATING THE NEURAL DIFFERENCES IN INDIVIDUALS WITH CHRONIC WIDESPREAD PAIN OR FIBROMYALGIA AND THE EFFECT OF SLEEP: A CROSSSECTIONAL STUDY USING DATA FROM UK BIOBANK	2022	Silva, N.W.I.L., Soni, A., Wanigasekera, V., Zhang, S., Wall, A.J.W., Tracey, I.	Y				
Embase	Immunoglobulin N-glycosylation Discriminates Acute Lyme disease from Endemic Healthy Controls and Mimic Diseases - A Novel Diagnostic and Prognostic	2022	Haslund-Gourley, B., Comunale, M.A.		NN			
Embase	Impact of sleep disturbances in Juvenile Fibromyalgia Syndrome	2022	Chiarella, L., Malattia, C., Sansone, M., Pistorio, A.,		NN			

			Lavarello, C., Carpaneto, M., Ferri, R., Nobili, L.		
Embase	Impact of sleep disturbances in juvenile fibromyalgia syndrome	2022	Chiarella, L., Malattia, C., Sansone, M., Pistorio, A., Lavarello, C., Carpaneto, M., Ferri, R., Nobili, L.	Y	
Embase	Laser evoked potentials in fibromyalgia with peripheral small fiber involvement	2022	Vecchio, E., Quitadamo, S.G., Ricci, K., Libro, G., Delussi, M., Lombardi, R., Lauria, G., de Tommaso, M.	Y	
Embase	Microstructural Evidence of Neuroinflammation for Psychological Symptoms and Pain in Patients With Fibromyalgia	2022	Lo, Y.-C., Li, T.J.T., Lin, T.-C., Chen, Y.-Y., Kang, J.-H.	Y	
Embase	Modification of Alpha Brain Oscillatory Activity in Fibromyalgia After Very Low Intensity Transcranial Magnetic Stimulation	2022	Gómez-Arguelles, J.M., López, I., Rodríguez-Rojo, I.C., Romero, V., Sabater, C., Corral, M., Bruña, R., Maestú, C.	Y	
Embase	Movement observation activates motor cortex in fibromyalgia patients: a fNIRS study	2022	Gentile, E., Brunetti, A., Ricci, K., Bevilacqua, V., Craighero, L., de Tommaso, M.	Y	
Embase	Multimodal MRI of myalgic encephalomyelitis/chronic fatigue syndrome: A cross-sectional neuroimaging study toward its neuropathophysiology and diagnosis	2022	Shan, Z.Y., Mohamed, A.Z., Andersen, T., Rendall, S., Kwiatek, R.A., Fante, P.D., Calhoun, V.D., Bhuta, S., Lagopoulos, J.	Y	
Embase	Neural correlates of the attentional bias towards pain-related faces in fibromyalgia patients: An ERP study using a dot-probe task	2022	Fernandes-Magalhaes, R., Ferrera, D., Peláez, I., Martín-Buro, M.C., Carpio, A., De Lahoz, M.E., Barjola, P., Mercado, F.	Y	
Embase	No change in interictal C-reactive protein levels in individuals with episodic and chronic migraine: A case-control study and literature review	2022	Park, C.G., Lee, S.H., Chu, M.K.		NN
Embase	Potential role of blood biomarkers in patients with fibromyalgia: A systematic review with meta-analysis	2022	Kumbhare, D., Hassan, S., Diep, D., Duarte, F.C.K., Hung, J., Damodara, S., West, D.W.D., Selvaganapathy, P.R.		NN

Embase	Proprioception in patients with fibromyalgia and chronic fatigue syndrome: a systematic review	2022	Ramakers, I., Feijen, S., Vaes, M., Van Aken, K., Janssens, L., Meyns, P., Van Den Houte, M., Sercu, P., Bogaerts, K.		NN			
Embase	Reduced midbrain raphe echogenicity in patients with fibromyalgia syndrome	2022	Üçeyler, N., Schließer, M., Evdokimov, D., Radziwon, J., Feulner, B., Unterecker, S., Rimmele, F., Walter, U.	Y				
Embase	Relationship between pineal gland, sleep and melatonin in fibromyalgia women: A magnetic resonance imaging study	2022	Leon-Llamas, J.L., Villafaina, S., Murillo-Garcia, A., Rohlfs Domínguez, P., Gusi, N.		YY	Y	N	No single brain measure eligible to the review reported in the study
Embase	Replication of neural responses to monetary incentives and exploration of reward-influenced network connectivity in fibromyalgia	2022	Park, S.H., Deng, E.Z., Baker, A.K., MacNiven, K.H., Knutson, B., Martucci, K.T.	Y				
Embase	Resting-state magnetoencephalographic oscillatory connectivity to identify patients with chronic migraine using machine learning	2022	Hsiao, F.-J., Chen, W.-T., Pan, L.-L.H., Liu, H.-Y., Wang, Y.-F., Chen, S.-P., Lai, K.-L., Coppola, G., Wang, S.-J.	Y				
Embase	Self-perceived cognitive function and neuropsychological performance in women with fibromyalgia	2022	Elgueta-Aguilera, N., Guede-Rojas, F., Mendoza, C., Carvajal-Parodi, C., jerez-Mayorga, D.		NN			
Embase	Serum anti-neural immunoreactivity in patients with fibromyalgia. Preliminary study	2022	de la Cruz-Aguilera, D.L., Mendieta-Cabrera, D., Rodríguez-Pérez, C.E., Martínez-Flores, F., Hernández-Gutiérrez, M.E., Becerril-Villanueva, E., Pérez-Sánchez, G., Pavón-Romero, L., Aguirre-Cruz, L.		NN			
Embase	Sleep disturbances in newly diagnosed treatment-naïve patients with Wilson's disease	2022	Jernajczyk, W., Litwin, T., Członkowska, A., Bembenek, J.P.		NN			
Embase	Sound-Induced Flash Illusions Support Cortex Hyperexcitability in Fibromyalgia	2022	Di Stefano, V., Iacono, S., Gagliardo, A., Maggio, B., Guggino, G., Gangitano, M., Monastero, R., Maggio, V.R., Bolognini, N., Brighina, F.	Y				
Embase	Thalamic Neurometabolite Alterations in Chronic Low Back Pain: A Common Phenomenon	2022	Knight, P., Weerasekera, A., Alshelh, Z., Morrissey, E.J., Saha, A., Kim, M., Zhang, Y.,		NN			

	across Musculoskeletal Pain Conditions?		Napadow, V., Torrado-Carvajal, A., Edwards, R.R., Ratai, E.-M., Loggia, M.L.					
Embase	The Chronic Pain Brain: Structural White and Gray Matter Correlates of Impaired Muscle Control and Deficient Pain Processing	2022			YN	N		
Embase	The Impact of Micro RNA-320a Serum Level on Severity of Symptoms and Cerebral Processing of Pain in Patients with Fibromyalgia	2022	Hussein, M., Fathy, W., Abdelaleem, E.A., Nasser, M., Yehia, A., Elanwar, R.	Y				
Embase	The Impact of THC on Pain Modulation in Fibromyalgia: A Cross-Over, Randomized, Double-Blind Placebo-Controlled Study	2022			YY	Y	N	Study protocol only
Embase	The Interoceptive Sensitivity and Attention Questionnaire: Evaluating Aspects of Self-Reported Interoception in Patients with Persistent Somatic Symptoms, Stress-Related Syndromes, and Healthy Controls	2022	Bogaerts, K., Walentynowicz, M., Van Den Houte, M., Constantinou, E., Van Den Bergh, O.		NN			
Embase	The need for controllability and predictability questionnaire: Psychometric properties and first findings in a clinical sample	2022	Bogaerts, K., Ramakers, I., Fonteyne, R., Walentynowicz, M., Van Den Houte, M., Van Oudenhove, L.		NN			
Embase	The translocator protein gene is associated with endogenous pain modulation and the balance between glutamate and γ-aminobutyric acid in fibromyalgia and healthy subjects: A multimodal neuroimaging study	2022	Fanton, S., Sandström, A., Tour, J., Kadetoff, D., Schalling, M., Jensen, K.B., Sitnikov, R., Ellerbrock, I., Kosek, E.	Y				
Embase	Trial of Auricular Vagus Nerve Stimulation in Painful Covid Long	2022			NN			
Embase	Variable Voxel-based Morphometry Observations of Gray Matter Differences in Fibromyalgia: Individual Differences or Methodological Inconsistency?	2022	Baker, A., Nanda, M., Park, S.H., Martucci, K.T.		YY	Y	N	conference abstract only – no full text available.

Embase	A Controlled Thermoalgesic Stimulation Device for Exploring Novel Pain Perception Biomarkers	2021	Nunez-Ibero, M., Camino-Pontes, B., Diez, I., Erramuzpe, A., Martinez-Gutierrez, E., Stramaglia, S., Alvarez-Cienfuegos, J.O., Cortes, J.M.	Y		
Embase	Abnormal neuroinflammation in fibromyalgia and CRPS using [11C]-(R)-PK11195 PET	2021	Seo, S., Jung, Y.-H., Lee, D., Lee, W.J., Jang, J.H., Lee, J.-Y., Choi, S.-H., Moon, J.Y., Lee, J.S., Cheon, G.J., Kang, D.-H.	Y		
Embase	Abnormal neurometabolites in fibromyalgia patients: Magnetic resonance spectroscopy study	2021	Jung, Y.-H., Kim, H., Lee, D., Lee, J.-Y., Lee, W.J., Moon, J.Y., Choi, S.-H., Kang, D.-H.	Y		
Embase	Alexithymia and psychological distress in chronic migraine and fibromyalgia: A comparative study	2021	Bottiroli, S., Ghiggia, A., Galli, F., Castelli, L., Sances, G., Guaschino, E., Allena, M., Tassorelli, C.		NN	
Embase	Alexithymia and psychological distress in chronic migraine and fibromyalgia: A comparative study	2021	Bottiroli, S., Ghiggia, A., Galli, F., Castelli, L., Sances, G., Guaschino, E., Allena, M., Tassorelli, C.	Y		
Embase	Alexithymia and psychological distress in chronic migraine and fibromyalgia: A comparative study	2021	Bottiroli, S., Ghiggia, A., Galli, F., Castelli, L., Sances, G., Guaschino, E., Allena, M., Tassorelli, C.	Y		
Embase	Alterations in Gray Matter Volume and GABA in Veterans Treated with Percutaneous Electric Neural Field Stimulation (PENFS)	2021	Gebre, M., Smith, J., Krishnamurthy, L., Allen, J., Crosson, B., Napadow, V., Woodbury, A.		NN	
Embase	Altered functional connectivity between hypothalamus and limbic system in fibromyalgia	2021	Kong, J., Huang, Y., Liu, J., Yu, S., Ming, C., Chen, H., Wilson, G., Harvey, W.F., Li, W., Wang, C.	Y		
Embase	Altered network architecture of functional brain communities in chronic nociplastic pain	2021	Larkin, T.E., Kaplan, C.M., Schrepf, A., Ichesco, E., Mawla, I., Harte, S.E., Mashour, G.A., Clauw, D.J., Harris, R.E.	Y		
Embase	Altered neurochemical ratio in the prefrontal cortex is associated with pain in fibromyalgia syndrome	2021	Bishop, J., Faerman, A., Geoly, A., Maron-Katz, A., Sacchet, M., Spiegel, D., Williams, N.		YN	N
Embase	Altered sensory nerve excitability in fibromyalgia	2021	Teng, H.-W., Tani, J., Chang, T.-S., Chen, H.-J., Lin, Y.-C., Lin, C.S.-Y., Sung, J.-Y.		NN	

Embase	Assessment of Balance and Gait in Patients With Diabetic Peripheral Neuropathy	2021			NN
Embase	Assessment of New Physiological, Radiological and Activity Markers in Patients Treated by Spinal Cord Stimulation for Chronic Lower Limb Pain	2021			NN
Embase	Beyond bones: The relevance of variants of connective tissue (hypermobility) to fibromyalgia, ME/CFS and controversies surrounding diagnostic classification: An observational study	2021	Eccles, J.A., Thompson, B., Themelis, K., Amato, M.L., Stocks, R., Pound, A., Jones, A.-M., Cipinova, Z., Shah-Goodwin, L., Timeyin, J., Thompson, C.R., Batty, H.T., Harrison, N.A., Critchley, H.D., Davies, K.A.		NN
Embase	Brain metabolite concentration in pain processing regions is linked with multidimensional morbidity in fibromyalgia - a voxel-wise 3D MR Spectroscopic Imaging study	2021	Lee, J., Andronesi, O.C., Torrado-Carvajal, A., Ratai, E.-M., Loggia, M.L., Weerasekera, A., Berry, M.P., Isaro, L., Lazaridou, A., Paschali, M., Grahl, A., Wasan, A.D., Edwards, R.R., Napadow, V.		NN
Embase	Brain–immune interactions as the basis of gulf war illness: Clinical assessment and deployment profile of 1990–1991 gulf war veterans in the gulf war illness consortium (gwic) multisite case-control study	2021	Steele, L., Klimas, N., Krengel, M., Quinn, E., Toomey, R., Little, D., Abreu, M., Aenlle, K., Killiany, R., Koo, B.-B., Janulewicz, P., Heeren, T., Clark, A.N., Ajama, J., Cirillo, J., Buentello, G., Lerma, V., Coller, J.K., Sullivan, K.		NN
Embase	Central Processing of Odour Stimuli in Patients With Multi-systemic Functional Somatic Disorder, Multiple Chemical Sensitivityor Post Covid Compared to Healthy Controls - the Pilot Study	2021			NN
Embase	Central nervous activity during implicit processing of emotional face expressions in fibromyalgia syndrome	2021	Fischer-Jbali, L.R., Montoro, C.I., Montoya, P., Halder, W., Duschek, S.	Y	
Embase	Cerebral Perfusion and Sensory Testing Results Differ in	2021	Deutsch, G., Deshpande, H., Lai, H.H., Kutch, J.J., Ness, T.J.	Y	

	Interstitial Cystitis/Bladder Pain Syndrome Patients with and without Fibromyalgia: A Site-Specific MAPP Network Study							
Embase	Chronic fatigue syndrome and fibromyalgia-like symptoms are an integral component of the phenome of schizophrenia: neuro-immune and opioid system correlates	2021	Mousa, R.F., Al-Hakeim, H.K., Alhaideri, A., Maes, M.		NN			
Embase	Coenzyme q10: Clinical applications beyond cardiovascular diseases	2021	Testai, L., Martelli, A., Flori, L., Colletti, A., Cicero, A.F.G.		NN			
Embase	Differences in Passive Shoulder Range of Motion Between Baseball Players With Neurogenic Thoracic Outlet Syndrome and Matched Healthy Controls	2021	Garrison, J.C., Hannon, J.P., Conway, J.E.		NN			
Embase	Dominance of the sympathetic nervous system in patients with fibromyalgia/chronic fatigue syndrome compared to healthy controls	2021	Ramakers, I., Van Den Houte, M., Van Den Bergh, O., Van Oudenhove, L., Bogaerts, K.		NN			
Embase	Dynamic functional connectivity underlying temporal summation of pain in fibromyalgia	2021	Cheng, J.C., Anzolin, A., Berry, M., Honari, H., Paschali, M., Lazaridou, A., Lee, J., Grahl, A., Lindquist, M., Edwards, R.R., Napadow, V.		YY	Y	N	conference abstract only – no full text available.
Embase	Dysfunctional eating behavior in fibromyalgia and its association with serum biomarkers of brain plasticity (BDNF and S100B): an exploratory study	2021	Elkfury, J.L., Antunes, L.C., Dal Moro Angoleri, L., Sipmann, R.B., de Souza, A., da Silva Torres, I.L., Caumo, W.	Y				
Embase	Dysfunctional energy metabolisms in fibromyalgia compared with healthy subjects	2021	Jung, Y.-H., Kim, H., Lee, D., Lee, J.-Y., Moon, J.Y., Choi, S.-H., Kang, D.-H.	Y				
Embase	End-tidal CO2 levels in rest, during and after respiratory challenges: A comparison between patients with medically unexplained physical symptoms, panic disorder patients and healthy controls	2021	Ramakers, I., Van Den Houte, M., Van Den Bergh, O., Van Oudenhove, L., Bogaerts, K.		NN			

Embase	Enhancing Analgesia in Chronic Pain Through Exercise: Responsivity of Sensory-Motor Networks and Factors That Moderate the Analgesic Response	2021			NN
Embase	Fibromyalgia Patients Are Not Only Hypersensitive to Painful Stimuli But Also to Acoustic Stimuli	2021	Staud, R., Godfrey, M.M., Robinson, M.E.	Y	
Embase	Fibromyalgia and depression in women: An 1h-nmr metabolomic study	2021	Marino, C., Grimaldi, M., Sabatini, P., Amato, P., Pallavicino, A., Ricciardelli, C., D’Ursi, A.M.		NN
Embase	Fibromyalgia: Year in review	2021	Alciati, A., Nucera, V., Giorgi, V.		NN
Embase	Gender influence on clinical manifestations, depressive symptoms and brain-derived neurotrophic factor (bdnf) serum levels in patients affected by fibromyalgia	2021	Gioia, C., Lucchino, B., Iannuccelli, C., Dolcini, G., Di Franco, M.		NN
Embase	Graph Measure Based Connectivity in Chronic Pain Patients: A Systematic Review	2021	Lenoir, D., Cagnie, B., Verhelst, H., De Pauw, R.		NN
Embase	High beta EEG functional connectivity is decreased in the left amygdala of fibromyalgia patients in correlation with the affective component of pain	2021	Makowka, S., Mory, N., Mouthon, M., Mancini, C., Annoni, J.-M., Guggisberg, A., Chabwine, J.N.		NN
Embase	How fMRI analysis using structural equation modeling techniques can improve our understanding of pain processing in fibromyalgia	2021	Warren, H.J.M., Ioachim, G., Powers, J.M., Stroman, P.W.	Y	
Embase	Impact of fibromyalgia in the hippocampal subfields volumes of women—an mri study	2021	Leon-Llamas, J.L., Villafaina, S., Murillo-Garcia, A., Gusi, N.	Y	
Embase	Increased alpha waves in the anterior cerebral quadrants in women with fibromyalgia	2021	Santos, M., Da Cunha, E., Pereira, A., Santana, L., Lima, M., Lima, I., Marchioro, M., De Santana, J.		NN
Embase	Neural effects of placebo analgesia in fibromyalgia patients and healthy individuals	2021	Frangos, E., Čeko, M., Wang, B., Richards, E.A., Gracely, J.L., Colloca, L., Schweinhardt, P., Bushnell, M.C.		NN

Embase	Neuroimaging of fibromyalgia: Where do we stand?	2021	Torta, D.M.		NN
Embase	Neuroinflammatory and functional connectivity signatures in radicular and axial chronic low back pain	2021	Alshelh, Z., Saha, A., Morrissey, E., Kim, M., Knight, P., Albrecht, D., Torrado-Carvajal, A., Bergan, C., Zhang, Y., Akeju, O., Edwards, R., Napadow, V., Loggia, M.		NN
Embase	Nitrous Oxide as Treatment for Fibromyalgia	2021			NN
Embase	OP0086 GENDER INFLUENCE ON CLINICAL MANIFESTATIONS, DEPRESSIVE SYMPTOMS AND BRAIN-DERIVED NEUROTROPHIC FACTOR (BDNF) SERUM LEVELS IN PATIENTS AFFECTED BY FIBROMYALGIA	2021	Gioia, C., Lucchino, B., Iannuccelli, C., Dolcini, G., DI Franco, M.	Y	
Embase	Orthostatic changes in fibromyalgia & me/CFS are associated with joint hypermobility	2021	O'Brien, O.A., Eccles, J.A.		NN
Embase	Pain Induced Changes in Brain Oxyhemoglobin: A Systematic Review and Meta-Analysis of Functional NIRS Studies	2021	Hall, M., Kidgell, D., Perraton, L., Morrissey, J., Jaberzadeh, S.		NN
Embase	Polymorphisms of the μ-opioid receptor gene influence cerebral pain processing in fibromyalgia	2021	Ellerbrock, I., Sandström, A., Tour, J., Kadetoff, D., Schalling, M., Jensen, K.B., Kosek, E.	Y	
Embase	Psychological distress and alexithymia in chronic migraine and fibromyalgia: A comparative study	2021	Bottiroli, S., Ada, G., Federica, G., Castelli, L., Sances, G., Ahmad, L., Guaschino, E., Allena, M., Tassorelli, C.	Y	
Embase	Relationships between mitochondrial function, ampk, and torc1 signaling in lymphoblasts with premutation alleles of the fmr1 gene	2021	Fisher, P.R., Allan, C.Y., Sanislav, O., Atkinson, A., Ngoei, K.R.W., Kemp, B.E., Storey, E., Loesch, D.Z., Annesley, S.J.		NN
Embase	Salivary cortisol is associated with cognitive changes in patients with fibromyalgia	2021	Lin, Y.-J., Ko, Y.-C., Chow, L.-H., Hsiao, F.-J., Liu, H.-Y., Wang, P.-N., Chen, W.-T.		NN
Embase	Serotonergic gene-to-gene interaction is associated with mood and GABA concentrations	2021	Ellerbrock, I., Sandström, A., Tour, J., Fanton, S., Kadetoff,	Y	

	but not with pain-related cerebral processing in fibromyalgia subjects and healthy controls		D., Schalling, M., Jensen, K.B., Sitnikov, R., Kosek, E.		
Embase	Sound-induced flash illusions support cortex hyperexcitability in fibromyalgia	2021	Iacono, S., Di Stefano, V., Gagliardo, A., Maggio, B., Guggino, G., Gangitano, M., Monastero, R., Bolognini, N., Brighina, F.		NN
Embase	Spinal Cord Resting State Activity in Individuals With Fibromyalgia Who Take Opioids	2021	Martucci, K.T., Weber, K.A., Mackey, S.C.	Y	
Embase	Spinal cord neural activity of patients with fibromyalgia and healthy controls during temporal summation of pain: An fMRI study	2021	Staud, R., Boissoneault, J., Lai, S., Mejia, M.S., Ramanlal, R., Godfrey, M.M., Stroman, P.W.	Y	
Embase	Structural and functional thalamocortical connectivity study in female fibromyalgia	2021	Kim, D.J., Lim, M., Kim, J.S., Chung, C.K.	Y	
Embase	The Benefits of Natural Medicine, Vitamin IV Therapy and Supplements: Maintaining Good Health Through Nature	2021			NN
Embase	5UTR polymorphism in the serotonergic receptor HTR3A gene is differently associated with striatal Dopamine D2/D3 receptor availability in the right putamen in Fibromyalgia patients and healthy controls—Preliminary evidence	2020	Ledermann, K., Hasler, G., Jenewein, J., Sprott, H., Schnyder, U., Martin-Soelch, C.	Y	
Embase	A review of non-invasive brain stimulation for cognitive impairment: Evidence for novel and widespread applications	2020	Jagtap, P., Chawa, M., Pasovic, V., Gautam, M.		NN
Embase	Aberrant Salience? Brain Hyperactivation in Response to Pain Onset and Offset in Fibromyalgia	2020	Hubbard, C.S., Lazaridou, A., Cahalan, C.M., Kim, J., Edwards, R.R., Napadow, V., Loggia, M.L.	Y	
Embase	An fMRI-based neural marker for migraine without aura	2020	Tu, Y., Zeng, F., Lan, L., Li, Z., Maleki, N., Liu, B., Chen, J., Wang, C., Park, J., Lang, C., Yujie, G., Liu, M., Fu, Z., Zhang, Z., Liang, F., Kong, J.	Y	
Embase	Autonomic Function Testing: Relevance to Inflammation and Autonomic Induced Pain and	2020	Themelis, K., Amato, M., Thompson, B., Stocks, R., Pound, A., Cipinova, Z.C.,		NN

	Fatigue in Fibromyalgia and Me/CFS		Shah-Goodwin, L., Timeyin, J., Barrit, A., Harrison, N.A., Critchley, H.D., Davies, K.A., Eccles, J.A.		
Embase	Autonomic and inflammatory mechanisms of pain and fatigue in fibromyalgia and ME/CFS: An interventional study	2020	Eccles, J., Amato, M., Thompson, C., Themelis, K., Critchley, H., Harrison, N., Davies, K.		NN
Embase	Biomarker for fibromyalgia-are we (already) there	2020	Evdokimov, D., Erbacher, C., Moshitzky, G., Greenberg, D., Soreq, H., Sommer, C., Üceyler, N.		NN
Embase	Clinical Manifestations of Trauma Exposure in Fibromyalgia: The Role of Anxiety in the Association Between Posttraumatic Stress Symptoms and Fibromyalgia Status	2020	Miró, E., Martínez, M.P., Sánchez, A.I., Cáliz, R.		NN
Embase	Cortical Binding Potential of Opioid Receptors in Patients With Fibromyalgia Syndrome and Reduced Systemic Interleukin-4 Levels – A Pilot Study	2020	Üçeyler, N., Buchholz, H.-G., Kewenig, S., Ament, S.-J., Birklein, F., Schreckenberger, M., Sommer, C.	Y	
Embase	DNA Methylation and Brain-Derived Neurotrophic Factor Expression Account for Symptoms and Widespread Hyperalgesia in Patients With Chronic Fatigue Syndrome and Comorbid Fibromyalgia	2020	Polli, A., Ghosh, M., Bakusic, J., Ickmans, K., Monteyne, D., Velkeniers, B., Bekaert, B., Godderis, L., Nijs, J.	Y	
Embase	Default mode network changes in fibromyalgia patients are largely dependent on current clinical pain	2020	Čeko, M., Frangos, E., Gracely, J., Richards, E., Wang, B., Schweinhardt, P., Catherine Bushnell, M.	Y	
Embase	Effects of Acute Exercise on BDNF Levels in Rheumatoid Arthritis Patients	2020			NN
Embase	Electroencephalography during nociceptive stimulation in chronic pain patients: A systematic review	2020	Lenoir, D., Willaert, W., Coppieters, I., Malfliet, A., Ickmans, K., Nijs, J., Vonck, K., Meeus, M., Cagnie, B.		NN
Embase	Epigenetic and miRNA Expression Changes in People with Pain: A Systematic Review	2020	Polli, A., Godderis, L., Ghosh, M., Ickmans, K., Nijs, J.		NN

Embase	Evaluation of olfactory and gustatory functions in patients with fibromyalgia syndrome: Its relationship with anxiety, depression, and alexithymia	2020	Özsoy-ünübol, T., Kullakçi, H., İlhan, İ., Yilmaz, F.		NN			
Embase	Evidence of mitochondrial dysfunction in fibromyalgia: Deviating muscle energy metabolism detected using microdialysis and magnetic resonance	2020	Gerdle, B., Ghafouri, B., Lund, E., Bengtsson, A., Lundberg, P., van Ettinger-Veenstra, H., Leinhard, O.D., Forsgren, M.F.		NN			
Embase	Exploration of functional connectivity changes previously reported in fibromyalgia and their relation to psychological distress and pain measures	2020	van Ettinger-Veenstra, H., Boehme, R., Ghafouri, B., Olausson, H., Wicksell, R.K., Gerdle, B.	Y				
Embase	Explosive Synchronization of Brain Network Activity in Chronic Pain	2020			YY	Y	N	Study protocol only
Embase	Fibromyalgia syndrome—A laser-evoked potentials study unsupportive of small nerve fibre involvement	2020	Van Assche, D.C.F., Plaghki, L., Masquelier, E., Hatem, S.M.	Y				
Embase	Il-6 Cytokine Level: Relevance to Inflammation and Autonomic Induced Pain and Fatigue in Fibromyalgia and Me/CFS	2020	Amato, M.L., Themelis, K., Thompson, B., Stocks, R., Pound, A., Cipinova, Z.C., Shah-Goodwin, L., Timeyin, J., Barritt, A., Critchley, H.D., Davies, K.A., Harrison, N.A., Eccles, J.A.	Y				
Embase	MCP-1 is increased in patients with CFS and FM, whilst several other immune markers are significantly lower than healthy controls	2020	Groven, N., Fors, E.A., Stunes, A.K., Reitan, S.K.		NN			
Embase	Magnetic resonance imaging of neuroinflammation in chronic pain: A role for astrogliosis?	2020	Jung, C., Ichesco, E., Ratai, E.-M., Gonzalez, R.G., Burdo, T., Loggia, M.L., Harris, R.E., Napadow, V.	Y				
Embase	Modulation of Pain Sensitization in Complex Regional Pain Syndrome	2020			NN			
Embase	Mutual interaction between motor cortex activation and pain in fibromyalgia: EEG-fNIRS study	2020	Gentile, E., Brunetti, A., Ricci, K., Delussi, M., Bevilacqua, V., de Tommaso, M.	Y				

Embase	Neural correlates of conditioned pain responses in fibromyalgia subjects indicate preferential formation of new pain associations rather than extinction of irrelevant ones	2020	Sandström, A., Ellerbrock, I., Tour, J., Kadetoff, D., Jensen, K.B., Kosek, E.	Y	
Embase	Neural correlates of conditioned pain responses in fibromyalgia subjects indicate preferential formation of new pain associations rather than extinction of irrelevant ones	2020	Sandström, A., Ellerbrock, I., Tour, J., Kadetoff, D., Jensen, K.B., Kosek, E.	Y	
Embase	Neurochemical correlates of brain atrophy in fibromyalgia syndrome: A magnetic resonance spectroscopy and cortical thickness study	2020	Feraco, P., Nigro, S., Passamonti, L., Grecucci, A., Caligiuri, M.E., Gagliardo, C., Bacci, A.	Y	
Embase	Neuroinflammation in fibromyalgia assessed by proton magnetic resonance spectroscopy-A pilot study	2020	Kwiatek, R., Crouch, B., True, B., Whittle, S., Walls, A., Sherwood, V.		NN
Embase	Neuromagnetic Amygdala Response to Pain-Related Fear as a Brain Signature of Fibromyalgia	2020	Hsiao, F.-J., Chen, W.-T., Ko, Y.-C., Liu, H.-Y., Wang, Y.-F., Chen, S.-P., Lai, K.-L., Lin, H.-Y., Coppola, G., Wang, S.-J.	Y	
Embase	Pain catastrophizing is associated with the Val66Met polymorphism of the brain-derived neurotrophic factor in fibromyalgia	2020	Da Silveira Alves, C.F., Caumo, W., Silvestri, J.M., Zortea, M., Dos Santos, V.S., Cardoso, D.F., Regner, A., De Souza, A.H., Simon, D.	Y	
Embase	Patients with fibromyalgia show increased beta connectivity across distant networks and microstates alterations in resting-state electroencephalogram	2020	González-Villar, A.J., Triñanes, Y., Gómez-Perretta, C., Carrillo-de-la-Peña, M.T.	Y	
Embase	Peripheral immune aberrations in fibromyalgia: A systematic review, meta-analysis and meta-regression	2020	Andrés-Rodríguez, L., Borràs, X., Feliu-Soler, A., Pérez-Aranda, A., Angarita-Osorio, N., Moreno-Peral, P., Montero-Marin, J., García-Campayo, J., Carvalho, A.F., Maes, M., Luciano, J.V.		NN
Embase	Pressure-induced referred pain as a biomarker of pain sensitivity in fibromyalgia	2020	Arroyo-Fernández, R., Bravo-Esteban, E., Doménech-García, V., Ferri-Morales, A.		NN

Embase	Randomised Cross-over Study of the Effect of Transcutaneous Vagal Nerve Stimulation (tVNS) on Brain Activation at Rest and During Oesophageal Pain in Healthy Humans	2020			NN
Embase	Serum agrin and talin are increased in major depression while agrin and creatine phosphokinase are associated with chronic fatigue and fibromyalgia symptoms in depression	2020	Al-Hakeim, H.K., Al-Issa, A.A.R., Maes, M.		NN
Embase	Sifting the wheat from the chaff? Evidence for the existence of an asymmetric fibromyalgia phenotype	2020	H. Kaziyama, H., Barbour, J., Galhardoni, R., Aparecida da Silva, V., R. D. Tesseroli de Siqueira, S., Listik, C., dos Santos, G.J., Yeng, L.T., Marcolin, M.A., Raicher, I., Teixeira, M.J., Ciampi de Andrade, D.		NN
Embase	Sleep quality and clinical and psychological manifestations in women with mild systemic lupus erythematosus activity compared to women with fibromyalgia: A preliminary study	2020	Cervilla, O., Miró, E., Martínez, M.P., Sánchez, A.I., Sabio, J.M., Prados, G.		NN
Embase	Striatal hypofunction as a neural correlate of mood alterations in chronic pain patients	2020	Kim, M., Mawla, I., Albrecht, D.S., Admon, R., Torrado-Carvajal, A., Bergan, C., Protsenko, E., Kumar, P., Edwards, R.R., Saha, A., Napadow, V., Pizzagalli, D.A., Loggia, M.L.	Y	
Embase	Subtypes of sensory sensitivity in overactive bladder syndrome: Results of neuroimaging and sensory testing from the symptoms of lower urinary tract dysfunction research network (LURN)	2020	Mawla, I., Schrepf, A., Ichesco, E., Kutch, H.J., Lai, H.H., Helmuth, M.E., Andreev, V.P., Harris, R.E., Kirkali, Z., Harte, S.E.		NN
Embase	Subtypes of sensory sensitivity in overactive bladder syndrome: Results of neuroimaging and sensory testing from the	2020	Mawla, I., Schreph, A., Ichesco, E., Kutch, H.J., Lai, H.H., Helmuth, M.E., Andreev, V.P.,	Y	

	symptoms of lower urinary tract dysfunction research network (LURN)		Harris, R.E., Harte, S.E., Kirkali, Z.					
Embase	The Innate Central Nervous System Immune Response to an Experimental Immune Challenge in People With Fibromyalgia	2020			NN			
Embase	The Role of Dopamine in Fronto-striatal Activation in Emotional-motivational Pain Processing in Patients With Chronic Pain	2020			YN	Y	N	Study protocol only
Embase	The comparison of the biological rhythms of patients with fibromyalgia syndrome with biological rhythms of healthy controls	2020	Bulbul, F., Koca, I., Savas, E., Dokuyucu, R.	Y				
Embase	ACUPUNCTURE PROMOTES SENSORIMOTOR NETWORK NEUROPLASTICITY IN FIBROMYALGIA	2019	Mawla, I., Ichesco, E., Kaplan, C., Clauw, D., Napadow, V., Harris, R.		NN			
Embase	ALTERED PERIPHERAL AXONAL PROPERTIES IN FIBROMYALGIA	2019	Lin, C., Teng, H.-W., Tani, J., Chen, H.-J., Lin, Y.-C., Chang, T.-S., Sung, J.-Y.		NN			
Embase	Aberrant amygdala activation to pain-elicited fear processing in patients with fibromyalgia	2019	Hsiao, F.-J., Chen, W.-T., Wang, S.-J.		YN	N		
Embase	Acupuncture promotes sensorimotor network neuroplasticity in fibromyalgia	2019	Mawla, I., Ichesco, E., Kaplan, C., Clauw, D., Napadow, V., Harris, R.	Y				
Embase	Altered Cervical Spinal Cord Resting-State Activity in Fibromyalgia	2019	Martucci, K.T., Weber, K.A., Mackey, S.C.	Y				
Embase	Altered resting state functional connectivity of the cognitive control network in fibromyalgia and the modulation effect of mind-body intervention	2019	Kong, J., Wolcott, E., Wang, Z., Jorgenson, K., Harvey, W.F., Tao, J., Rones, R., Wang, C.	Y				
Embase	Apparent Effects of Opioid Use on Neural Responses to Reward in Chronic Pain	2019	Martucci, K.T., MacNiven, K.H., Borg, N., Knutson, B., Mackey, S.C.	Y				
Embase	BDNF and serum S100B levels according the spectrum of structural pathology in chronic pain patients	2019	Stefani, L.C., Leite, F.M., da Graça L. Tarragó, M., Zanette, S.A., de Souza, A., Castro, S.M., Caumo, W.	Y				

Embase	Brain Electrical Activity Associated with Visual Attention and Reactive Motor Inhibition in Patients with Fibromyalgia	2019	González-Villar, A.J., Arias, M., Carrillo-De-La-Peña, M.T.	Y	
Embase	Brain glial activation in fibromyalgia – A multi-site positron emission tomography investigation	2019	Albrecht, D.S., Forsberg, A., Sandström, A., Bergan, C., Kadetoff, D., Protsenko, E., Lampa, J., Lee, Y.C., Höglund, C.O., Catana, C., Cervenka, S., Akeju, O., Lekander, M., Cohen, G., Halldin, C., Taylor, N., Kim, M., Hooker, J.M., Edwards, R.R., Napadow, V., Kosek, E., Loggia, M.L.	Y	
Embase	Brain responses to other people's pain in fibromyalgia: a magnetoencephalography study	2019	Goldstein, A., Zeev-Wolf, M., Herz, N., Ablin, J.N.	Y	
Embase	Dietary intake does not explain microbiome alterations or symptom severity in fibromyalgia	2019	Minerbi, A., Chevalier, S., Anjarkouchian, A., Moyen, A., Shir, Y., Fitzcharles, M.-A.		NN
Embase	Differential neuroplastic changes in fibromyalgia and depression indexed by up-regulation of motor cortex inhibition and disinhibition of the descending pain system: An exploratory study	2019	Cardinal, T.M., Antunes, L.C., Brietzke, A.P., Parizotti, C.S., Carvalho, F., De Souza, A., da Silva Torres, I.L., Fregni, F., Caumo, W.	Y	
Embase	Does Occipital Nerve Stimulation Alter Pain Response as Measured by Quantitative Sensory Testing in Patients With Chronic Migraine or Cluster Headache	2019			NN
Embase	Exploratory Investigation of a Brief Cognitive Behavioral Intervention and Transcranial Direct Current Stimulation on Odor Sensitivity	2019	Houghton, D.C., Uhde, T.W., Borckardt, J.J., Cortese, B.M.		NN
Embase	Functional connectivity of music-induced analgesia in fibromyalgia	2019	Pando-Naude, V., Barrios, F.A., Alcauter, S., Pasaye, E.H., Vase, L., Brattico, E., Vuust, P., Garza-Villarreal, E.A.	Y	
Embase	Impact of Fibromyalgia on Alpha-2 EEG Power Spectrum in the Resting Condition: A Descriptive Correlational Study	2019	Villafaina, S., Collado-Mateo, D., Fuentes-García, J.P., Cano-Plasencia, R., Gusi, N.	Y	

Embase	Influence of pain anticipation on brain activity and pain perception in Gulf War Veterans with chronic musculoskeletal pain	2019	Lindheimer, J.B., Stegner, A.J., Ellingson-Sayen, L.D., Van Riper, S.M., Dougherty, R.J., Falvo, M.J., Cook, D.B.	Y	
Embase	Mechanisms of chronic pain and fatigue in fibromyalgia and ME/CFS: Contribution of autonomic arousal	2019	Winchester, R.L., Themelis, K., Cipinova, Z.C., Shah-Goodwin, L., Davies, K., Eccles, J.		NN
Embase	Modulatory effect of transcranial direct current stimulation (TDCS) on hand sensibility measured by objective quantitative analysis device	2019	Abdelrahman, H.I.Z., Ueda, A., Kurumadani, H., Sunagawa, T.		NN
Embase	Motor cortex function in fibromyalgia: A pilot study involving near-infrared spectroscopy and co-recording of laser-evoked potentials	2019	Gentile, E., Ricci, K., Delussi, M., De Tommaso, M.	Y	
Embase	Movement and pain in fibromyalgia: a co-registration by fNIRS and LEPs	2019	Gentile, E., Ricci, K., Montemurno, A., Delussi, M., Libro, G., de Tommaso, M.		NN
Embase	Neural Correlates of Knee Sensorimotor Control in Patients With Patellofemoral Pain Syndrome	2019			NN
Embase	Neural correlates of cognitive dysfunction in fibromyalgia patients: Reduced brain electrical activity during the execution of a cognitive control task	2019	Samartin-Veiga, N., González-Villar, A.J., Carrillo-de-la-Peña, M.T.	Y	
Embase	Neuroinflammation in fibromyalgia assessed by proton magnetic resonance spectroscopy - a pilot study	2019	Kwiatek, R., Crouch, B., True, B., Whittle, S., Wall, A., Sherwood, V.		NN
Embase	No evidence for altered plasma NGF and BDNF levels in fibromyalgia patients	2019	Baumeister, D., Eich, W., Saft, S., Geisel, O., Hellweg, R., Finn, A., Svensson, C.I., Tesarz, J.	Y	
Embase	P44-S Sensorimotor integration in fibromyalgia	2019	Vuralli, D., Ayyildiz, T., Bozdag, Y., Cengiz, B., Gunendi, Z.		NN
Embase	Pain expressions and inhibitory control in patients with fibromyalgia: Behavioral and neural correlates	2019	Pidal-Miranda, M., González-Villar, A.J., Carrillo-de-la-Peña, M.T.		NN

Embase	Patients with Fibromyalgia and Chronic Fatigue Syndrome show increased hsCRP compared to healthy controls	2019	Groven, N., Fors, E.A., Reitan, S.K.		NN
Embase	Potency of descending pain modulatory system is linked with peripheral sensory dysfunction in fibromyalgia	2019	Brietzke, A.P., Antunes, L.C., Carvalho, F., Elkifury, J., Gasparin, A., Sanches, P.R.S., da Silva, D.P., Dussán-Sarria, J.A., Souza, A., da Silva Torres, I.L., Fregni, F., Caumo, W.		NN
Embase	Quantitative sensitivity testing in paediatric patients with chronic pain: A systematic review and meta-analysis	2019	Schoth, D., Broadbent, P., Brown, H., Down, N., Gibbons, V., Ranger, O., Zhang, J.		NN
Embase	Relationship between mentalizing abilities, attachment styles, anxiety and depressive symptom levels in fibromyalgia patients	2019	Inanc, L., Bodur, N.E., Parlak, S.C., Sarikaya, M., Sari, A., Gulec, H., Citak, S.		NN
Embase	Subliminal emotional pictures are capable of modulating early cerebral responses to pain in fibromyalgia	2019	Peláez, I., Ferrera, D., Barjola, P., Fernandes, R., Mercado, F.	Y	
Embase	Subtle changes of gray matter volume in fibromyalgia reflect chronic musculoskeletal pain rather than disease-specific effects	2019	Sundermann, B., Dehghan Nayyeri, M., Pfleiderer, B., Stahlberg, K., Jünke, L., Baie, L., Dieckmann, R., Liem, D., Happe, T., Burgmer, M.	Y	
Embase	THE MITOCHONDRIAL TRANSLOCATOR PROTEIN SINGLE NUCLEOTIDE POLYMORPHISM RS6971 MAY INFLUENCE PAIN AND FATIGUE IN RHEUMATOID ARTHRITIS: RESULTS OF A PRELIMINARY STUDY	2019	Narayan, N., Mandhair, H., Sabokbar, A., Taylor, P.		NN
Embase	The mitochondrial translocator protein single nucleotide polymorphism RS6971 may influence pain and fatigue in rheumatoid arthritis: Results of a preliminary study	2019	Narayan, N., Mandhair, H., Sabokbar, A., Taylor, P.	Y	
Embase	Unaltered low nerve growth factor and high brain-derived neurotrophic factor levels in plasma from patients with	2019	Jablochkova, A., Bäckryd, E., Kosek, E., Mannerkorpi, K., Ernberg, M., Gerdle, B., Ghafouri, B.	Y	

	fibromyalgia after a 15-week progressive resistance exercise							
Embase	Visual cortical excitability in fibromyalgic and migraine patients: A study with sound induced flash illusion	2019	Damerino, G., Torrente, A., Aronica, R., Scardina, S., Ferlisi, S., Pilati, L., Di Marco, S., Aprile, M., Bolognini, N., Guggino, G., Brighina, F.		NN			
Embase	A retrospective 2D morphometric analysis of adult female chiari type I patients with commonly reported and related conditions	2018	Eppelheimer, M.S., Houston, J.R., Bapuraj, J.R., Labuda, R., Loth, D.M., Braun, A.M., Allen, N.J., Pahlavian, S.H., Biswas, D., Urbizu, A., Martin, B.A., Maher, C.O., Allen, P.A., Loth, F.		NN			
Embase	Alteration of resting-state networks in patients with fibromyalgia, complex regional pain syndrome, and other functional somatic pain syndrome	2018	Shibata, M., Watanabe, Y., Tanaka, H., Kan, S.		YY	Y	N	conference abstract only – no full text available.
Embase	Altered theta oscillations in resting EEG of fibromyalgia syndrome patients	2018	Fallon, N., Chiu, Y., Nurmikko, T., Stancak, A.	Y				
Embase	An investigational study on effect of burst stimulation on chronic pain in EEG, fMRI and PET	2018	Ahmed, S., Vanneste, S.		YN	Y	N	conference abstract only – no full text available.
Embase	Antibodies to post-translationally modified collagen II in spondyloarthritis	2018	Vinci, C.		NN			
Embase	Association between brain-derived neurotrophic factor gene polymorphisms and fibromyalgia in a Korean population: A multicenter study	2018	Park, D.-J., Kim, S.-H., Nah, S.-S., Lee, J.H., Kim, S.-K., Lee, Y.-A., Hong, S.-J., Kim, H.-S., Lee, H.-S., Kim, H.A., Joung, C.-I., Kim, S.-H., Lee, S.-S.	Y				
Embase	Association of Fine Motor Loss and Allodynia in Fibromyalgia: An fNIRS Study	2018	Eken, A., Gökçay, D., Yılmaz, C., Baskak, B., Baltacı, A., Kara, M.	Y				
Embase	Attenuation of offset analgesia is associated with suppression of descending pain modulatory and reward systems in patients with chronic pain	2018	Zhang, S., Li, T., Kobinata, H., Ikeda, E., Ota, T., Kurata, J.		NN			
Embase	Brain Mechanisms of Altered Sensory Perception and Self-Referential Processing in Juvenile Fibromyalgia	2018			YN	N		

Embase	Catastrophizing interferes with cognitive modulation of pain in women with fibromyalgia	2018	Ellingson, L.D., Stegner, A.J., Schwabacher, I.J., Lindheimer, J.B., Cook, D.B.	Y	
Embase	Cerebral blood flow volume using color duplex sonography in patients with fibromyalgia syndrome	2018	Kaya, A., Akgöl, G., Gülkesen, A., Poyraz, A.K., Yildirim, T., Atmaca, M.	Y	
Embase	Cerebral vasomotor reactivity in fibromyalgia patients and its relationship to central neuropathic pain	2018	Guler, S., Kurtoglu, H.		NN
Embase	Characterization of the DELPhI System in Assessing Brain's Functionality in Different Neurological Disorders-A Pilot Study	2018			NN
Embase	Cold Water Pressor Test Differentially Modulates Functional Network Connectivity in Fibromyalgia Patients Compared with Healthy Controls	2018	Jarrahi, B., Martucci, K.T., Nilakantan, A.S., Mackey, S.	Y	
Embase	Correlations between brain changes and pain management after cognitive and meditative therapies: A systematic review of neuroimaging studies	2018	Nascimento, S.S., Oliveira, L.R., DeSantana, J.M.		NN
Embase	Design and Validation of an FPGA-Based Configurable Transcranial Doppler Neurofeedback System for Chronic Pain Patients	2018	Rey, B., Rodríguez, A., Lloréns-Bufort, E., Tembl, J., Muñoz, M.Á., Montoya, P., Herrero-Bosch, V., Monzo, J.M.	Y	
Embase	Differential efficiency of transcutaneous electrical nerve stimulation in dominant versus nondominant hands in fibromyalgia: Placebo-controlled functional near-infrared spectroscopy study	2018	Eken, A., Kara, M., Baskak, B., Baltaci, A., Gökçay, D.	Y	
Embase	Disrupted Resting State Network of Fibromyalgia in Theta frequency	2018	Choe, M.K., Lim, M., Kim, J.S., Lee, D.S., Chung, C.K.	Y	
Embase	Dopaminergic and serotonergic mechanisms in the modulation of pain: In vivo studies in human brain	2018	Martikainen, I.K., Hagelberg, N., Jääskeläinen, S.K., Hietala, J., Pertovaara, A.		NN

Embase	Effect of distress on transient network dynamics and topological equilibrium in phantom sound perception	2018	Mohan, A., Alexandra, S.J., Johnson, C.V., De Ridder, D., Vanneste, S.	Y		
Embase	Functional connectivity alterations: Novel therapy and future implications in chronic pain management	2018	Thorp, S.L., Suchy, T., Vadivelu, N., Helander, E.M., Urman, R.D., Kaye, A.D.		YN	N
Embase	Home-Based Transcranial Direct Current Stimulation Device Development: An Updated Protocol Used at Home in Healthy Subjects and Fibromyalgia Patients	2018	Carvalho, F., Brietzke, A.P., Gasparin, A., Dos Santos, F.P., Vercelino, R., Ballester, R.F., Sanches, P.R.S., da Silva, D.P., Torres, I.L.S., Fregni, F., Caumo, W.	Y		
Embase	Hyperexcitability of the Central Nervous System in Children with Chronic Pain: A Systematic Review	2018	Pas, R., Ickmans, K., Van Oosterwijck, S., Van der Cruyssen, K., Foubert, A., Leysen, L., Nijs, J., Meeus, M.		NN	
Embase	Morphology of subcortical brain nuclei is associated with autonomic function in healthy humans	2018	Ruffle, J.K., Coen, S.J., Giampietro, V., Williams, S.C.R., Apkarian, A.V., Farmer, A.D., Aziz, Q.	Y		
Embase	Novel insights of effects of pregabalin on neural mechanisms of intracortical disinhibition in physiopathology of fibromyalgia: An explanatory, randomized, double-blind crossover study	2018	Deitos, A., Soldatelli, M.D., Dussán-Sarria, J.A., Souza, A., da Silva Torres, I.L., Fregni, F., Caumo, W.	Y		
Embase	Offset Analgesia as a Measure of Central Sensitization in Children With Chronic Pain Disorders	2018			NN	
Embase	Pain Modulation: From Conditioned Pain Modulation to Placebo and Nocebo Effects in Experimental and Clinical Pain	2018	Damien, J., Colloca, L., Bellei-Rodriguez, C.-É., Marchand, S.		NN	
Embase	Perception of induced dyspnea in fibromyalgia and chronic fatigue syndrome	2018	Van Den Houte, M., Bogaerts, K., Van Diest, I., De Bie, J., Persoons, P., Van Oudenhove, L., Van den Bergh, O.		NN	
Embase	Repetitive Transcranial Magnetic Stimulation of the Precentral-gyrus in the Relief of Fibromyalgia Pain: an International Multicenter Controlled Adaptative Trial	2018			YN	N

Embase	Resting Functional Connectivity of the Periaqueductal Gray Is Associated With Normal Inhibition and Pathological Facilitation in Conditioned Pain Modulation	2018	Harper, D.E., Ichesco, E., Schrepf, A., Hampson, J.P., Clauw, D.J., Schmidt-Wilcke, T., Harris, R.E., Harte, S.E.	Y		
Embase	The effect of depressive symptoms on cognition in patients with fibromyalgia	2018	Gelonch, O., Garolera, M., Valls, J., Castella, G., Varela, O., Rossello, L., Pifarre, J.		NN	
Embase	The role of long-term physical exercise on performance and brain activation during the Stroop colour word task in fibromyalgia patients	2018	Martinsen, S., Flodin, P., Berrebi, J., Löfgren, M., Bileviciute-Ljungar, I., Mannerkorpi, K., Ingvar, M., Fransson, P., Kosek, E.	Y		
Embase	Thyroid functioning and fatigue in functional somatic syndromes-The role of early life adversity	2018	Fischer, S., Strahler, J., Markert, C., Skoluda, N., Doerr, J.M., Nater, U.M.		NN	
Embase	Using advanced MRS to estimate brain metabolite level at anterior cingulate cortex in fibromyalgia	2018	Liou, J.-K.A., Hsu, T.-W., Chen, W.-T., Lin, C.-Y.E., Lirng, J.-F.		YN	N
Embase	A study of brain metabolism in fibromyalgia by positron emission tomography	2017	Usui, C., Soma, T., Hatta, K., Aratani, S., Fujita, H., Nishioka, K., Machida, Y., Kuroiwa, Y., Nakajima, T., Nishioka, K.	Y		
Embase	Altered cerebral blood flow velocity features in fibromyalgia patients in resting-state conditions	2017	Rodríguez, A., Tembl, J., Mesa-Gresa, P., Muñoz, M.Á., Montoya, P., Rey, B.	Y		
Embase	Antibodies to type II collagen: A novel tool for the spondyloarthritis diagnosis?	2017	Vinci, C., Infantino, M., Pozzilli, P., Grossi, V., Manfredi, M., Bandinelli, F., Li Gobbi, F., Damiani, A., Strollo, R., Benucci, M., Nissim, A.		NN	
Embase	Biomarkers for Musculoskeletal Pain Conditions: Use of Brain Imaging and Machine Learning	2017	Boissoneault, J., Sevel, L., Letzen, J., Robinson, M., Staud, R.		NN	
Embase	Brain signature and functional impact of centralized pain: A multidisciplinary approach to the study of chronic pelvic pain (MAPP) network study	2017	Kutch, J.J., Ichesco, E., Hampson, J.P., Labus, J.S., Farmer, M.A., Martucci, K.T., Ness, T.J., Deutsch, G., Vania Apkarian, A., MacKey, S.C., Klumpp, D.J., Schaeffer, A.J., Rodriguez, L.V., Kreder, K.J., Buchwald, D., Andriole, G.L., Henry Lai, H., Mullins, C., Kusek, J.W., Richard Landis, J.,	Y		

			Mayer, E.A., Quentin Clemens, J., Clauw, D.J., Harris, R.E.		
Embase	Classical Conditioning Differences Associated With Chronic Pain: A Systematic Review	2017	Harvie, D.S., Moseley, G.L., Hillier, S.L., Meulders, A.		NN
Embase	Cortical treatment of neuropathic with rTMS and tDCS: From anecdote to evidence	2017	Garcia-Larrea, L.		NN
Embase	Difference in Regional Brain Volume between Fibromyalgia Patients and Long-Term Meditators	2017	Fayed, N., García-Martí, G., Sanz-Requena, R., Marti-Bonmatí, L., Garcia-Campayo, J.	Y	
Embase	Dissociative experiences in patients with fibromyalgia are mediated by symptoms of autonomic dysfunction	2017	Eccles, J.A., Aslanyan, D., Harrison, N.A., Davies, K.A., Critchley, H.D.		NN
Embase	Electroencephalographic Evidence of Altered Top–Down Attentional Modulation in Fibromyalgia Patients During a Working Memory Task	2017	González-Villar, A.J., Pidal-Miranda, M., Arias, M., Rodríguez-Salgado, D., Carrillo-de-la-Peña, M.T.	Y	
Embase	Elevated levels of eotaxin-2 in serum of fibromyalgia patients	2017	Furer, V., Hazan, E., Mor, A., Segal, M., Katav, A., Aloush, V., Elkayam, O., George, J., Ablin, J.N.		NN
Embase	Elevations of ventricular lactate levels occur in both chronic fatigue syndrome and fibromyalgia	2017	Natelson, B.H., Vu, D., Coplan, J.D., Mao, X., Blate, M., Kang, G., Soto, E., Kapusuz, T., Shungu, D.C.	Y	
Embase	Epigenetics insights into chronic pain: DNA hypomethylation in fibromyalgia - A controlled pilot-study	2017	Ciampi De Andrade, D., Maschietto, M., Galhardoni, R., Gouveia, G., Chile, T., Victorino Krepischi, A.C., Dale, C.S., Brunoni, A.R., Parravano, D.C., Cueva Moscoso, A.S., Raicher, I., Kaziyama, H.H.S., Teixeira, M.J., Brentani, H.P.	Y	
Embase	Functional MRI of the Reserpine-Induced Putative Rat Model of Fibromyalgia Reveals Discriminatory Patterns of Functional Augmentation to Acute Nociceptive Stimuli	2017	Wells, J.A., Shibata, S., Fujikawa, A., Takahashi, M., Saga, T., Aoki, I.	Y	

Embase	Functional connectivity-based biomarkers for chronic musculoskeletal pain	2017	Edwards, R.		NN
Embase	Hemodynamic and EEG changes in patients with fibromyalgia	2017	Gentile, E., Venutelli, M.E., Ricci, K., Vecchio, E., Montemurno, A., Delussi, M., Balconi, M., De Tommaso, M.		NN
Embase	Histological underpinnings of grey matter changes in fibromyalgia investigated using multimodal brain imaging	2017	Pomares, F.B., Funck, T., Feier, N.A., Roy, S., Daigle-Martel, A., Ceko, M., Narayanan, S., Araujo, D., Thiel, A., Stikov, N., Fitzcharles, M.-A., Schweinhardt, P.	Y	
Embase	Investigating the BOLD spectral power of the intrinsic connectivity networks in fibromyalgia patients: A resting-state fMRI study	2017	Jarrahi, B., Martucci, K.T., Nilakantan, A.S., Mackey, S.	Y	
Embase	Is the HTR3A rs1062613 polymorphism differently associated with reward-related dopamine release in fibromyalgia patients and healthy controls?	2017	Ledermann, K., Jenewein, J., Sprott, H., Hasler, G., Schnyder, U., Warnock, G., Johayem, A., Kollias, S., Buck, A., Martin Soelch, C.		NN
Embase	Lower functional connectivity of the periaqueductal gray is related to negative affect and clinical manifestations of fibromyalgia	2017	Coulombe, M.-A., Lawrence, K.S., Moulin, D.E., Morley-Forster, P., Shokouhi, M., Nielson, W.R., Davis, K.D.	Y	
Embase	Measuring Glutamate Levels in the Brains of Fibromyalgia Patients and a Potential Role for Glutamate in the Pathophysiology of Fibromyalgia Symptoms	2017	Pyke, T.L., Osmotherly, P.G., Baines, S.		NN
Embase	Mood Disorders and Oxytocin	2017	Açikel, S.B.		NN
Embase	Mood Disorders and Oxytocin	2017	Açikel, S.B.	Y	
Embase	Multivariate pattern analysis utilizing structural or functional MRI—In individuals with musculoskeletal pain and healthy controls: A systematic review	2017	Smith, A., López-Solà, M., McMahon, K., Pedler, A., Sterling, M.		NN
Embase	Neuromodulation by electroacupuncture for migraine without aura analysis using diffusion tensor imaging	2017	Ishiyama, S., Shibata, Y., Ayuzawa, S., Matsushita, A., Matsumura, A.		NN
Embase	New insights in vivo into the treatment of migraine and other	2017	Da Silva, A.		NN

	chronic pain disorders using non-invasive neuromodulation							
Embase	Nondermatomal somatosensory deficits in chronic pain are associated with cerebral grey matter changes	2017	Riederer, F., Landmann, G., Gantenbein, A.R., Stockinger, L., Egloff, N., Sprott, H., Schleinzer, W., Pirrotta, R., Dumat, W., Luechinger, R., Baumgartner, C., Kollias, S., Sándor, P.S.		NN			
Embase	Normal placebo response in fibromyalgia patients despite reduced gray matter in DLPFC	2017	Ceko, M., Frangos, E., Richards, E., Wang, B., Schweinhardt, P., Bushnell, C.		YY	Y	N	conference abstract only – no full text available.
Embase	Occipital Nerve Field Transcranial Direct Current Stimulation Normalizes Imbalance Between Pain Detecting and Pain Inhibitory Pathways in Fibromyalgia	2017	De Ridder, D., Vanneste, S.	Y				
Embase	Pain catastrophizing and features of cortical response to painful stimuli in fibromyalgia	2017	Ricci, K., Vecchio, E., Delussi, M., Montemurno, A., Gentile, E., De Tommaso, M.		NN			
Embase	Painful After-Sensations in Fibromyalgia are Linked to Catastrophizing and Differences in Brain Response in the Medial Temporal Lobe	2017	Schreiber, K.L., Loggia, M.L., Kim, J., Cahalan, C.M., Napadow, V., Edwards, R.R.	Y				
Embase	Physio-somatic symptoms in schizophrenia: association with depression, anxiety, neurocognitive deficits and the tryptophan catabolite pathway	2017	Kanchanatawan, B., Sirivichayakul, S., Thika, S., Ruxrungtham, K., Carvalho, A.F., Geffard, M., Anderson, G., Noto, C., Ivanova, R., Maes, M.		NN			
Embase	RTMS of the prefrontal cortex has analgesic effects on neuropathic pain in subjects with spinal cord injury	2017	Nardone, R., Höller, Y., Langthaler, P.B., Lochner, P., Golaszewski, S., Schwenker, K., Brigo, F., Trinka, E.	Y				
Embase	Reduced laser-evoked potential habituation detects abnormal central pain processing in painful radiculopathy patients	2017	Hüllemann, P., von der Brelie, C., Manthey, G., Düsterhöft, J., Helmers, A.K., Synowitz, M., Baron, R.	Y				
Embase	Resting state electrical brain activity and connectivity in fibromyalgia	2017	Vanneste, S., Ost, J., Van Havenbergh, T., De Ridder, D.	Y				
Embase	Suggestions to Reduce Clinical Fibromyalgia Pain and Experimentally Induced Pain	2017	Derbyshire, S.W.G., Whalley, M.G., Seah, S.T.H., Oakley, D.A.	Y				

	Produce Parallel Effects on Perceived Pain but Divergent Functional MRI-Based Brain Activity				
Embase	Towards a neurophysiological signature for fibromyalgia	2017	López-Solà, M., Woo, C.-W., Pujol, J., Deus, J., Harrison, B.J., Monfort, J., Wager, T.D.	Y	
Embase	A neurometabolite study of chronic daily headache in patients with systemic lupus erythematosus using magnetic resonance spectroscopy: Comparison with fibromyalgia patients and healthy controls	2016	Son, C.-N., Kim, S.-H., Chang, H.W., Kim, J.-M.	Y	
Embase	A possible neural mechanism for photosensitivity in chronic pain	2016	Martenson, M.E., Halawa, O.I., Tonsfeldt, K.J., Maxwell, C.A., Hammack, N., Mist, S.D., Pennesi, M.E., Bennett, R.M., Mauer, K.M., Jones, K.D., Heinriche, M.M.	Y	
Embase	Abnormal resting state functional connectivity of the periaqueductal grey in patients with fibromyalgia	2016	Truini, A., Tinelli, E., Gerardi, M.C., Calistri, V., Iannuccelli, C., La Cesa, S., Tarsitani, L., Mainero, C., Sarzi-Puttini, P., Cruccu, G., Caramia, F., Di Franco, M.	Y	
Embase	Altered fMRI resting-state connectivity in individuals with fibromyalgia on acute pain stimulation	2016	Ichesco, E., Puiu, T., Hampson, J.P., Kairys, A.E., Clauw, D.J., Harte, S.E., Peltier, S.J., Harris, R.E., Schmidt-Wilcke, T.	Y	
Embase	Augmented Pain Processing in Primary and Secondary Somatosensory Cortex in Fibromyalgia: A Magnetoencephalography Study Using Intra-Epidermal Electrical Stimulation	2016	Lim, M., Roosink, M., Kim, J.S., Kim, H.W., Lee, E.B., Son, K.M., Kim, H.A., Chung, C.K.	Y	
Embase	Blink reflex and prepulse inhibition in fibromyalgia	2016	Kofler, M.		NN
Embase	Brain responses to other's pain in fibromyalgia-a magnetoencephalography (MEG) study	2016	Goldstein, A., Wolf, M., Ablin, J.N.		NN

Embase	Cerebral vasomotor reactivity in fibromyalgia patients and its relationship to central neuropathic pain	2016	Guler, S., Kurtoğlu, H.S., Kehaya, S., Pamuk, N., Çelik, Y.	Y	
Embase	Characterizing "fibrofog": Subjective appraisal, objective performance, and task-related brain activity during a working memory task	2016	Walitt, B., Čeko, M., Khatiwada, M., Gracely, J.L., Rayhan, R., Vanmeter, J.W., Gracely, R.H.	Y	
Embase	Controllability and hippocampal activation during pain expectation in fibromyalgia syndrome	2016	González-Roldán, A.M., Bomba, I.C., Diesch, E., Montoya, P., Flor, H., Kamping, S.	Y	
Embase	Discordant dry eye disease (An American ophthalmological society thesis)	2016	Shtein, R.M., Harper, D.E., Pallazola, V., Harte, S.E., Hussain, M., Sugar, A., Williams, D.A., Clauw, D.J.	Y	
Embase	Endogenous opioidergic dysregulation of pain in fibromyalgia: A PET and fMRI study	2016	Schrepf, A., Harper, D.E., Harte, S.E., Wang, H., Ichesco, E., Hampson, J.P., Zubieta, J.-K., Clauw, D.J., Harris, R.E.	Y	
Embase	Endogenous opioidergic dysregulation of pain in fibromyalgia: A PET and fMRI study	2016	Schrepf, A., Harper, D.E., Harte, S.E., Wang, H., Ichesco, E., Hampson, J.P., Zubieta, J.-K., Clauw, D.J., Harris, R.E.	Y	
Embase	Evaluation of cytokines, oxidative stress markers and brain-derived neurotrophic factor in patients with fibromyalgia - A controlled cross-sectional study	2016	Ranzolin, A., Duarte, A.L.B.P., Bredemeier, M., da Costa Neto, C.A., Ascoli, B.M., Wollenhaupt-Aguiar, B., Kapczinski, F., Xavier, R.M.	Y	
Embase	Executive function in fibromyalgia: Comparing subjective and objective measures	2016	Gelonch, O., Garolera, M., Valls, J., Rosselló, L., Pifarré, J.		NN
Embase	Experimental hypervigilance changes the intensity/unpleasantness ratio of pressure sensations: evidence for the generalized hypervigilance hypothesis	2016	Hollins, M., Walters, S.		NN
Embase	Fibromyalgia is correlated with retinal nerve fiber layer thinning	2016	Garcia-Martin, E., Garcia-Campayo, J., Puebla-Guedea, M., Ascaso, F.J., Roca, M., Gutierrez-Ruiz, F., Vilades, E.,	Y	

			Polo, V., Larrosa, J.M., Pablo, L.E., Satue, M.						
Embase	Functional connectivity with the default mode network is altered in fibromyalgia patients	2016	Fallon, N., Chiu, Y., Nurmikko, T., Stancak, A.	Y					
Embase	Gray Matter Atrophy within the Default Mode Network of Fibromyalgia: A Meta-Analysis of Voxel-Based Morphometry Studies	2016	Lin, C., Lee, S.-H., Weng, H.-H.		NN				
Embase	Gray matter abnormalities associated with fibromyalgia: A meta-analysis of voxel-based morphometric studies	2016	Shi, H., Yuan, C., Dai, Z., Ma, H., Sheng, L.		NN				
Embase	Habituation deficit of auditory N100m in patients with fibromyalgia	2016	Choi, W., Lim, M., Kim, J.S., Chung, C.K.	Y					
Embase	Identifying a brain marker for fibromyalgia using brain responses to pain and multisensory stimulation	2016	Sola, M.L., Woo, C.W., Pujol, J., Deus, J.		YY	Y	N		conference abstract only – no full text available.
Embase	Increased low-and high-frequency oscillatory activity in the prefrontal cortex of fibromyalgia patients	2016	Lim, M., Kim, J.S., Kim, D.J., Chung, C.K.	Y					
Embase	Is Motor Cortical Excitability Altered in People with Chronic Pain? A Systematic Review and Meta-Analysis	2016	Parker, R.S., Lewis, G.N., Rice, D.A., Mcnair, P.J.		NN				
Embase	MRI based classification of chronic fatigue, fibromyalgia patients and healthy controls using machine learning algorithms: A comparison study	2016	Sevel, L., Letzen, J., Boissoneault, J., O'Shea, A., Robinson, M., Staud, R.		YY	Y	N		conference abstract only – no full text available.
Embase	Motor cortex excitability and BDNF levels in chronic musculoskeletal pain according to structural pathology	2016	Caumo, W., Deitos, A., Carvalho, S., Leite, J., Carvalho, F., Dussán-Sarria, J.A., Tarragó, M.G.L., Souza, A., Torres, I.L.S., Fregni, F.	Y					
Embase	Pharmacologic attenuation of cross-modal sensory augmentation within the chronic pain insula	2016	Harte, S.E., Ichesco, E., Hampson, J.P., Peltier, S.J., Schmidt-Wilcke, T., Clauw, D.J., Harris, R.E.	Y					

Embase	Physical Approaches to the Management of Fibromyalgia - A Multidisciplinary Approach	2016			NN			
Embase	Quantitative and Qualitative EEG From Fibromyalgia Patients for the Identification of Abnormal Patterns on Closed Eyes EEG	2016			NN			
Embase	Reduced brain somatosensory network connectivity in cyclic vomiting syndrome and episodic migraine is region-specific	2016	Ellingsen, D.-M., Garcia, R., Lin, R., Lee, J., Kim, J., Kim, H., Thurler, A.H., Dimisko, L., Loggia, M., Hadjikhani, N., Kuo, B., Napadow, V.		NN			
Embase	Relation of dopamine receptor 2 binding to pain perception in female fibromyalgia patients with and without depression - A [11C] raclopride PET-study	2016	Ledermann, K., Jenewein, J., Sprott, H., Hasler, G., Schnyder, U., Warnock, G., Johayem, A., Kollias, S., Buck, A., Martin-Soelch, C.	Y				
Embase	Tai Chi significantly modulates resting state functional connectivity of the cognitive control network in fibromyalgia	2016	Kong, J., Jorgenson, K., Wang, Z., Wolcott, E., Harvey, W.F., Wang, C.		YY	Y	N	conference abstract only – no full text available.
Embase	Touch perception altered by chronic pain and by opioid blockade	2016	Case, L.K., Čeko, M., Gracely, J.L., Richards, E.A., Olausson, H., Bushnell, M.C.		NN			
Embase	Trigeminal nerve reflexes in chronic pain syndromes	2016	Reshkova, V., Rashkov, R., Milanov, I., Bogdanova, D.		NN			
Embase	Use of Repetitive Transcranial Magnetic Stimulation to Augment Hypnotic Analgesia	2016			NN			
Embase	A Pilot fMRI Study of Fibromyalgia: Tai Chi Intervention	2015			YY	Y	N	Study protocol only
Embase	A common neurobiology for pain, depression and reward in women with fibromyalgia?	2015	Ledermann, K., Jenewein, J., Sprott, H., Hasler, G., Schnyder, U., Warnock, G., Johayem, A., Kollias, S., Martin-Soelch, C.		NN			
Embase	Aberrant cerebral blood flow responses during cognition: Implications for the understanding of cognitive deficits in fibromyalgia	2015	Montoro, C.I., Duschek, S., De Guevara, C.M.L., Fernández-Serrano, M.J., Reyes del Paso, G.A.	Y				
Embase	Affective Modulation of Brain and Autonomic Responses in Patients with Fibromyalgia	2015	Rosselló, F., Muñoz, M.A., Duschek, S., Montoya, P.	Y				

Embase	Altered Cortical Processing of Observed Pain in Patients with Fibromyalgia Syndrome	2015	Fallon, N., Li, X., Chiu, Y., Nurmikko, T., Stancak, A.	Y				
Embase	Angiotensin-converting enzyme and methylenetetrahydrofolate reductase gene variations in fibromyalgia syndrome	2015	Inanir, A., Inanir, S., Yigit, S., Tekcan, A., Pinarli, F.A., Karakus, N.		NN			
Embase	Attentional bias towards subjectively unconscious threat-related information in fibromyalgia	2015	Mercado, F., Barjola, P., Peláez, I., Cardoso, S., Guerra, V., Gómez-Esquer, F.		NN			
Embase	Chronic daily headache in Korean patients with systemic lupus erythematosus	2015	Son, C., Kim, J., Kim, S.		NN			
Embase	Comparison of machine classification algorithms for fibromyalgia: Neuroimages versus self-report	2015	Robinson, M.E., O'Shea, A.M., Craggs, J.G., Price, D.D., Letzen, J.E., Staud, R.	Y				
Embase	Comparison of postural control in participants with chronic fatigue syndrome and fibromyalgia syndrome	2015	Rasouli, O., Fors, E.A., Loras, H., Vasseljen, O., Stensdotter, A.-K.		NN			
Embase	Default mode network connectivity in chronic fatigue syndrome patients	2015	Gay, C., O'Shea, A., Robinson, M., Craggs, J., Staud, R.		YN	N		
Embase	Disinhibition of the primary somatosensory cortex in patients with fibromyalgia	2015	Lim, M., Roosink, M., Kim, J.S., Kim, D.J., Kim, H.W., Lee, E.B., Kim, H.A., Chung, C.K.	Y				
Embase	Effect of Milnacipran Treatment on Ventricular Lactate in Fibromyalgia: A Randomized, Double-Blind, Placebo-Controlled Trial	2015	Natelson, B.H., Vu, D., Mao, X., Weiduschat, N., Togo, F., Lange, G., Blate, M., Kang, G., Coplan, J.D., Shungu, D.C.	Y				
Embase	Effect of cognitive-behavioral therapy on brain connectivity supporting hyperalgesia in fibromyalgia patients	2015	Lazaridou, A., Kim, J., Loggia, M., Cahalan, C., Harris, R., Beissner, F., Garcia, R., Kim, H., Wasan, A., Napadow, V., Edwards, R.		YY	Y	N	conference abstract only – no full text available.
Embase	Fibromyalgia is characterized by altered frontal and cerebellar structural covariance brain networks	2015	Kim, H., Kim, J., Loggia, M.L., Cahalan, C., Garcia, R.G., Vangel, M.G., Wasan, A.D., Edwards, R.R., Napadow, V.	Y				
Embase	Fibromyalgia patients have reduced hippocampal volume compared with healthy controls	2015	McCrae, C.S., O’Shea, A.M., Boissoneault, J., Vatthauer, K.E., Robinson, M.E., Staud,	Y				

			R., Perlstein, W.M., Craggs, J.G.			
Embase	Fibromyalgia update	2015	Hernandez Islas, A.		NN	
Embase	Gray matter abnormalities in chronic fatigue syndrome	2015	O'Shea, A., Gay, C., Robinson, M., Craggs, J., Staud, R.		NN	
Embase	Impaired pre-attentive auditory processing in fibromyalgia: A mismatch negativity (MMN) study	2015	Choi, W., Lim, M., Kim, J.S., Kim, D.J., Chung, C.K.	Y		
Embase	Increased Brain Gray Matter in the Primary Somatosensory Cortex is Associated with Increased Pain and Mood Disturbance in Patients with Interstitial Cystitis/Painful Bladder Syndrome	2015	Kairys, A.E., Schmidt-Wilcke, T., Puiu, T., Ichesco, E., Labus, J.S., Martucci, K., Farmer, M.A., Ness, T.J., Deutsch, G., Mayer, E.A., Mackey, S., Apkarian, A.V., Maravilla, K., Clauw, D.J., Harris, R.E.		NN	
Embase	Increased cortical activation upon painful stimulation in fibromyalgia syndrome	2015	Üçeyler, N., Zeller, J., Kewenig, S., Kittel-Schneider, S., Fallgatter, A.J., Sommer, C.	Y		
Embase	Integration of Neuroimaging and Transcranial Magnetic Stimulation: an Exploration of the Neuroplasticity of Endogenous Analgesia in Health and Chronic Pain	2015			YN	N
Embase	MAPP Research Network: Trans-MAPP Study of Urologic Chronic Pelvic Pain: Symptom Patterns Study (SPS)	2015			NN	
Embase	Men and women with fibromyalgia: Relation between attentional function and clinical symptoms	2015	Miró, E., Martínez, M.P., Sánchez, A.I., Prados, G., Lupiáñez, J.		NN	
Embase	Multimodal Characterization of the Prosocial and Prosexual Effects of GHB Assessing Behavior, fMRI, EEG, and Neuroendocrine Mechanisms	2015			NN	
Embase	Neurocognitive functions and brain atrophy after proven neuroborreliosis: A case-control study	2015	Schmidt, H., Djukic, M., Jung, K., Holzgraefe, M., Dechent, P., Steinbüchel, N., Blocher, J., Eiffert, H., Schmidt-Samoa, C.		NN	
Embase	Normalization of aberrant resting state functional connectivity in fibromyalgia patients following a	2015	Flodin, P., Martinsen, S., Mannerkorpi, K., Löfgren, M., Bileviciute-Ljungar, I., Kosek, E., Fransson, P.	Y		

	three month physical exercise therapy				
Embase	Opioid and dopamine transmission in chronic pain and health are diametrically opposed: A phMRI study	2015	Bourke, J., Lyons, G., Wodehouse, T., Constantinou, E., Knopp, P., Antoniades, M., Howard, J., White, P., Rabiner, E., Wall, M.		NN
Embase	Pregabalin Acute Effects on Cortical Excitability, Psychophysical Parameters, and Serum Markers of Neuroplastic Processes in Fibromyalgia: a Placebo Controlled, Double Blinded, Randomized, Crossover Clinical Trial	2015			NN
Embase	Reaction time, cerebral blood flow, and heart rate responses in fibromyalgia: Evidence of alterations in attentional control	2015	Reyes Del Paso, G.A., Montoro, C.I., Duschek, S.	Y	
Embase	Self-perspective leads to increased activation of pain processing brain regions in fibromyalgia	2015	Rahm, B., Lacour, M., Decety, J., Müller, J., Scheidt, C.-E., Bauer, J., König, R., Wirsching, M., Glauche, V., Ohlendorf, S., Unterbrink, T., Hartmann, A., Joos, A.A.	Y	
Embase	Self-perspective leads to increased activation of pain processing brain regions in fibromyalgia	2015	Rahm, B., Lacour, M., Decety, J., Müller, J., Scheidt, C.-E., Bauer, J., König, R., Wirsching, M., Glauche, V., Ohlendorf, S., Unterbrink, T., Hartmann, A., Joos, A.A.	Y	
Embase	Significance of measuring alpha-galactosidase a in fibromyalgia patients: Possibility of Fabry disease in fibromyalgia patients	2015	Hasunuma, T., Araki, N., Nakamura, K., Shikano, K., Momosaki, K., Kawai, S., Endo, F., Takada, F.		NN
Embase	Sleep disturbances in fibromyalgia syndrome: The role of clinical and polysomnographic variables explaining poor sleep quality in patients	2015	Diaz-Piedra, C., Catena, A., Sánchez, A.I., Miró, E., Pilar Martínez, M., Buela-Casal, G.		NN
Embase	Sympathetic activity in patients with fibromyalgia-A microneurographic study	2015	Lautenschläger, G., Thieme, K., Malinowski, R., Meller, T., Kaps, M., Krämer, H.		NN

Embase	The N170 and face perception in psychiatric and neurological disorders: A systematic review	2015	Feuerriegel, D., Churches, O., Hofmann, J., Keage, H.A.D.		NN			
Embase	The somatosensory link in fibromyalgia: Functional connectivity of the primary somatosensory cortex is altered by sustained pain and is associated with clinical/autonomic dysfunction	2015	Kim, J., Loggia, M.L., Cahalan, C.M., Harris, R.E., Beissner, F., Garcia, R.G., Kim, H., Barbieri, R., Wasan, A.D., Edwards, R.R., Napadow, V.	Y				
Embase	Vitamin D deficiency in depressive, anxiety and adjustment disorder	2015	Aydin, E.P., Varkal, M.D., Toker, O.G., Ozer, O.A., Karamustafalioglu, O.K.		NN			
Embase	Alterations in excitatory and inhibitory brainstem interneuronal circuits in fibromyalgia: Evidence of brainstem dysfunction	2014	Kofler, M., Halder, W.	Y				
Embase	Altered resting state connectivity of the insular cortex in individuals with fibromyalgia	2014	Ichesco, E., Schmidt-Wilcke, T., Bhavsar, R., Clauw, D.J., Peltier, S.J., Kim, J., Napadow, V., Hampson, J.P., Kairys, A.E., Williams, D.A., Harris, R.E.	Y				
Embase	Altered white matter integrity in corpus callosal area in fibromyalgia identified with tract based spatial statistical analysis	2014	Kim, H.A., Kim, D., Son, K.M., Chung, C.-K.	Y				
Embase	An investigation of emotional processing abilities in fibromyalgia syndrome	2014	Di Tella, M., Tesio, V., Leombruni, P., Colonna, F., Bruzzone, M., Scarati, M., Fusaro, E., Torta, R., Castelli, L.		NN			
Embase	Bilateral hippocampal volume reductions in fibromyalgia	2014	O'Shea, A., McCrae, C., Vatthauer, K., Robinson, M., Staud, R., Perlstein, W., Craggs, J.		YY	Y	N	conference abstract only – no full text available.
Embase	Brain Rhythms in Fibromyalgia: A Magnetoencephalography (MEG) Study	2014			YN	N		
Embase	Changes in clinical pain in fibromyalgia patients correlate with changes in brain activation in the cingulate cortex in a response inhibition task	2014	Schmidt-Wilcke, T., Kairys, A., Ichesco, E., Fernandez-Sanchez, M.L., Barjola, P., Heitzeg, M., Harris, R.E., Clauw, D.J., Glass, J., Williams, D.A.	Y				

Embase	Classification of brain activity response to painful heat stimuli in fibromyalgia patients and healthy controls	2014	Borja, M., Martucci, K., Nilakantan, A., Mackey, S.		YY	Y	N	conference abstract only – no full text available.
Embase	Disrupted brain circuitry for pain-related reward/punishment in fibromyalgia	2014	Loggia, M.L., Berna, C., Kim, J., Cahalan, C.M., Gollub, R.L., Wasan, A.D., Harris, R.E., Edwards, R.R., Napadow, V.	Y				
Embase	Electrophysiological evaluation of autonomic nervous system and cutaneous silent period in patients with fibromyalgia syndrome	2014	Ustun, I., Yagci, I., Akyuz, G., Unlu Ozkan, F.		NN			
Embase	FMRI pain activation in the periaqueductal gray in healthy volunteers during the cold pressor test	2014	La Cesa, S., Tinelli, E., Toschi, N., Di Stefano, G., Collorone, S., Aceti, A., Francia, A., Cruccu, G., Truini, A., Caramia, F.		YN	N		
Embase	Fibromyalgia patients had normal distraction related pain inhibition but cognitive impairment reflected in caudate nucleus and hippocampus during the Stroop color word test	2014	Martinsen, S., Flodin, P., Berrebi, J., Löfgren, M., Bileviciute-Ljungar, I., Ingvar, M., Fransson, P., Kosek, E.	Y				
Embase	Higher glutamate + glutamine and reduction of N-acetylaspartate in posterior cingulate according to age range in patients with cognitive impairment and/or pain	2014	Fayed, N., Andrés, E., Viguera, L., Modrego, P.J., Garcia-Campayo, J.	Y				
Embase	Impaired pre-attentive auditory processing in fibromyalgia: A mismatch negativity study	2014	Kim, H.A., Choi, W., Chung, C.-K.		NN			
Embase	Joint hypermobility and autonomic hyperactivity: Relevance to the expression of psychiatric symptoms	2014	Eccles, J.A., Iodice, V., Dowell, N.G., Owens, A., Hughes, L., Skipper, S., Humphryes, K., Lycette, Y., Harrison, N.A., Mathias, C.J., Critchley, H.D.		NN			
Embase	Management of fibromyalgia	2014	Mckenna, F.		NN			
Embase	Neuroimaging Approaches to Deconstructing Acupuncture for Chronic Pain	2014			YN	N		
Embase	Pain Sensitization and the Risk of Poor Outcome Following Physiotherapy for Knee	2014			NN			

	Osteoarthritis: A Protocol for a Prospective Cohort Study				
Embase	Regional gray matter density differences predict classification of chronic pelvic pain and fibromyalgia: Findings from the MAPP Research Network	2014	Martucci, K., Bagarinao, E., Johnson, K., Ichesco, E., Farmer, M., Labus, J., Ness, T., Harris, R., Deutsch, G., Apkarian, A., Mayer, E., Clauw, D., Mackey, S.		NN
Embase	Repeated transcranial magnetic stimulation relieves pain in fibromyalgia patients: An electrophysiological approach to evaluate pain	2014	Ansari, A.H., Mathur, R., Jain, S., Mukherjee, K., Mukherjee, K.		NN
Embase	Repetitive transcranial magnetic stimulation of the left premotor/dorsolateral prefrontal cortex does not have analgesic effect on central poststroke pain	2014	De Oliveira, R.A.A., De Andrade, D.C., Mendonça, M., Barros, R., Luvisoto, T., Myczkowski, M.L., Marcolin, M.A., Teixeira, M.J.	Y	
Embase	Small fiber neuropathy in women with fibromyalgia. A clinical-pathological correlation using confocal corneal biomicroscopy	2014	Ramírez-Fernández, M., Martinez-Martinez, L.-A., Vargas-Guerrero, A., Martínez-Lavín, M., Hernandez Quintela, E., Velazco-Caspia, J.		NN
Embase	The rubber hand illusion increases heat pain threshold	2014	Hegedüs, G., Darnai, G., Szolcsányi, T., Feldmann, A., Janszky, J., Kállai, J.		NN
Embase	Altered white matter integrity in the corpus callosum in fibromyalgia patients identified by tract-based spatial statistical analysis	2014	Kim, D.J., Lim, M., Kim, J.S., Son, K.M., Kim, H.A., Chung, C.K.	Y	
Embase	Fibromyalgia is associated with decreased connectivity between pain- and sensorimotor brain areas	2014	Flodin, P., Martinsen, S., Löfgren, M., Bileviciute-Ljungar, I., Kosek, E., Fransson, P.	Y	
Embase	The MAPP research network: Design, patient characterization and operations	2014	Landis, J.R., Williams, D.A., Lucia, M.S., Clauw, D.J., Naliboff, B.D., Robinson, N.A., Van Bokhoven, A., Sutcliffe, S., Schaeffer, A.J., Rodriguez, L.V., Mayer, E.A., Lai, H.H., Krieger, J.N., Kreder, K.J., Afari, N., Andriole, G.L., Bradley, C.S., Griffith, J.W., Klumpp, D.J., Hong, B.A.,	Y	

			Lutgendorf, S.K., Buchwald, D., Yang, C.C., Mackey, S., Pontari, M.A., Hanno, P., Kusek, J.W., Mullins, C., Clemens, J.Q., Kirkali, Z., Farmer, M.A., Fitzgerald, C., Cella, D., Heckman, C.J., Jiang, M., Keefer, L., Marko, D.S., Michniewicz, J., Parrish, T., Tu, F., Alger, J., Ashe-McNalley, C.P., Ellingson, B., Heendeniya, N., Kilpatrick, L., Kutch, J., Labus, J.S., Randal, F., Smith, S.R., Eno, M., Greiner, K., Luo, Y., O'Donnell, M.A., Ziegler, B., As-Sanie, S., Berry, S., Halvorson, M.E., Harris, R., Harte, S., Ichesco, E., Oldendorf, A., Scott, K.A., Krieger, J., Miller, J., Richey, S., Ross, S.O., Spiro, R., Sundsvold, T.J., Strachan, E., Bristol, R.L., Colditz, G., Deutsch, G., Gardner, V... ### LONG-TEXT-TRUNCATED-BY-CATCHII ###			
Embase	The impact of the stimulation method on differences in pain thresholds and brain responses between chronic pain patients and healthy controls	2014	Diers, M.		NN	
Embase	A common neurobiology for reward, chronic pain and depression? A [11C]raclopride bolus plus constant infusion PET-study	2013	Ledermann, K., Jenewein, J., Sprott, H., Hasler, G., Schnyder, U., Burger, C., Johayem, A., Kollias, S., Buck, A., Martin-Soelch, C.		YN	N
Embase	Alterations in endogenous opioid functional measures in chronic back pain	2013	Martikainen, I.K., Peciña, M., Love, T.M., Nuechterlein, E.B., Cummiford, C.M., Green, C.R., Harris, R.E., Stohler, C.S., Zubieta, J.-K.	Y		
Embase	Altered intrinsic brain connectivity in the salience network of fibromyalgia patients at rest	2013	Kim, S., Lee, S.	Y		

Embase	Altered intrinsic brain connectivity in the salience network of fibromyalgia patients at rest	2013	Kim, S.-H.		YY	Y	N	conference abstract only – no full text available.
Embase	Augmented central pain processing in vulvodynia	2013	Hampson, J.P., Reed, B.D., Clauw, D.J., Bhavsar, R., Gracely, R.H., Haefner, H.K., Harris, R.E.	Y				
Embase	Bilateral deficits in fine motor control ability and manual dexterity in women with fibromyalgia syndrome	2013	Pérez-De-Heredia-Torres, M., Martínez-Piédrola, R.M., Cigarán-Méndez, M., Ortega-Santiago, R., Fernández-De-Las-Peñas, C.		NN			
Embase	Brain correlates of cognitive inhibition in fibromyalgia: Emotional intrusion of symptom-related words	2013	Mercado, F., González, J.L., Barjola, P., Fernández-Sánchez, M., López-López, A., Alonso, M., Gómez-Esquer, F.	Y				
Embase	Brain processing of pain and expectancy of relief in fibromyalgia is modulated by catastrophizing	2013	Loggia, M., Berna Renella, C., Kim, J., Cahalan, C., Gollub, R., Wasan, A., Edwards, R., Napadow, V.		YN	Y	N	conference abstract only – no full text available.
Embase	Catastrophizing mediates pressure pain induced change in brain functional connectivity in fibromyalgia	2013	Kim, J., Loggia, M., Cahalan, C., Wasan, A., Edwards, R., Napadow, V.		YY	Y	N	conference abstract only – no full text available.
Embase	Chronic fatigue syndrome and fibromyalgia	2013	McKenna, F.		NN			
Embase	Classical conditioning and chronic pain: A systematic review	2013	Harvie, D.S., Hillier, S.L., Meulders, A., Moseley, G.L.		NN			
Embase	Comorbid diagnosis of insomnia and chronic pain associated with diffuse cortical thinning	2013	O'Shea, A., Craggs, J., Robinson, M.E., Staud, R., Berry, R.B., Price, D.D., Perlstein, W.M., McCrae, C.		NN			
Embase	Cytokines, oxidative stress markers and brain-derived neurotrophic factor in fibromyalgia syndrome	2013	Ranzolin, A., Neto, C.A.D.C., Ascoli, B.M., Wollenhaupt-Aguiar, B., Pinto Duarte, A.L.B., Bredemeier, M., Kapczinski, F., Xavier, R.M.		NN			
Embase	Decreased muscle concentrations of ATP and PCR in the quadriceps muscle of fibromyalgia patients - A 31P-MRS study	2013	Gerdle, B., Forsgren, M.F., Bengtsson, A., Dahlqvist Leinhard, O., Sören, B., Karlsson, A., Brandejsky, V., Lund, E., Lundberg, P.	Y				
Embase	Deficient modulation of pain by a positive emotional context in fibromyalgia patients	2013	Kamping, S., Bomba, I.C., Kanske, P., Diesch, E., Flor, H.	Y				

Embase	Diffuse tensor imaging-based brain signatures accurately discriminate a functional pain from health: Examining central mechanisms in visceral pain	2013	Labus, J., Van Horn, J.D., Torgerson, C., Ashe-McNalley, C., Irimia, A., Chambers, M.C., Gupta, A., Tillisch, K., Mayer, E.A.		NN			
Embase	Do patients with fibromyalgia show abnormal neural responses to the observation of pain in others?	2013	Lee, S.J., Song, H.-J., Decety, J., Seo, J., Kim, S.-H., Kim, S.-H., Nam, E.J., Kim, S.-K., Han, S.W., Lee, H.J., Do, Y., Chang, Y.	Y				
Embase	Epigenetic alterations and an increased frequency of micronuclei in women with fibromyalgia	2013	Lyon, D., Menzies, V., Archer, K., Brumelle, J.R., Jones, K.H., Gao, G., Elswick Jr., R.K., York, T., Jackson-Cook, C.		NN			
Embase	Evaluation of the Effectiveness of Pregabalin in Alleviating Pain Associated with Fibromyalgia: Using Functional Magnetic Resonance Imaging Study	2013	Kim, S.-H., Lee, Y., Lee, S., Mun, C.-W.	Y				
Embase	Evidence for working memory deficits in chronic pain: A systematic review and meta-analysis	2013	Berryman, C., Stanton, T.R., Jane Bowering, K., Tabor, A., McFarlane, A., Lorimer Moseley, G.		NN			
Embase	Fibromyalgia for neurologists. Disputes frequently consulted	2013	Marchesoni, C.L., Buonanotte, F., Rey, R.		NN			
Embase	Fibromyalgia interacts with age to change the brain	2013	Ceko, M., Bushnell, M.C., Fitzcharles, M.-A., Schweinhardt, P.	Y				
Embase	How to treat: Fibromyalgia	2013	Choy, E.		NN			
Embase	Ipsilateral cortical activation in fibromyalgia patients during brushing correlates with symptom severity	2013	Fallon, N., Chiu, Y.H., Li, X., Nurmikko, T.J., Stancak, A.	Y				
Embase	Mechanisms of Pain Control in Chronic Pain Patients	2013			YN	Y	N	Study protocol only
Embase	Muscle fatigue in fibromyalgia is in the brain, not in the muscles: A case-control study of perceived versus objective muscle fatigue	2013	Bandak, E., Amris, K., Bliddal, H., Danneskiold-Samsøe, B., Henriksen, M.	Y				
Embase	Overlapping structural and functional brain changes in patients with long-term exposure to fibromyalgia pain	2013	Jensen, K.B., Srinivasan, P., Spaeth, R., Tan, Y., Kosek, E., Petzke, F., Carville, S., Fransson, P., Marcus, H., Williams, S.C.R., Choy, E.,	Y				

			Vitton, O., Gracely, R., Ingvar, M., Kong, J.					
Embase	Pain and psychiatric disorders-Pain, anxiety and depression	2013	Gormsen, L.		NN			
Embase	Pain treatment by tDCS of the human primary motor cortex	2013	Hansen, N.		NN			
Embase	Placebo tDCS induces acute changes in the endogenous mu-opioid system	2013	DosSantos, M.F., Martikainen, I.K., Nascimento, T.D., Love, T.M., Deboer, M.D., Zubieta, J.K., DaSilval, A.F.		NN			
Embase	Primary Motor Cortex Plasticity and the Bottom up Effect of Deep Intramuscular Needling Stimulation Therapy (DIMST)in Osteoarthritis Chronic Pain	2013			NN			
Embase	Small fibre pathology in patients with fibromyalgia syndrome	2013	Üçeyler, N., Zeller, D., Kahn, A.-K., Kewenig, S., Kittel-Schneider, S., Schmid, A., Casanova-Molla, J., Reiners, K., Sommer, C.		NN			
Embase	Structural alterations in brainstem of fibromyalgia syndrome patients correlate with sensitivity to mechanical pressure	2013	Fallon, N., Alghamdi, J., Chiu, Y., Sluming, V., Nurmikko, T., Stancak, A.	Y				
Embase	Towards a better understanding of learning and memory processes in abdominal pain: Evidence of enhanced reactivation of classically-conditioned fear memories in healthy women compared to males	2013	Elsenbruch, S., Kattoor, J., Theysohn, N., Kotsis, V., Benson, S.		NN			
Embase	Treatment with paroxetine increases levels of nociceptine in cerebrospinal fluid in females with fibromyalgia	2013	Tanum, L.H., Vinje, M., Ordeberg, G., Nyberg, F.		NN			
Embase	White matter microstructural integrity assessment in fibromyalgia using cardiac-gated diffusion tensor imaging	2013	Moana-Filho, E., Tchivileva, I., Gracely, R.		YY	Y	N	conference abstract only – no full text available.
Embase	A Brain Imaging Study of Tai Chi on Fibromyalgia	2012			YY	Y	N	Study protocol only
Embase	Altered resting connectivity between the insula and cingulate	2012	Ichesco, E., Schmidt-Wilcke, T., Clauw, D., Peltier, S., Williams, D., Harris, R.		YY	Y	N	conference abstract only – no full text available.

	cortex is related to chronic fibromyalgia pain					
Embase	An exploratory study of dissociated states during sleep in patients with fibromyalgia	2012	Oliveira, T.F., Ferreira, L., Sanches, J., Paiva, T.		NN	
Embase	Auditory evoked potential as a method of diagnosis of hypervigilance in fibromyalgia	2012	Kamel, N.S., Kamal, N.M., El-Ganzouri, A.M., Omar, A.N., Al-Zifzaf, D.S., Abdel-Kader, A.A.		NN	
Embase	Behavioral and neuronal investigations of hypervigilance in patients with fibromyalgia syndrome	2012	Tiemann, L., Schulz, E., Winkelmann, A., Ronel, J., Henningsen, P., Ploner, M.	Y		
Embase	Brain dysfunction in fibromyalgia and somatization disorder using proton magnetic resonance spectroscopy: A controlled study	2012	Fayed, N., Andres, E., Rojas, G., Moreno, S., Serrano-Blanco, A., Roca, M., Garcia-Campayo, J.	Y		
Embase	Centrally altered pain processing in sickle cell disease: A preliminary study	2012	Zempsky, W.T., Stevens, M., Santanelli, J.P., Gaynor, L.		YN	N
Embase	Cerebral mechanisms of experimental hyperalgesia in fibromyalgia	2012	Burgmer, M., Pfleiderer, B., Maihöfner, C., Gaubitz, M., Wessolleck, E., Heuft, G., Pogatzki-Zahn, E.	Y		
Embase	Chronic fatigue syndrome, the immune system and viral infection	2012	Bansal, A.S., Bradley, A.S., Bishop, K.N., Kiani-Alikhan, S., Ford, B.		NN	
Embase	Cognitive Dysfunction in Fibromyalgia Patients: Specific Neuro-psychological Dysfunctions, Psychiatric Comorbidity and Integrative Assessments	2012			NN	
Embase	Complex systems approaches to stress-arousal & psychobiological data	2012	Aschbacher, K.		NN	
Embase	Differences in metabolite-detecting, adrenergic, and immune gene expression after moderate exercise in patients with chronic fatigue syndrome, patients with multiple sclerosis, and healthy controls	2012	White, A.T., Light, A.R., Hughen, R.W., Vanhaitsma, T.A., Light, K.C.		NN	
Embase	Disrupted functional connectivity of the pain network in fibromyalgia	2012	Cifre, I., Sitges, C., Fraiman, D., Muñoz, M.A., Balenzuela, P., González-Roldán, A., Martínez-	Y		

			Jauand, M., Birbaumer, N., Chialvo, D.R., Montoya, P.					
Embase	Dynamic Contrast Enhanced MRI of the Hands in Patients With Fibromyalgia. A Pilot Study	2012			NN			
Embase	Effective connectivity among brain regions associated with slow temporal summation of C-fiber-evoked pain in fibromyalgia patients and healthy controls	2012	Craggs, J.G., Staud, R., Robinson, M.E., Perlstein, W.M., Price, D.D.	Y				
Embase	Effects of Direct Transcranial Current Stimulation on Central Neural Pain Processing in Fibromyalgia	2012			YN	N		
Embase	Effects of pregabalin on visual activation of the insular cortex in patients with fibromyalgia: An fMRI study	2012	Ichesco, E., Huggins, J., Pauer, L., Clauw, D., Harte, S., Harris, R.		YN	Y	N	conference abstract only – no full text available.
Embase	Functional connectivity during moderate pain in women with fibromyalgia	2012	Shields, M., Ellingson, L., Stegner, A., Cook, D.		YY	Y	N	conference abstract only – no full text available.
Embase	Functional magnetic resonance imaging of working memory in fibromyalgia: Support for a “competing demands” theory of cognitive function in chronic pain	2012	Kairys, A.E., Ramirez, G., Ichesco, E., Hampson, J.P., Harris, R.E., Clauw, D.J., Tobias, S.-W., Glass, J.M.		YY	Y	N	conference abstract only – no full text available.
Embase	Gene expression alterations at baseline and following moderate exercise in patients with Chronic Fatigue Syndrome and Fibromyalgia Syndrome	2012	Light, A.R., Bateman, L., Jo, D., Hughen, R.W., Vanhaitsma, T.A., White, A.T., Light, K.C.		NN			
Embase	Initial assessment of the dopamine system in fibromyalgia	2012	Albrecht, D., MacKie, P., Christian, B., Brown-Proctor, C., Federici, L., Kareken, D., Herring, C., Walters, J., Yoder, K.		YY	Y	N	conference abstract only – no full text available.
Embase	Linking disease symptoms and subtypes with personalized systems-based phenotypes: A proof of concept study	2012	Aschbacher, K., Adam, E.K., Crofford, L.J., Kemeny, M.E., Demitrack, M.A., Ben-Zvi, A.	Y				
Embase	Pain processing regions in fibromyalgia syndrome-functional magnetic resonance imaging study in Korea	2012	Kim, S., Kim, G., Lee, J., Mun, C., Lee, Y., Lee, S.		YY	Y	N	conference abstract only – no full text available.

Embase	Patients with fibromyalgia display less functional connectivity in the brain's pain inhibitory network	2012	Jensen, K.B., Loitoile, R., Kosek, E., Petzke, F., Carville, S., Fransson, P., Marcus, H., Williams, S.C.R., Choy, E., Mainguy, Y., Vitton, O., Gracely, R.H., Gollub, R., Ingvar, M., Kong, J.	Y				
Embase	Reduced insular γ-aminobutyric acid in fibromyalgia	2012	Foerster, B.R., Petrou, M., Edden, R.A.E., Sundgren, P.C., Schmidt-Wilcke, T., Lowe, S.E., Harte, S.E., Clauw, D.J., Harris, R.E.	Y				
Embase	Self-ratings of higher olfactory acuity contrast with reduced olfactory test results of fibromyalgia patients	2012	Lötsch, J., Kraetsch, H.-G., Wendler, J., Hummel, T.	Y				
Embase	The 5-HT2A T102C polymorphism is not related to thermal pain perception/modulation in subjects with or without chronic widespread pain	2012	Potvin, S., Larouche, A., Gaumond, I., Grignon, S., Marchand, S.		NN			
Embase	The relationship between balance and knee proprioception in patients with fibromyalgia	2012	Ulus, Y., Akyol, Y., Tander, B., Bilgici, A., Kuru, O.		NN			
Embase	Transcranial Direct Current Stimulation (tDCS) as Treatment Method for Pain in Fibromyalgia	2012			YY	Y	N	Study protocol only
Embase	Transcranial direct current stimulation does neither modulate results of a quantitative sensory testing protocol nor ratings of suprathreshold heat stimuli in healthy volunteers	2012	Jürgens, T.P., Schulte, A., Klein, T., May, A.		NN			
Embase	Working memory impairment in fibromyalgia patients associated with altered frontoparietal memory network	2012	Seo, J., Kim, S.-H., Kim, Y.-T., Song, H.-j., Lee, J.-j., Kim, S.-H., Han, S.W., Nam, E.J., Kim, S.-K., Lee, H.J., Lee, S.-J., Chang, Y.	Y				
Embase	A Phase II, Multi-Center, Randomized, Double-Blind, Placebo-Controlled, Dose-Response, Study To Assess The Clinical Benefit Of Droxidopa and	2011			NN			

	Droxidopa/Carbidopa In Subjects With Fibromyalgia				
Embase	Altered brain and cardiac responses to anger and pain faces in chronic pain patients	2011	Gonzalez-Roldan, A.M., Muñoz, M.A., Martinez-Jauand, M., Cifre, I., Sitges, C., Montoya, P.		NN
Embase	Are fibromyalgic patients hypervigilant to non-painful stimulation? A test with the loudness dependence of the auditory evoked potentials	2011	Carrillo-De-La-Peña, M.T., Triñanes, Y., González-Villar, A., Gómez-Perretta, C.		NN
Embase	Association of Membrane Polyunsaturated Fatty Acid Content and Intracellular Magnesium Concentration With Mental and Physical Symptoms in Fibromyalgia and SLE: a Case-control Study	2011			NN
Embase	Behavioral and neurophysiological investigations of hypervigilance in patients with fibromyalgia syndrome	2011	Tiemann, L., Schulz, E., Winkelmann, A., Henningsen, P., Ronel, J., Ploner, M.		NN
Embase	Central mechanisms during fatiguing muscle exercise in muscular dystrophy and fibromyalgia syndrome: A study with transcranial magnetic stimulation	2011	Schwenkreis, P., Voigt, M., Hasenbring, M., Tegenthoff, M., Vorgerd, M., Kley, R.A.	Y	
Embase	Cerebral activation and catastrophizing during pain anticipation in patients with fibromyalgia	2011	Burgmer, M., Petzke, F., Giesecke, T., Gaubitz, M., Heuft, G., Pfleiderer, B.	Y	
Embase	Cerebral blood flow alterations in pain-processing regions of patients with fibromyalgia using perfusion MR imaging	2011	Foerster, B.R., Petrou, M., Harris, R.E., Barker, P.B., Hoeffner, E.G., Clauw, D.J., Sundgren, P.C.	Y	
Embase	Consideration of sleep dysfunction in rehabilitation	2011	Valenza, M.C., Rodenstein, D.O., Fernández-de-las-Peñas, C.		NN
Embase	Cortical oscillatory changes during mechanical brushing in fibromyalgia syndrome patients	2011	Fallon, N., Chiu, Y.H., Li, X., Nurmikko, T., Stancak, A.		NN
Embase	Differential central pain processing following repetitive intramuscular proton/prostaglandin E2	2011	Diers, M., Schley, M.T., Rance, M., Yilmaz, P., Lauer, L., Rukwied, R., Schmelz, M., Flor, H.	Y	

	injections in female fibromyalgia patients and healthy controls							
Embase	Efficacy of executive functioning in fibromyalgia patients	2011	Sondaal, S.F., Oosterman, J., Veldhuijzen, D.S.		NN			
Embase	Executive function in chronic pain patients and healthy controls: Different cortical activation during response inhibition in fibromyalgia	2011	Glass, J.M., Williams, D.A., Fernandez-Sanchez, M.-L., Kairys, A., Barjola, P., Heitzeg, M.M., Clauw, D.J., Schmidt-Wilcke, T.	Y				
Embase	Gray matter volumes of pain-related brain areas are decreased in fibromyalgia syndrome	2011	Robinson, M.E., Craggs, J.G., Price, D.D., Perlstein, W.M., Staud, R.	Y				
Embase	How does physical activity relate to cognitive pain modulation in women?	2011	Shields, M., Ellingson, L., Stegner, A., Cook, D.		YY	Y	N	conference abstract only – no full text available.
Embase	Is fibromyalgia a neuropathic pain disease?	2011	Martin Soelch, C., Ledermann, K., Jenewein, J., Hasler, G., Schnyder, U., Burger, C., Johayem, A., Cservenyak, T., Kollias, S., Buck, A., Sprott, H.		NN			
Embase	Juvenile-onset small-fiber polyneuropathy: A treatable cause of chronic pain and multi-somatic complaints in the young	2011	Oaklander, A.L., Klein, M.M.		NN			
Embase	Migraine and restless legs syndrome	2011	Akdał Uzun, Z., Kurt, S., Karaer Ünaldi, H.		NN			
Embase	Musculoskeletal pain: Basic central mechanisms	2011	Curatolo, M.		NN			
Embase	Neuroimaging Effects of Cognitive Behavioral Therapy in Fibromyalgia	2011			YY	Y	N	Study protocol only
Embase	Perceived cognitive dysfunction in fibromyalgia syndrome	2011	Williams, D.A., Clauw, D.J., Glass, J.M.		NN			
Embase	Prevalence, sensitivity, and specificity of chronic cerebrospinal venous insufficiency in MS	2011	Zivadinov, R., Marr, K., Cutter, G., Ramanathan, M., Benedict, R.H.B., Kennedy, C., Elfadil, M., Yeh, A.E., Reuther, J., Brooks, C., Hunt, K., Andrews, M., Carl, E., Dwyer, M.G., Hojnacki, D., Weinstock-Guttman, B.		NN			
Embase	Prolonged sleep disruption and musculoskeletal sensitization alter sleep and cytokines	2011	Sutton, B., Opp, M.R.		NN			

Embase	Quantitative sensory testing profiles in chronic back pain are distinct from those in fibromyalgia	2011	Blumenstiel, K., Gerhardt, A., Rolke, R., Bieber, C., Tesarz, J., Friederich, H.-C., Eich, W., Treede, R.-D.		NN			
Embase	Reduced insular gamma-aminobutyric acid in fibromyalgia	2011	Foerster, B., Petrou, M., Edden, R., Sundgren, P., Schmidt-Wilcke, T., Lowe, S.E., Harte, S.		YN	N		
Embase	Sleep architecture in patients with fibromyalgia	2011	González, J.L.B., Fernández, T.V.S., Rodríguez, L.A., Muñiz, J., Giráldez, S.L., Fernández, A.A.	Y				
Embase	The effect of electrical stimulation on excitability of the corticomotor pathway	2011	Chipchase, L., Schabrun, S., Hodges, P.		NN			
Embase	Transcranial sonography and clinical findings in fibromyalgia patients - A pilot study	2011	Moscoso, A.S.C., Shu, E.B.-S., Galhardoni, R., Teixeira, M.J., Cecilio, S.B., Pinto, L.F., Nascimento, C.N.G., Araujo, H.A., Kaziyama, H.H.S., Fonoff, E.T., Raicher, I., Andrade, D.C.		NN			
Embase	Urocortins and Musculoskeletal Hyperalgesia	2011			YN	N		
Embase	5-HT3 receptor subunit type a gene (HTR3A) polymorphism is associated with anxiety and increased amygdala responsiveness in healthy controls and irritable bowel syndrome (IBS) patients	2010	Kilpatrick, L.A., Labus, J.S., Coveleskie, K., Hammer, C., Jarcho, J., McRoberts, J.A., Rappold, G., Bueller, J., Suyenobu, B., Papp, J.C., Dandekar, S., Tillisch, K., Niesler, B., Mayer, E.A.		NN			
Embase	Alteration of delay and trace eyeblink conditioning in fibromyalgia patients	2010	Nees, F., Rüddel, H., Mussgay, L., Kuehl, L.K., Römer, S., Schächinger, H.	Y				
Embase	Altered resting connectivity between individuals with fibromyalgia and healthy controls	2010	Schmidt-Wilcke, T., Bhavsar, R., Clauw, D.J., Williams, D.A.		YY	Y	N	conference abstract only – no full text available.
Embase	Cerebral changes detected in fibromyalgia syndrome using novel analysis of 3-D structural magnetic resonance imaging	2010	Kwiatek, R., Crouch, B., Barnden, L., Pile, K.		YY	Y	N	conference abstract only – no full text available.
Embase	Comorbidity of pain and psychoaffective diseases - An update	2010	Sommer, C.		NN			
Embase	Depression, anxiety, health-related quality of life and pain in	2010	Gormsen, L., Rosenberg, R., Bach, F.W., Jensen, T.S.	Y				

	patients with chronic fibromyalgia and neuropathic pain				
Embase	Differential effects of painful and non-painful stimulation on tactile processing in fibromyalgia syndrome and subjects with masochistic behaviour	2010	Pollok, B., Krause, V., Legrain, V., Ploner, M., Freynhagen, R., Melchior, I., Schnitzler, A.	Y	
Embase	Diffuse Noxious Inhibitory Controls (DNIC): Nociceptive Modulation and Interaction With Neurocognitive Performance in Chronic Pain	2010			NN
Embase	Distorting proprioception in patients with rheumatic diseases exacerbates sensory disturbances: Further evidence for central pain mechanisms	2010	Cohen, H., Harris, N., McCabe, C.		NN
Embase	Electrified minds: Transcranial direct current stimulation (tDCS) and Galvanic Vestibular Stimulation (GVS) as methods of non-invasive brain stimulation in neuropsychology-A review of current data and future implications	2010	Utz, K.S., Dimova, V., Oppenländer, K., Kerkhoff, G.		NN
Embase	Endogenous opioid system at the interface of emotion and pain regulatory mechanisms	2010	Zubieta, J.-K.		NN
Embase	Evidence of reduced sympatho-adrenal and hypothalamic-pituitary activity during static muscular work in patients with fibromyalgia	2010	Kadetoff, D., Kosek, E.	Y	
Embase	Fibromyalgia is associated with a disruption of emotional modulation of pain, but not emotional modulation of spinal nociception	2010	DelVentura, J.L., Terry, E.L., Bartley, E.J., Vincent, A., Olech, E., Rhudy, J.L.		NN
Embase	Fibromyalgia unique temporal brain activation during experimental pain: A controlled fMRI Study	2010	Burgmer, M., Pogatzki-Zahn, E., Gaubitz, M., Stüber, C., Wessoleck, E., Heuft, G., Pfleiderer, B.	Y	
Embase	Increased glutamate/glutamine compounds in the brains of patients with fibromyalgia: A	2010	Valdés, M., Collado, A., Bargalló, N., Vázquez, M.,	Y	

	magnetic resonance spectroscopy study		Rami, L., Gómez, E., Salamero, M.					
Embase	Intrinsic brain connectivity in fibromyalgia is associated with chronic pain intensity	2010	Napadow, V., LaCount, L., Park, K., As-Sanie, S., Clauw, D.J., Harris, R.E.	Y				
Embase	Is it all central sensitization? Role of peripheral tissue nociception in chronic musculoskeletal pain	2010	Staud, R.		NN			
Embase	Localized 1H-NMR spectroscopy in patients with fibromyalgia: A controlled study of changes in cerebral glutamate/glutamine, inositol, choline, and N-acetylaspartate	2010	Fayed, N., Garcia-Campayo, J., Magallón, R., Andrés-Bergareche, H., Luciano, J.V., Andres, E., Beltrán, J.	Y				
Embase	Pain Modulation in RA - Influence of Adalimumab. A Randomized, Placebo-controlled Study Using Functional Magnetic Resonance Imaging (PARADE)	2010			NN			
Embase	Quantitative electroencephalographic abnormalities in fibromyalgia patients	2010	Hargrove, J.B., Bennett, R.M., Simons, D.G., Smith, S.J., Nagpal, S., Deering, D.E.	Y				
Embase	Resting fMRI findings in Korean patients with fibromyalgia	2010	Kim, S.H., Chang, Y., Seo, J.		YY	Y	N	conference abstract only – no full text available.
Embase	SPECT imaging of the brain: Regional cerebral blood flow before and after treatment of patients with primary fibromyalgia	2010	Osman, M., Hajji, O., Abdul Nasser, O., Khodair, A., Al Sarraf, N.		NN			
Embase	Unraveling Impaired Pain Inhibition in Patients With Rheumatoid Arthritis and Central Sensitivity Syndromes: a Series of Experiments Targeting Brain Neurotransmission	2010			NN			
Web Of Science	An ALE meta-analysis of pain processing alterations in fibromyalgia: Toward an evidence-based process model	2025	Cavicchioli, M, Caruso, A, Scalabrini, A, Torelli, A, Bottiroli, S, Pichiecchio, A, Prodi, E, Cangelosi, M, Lai, C, Vitali, P, Sconfienza, LM, Sarzi-Puttini, P, Galli, F	Y				
Web Of Science	Brain structural differences between fibromyalgia patients and healthy control subjects: a	2025	Agoalikum, E, Wu, HZ, Klugah-Brown, B, Maes, M	Y				

	source-based morphometric study				
Web Of Science	Chronic pain is associated with greater brain entropy in the prefrontal cortex	2025	Del Mauro, G, Li, YR, Yu, JA, Kochunov, P, Sevel, LS, Boissoneault, J, Chen, S, Wang, Z	Y	
Web Of Science	Combined Functional and Structural Imaging of White Matter Reveals Brain Connectivity Alterations in Fibromyalgia Patients	2025	Gao, ZY, Xie, XY, Liu, FY, Xu, T, Zhang, N, Zhang, XR, Li, Y, Kong, YY, Lv, DL, Wu, T	Y	
Web Of Science	Common neural correlates of chronic pain - A systematic review and meta-analysis of resting-state fMRI studies	2025	Fiúza-Fernandes, J, Pereira-Mendes, J, Esteves, M, Radua, J, Picó-Pérez, M, Leite-Almeida, H		NN
Web Of Science	Elevated posterior insula glutamate in patients with sickle cell disease	2025	Zhou, XP, Ichesco, E, Pucka, AQ, Liu, ZY, O'Brien, AR, Harte, SE, Harris, RE, Wang, Y	Y	
Web Of Science	Enhanced behavioural and neural sensitivity to punishments in chronic pain and fatigue	2025	Mancini, F, Mahajan, P, Guttesen, AA, Onysk, J, Scholtes, I, Shenker, N, Lee, M, Seymour, B		NN
Web Of Science	Fibromyalgia and the painful self: A meta-analysis of resting-state fMRI data	2025	Cavicchioli, M, Scalabrini, A, Nimbi, F, Torelli, A, Bottiroli, S, Pichiecchio, A, Prodi, E, Trentini, C, Sarzi-Puttini, P, Galli, F	Y	
Web Of Science	Fractional Amplitude of Low-Frequency Fluctuations and Regional Homogeneity Analyses Revealed Altered Local Spontaneous Neural Activities in Ankylosing Spondylitis	2025	Lin, CR, Xie, Y, Liu, D, Liu, BD, Gu, JR, Wang, XH, Qin, J		NN
Web Of Science	Functional brain changes in Mexican women with fibromyalgia	2025	Elkana, O, Beheshti, I	Y	
Web Of Science	Investigating the neural correlates of the left thalamus in women with fibromyalgia: A Granger causality and voxel-based morphometry approach	2025	Agoalikum, E, Wu, HZ, Klugah-Brown, B, Maes, M	Y	
Web Of Science	Looking at Burning Mouth Syndrome Brain: The Prevalence of Idiopathic Intracranial Hypertension Radiologic Signs in BMS Patients	2025	Mignogna, MD, Coppola, N, Leuci, S, Sansone, M, Canfora, F, Adamo, D, De Simone, R		NN

Web Of Science	Muscle and cerebral oxygenation during exercise in fibromyalgia: a near-infrared spectroscopy study	2025	Lehto, T, Zetterman, T, Gagnon, D, Markkula, R, Arokoski, J, Kalso, E, Peltonen, JE	Y	
Web Of Science	Patient subtyping in juvenile fibromyalgia: the role of multisensory hypersensitivity and neurophysiological correlates	2025	Martín-Herrero, L, Suñol, M, Pascual-Diaz, S, Ting, T, Dudley, JA, Jackson, C, Kashikar-Zuck, S, Coghill, RC, López-Solà, M	Y	
Web Of Science	Peak alpha frequency differs between chronic back pain and chronic widespread pain	2025	McLain, N, Cavaleri, R, Kutch, J		NN
Web Of Science	Resting-state functional connectivity between the frontoparietal network and the default mode network is aberrantly increased in ankylosing spondylitis	2025	Liu, D, Lin, CR, Liu, BD, Zhang, YL, Jiang, YT, Gu, JR, Jin, O		NN
Web Of Science	Serum Interleukin-8 Levels and Their Association with Anxiety and Functional Disability in Military Personnel with Chronic Low Back Pain	2025	Dhahri, R, Ben Ayed, H, Dergaa, I, Ceylan, HI, Tazaghdanti, A, Kochkar, R, Ghazouani, E, Fenniche, I, Ben Ammar, L, Jebri, R, Dorgham, I, Slouma, M, Muntean, RI, Gharsallah, I	Y	
Web Of Science	Temporal interaction information and laser evoked responses: preliminary results in fibromyalgia patients with small fibers pathology	2025	Clemente, L, La Rocca, M, Stramaglia, S, Marinazzo, D, Lombardi, R, Lauria, G, de Tommaso, M	Y	
Web Of Science	Temporal lobe dysfunction for comorbid depressive symptoms in postherpetic neuralgia patients	2025	Wu, Y, Wang, C, Qian, W, Wang, LJ, Yu, LN, Zhang, MM, Yan, M		NN
Web Of Science	Two Neuroanatomical Subtypes in Fibromyalgia Patients: Distinct Morphological Patterns and Treatment Outcomes	2025	Wu, SY, Jing, B, Wang, YD, Long, MJ, Li, YT, Li, ZH, Jiao, J	Y	
Web Of Science	"Neuroinflammation": does it have a role in chronic pain? Evidence from human imaging	2024	Loggia, ML		NN
Web Of Science	A Comparative Analysis of Cognitive Deficits in Rheumatoid Arthritis and Fibromyalgia: Impact of Symptoms Severity and Its Clinical Implications	2024	Galvez-Sánchez, CM, Duschek, S, del Paso, GAR		NN

Web Of Science	Abnormal functional neurocircuitry underpinning emotional processing in fibromyalgia	2024	Balducci, T, Garza-Villarreal, EA, Valencia, A, Aleman, A, van Tol, MJ	Y	
Web Of Science	Affective dysfunction mediates the link between neuroimmune markers and the default mode network functional connectivity, and the somatic symptoms in somatic symptom disorder	2024	Park, B, Lee, S, Jang, Y, Park, HY		NN
Web Of Science	Alterations of the resting-state brain network connectivity and gray matter volume in patients with fibromyalgia in comparison to ankylosing spondylitis	2024	Liu, D, Zhang, YL, Zhao, JS, Liu, BD, Lin, CR, Yang, MC, Gu, JR, Jin, O	Y	
Web Of Science	Amygdala self-neuromodulation capacity as a window for process-related network recruitment	2024	Gurevitch, G, Lubianiker, N, Markovits, T, Or-Borichev, A, Sharon, H, Fine, NB, Fruchtman-Steinbok, T, Keynan, JN, Shahar, M, Friedman, A, Singer, N, Hendler, T	Y	
Web Of Science	Cervical and Ocular Vestibular Evoked Myogenic Potentials in Fibromyalgia Syndrome Patients	2024	Dabbous, AO, Baki, NMA, Hassanein, MM, Sheta, SM	Y	
Web Of Science	Disease-specific alterations in central fear network engagement during acquisition and extinction of conditioned interoceptive fear in inflammatory bowel disease	2024	Lanters, LR, Öhlmann, H, Langhorst, J, Theysohn, N, Engler, H, Icenhour, A, Elsenbruch, S		NN
Web Of Science	Disentangling pain and fatigue in chronic fatigue syndrome: a resting state connectivity study before and after cognitive behavioral therapy	2024	van der Schaaf, ME, Geerligs, L, Toni, I, Knoop, H, Oosterman, JM		NN
Web Of Science	Effects of Different Transcranial Direct Current Stimulation Intensities over Dorsolateral Prefrontal Cortex on Brain Electrical Activity and Heart Rate Variability in Healthy and Fibromyalgia Women: A Randomized Crossover Trial	2024	Gomez-Alvaro, MC, Gusi, N, Cano-Plasencia, R, Leon-Llamas, JL, Murillo-Garcia, A, Melo-Alonso, M, Villafaina, S	Y	

Web Of Science	Enhanced motor network engagement during reward gain anticipation in fibromyalgia	2024	Park, SH, Michael, AM, Baker, AK, Lei, CRA, Martucci, KT	Y	
Web Of Science	Identification of Specific Abnormal Brain Functional Activity and Connectivity in Cancer Pain Patients: A Preliminary Resting-State fMRI Study	2024	Hua, YJ, Geng, YK, Liu, SR, Xia, SW, Liu, Y, Cheng, SF, Chen, CM, Pang, CY, Zhao, ZW, Peng, B, Dai, YK, Ji, JS, Wu, D		NN
Web Of Science	Identification of texture MRI brain abnormalities on Fibromyalgia syndrome using interpretable machine learning models	2024	Jiang, HY, Liu, AH, Ying, ZH	Y	
Web Of Science	Investigating Descending Pain Regulation in Fibromyalgia and the Link to Altered Autonomic Regulation by Means of Functional MRI Data	2024	Hassanpour, S, Algitami, H, Umraw, M, Merletti, J, Keast, B, Stroman, PW	Y	
Web Of Science	Pain and the biochemistry of fibromyalgia: patterns of peripheral cytokines and chemokines contribute to the differentiation between fibromyalgia and controls and are associated with pain, fat infiltration and content	2024	Gerdle, B, Leinhard, OD, Lund, E, Lundberg, P, Forsgren, MF, Ghafouri, B		NN
Web Of Science	Probing white matter microstructure in youth with chronic pain and its relation to catastrophizing using neurite orientation dispersion and density imaging	2024	Timmers, I, Biggs, EE, Bruckert, L, Tremblay-McGaw, AG, Zhang, H, Borsook, D, Simons, LE		NN
Web Of Science	Thalamic neurometabolite alterations in chronic low back pain: a common phenomenon across musculoskeletal pain conditions?	2024	Weerasekera, A, Knight, PC, Alshelh, Z, Morrissey, EJ, Kim, M, Zhang, Y, Napadow, V, Anzolin, A, Torrado-Carvajal, A, Edwards, RR, Ratai, EM, Loggia, ML		NN
Web Of Science	The role of the progesterone receptor PROGINS variant in the development of fibromyalgia syndrome and its psychological findings	2024	Nursal, AF, Turk, AC, Kuruca, N, Yigit, S	Y	
Web Of Science	Abnormal immune system response in the brain of women	2023	Mueller, C, Jordan, I, Jones, C, Lawson, P, Younger, JW	Y	

	with Fibromyalgia after experimental endotoxin challenge				
Web Of Science	Anti-satellite glia cell IgG antibodies in fibromyalgia patients are related to symptom severity and to metabolite concentrations in thalamus and rostral anterior cingulate cortex	2023	Fanton, S, Menezes, J, Krock, E, Sandström, A, Tour, J, Sandor, K, Jurczak, A, Hunt, M, Baharpoor, A, Kadetoff, D, Jensen, KB, Fransson, P, Ellerbrock, I, Sitnikov, R, Svensson, CI, Kosek, E	Y	
Web Of Science	Brain mediators of negative affect-induced physical symptom reporting in patients with functional somatic syndromes	2023	Bogaerts, K, van den Houte, M, Jongen, D, Ly, HG, Coppens, E, Schruers, K, Van Diest, I, Jan, TC, Van Wambeke, P, Petre, B, Kragel, PA, Lindquist, MA, Wager, TD, Van Oudenhove, L, van den Bergh, O	Y	
Web Of Science	Brain morphometric changes in fibromyalgia and the impact of psychometric and clinical factors: a volumetric and diffusion-tensor imaging study	2023	Mosch, B, Hagena, V, Herpertz, S, Diers, M	Y	
Web Of Science	Characteristic oscillatory brain networks for predicting patients with chronic migraine	2023	Hsiao, FJ, Chen, WT, Wu, YT, Pan, LLH, Wang, YF, Chen, SP, Lai, KL, Coppola, G, Wang, SJ	Y	
Web Of Science	Chronic pain in osteoarthritis of the hip is associated with selective cognitive impairment	2023	Kazim, MA, Strahl, A, Moritz, S, Arlt, S, Niemeier, A		NN
Web Of Science	Cognitive and mental fatigue in chronic pain: cognitive functions, emotional aspects, biomarkers and neuronal correlates-protocol for a descriptive cross-sectional study	2023	Möller, MC, Berginström, N, Ghafouri, B, Holmqvist, A, Löfgren, M, Nordin, L, Stålnacke, BM		NN
Web Of Science	Decreased DTI-ALPS and choroid plexus enlargement in fibromyalgia: a preliminary multimodal MRI study	2023	Tu, Y, Li, Z, Xiong, F, Gao, F	Y	
Web Of Science	Distinct neural signaling characteristics between fibromyalgia and provoked vestibulodynia revealed by means of functional magnetic	2023	Ioachim, G, Warren, HJM, Powers, JM, Staud, R, Pukall, CF, Stroman, PW	Y	

	resonance imaging in the brainstem and spinal cord				
Web Of Science	Dysfunctional Activation of the Dorsolateral Prefrontal Cortex During Pain Anticipation Is Associated With Altered Subsequent Pain Experience in Fibromyalgia Patients	2023	Sandström, A, Ellerbrock, I, Tour, J, Kadetoff, D, Jensen, K, Kosek, E	Y	
Web Of Science	Evidence of neuroinflammation in fibromyalgia syndrome: a [18F]DPA-714 positron emission tomography study	2023	Mueller, C, Fang, YHD, Jones, C, Mcconathy, JE, Raman, F, Lapi, SE, Younger, JW	Y	
Web Of Science	Functional Magnetic Resonance Imaging Signal Variability Is Associated With Neuromodulation in Fibromyalgia	2023	Lim, M, Kim, DJ, Nascimento, TD, Ichesco, E, Kaplan, C, Harris, RE, DaSilva, AF	Y	
Web Of Science	Neural correlates of control over pain in fibromyalgia patients	2023	Mosch, B, Hagena, V, Herpertz, S, Ruttorf, M, Diers, M	Y	
Web Of Science	Neuroimaging in Breast Implant Illness: An fMRI Pilot Study	2023	Miseré, RML, Rutten, S, van den Hurk, J, Colaris, MJL, van der Hulst, RRWJ	Y	
Web Of Science	Not a general, symptom-unspecific, transdiagnostic marker for functional symptoms: sensorimotor processing of head control is intact in chronic pain	2023	Regnath, F, Biersack, K, Jaeger, N, Glasauer, S, Lehnen, N		NN
Web Of Science	Psychological characteristics associated with the brain volume of patients with fibromyalgia	2023	Izuno, S, Yoshihara, K, Hosoi, M, Eto, S, Hirabayashi, N, Todani, T, Gondo, M, Hayaki, C, Anno, K, Hiwatashi, A, Sudo, N	Y	
Web Of Science	The effects of a 15-week physical exercise intervention on pain modulation in fibromyalgia: Increased pain-related processing within the cortico-striatal- occipital networks, but no improvement of exercise-induced hypoalgesia	2023	Löfgren, M, Sandström, A, Bileviciute-Ljungar, I, Mannerkorpi, K, Gerdle, B, Ernberg, M, Fransson, P, Kosek, E	Y	
Web Of Science	Topological alterations in white matter structural networks in fibromyalgia	2023	Tu, Y, Wang, JH, Li, Z, Xiong, F, Gao, F	Y	
Web Of Science	A behavioral and brain imaging dataset with focus on emotion	2022	Balducci, T, Rasgado-Toledo, J, Valencia, A, van Tol, MJ, Aleman, A, Garza-Villarreal, EA	Y	

	regulation of women with fibromyalgia				
Web Of Science	Abnormal Visual Evoked Responses to Emotional Cues Correspond to Diagnosis and Disease Severity in Fibromyalgia	2022	Goldway, N, Petro, NM, Ablin, J, Keil, A, Ben Simon, E, Zamir, Y, Weizman, L, Greental, A, Hendler, T, Sharon, H	Y	
Web Of Science	Altered Effective Connectivity of Resting-State Networks by Tai Chi Chuan in Chronic Fatigue Syndrome Patients: A Multivariate Granger Causality Study	2022	Li, YY, Wu, K, Hu, XJ, Xu, TJ, Li, ZH, Zhang, Y, Li, KS		NN
Web Of Science	Altered Pain Processing Associated with Administration of Dopamine Agonist and Antagonist in Healthy Volunteers	2022	Martin, SL, Jones, AKP, Brown, CA, Kobylecki, C, Whitaker, GA, El-Deredy, W, Silverdale, MA	Y	
Web Of Science	Altered Pain in the Brainstem and Spinal Cord of Fibromyalgia Patients During the Anticipation and Experience of Experimental Pain	2022	Ioachim, G, Warren, HJM, Powers, JM, Staud, R, Pukall, CF, Stroman, PW	Y	
Web Of Science	Altered Subprocesses of Working Memory in Patients with Fibromyalgia: An Event-Related Potential Study Using N-Back Task	2022	Mercado, F, Ferrera, D, Fernandes-Magalhaes, R, Peláez, I, Barjola, P	Y	
Web Of Science	Altered brain activity in end-stage knee osteoarthritis revealed by resting-state functional magnetic resonance imaging	2022	Kang, BX, Ma, J, Shen, J, Xu, H, Wang, HQ, Zhao, C, Xie, J, Zhong, S, Gao, CX, Xu, XR, A, XY, Gu, XL, Xiao, LB, Xu, JG		NN
Web Of Science	Altered resting-state functional connectivity within corticostriatal and subcortical-striatal circuits in chronic pain	2022	Park, SH, Baker, AK, Krishna, V, Mackey, SC, Martucci, KT	Y	
Web Of Science	Assessment of retinal nerve fiber thickness and optic nerve head blood flow in female patients diagnosed with fibromyalgia syndrome	2022	Urfalioglu, S, Berk, E	Y	
Web Of Science	Association between descending pain modulatory system and cognitive impairment in fibromyalgia: A cross-sectional exploratory study	2022	Serrano, PV, Zortea, M, Alves, RL, Beltran, G, Deliberali, CB, Maule, A, Torres, ILS, Fregni, F, Caumo, W	Y	

Web Of Science	Attention deficits in Brazilian health care workers with chronic pain	2022	Schmidt, SL, Araguez, IM, Neves, VV, van Duinkerken, E, Schmidt, GJ, Tolentino, JC, Gjorup, ALT		NN
Web Of Science	Auditory change-related cortical response is associated with hypervigilance to pain in healthy volunteers	2022	Otsuru, N, Ogawa, M, Yokota, H, Miyaguchi, S, Kojima, S, Saito, K, Inukai, Y, Onishi, H		NN
Web Of Science	Brain morphometric changes in patients with fibromyalgia	2022	Karayol, KC, Karayol, SS	Y	
Web Of Science	CNS imaging characteristics in fibromyalgia patients with and without peripheral nerve involvement	2022	Aster, HC, Evdokimov, D, Braun, A, Üçeyler, N, Kampf, T, Pham, M, Homola, GA, Sommer, C	Y	
Web Of Science	Central nervous activity during a dot probe task with facial expressions in fibromyalgia	2022	Fischer-Jbali, LR, Montoro, CI, Montoya, P, Halder, W, Duschek, S		NN
Web Of Science	Central pain modulatory mechanisms of attentional analgesia are preserved in fibromyalgia	2022	Oliva, V, Gregory, R, Brooks, JCW, Pickering, AE	Y	
Web Of Science	Cerebral White Matter Alterations Revealed by Multiple Diffusion Metrics in Cervical Spondylotic Patients with Pain: A TBSS Study	2022	Li, D, Xu, H, Yang, Q, Zhang, M, Wang, Y		NN
Web Of Science	Comparison of Pineal Gland Volume Between Patients with Fibromyalgia and Healthy Controls	2022	Karabas, Ç, Karahan, S, Çalis, HT, Koç, A		NN
Web Of Science	Cortical Abnormalities in Patients with Fibromyalgia: A Pilot Study of Surface-Based Morphometry Analysis	2022	Tu, Y, Wang, JH, Xiong, F, Gao, F	Y	
Web Of Science	Diagnostic Ability and Capacity of Optical Coherence Tomography-Angiography to Detect Retinal and Vascular Changes in Patients with Fibromyalgia	2022	Garcia-Martin, E, Tello, A, Vilades, E, Perez-Velilla, J, Cordon, B, Fernandez-Velasco, D, Garcia-Campayo, J, Puebla-Guedea, M, Satue, M		NN
Web Of Science	Disrupted White Matter Microstructure in Patients With Fibromyalgia Owing Predominantly to Psychological Factors: A Diffusion Tensor Imaging Study	2022	Tu, Y, Wang, JH, Xiong, F, Gao, F	Y	

Web Of Science	Distinct CholinomiR Blood Cell Signature as a Potential Modulator of the Cholinergic System in Women with Fibromyalgia Syndrome	2022	Erbacher, C, Vaknine, S, Moshitzky, G, Lobentanzer, S, Eisenberg, L, Evdokimov, D, Sommer, C, Greenberg, DS, Soreq, H, Üçeyler, N	Y	
Web Of Science	Distinct aberrations in cerebral pain processing differentiating patients with fibromyalgia from patients with rheumatoid arthritis	2022	Sandström, A, Ellerbrock, I, Löfgren, M, Altawil, R, Bileviciute-Ljungar, I, Lampa, J, Kosek, E	Y	
Web Of Science	Dynamic Functional Brain Connectivity Underlying Temporal Summation of Pain in Fibromyalgia	2022	Cheng, JC, Anzolin, A, Berry, M, Honari, H, Paschali, M, Lazaridou, A, Lee, J, Ellingsen, DM, Loggia, ML, Grahl, A, Lindquist, MA, Edwards, RR, Napadow, V	Y	
Web Of Science	Effect of Single Session of Anodal M1 Transcranial Direct Current Stimulation-TDCS-On Cortical Hemodynamic Activity: A Pilot Study in Fibromyalgia	2022	La Rocca, M, Clemente, L, Gentile, E, Ricci, K, Delussi, M, de Tommaso, M	Y	
Web Of Science	Effects of Transcranial Direct Current Stimulation on Brain Electrical Activity, Heart Rate Variability, and Dual-Task Performance in Healthy and Fibromyalgia Women: A Study Protocol	2022	Gomez-Alvaro, MC, Villafaina, S, Leon-Llamas, JL, Murillo-Garcia, A, Melo-Alonso, M, Sanchez-Gomez, J, Molero, P, Cano-Plasencia, R, Gusi, N	Y	
Web Of Science	Electrophysiological indices of pain expectation abnormalities in fibromyalgia patients	2022	Barjola, P, Peláez, I, Ferrera, D, González-Gutiérrez, JL, Velasco, L, Peñacoba-Puente, C, López-López, A, Fernandes-Magalhaes, R, Mercado, F	Y	
Web Of Science	Evaluation of visual reaction time in patients with fibromyalgia syndrome	2022	Kurtgil, ME, Kocyigit, BF, Berk, E, Koca, TT, Akyol, A, Nacitarhan, V		NN
Web Of Science	Fibromyalgia: Associations Between Fat Infiltration, Physical Capacity, and Clinical Variables	2022	Gerdle, B, Leinhard, OD, Lund, E, Bengtsson, A, Lundberg, P, Ghafouri, B, Forsgren, MF	Y	
Web Of Science	Functional connectivity and neurotransmitter impairments of the salience brain network in chronic low back pain patients: a combined resting-state functional magnetic resonance imaging and 1H-MRS study	2022	Baumbach, P, Meissner, W, Reichenbach, JR, Gussew, A		NN

Web Of Science	Functional connectivity modulations during offset analgesia in chronic pain patients: an fMRI study	2022	Li, TJ, Zhang, S, Ikeda, E, Kobinata, H	Y	
Web Of Science	Gender influence on clinical manifestations, depressive symptoms and brain-derived neurotrophic factor (BDNF) serum levels in patients affected by fibromyalgia	2022	Iannuccelli, C, Lucchino, B, Gioia, C, Dolcini, G, Rabasco, J, Venditto, T, Ioppolo, F, Santilli, V, Conti, F, Di Franco, M	Y	
Web Of Science	Gray Matter Abnormalities in Patients with Chronic Primary Pain: A Coordinate-Based Meta-Analysis	2022	Wang, ZX, Yuan, ML, Xiao, J, Chen, LL, Guo, XY, Dou, YK, Jiang, FG, Min, WJ, Zhou, B		NN
Web Of Science	Hand size estimates of fibromyalgia patients are associated with clinical and experimental pain	2022	Staud, R, Carpenter, R, Godfrey, M, Robinson, ME		NN
Web Of Science	Identification of Resting-State Network Functional Connectivity and Brain Structural Signatures in Fibromyalgia Using a Machine Learning Approach	2022	Nhu, NT, Chen, DYT, Kang, JH	Y	
Web Of Science	Impaired pain-related threat and safety learning in patients with chronic back pain	2022	Schlitt, F, Schmidt, K, Merz, CJ, Wolf, OT, Kleine-Borgmann, J, Elsenbruch, S, Wiech, K, Forkmann, K, Bingel, U		NN
Web Of Science	Laser evoked potentials in fibromyalgia with peripheral small fiber involvement	2022	Vecchio, E, Quitadamo, SG, Ricci, K, Libro, G, Delussi, M, Lombardi, R, Lauria, G, de Tommaso, M	Y	
Web Of Science	Microstructural Evidence of Neuroinflammation for Psychological Symptoms and Pain in Patients With Fibromyalgia	2022	Lo, YC, Li, TJT, Lin, TC, Chen, YY, Kang, JH	Y	
Web Of Science	Modification of Alpha Brain Oscillatory Activity in Fibromyalgia After Very Low Intensity Transcranial Magnetic Stimulation	2022	Gómez-Arguelles, JM, López, I, Rodríguez-Rojo, IC, Romero, V, Sabater, C, Corral, M, Bruña, R, Maestú, C	Y	
Web Of Science	Morphometric similarity networks discriminate patients with lumbar disc herniation from healthy controls and predict pain intensity	2022	Yang, LL, Vigotsky, AD, Wu, BB, Shen, BL, Yan, ZH, Apkarian, AV, Huang, LJ		NN

Web Of Science	Movement observation activates motor cortex in fibromyalgia patients: a fNIRS study	2022	Gentile, E, Brunetti, A, Ricci, K, Bevilacqua, V, Craighero, L, de Tommaso, M	Y	
Web Of Science	Multimodal MRI of myalgic encephalomyelitis/chronic fatigue syndrome: A cross-sectional neuroimaging study toward its neuropathophysiology and diagnosis	2022	Shan, ZY, Mohamed, AZ, Andersen, T, Rendall, S, Kwiatek, RA, Del Fante, P, Calhoun, VD, Bhuta, S, Lagopoulos, J	Y	
Web Of Science	Neural correlates of the attentional bias towards pain-related faces in fibromyalgia patients: An ERP study using a dot-probe task	2022	Fernandes-Magalhaes, R, Ferrera, D, Peláez, I, Martín-Buro, MC, Carpio, A, De Lahoz, ME, Barjola, P, Mercado, F	Y	
Web Of Science	Reduced midbrain raphe echogenicity in patients with fibromyalgia syndrome	2022	Üçeyler, N, Schliesser, M, Evdokimov, D, Radziwon, J, Feulner, B, Unterecker, S, Rimmele, F, Walter, U	Y	
Web Of Science	Relations between short-term memory and the within-subject variability of experimental pain intensity reports: Results from healthy and Fibromyalgia patients	2022	Canaipa, R, Khallouf, A, Magalhaes, AR, Teodoro, R, Pao-Mole, V, Agostinho, M, Pimentel-Santos, F, Honigman, L, Treister, R		NN
Web Of Science	Relationship between pineal gland, sleep and melatonin in fibromyalgia women: a magnetic resonance imaging study	2022	Leon-Llamas, JL, Villafaina, S, Murillo-Garcia, A, Domínguez, PR, Gusi, N	Y	
Web Of Science	Resting-state magnetoencephalographic oscillatory connectivity to identify patients with chronic migraine using machine learning	2022	Hsiao, FJ, Chen, WT, Pan, LLH, Liu, HY, Wang, YF, Chen, SP, Lai, KL, Coppola, G, Wang, SJ	Y	
Web Of Science	Sound-Induced Flash Illusions Support Cortex Hyperexcitability in Fibromyalgia	2022	Di Stefano, V, Iacono, S, Gagliardo, A, Maggio, B, Guggino, G, Gangitano, M, Monastero, R, Maggio, VR, Bolognini, N, Brighina, F	Y	
Web Of Science	The Impact of Micro RNA-320a Serum Level on Severity of Symptoms and Cerebral Processing of Pain in Patients with Fibromyalgia	2022	Hussein, M, Fathy, W, Abdelaleem, EA, Nasser, M, Yehia, A, Elanwar, R	Y	
Web Of Science	The translocator protein gene is associated with endogenous pain modulation and the balance	2022	Fanton, S, Sandström, A, Tour, J, Kadetoff, D, Schalling, M,	Y	

	between glutamate and γ-aminobutyric acid in fibromyalgia and healthy subjects: a multimodal neuroimaging study		Jensen, KB, Sitnikov, R, Ellerbrock, I, Kosek, E		
Web Of Science	Towards the Objective Identification of the Presence of Pain Based on Electroencephalography Signals' Analysis: A Proof-of-Concept	2022	Segning, CM, Harvey, J, Ezzaidi, H, Fernandes, KBP, da Silva, RA, Ngomo, S		NN
Web Of Science	Uncovering a Genetic Polymorphism Located in Huntingtin Associated Protein 1 in Modulation of Central Pain Sensitization Signaling Pathways	2022	Gloor, Y, Matthey, A, Sobo, K, Mouterde, M, Kosek, E, Pickering, G, Poloni, ES, Cedraschi, C, Ehret, G, Desmeules, JA		NN
Web Of Science	Variability of Reaction Time as a Marker of Executive Function Impairments in Fibromyalgia	2022	Duschek, S, de Guevara, CML, Serrano, MJF, Montoro, CI, Lopez, SP, del Paso, GRA		NN
Web Of Science	A Controlled Thermoalgesic Stimulation Device for Exploring Novel Pain Perception Biomarkers	2021	Nunez-Ibero, M, Camino-Pontes, B, Diez, I, Erramuzpe, A, Martinez-Gutierrez, E, Stramaglia, S, Alvarez-Cienfuegos, JO, Cortes, JM	Y	
Web Of Science	Abnormal neuroinflammation in fibromyalgia and CRPS using [11C]-(R)-PK11195 PET	2021	Seo, S, Jung, YH, Lee, D, Lee, WJ, Jang, JH, Lee, JY, Choi, SH, Moon, JY, Lee, JS, Cheon, GJ, Kang, DH	Y	
Web Of Science	Abnormal neurometabolites in fibromyalgia patients: Magnetic resonance spectroscopy study	2021	Jung, YH, Kim, H, Lee, D, Lee, JY, Lee, WJ, Moon, JY, Choi, SH, Kang, DH	Y	
Web Of Science	Aerobic Exercise Attenuates Pain Sensitivity: An Event-Related Potential Study	2021	Zheng, KY, Chen, CC, Yang, SY, Wang, XQ		NN
Web Of Science	Altered Structural Covariance of Insula, Cerebellum and Prefrontal Cortex Is Associated with Somatic Symptom Levels in Irritable Bowel Syndrome (IBS)	2021	Grinsvall, C, Van Oudenhove, L, Dupont, P, Ryu, HJ, Ljungberg, M, Labus, JS, Tornblom, H, Mayer, EA, Simren, M		NN
Web Of Science	Altered functional connectivity between hypothalamus and limbic system in fibromyalgia	2021	Kong, J, Huang, YT, Liu, J, Yu, SY, Ming, C, Chen, H, Wilson, G, Harvey, WF, Li, W, Wang, CC	Y	
Web Of Science	Altered network architecture of functional brain communities in chronic nociplastic pain	2021	Larkin, TE, Kaplan, CM, Schrepf, A, Ichesco, E, Mawla, I, Harte, SE, Mashour, GA, Clauw, DJ, Harris, RE	Y	

Web Of Science	Brain-Derived Neurotrophic Factor and Immune Cells in Osteoarthritis, Chronic Low Back Pain, and Chronic Widespread Pain Patients: Association with Anxiety and Depression	2021	Dimmek, DJ, Korallus, C, Buyny, S, Christoph, G, Lichtinghagen, R, Jacobs, R, Nugraha, B		NN
Web Of Science	Cerebral Perfusion and Sensory Testing Results Differ in Interstitial Cystitis/Bladder Pain Syndrome Patients with and without Fibromyalgia: A Site-Specific MAPP Network Study	2021	Deutsch, G, Deshpande, H, Lai, HH, Kutch, JJ, Ness, TJ	Y	
Web Of Science	Chronic fatigue syndrome and fibromyalgia-like symptoms are an integral component of the phenome of schizophrenia: neuro-immune and opioid system correlates	2021	Mousa, RF, Al-Hakeim, HK, Alhaideri, A, Maes, M	Y	
Web Of Science	Dynamic alterations of amplitude of low-frequency fluctuations in patients with chronic neck pain	2021	Zhang, JB, Xu, T, Wang, LJ, Chen, D, Gong, LS, Chen, HF, Yu, JL, Zhao, L, Gao, Q		NN
Web Of Science	Dysfunctional eating behavior in fibromyalgia and its association with serum biomarkers of brain plasticity (BDNF and S100B): an exploratory study	2021	Elkfury, JL, Antunes, LC, Angoleri, LD, Sipmann, RB, de Souza, A, Torres, ILD, Caumo, W	Y	
Web Of Science	Dysfunctional energy metabolisms in fibromyalgia compared with healthy subjects	2021	Jung, YH, Kim, H, Lee, D, Lee, JY, Moon, JY, Choi, SH, Kang, D	Y	
Web Of Science	Effects of repeated menstrual pain on empathic neural responses in women with primary dysmenorrhea across the menstrual cycle	2021	Wang, C, Liu, Y, Dun, WH, Zhang, T, Yang, J, Wang, K, Mu, JY, Zhang, M, Liu, JX		NN
Web Of Science	Emotional and Attentional Bias in Fibromyalgia: A Pilot ERP Study of the Dot-Probe Task	2021	Cardoso, S, Fernandes, C, Barbosa, F		NN
Web Of Science	Fatigue in irritable bowel syndrome is associated with plasma levels of TNF-α and mesocorticolimbic connectivity	2021	Norlin, AK, Walter, S, Icenhour, A, Keita, Å, Elsenbruch, S, Bednarska, O, Jones, MP, Simon, R, Engström, M		NN
Web Of Science	Fibromyalgia Patients Are Not Only Hypersensitive to Painful Stimuli But Also to Acoustic Stimuli	2021	Staud, R, Godfrey, MM, Robinson, ME	Y	

Web Of Science	How fMRI Analysis Using Structural Equation Modeling Techniques Can Improve Our Understanding of Pain Processing in Fibromyalgia	2021	Warren, HJM, Ioachim, G, Powers, JM, Stroman, PW	Y	
Web Of Science	Impact of Fibromyalgia in the Hippocampal Subfields Volumes of Women-An MRI Study	2021	Leon-Llamas, JL, Villafaina, S, Murillo-Garcia, A, Gusi, N	Y	
Web Of Science	Kynurenine metabolites and ratios differ between Chronic Fatigue Syndrome, Fibromyalgia, and healthy controls	2021	Groven, N, Reitan, SK, Fors, EA, Guzey, IC		NN
Web Of Science	Neural effects of placebo analgesia in fibromyalgia patients and healthy individuals	2021	Frangos, E, Ceko, M, Wang, BQ, Richards, EA, Gracely, JL, Colloca, L, Schweinhardt, P, Bushnell, MC	Y	
Web Of Science	Orthostatic stress testing in myalgic encephalomyelitis/chronic fatigue syndrome patients with or without concomitant fibromyalgia: effects on pressure pain thresholds and temporal summation	2021	van Campen, CMC, Rowe, PC, Verheugt, FWA, Visser, FC		NN
Web Of Science	Polymorphisms of the μ-opioid receptor gene influence cerebral pain processing in fibromyalgia	2021	Ellerbrock, I, Sandström, A, Tour, J, Kadetoff, D, Schalling, M, Jensen, KB, Kosek, E	Y	
Web Of Science	Serotonergic gene-to-gene interaction is associated with mood and GABA concentrations but not with pain-related cerebral processing in fibromyalgia subjects and healthy controls	2021	Ellerbrock, I, Sandström, A, Tour, J, Fanton, S, Kadetoff, D, Schalling, M, Jensen, KB, Sitnikov, R, Kosek, E	Y	
Web Of Science	Spinal Cord Resting State Activity in Individuals With Fibromyalgia Who Take Opioids	2021	Martucci, KT, Weber, KA, Mackey, SC	Y	
Web Of Science	Spinal cord neural activity of patients with fibromyalgia and healthy controls during temporal summation of pain: an fMRI study	2021	Staud, R, Boissoneault, J, Lai, S, Mejia, MS, Ramanlal, R, Godfrey, MM, Stroman, PW	Y	
Web Of Science	Structural and functional thalamocortical connectivity study in female fibromyalgia	2021	Kim, DJ, Lim, M, Kim, JS, Chung, CK	Y	
Web Of Science	The p38/MK2 Axis in Monocytes of Fibromyalgia Syndrome Patients: An Explorative Study	2021	Nugraha, B, Scheibe, R, Korallus, C, Gaestel, M, Gutenbrunner, C		NN

Web Of Science	Virtual Reality-Induced Sensorimotor Conflict Evokes Limb-Specific Sensory Disturbances in Complex Regional Pain Syndrome	2021	Brun, C, Pinard, AM, McCabe, CS, Mercier, C		NN
Web Of Science	5MODIFIER LETTER PRIMEUTR polymorphism in the serotonergic receptor HTR3A gene is differently associated with striatal Dopamine D2/D3 receptor availability in the right putamen in Fibromyalgia patients and healthy controls-Preliminary evidence	2020	Ledermann, K, Hasler, G, Jenewein, J, Sprott, H, Schnyder, U, Martin-Soelch, C	Y	
Web Of Science	A neurophysiological investigation of anticipation to pain in Parkinson's disease	2020	Sarah, ML, Anthony, JKP, Christopher, BA, Christopher, K, Monty, SA		NN
Web Of Science	Aberrant Salience? Brain Hyperactivation in Response to Pain Onset and Offset in Fibromyalgia	2020	Hubbard, CS, Lazaridou, A, Cahalan, C, Kim, J, Edwards, RR, Napadow, V, Loggia, M	Y	
Web Of Science	An fMRI-based neural marker for migraine without aura	2020	Tu, YH, Zeng, F, Lan, L, Li, ZJ, Maleki, N, Liu, B, Chen, J, Wang, CC, Park, J, Lang, C, Yujie, G, Liu, ML, Fu, ZN, Zhang, ZG, Liang, FR, Kong, J	Y	
Web Of Science	Analysis of Epigenetic Age Predictors in Pain-Related Conditions	2020	Kwiatkowska, KM, Bacalini, MG, Sala, C, Kaziyama, H, de Andrade, DC, Terlizzi, R, Giannini, G, Cevoli, S, Pierangeli, G, Cortelli, P, Garagnani, P, Pirazzini, C		NN
Web Of Science	Chronic Pain and Cognition	2020	Hedges, D, Farrer, TJ, Bigler, ED, Hopkins, RO		NN
Web Of Science	Cortical Binding Potential of Opioid Receptors in Patients With Fibromyalgia Syndrome and Reduced Systemic Interleukin-4 Levels - A Pilot Study	2020	Üçeyler, N, Buchholz, HG, Kewenig, S, Ament, SJ, Birklein, F, Schreckenberger, M, Sommer, C	Y	
Web Of Science	DNA Methylation and Brain-Derived Neurotrophic Factor Expression Account for Symptoms and Widespread Hyperalgesia in Patients With Chronic Fatigue Syndrome and Comorbid Fibromyalgia	2020	Polli, A, Ghosh, M, Bakusic, J, Ickmans, K, Monteyne, D, Velkeniers, B, Bekaert, B, Godderis, L, Nijs, J	Y	

Web Of Science	Evidence of Mitochondrial Dysfunction in Fibromyalgia: Deviating Muscle Energy Metabolism Detected Using Microdialysis and Magnetic Resonance	2020	Gerdle, B, Ghafouri, B, Lund, E, Bengtsson, A, Lundberg, P, van Ettinger-Veenstra, H, Leinhard, OD, Forsgren, MF	Y	
Web Of Science	Exploration of Functional Connectivity Changes Previously Reported in Fibromyalgia and Their Relation to Psychological Distress and Pain Measures	2020	van Ettinger-Veenstra, H, Boehme, R, Ghafouri, B, Olausson, H, Wicksell, RK, Gerdle, B	Y	
Web Of Science	Fibromyalgia syndrome-A laser-evoked potentials study unsupportive of small nerve fibre involvement	2020	Van Assche, DCF, Plaghki, L, Masquelier, E, Hatem, SM	Y	
Web Of Science	Hyperparameter-tuned prediction of somatic symptom disorder using functional near-infrared spectroscopy-based dynamic functional connectivity	2020	Eken, A, Çolak, B, Bal, NB, Kusman, A, Kizilpinar, SC, Akaslan, DS, Baskak, B		NN
Web Of Science	Impaired hemodynamic activity in the right dorsolateral prefrontal cortex is associated with impairment of placebo analgesia and clinical symptoms in postherpetic neuralgia	2020	Hibi, D, Takamoto, K, Iwama, Y, Ebina, S, Nishimaru, H, Matsumoto, J, Takamura, Y, Yamazaki, M, Nishijo, H		NN
Web Of Science	Magnetic resonance imaging of neuroinflammation in chronic pain: a role for astrogliosis?	2020	Jung, CJ, Ichesco, E, Ratai, EM, Gonzalez, RG, Burdo, T, Loggia, ML, Harris, RE, Napadow, V	Y	
Web Of Science	Mutual interaction between motor cortex activation and pain in fibromyalgia: EEG-fNIRS study	2020	Gentile, E, Brunetti, A, Ricci, K, Delussi, M, Bevilacqua, V, de Tommaso, M	Y	
Web Of Science	Neural correlates of conditioned pain responses in fibromyalgia subjects indicate preferential formation of new pain associations rather than extinction of irrelevant ones	2020	Sandström, A, Ellerbrock, I, Tour, J, Kadetoff, D, Jensen, KB, Kosek, E	Y	
Web Of Science	Neurochemical Correlates of Brain Atrophy in Fibromyalgia Syndrome: A Magnetic Resonance Spectroscopy and Cortical Thickness Study	2020	Feraco, P, Nigro, S, Passamonti, L, Grecucci, A, Caligiuri, ME, Gagliardo, C, Bacci, A	Y	

Web Of Science	Neuromagnetic Amygdala Response to Pain-Related Fear as a Brain Signature of Fibromyalgia	2020	Hsiao, FJ, Chen, WT, Ko, YC, Liu, HY, Wang, YF, Chen, SP, Lai, KL, Lin, HY, Coppola, G, Wang, SJ	Y	
Web Of Science	Neurophysiological Differences Between Women With Fibromyalgia and Healthy Controls During Dual Task: A Pilot Study	2020	Villafaina, S, Fuentes-García, JP, Cano-Plasencia, R, Gusi, N	Y	
Web Of Science	Pain catastrophizing is associated with the Val66Met polymorphism of the brain-derived neurotrophic factor in fibromyalgia	2020	Alves, CFD, Caumo, W, Silvestri, JM, Zortea, M, dos Santos, VS, Cardoso, DF, Regner, A, de Souza, AH, Simon, D	Y	
Web Of Science	Painful stimulation increases spontaneous blink rate in healthy subjects	2020	Paparella, G, Di Stefano, G, Fasolino, A, Di Pietro, G, Colella, D, Truini, A, Cruccu, G, Berardelli, A, Bologna, M		NN
Web Of Science	Patients with fibromyalgia show increased beta connectivity across distant networks and microstates alterations in resting-state electroencephalogram	2020	González-Villar, AJ, Triñanes, Y, Gómez-Perretta, C, Carrillo-de-la-Peña, MT	Y	
Web Of Science	Reductions in Cerebral Blood Flow Can Be Provoked by Sitting in Severe Myalgic Encephalomyelitis/Chronic Fatigue Syndrome Patients	2020	van Campen, CMC, Rowe, PC, Visser, FC	Y	
Web Of Science	Sifting the wheat from the chaff? Evidence for the existence of an asymmetric fibromyalgia phenotype	2020	Kaziyama, HH, Barbour, J, Galhardoni, R, da Silva, VA, de Siqueira, SRDT, Listik, C, dos Santos, GJ, Yeng, LT, Marcolin, MA, Raicher, I, Teixeira, MJ, de Andrade, DC	Y	
Web Of Science	Sleep stage dynamics in young patients with sleep bruxism	2020	Kishi, A, Haraki, S, Toyota, R, Shiraishi, Y, Kamimura, M, Taniike, M, Yatani, H, Kato, T		NN
Web Of Science	Striatal hypofunction as a neural correlate of mood alterations in chronic pain patients	2020	Kim, M, Mawla, I, Albrecht, DS, Admon, R, Torrado-Carvajal, A, Bergan, C, Protsenko, E, Kumar, P, Edwards, RR, Saha, A, Napadow, V, Pizzagalli, DA, Loggia, ML	Y	
Web Of Science	The Comparison of the Biological Rhythms of Patients with	2020	Bulbul, F, Koca, I, Savas, E, Dokuyucu, R	Y	

	Fibromyalgia Syndrome with Biological Rhythms of Healthy Controls				
Web Of Science	Altered Cervical Spinal Cord Resting-State Activity in Fibromyalgia	2019	Martucci, KT, Weber, KA, Mackey, SC	Y	
Web Of Science	Altered cerebral pain processing of noxious stimuli from inflamed joints in rheumatoid arthritis: An event-related fMRI study	2019	Sandström, A, Ellerbrock, I, Jensen, KB, Martinsen, S, Altawil, R, Hakeberg, P, Fransson, P, Lampa, J, Kosek, E		NN
Web Of Science	Altered near-infrared spectroscopy response to breath-holding in patients with fibromyalgia	2019	Chen, WT, Yu, CH, Sun, CW		NN
Web Of Science	Altered resting state functional connectivity of the cognitive control network in fibromyalgia and the modulation effect of mind-bodyintervention	2019	Kong, J, Wolcott, E, Wang, ZJ, Jorgenson, K, Harvey, WF, Tao, J, Rones, R, Wang, CC	Y	
Web Of Science	Anger rumination mediates differences between fibromyalgia patients and healthy controls on mental health and quality of life	2019	Toussaint, L, Sirois, F, Hirsch, J, Kohls, N, Weber, A, Schelling, J, Vajda, C, Offenbäecher, M		NN
Web Of Science	Apparent Effects of Opioid Use on Neural Responses to Reward in Chronic Pain	2019	Martucci, KT, MacNiven, KH, Borg, N, Knutson, B, Mackey, SC	Y	
Web Of Science	BDNF and serum S100B levels according the spectrum of structural pathology in chronic pain patients	2019	Stefani, LC, Leite, FM, Tarragó, MDL, Zanette, SA, de Souza, A, Castro, SM, Caumo, W	Y	
Web Of Science	Brain Electrical Activity Associated With Visual Attention and Reactive Motor Inhibition in Patients With Fibromyalgia	2019	González-Villar, AJ, Arias, M, Carrillo-de-la-Peña, MT	Y	
Web Of Science	Brain glial activation in fibromyalgia - A multi-site positron emission tomography investigation	2019	Albrecht, DS, Forsberg, A, Sandström, A, Bergan, C, Kadetoff, D, Protsenko, E, Lampa, J, Lee, YC, Höglund, CO, Catana, C, Cervenka, S, Akeju, O, Lekander, M, Cohen, G, Halldin, C, Taylor, N, Kim, M, Hooker, JM, Edwards, RR, Napadow, V, Kosek, E, Loggia, ML	Y	

Web Of Science	Brain responses to other people's pain in fibromyalgia: a magnetoencephalography study	2019	Goldstein, A, Zeev-Wolf, M, Herz, N, Ablin, JN	Y				
Web Of Science	Correlation Between Thalamus-Related Functional Connectivity and Serum BDNF Levels During the Periovulatory Phase of Primary Dysmenorrhea	2019	Han, F, Liu, HJ, Wang, K, Yang, J, Yang, L, Liu, JX, Zhang, M, Dun, WH		NN			
Web Of Science	Differential Neuroplastic Changes in Fibromyalgia and Depression Indexed by Up-Regulation of Motor Cortex Inhibition and Disinhibition of the Descending Pain System: An Exploratory Study	2019	Cardinal, TM, Antunes, LC, Brietzke, AP, Parizotti, CS, Carvalho, F, De Souza, A, Torres, ILD, Fregni, F, Caumo, W	Y				
Web Of Science	Frequency-specific alterations in cortical rhythms and functional connectivity in trigeminal neuralgia	2019	Zhang, YY, Mao, ZQ, Pan, LS, Ling, ZP, Liu, XY, Zhang, J, Yu, XG		NN			
Web Of Science	Functional and neurochemical disruptions of brain hub topology in chronic pain	2019	Kaplan, CM, Schrepf, A, Vatansever, D, Larkin, TE, Mawla, I, Ichesco, E, Kochlefl, L, Harte, SE, Clauw, DJ, Mashour, GA, Harris, RE		YY	Y	Y	Y
Web Of Science	Functional connectivity of music-induced analgesia in fibromyalgia	2019	Pando-Naude, V, Barrios, FA, Alcauter, S, Pasaye, EH, Vase, L, Brattico, E, Vuust, P, Garza-Villarreal, EA	Y				
Web Of Science	Hormonal Status and Cognitivo-Emotional Profile in Real-Life Patients With Neuropathic Pain: A Case Control Study	2019	Corriger, A, Duclos, M, Corcuff, JB, Lambert, C, Marceau, G, Sapin, V, Macian, N, Roux, D, Pereira, B, Pickering, G		NN			
Web Of Science	Impact of Fibromyalgia on Alpha-2 EEG Power Spectrum in the Resting Condition: A Descriptive Correlational Study	2019	Villafaina, S, Collado-Mateo, D, Fuentes-García, JP, Cano-Plasencia, R, Gusi, N	Y				
Web Of Science	Influence of pain anticipation on brain activity and pain perception in Gulf War Veterans with chronic musculoskeletal pain	2019	Lindheimer, JB, Stegner, AJ, Ellingson-Sayen, LD, Van Riper, SM, Dougherty, RJ, Falvo, MJ, Cook, DB	Y				
Web Of Science	Motor Cortex Function in Fibromyalgia: A Study by Functional Near-Infrared Spectroscopy	2019	Gentile, E, Ricci, K, Delussi, M, Brighina, F, de Tommaso, M	Y				

Web Of Science	NYX-2925, A Novel N-methyl-D-aspartate Receptor Modulator: A First-in-Human, Randomized, Double-blind Study of Safety and Pharmacokinetics in Adults	2019	Houck, DR, Sindelar, L, Sanabria, CR, Stanworth, SH, Krueger, M, Suh, M, Madsen, TM	Y	
Web Of Science	Neural correlates of cognitive dysfunction in fibromyalgia patients: Reduced brain electrical activity during the execution of a cognitive control task	2019	Samartin-Veiga, N, González-Villar, AJ, Carrillo-de-la-Peña, MT	Y	
Web Of Science	No evidence for altered plasma NGF and BDNF levels in fibromyalgia patients	2019	Baumeister, D, Eich, W, Saft, S, Geisel, O, Hellweg, R, Finn, A, Svensson, C, Tesarz, J	Y	
Web Of Science	Nocturnal Gamma-Hydroxybutyrate Reduces Cortisol-Awakening Response and Morning Kynurenine Pathway Metabolites in Healthy Volunteers	2019	Dornbierer, DA, Boxler, M, Voegel, CD, Stucky, B, Steuer, AE, Binz, TM, Baumgartner, MR, Baur, DM, Quednow, BB, Kraemer, T, Seifritz, E, Landolt, HP, Bosch, OG	Y	
Web Of Science	Pain Expressions and Inhibitory Control in Patients With Fibromyalgia: Behavioral and Neural Correlates	2019	Pidal-Miranda, M, González-Villar, AJ, Carrillo-de-la-Peña, MT	Y	
Web Of Science	Potency of descending pain modulatory system is linked with peripheral sensory dysfunction in fibromyalgia An exploratory study	2019	Brietzke, AP, Antunes, LC, Carvalho, F, Elkifury, J, Gasparin, A, Sanches, PRS, da Silva, DP, Dussán-Sarria, JA, Souza, A, Torres, TLD, Fregni, F, Caumo, W	Y	
Web Of Science	Prepulse inhibition of the blink reflex is abnormal in functional movement disorders	2019	Hanzlíková, Z, Kofler, M, Slovák, M, Vechetová, G, Fecíková, A, Kemlink, D, Sieger, T, Ruzicka, E, Valls-Solé, J, Edwards, MJ, Serranová, T		NN
Web Of Science	Quantitative assessment of nonpelvic pressure pain sensitivity in urologic chronic pelvic pain syndrome: a MAPP Research Network study	2019	Harte, SE, Scnrepf, A, Gallop, R, Kruger, GH, Lai, HHH, Sutcliffe, S, Halvorson, M, Ichesco, E, Naliboff, BD, Afari, N, Harris, RE, Farrar, JT, Tu, F, Landis, JR, Clauw, DJ		NN
Web Of Science	Reduced excitatory neurotransmitter levels in anterior insulae are associated with	2019	Bednarska, O, Icenhour, A, Tapper, S, Witt, ST, Tisell, A, Lundberg, P, Elsenbruch, S, Engström, M, Walter, S		NN

	abdominal pain in irritable bowel syndrome				
Web Of Science	Subliminal emotional pictures are capable of modulating early cerebral responses to pain in fibromyalgia	2019	Peláez, I, Ferrera, D, Barjola, P, Fernandes, R, Mercado, F	Y	
Web Of Science	Subtle changes of gray matter volume in fibromyalgia reflect chronic musculoskeletal pain rather than disease-specific effects	2019	Sundermann, B, Nayyeri, MD, Pfleiderer, B, Stahlberg, K, Jünke, L, Baie, L, Dieckmann, R, Liem, D, Happe, T, Burgmer, M	Y	
Web Of Science	UNALTERED LOW NERVE GROWTH FACTOR AND HIGH BRAIN-DERIVED NEUROTROPHIC FACTOR LEVELS IN PLASMA FROM PATIENTS WITH FIBROMYALGIA AFTER A 15-WEEK PROGRESSIVE RESISTANCE EXERCISE	2019	Jablochkova, A, Bäckryd, E, Kosek, E, Mannerkorpi, K, Ernberg, M, Gerdle, B, Ghafouri, B	Y	
Web Of Science	Whole-brain structural magnetic resonance imaging-based classification of primary dysmenorrhea in pain-free phase: a machine learning study	2019	Chen, T, Mu, JY, Xue, QW, Yang, L, Dun, WH, Zhang, M, Liu, JX		NN
Web Of Science	A Retrospective 2D Morphometric Analysis of Adult Female Chiari Type I Patients with Commonly Reported and Related Conditions	2018	Eppelheimer, MS, Houston, JR, Bapuraj, JR, Labuda, R, Loth, DM, Braun, AM, Allen, NJ, Pahlavian, SH, Biswas, D, Urbizu, A, Martin, BA, Maher, CO, Allen, PA, Loth, F	Y	
Web Of Science	Altered connectivity of the right anterior insula drives the pain connectome changes in chronic knee osteoarthritis	2018	Cottam, WJ, Iwabuchi, SJ, Drabek, MM, Reckziegel, D, Auer, DP		NN
Web Of Science	Altered theta oscillations in resting EEG of fibromyalgia syndrome patients	2018	Fallon, N, Chiu, Y, Nurmikko, T, Stancak, A	Y	
Web Of Science	Association between brain-derived neurotrophic factor gene polymorphisms and fibromyalgia in a Korean population: a multicenter study	2018	Park, DJ, Kim, SH, Nah, SS, Lee, JH, Kim, SK, Lee, YA, Hong, SJ, Kim, HS, Lee, HS, Kim, HA, Joungm, CI, Kim, SH, Lee, SS	Y	

Web Of Science	Association of Fine Motor Loss and Allodynia in Fibromyalgia: An fNIRS Study	2018	Eken, A, Gökçay, D, Yilmaz, C, Baskak, B, Baltaci, A, Kara, M	Y	
Web Of Science	Brain Dynamics and Temporal Summation of Pain Predicts Neuropathic Pain Relief from Ketamine Infusion	2018	Bosma, RL, Cheng, JC, Rogachov, A, Kim, JA, Hemington, KS, Osborne, NR, Raghavan, LV, Bhatia, A, Davis, KD		NN
Web Of Science	Brain gray matter alterations in Chinese patients with chronic knee osteoarthritis pain based on voxel-based morphometry	2018	Liao, X, Mao, CP, Wang, Y, Zhang, QF, Cao, DY, Seminowicz, DA, Zhang, M, Yang, XL		NN
Web Of Science	Catastrophizing Interferes with Cognitive Modulation of Pain in Women with Fibromyalgia	2018	Ellingson, LD, Stegner, AJ, Schwabacher, IJ, Lindheimer, JB, Cook, DB	Y	
Web Of Science	Cerebral Blood Flow Volume Using Color Duplex Sonography in Patients With Fibromyalgia Syndrome	2018	Kaya, A, Akgöl, G, Gülkesen, A, Poyraz, AK, Yildirim, T, Atmaca, M	Y	
Web Of Science	Cerebral Blood Flow and Heart Rate Variability in Chronic Fatigue Syndrome: A Randomized Cross-Over Study	2018	Malfliet, A, Pas, R, Brouns, R, De Win, J, Hatem, SM, Meeus, M, Ickmans, K, van Hooff, RJ, Nijs, J		NN
Web Of Science	Cerebral blood flow modulations during cognitive control in major depressive disorder	2018	Hoffmann, A, Montoro, CI, Del Paso, GAR, Duschek, S		NN
Web Of Science	Cold Water Pressor Test Differentially Modulates Functional Network Connectivity in Fibromyalgia Patients Compared with Healthy Controls	2018	Jarrahi, B, Martucci, KT, Nilakantan, AS, Mackey, S	Y	
Web Of Science	Design and Validation of an FPGA-Based Configurable Transcranial Doppler Neurofeedback System for Chronic Pain Patients	2018	Rey, B, Rodríguez, A, Lloréns-Bufort, E, Tembl, J, Muñoz, MA, Montoya, P, Herrero-Bosch, V, Monzo, JM	Y	
Web Of Science	Differential efficiency of transcutaneous electrical nerve stimulation in dominant versus nondominant hands in fibromyalgia: placebo-controlled functional near-infrared spectroscopy study	2018	Eken, A, Kara, M, Baskak, B, Baltaci, A, Gökçay, D	Y	

Web Of Science	Disrupted Resting State Network of Fibromyalgia in Theta frequency	2018	Choe, MK, Lim, M, Kim, JS, Lee, DS, Chung, CK	Y	
Web Of Science	Dopaminergic and serotonergic mechanisms in the modulation of pain: In vivo studies in human brain	2018	Martikainen, IK, Hagelberg, N, Jääskeläinen, SK, Hietala, J, Pertovaara, A	Y	
Web Of Science	Effect of distress on transient network dynamics and topological equilibrium in phantom sound perception	2018	Mohan, A, Alexandra, SJ, Johnson, CV, De Ridder, D, Vanneste, S	Y	
Web Of Science	Emotional Influences on Cognitive Processing in Fibromyalgia Patients With Different Depression Levels: An Event-related Potential Study	2018	Sitges, C, González-Roldán, AM, Duschek, S, Montoya, P		NN
Web Of Science	Home-Based Transcranial Direct Current Stimulation Device Development: An Updated Protocol Used at Home in Healthy Subjects and Fibromyalgia Patients	2018	Carvalho, F, Brietzke, AP, Gasparin, A, dos Santos, FP, Vercelino, R, Ballester, RF, Sanches, PRS, da Silva, DP, Torres, ILS, Fregni, F, Caumo, W	Y	
Web Of Science	Imaging of joints in systemic lupus erythematosus	2018	Tani, C, Carli, L, Stagnaro, C, Elefante, E, Signorini, V, Balestri, F, Delle Sedie, A, Mosca, M		NN
Web Of Science	Increased thalamic glutamate/glutamine levels in migraineurs	2018	Bathel, A, Schweizer, L, Stude, P, Glaubitz, B, Wulms, N, Delicel, S, Schmidt-Wilcke, T		NN
Web Of Science	Meta-analysis of cognitive performance in fibromyalgia	2018	Bell, T, Trost, Z, Buelow, MT, Clay, O, Younger, J, Moore, D, Crowe, M		NN
Web Of Science	Morphology of subcortical brain nuclei is associated with autonomic function in healthy humans	2018	Ruffle, JK, Coen, SJ, Giampietro, V, Williams, SCR, Apkarian, AV, Farmer, AD, Aziz, Q	Y	
Web Of Science	Novel Insights of Effects of Pregabalin on Neural Mechanisms of Intracortical Disinhibition in Physiopathology of Fibromyalgia: An Explanatory, Randomized, Double-Blind Crossover Study	2018	Deitos, A, Soldatelli, MD, Dussán-Sarria, JA, Souza, A, Torres, ILD, Fregni, F, Caumo, W	Y	

Web Of Science	Oculomotor Disturbances in Patients with Chronic Nonspecific Spinal Pain	2018	Ruscheweyh, R, Fritz, A, Eggert, T, Azad, SC, Straube, A		NN
Web Of Science	Perception of induced dyspnea in fibromyalgia and chronic fatigue syndrome	2018	Van Den Houte, M, Bogaerts, K, Van Diest, I, De Bie, J, Persoons, P, Van Oudenhove, L, Van den Bergh, O	Y	
Web Of Science	Posterior Insular GABA Levels Inversely Correlate with the Intensity of Experimental Mechanical Pain in Healthy Subjects	2018	Thiaucourt, M, Shabes, P, Schloss, N, Sack, M, Baumgärtner, U, Schmahl, C, Ende, G		NN
Web Of Science	Relationships between brain metabolite levels, functional connectivity, and negative mood in urologic chronic pelvic pain syndrome patients compared to controls: A MAPP research network study	2018	Harper, DE, Ichesco, E, Schrepf, A, Halvorson, M, Puiu, T, Clauw, DJ, Harris, RE, Harte, SE		NN
Web Of Science	Salivary glutamate is elevated in individuals with chronic migraine	2018	Nam, JH, Lee, HS, Kim, J, Kim, J, Chu, MK	Y	
Web Of Science	The relationship with restless legs syndrome, fibromyalgia, and depressive symptoms in migraine patients	2018	Uzun, ZA, Kurt, S, Unaldi, HK		NN
Web Of Science	The role of long-term physical exercise on performance and brain activation during the Stroop colour word task in fibromyalgia patients	2018	Martinsen, S, Flodin, P, Berrebi, J, Löfgren, M, Bileviciute-Ljungar, I, Mannerkorpi, K, Ingvar, M, Fransson, P, Kosek, E	Y	
Web Of Science	Yawning and cortisol levels in multiple sclerosis: Potential new diagnostic tool	2018	Thompson, SBN, Coleman, A, Williams, N		NN
Web Of Science	A study of brain metabolism in fibromyalgia by positron emission tomography	2017	Usui, C, Soma, T, Hatta, K, Aratani, S, Fujita, H, Nishioka, K, Machida, Y, Kuroiwa, Y, Nakajima, T, Nishioka, K	Y	
Web Of Science	ASSESSMENT OF INTRA- AND INTER-REGIONAL INTERRELATIONS BETWEEN GABA plus , GLX AND BOLD DURING PAIN PERCEPTION IN THE HUMAN BRAIN - A COMBINED 1H FMRS AND FMRI STUDY	2017	Cleve, M, Gussew, A, Wagner, G, Bär, KJ, Reichenbach, JR		NN

Web Of Science	Abnormal structure and functional connectivity of the anterior insula at pain-free periovulation is associated with perceived pain during menstruation	2017	Dun, WH, Yang, J, Yang, L, Ding, D, Ma, XY, Liang, FL, von Deneen, KM, Ma, SH, Xu, XL, Liu, JX, Zhang, M		NN
Web Of Science	Acute effects of physical exercise on the serum insulin-like growth factor system in women with fibromyalgia	2017	Mannerkorpi, K, Landin-Wilhelmsen, K, Larsson, A, Cider, Å, Arodell, O, Bjersing, JL		NN
Web Of Science	Altered brain structure and function associated with sensory and affective components of classic trigeminal neuralgia	2017	Wang, Y, Cao, DY, Remeniuk, B, Krimmel, S, Seminowicz, DA, Zhang, M		NN
Web Of Science	Altered cerebral blood flow velocity features in fibromyalgia patients in resting-state conditions	2017	Rodríguez, A, Tembl, J, Mesa-Gresa, P, Muñoz, MA, Montoya, P, Rey, B	Y	
Web Of Science	Biomarkers for Musculoskeletal Pain Conditions: Use of Brain Imaging and Machine Learning	2017	Boissoneault, J, Sevel, L, Letzen, J, Robinson, M, Staud, R	Y	
Web Of Science	Brain 18F-FDG PET Metabolic Abnormalities in Patients with Long-Lasting Macrophagic Myofascitis	2017	Van der Gucht, A, Sebaiti, MA, Guedj, E, Aouizerate, J, Yara, S, Gherardi, RK, Evangelista, E, Chalaye, J, Cottereau, AS, Verger, A, Bachoud-Levi, AC, Abulizi, M, Itti, E, Authier, FJ		NN
Web Of Science	Brain signature and functional impact of centralized pain: a multidisciplinary approach to the study of chronic pelvic pain (MAPP) network study	2017	Kutch, JJ, Ichesco, E, Hampson, JP, Labus, JS, Farmer, MA, Martucci, KT, Ness, TJ, Deutsch, G, Apkarian, AV, Mackey, SC, Klumpp, DJ, Schaeffer, AJ, Rodriguez, LV, Kreder, KJ, Buchwald, D, Andriole, GL, Lai, HH, Mullins, C, Kusek, JW, Landis, JR, Mayer, EA, Clemens, JQ, Clauw, DJ, Harris, RE	Y	
Web Of Science	Brain structural changes in patients with chronic myofascial pain	2017	Niddam, DM, Lee, SH, Su, YT, Chan, RC		NN
Web Of Science	Cerebral 18F-FDG PET in macrophagic myofasciitis: An individual SVM-based approach	2017	Blanc-Durand, P, Van Der Gucht, A, Guedj, E, Abulizi, M, Aoun-Sebaiti, M, Lerman, L, Verger, A, Authier, FJ, Itti, E		NN

Web Of Science	Cognitive Impairment in Patients with Chronic Neuropathic or Radicular Pain: An Interaction of Pain and Age	2017	Moriarty, O, Ruane, N, O'Gorman, D, Maharaj, CH, Mitchell, C, Sarma, KM, Finn, DP, McGuire, BE		NN
Web Of Science	Cortical Thickness and Functional Connectivity Abnormality in Chronic Headache and Low Back Pain Patients	2017	Yang, Q, Wang, ZW, Yang, LX, Xu, YH, Chen, LM		NN
Web Of Science	Decreased prefrontal brain activation during verbal fluency task in patients with somatoform pain disorder: An exploratory multi-channel near-infrared spectroscopy study	2017	Ren, XJ, Lu, JL, Liu, XM, Shen, CY, Zhang, XQ, Ma, XY, Sun, JJ, Sun, GX, Feng, K, Xu, B, Liu, PZ		NN
Web Of Science	Difference in Regional Brain Volume between Fibromyalgia Patients and Long-Term Meditators	2017	Fayed, N, Garcia-Marti, G, Sanz-Requena, R, Marti-Bonmati, L, Garcia-Campayo, J	Y	
Web Of Science	Electroencephalographic Evidence of Altered Top-Down Attentional Modulation in Fibromyalgia Patients During a Working Memory Task	2017	González-Villar, AJ, Pidal-Miranda, M, Arias, M, Rodríguez-Salgado, D, Carrillo-de-la-Peña, MT	Y	
Web Of Science	Elevations of ventricular lactate levels occur in both chronic fatigue syndrome and fibromyalgia	2017	Natelson, BH, Vu, D, Coplan, JD, Mao, XL, Blate, M, Kang, GX, Soto, E, Kapusuz, T, Shungu, DC	Y	
Web Of Science	Epigenetics insights into chronic pain: DNA hypomethylation in fibromyalgia-a controlled pilot-study	2017	de Andrade, DC, Maschietto, M, Galhardoni, R, Gouveia, G, Chile, T, Krepischi, ACV, Dale, CS, Brunoni, AR, Parravano, DC, Moscoso, ASC, Raicher, I, Kaziyama, HHS, Teixeira, MJ, Brentani, HP	Y	
Web Of Science	Features of Resting-State Electroencephalogram Theta Coherence in Somatic Symptom Disorder Compared With Major Depressive Disorder: A Pilot Study	2017	Ahn, J, Han, DH, Hong, JS, Min, KJ, Lee, YS, Hahm, BJ, Kim, SM		NN
Web Of Science	Functional MRI of the Reserpine-Induced Putative Rat Model of Fibromyalgia Reveals Discriminatory Patterns of	2017	Wells, JA, Shibata, S, Fujikawa, A, Takahashi, M, Saga, T, Aoki, I	Y	

	Functional Augmentation to Acute Nociceptive Stimuli				
Web Of Science	Histological Underpinnings of Grey Matter Changes in Fibromyalgia Investigated Using Multimodal Brain Imaging	2017	Pomares, FB, Funck, T, Feier, NA, Roy, S, Daigle-Martel, A, Ceko, M, Narayanan, S, Araujo, D, Thiel, A, Stikov, N, Fitzcharles, MA, Schweinhardt, P	Y	
Web Of Science	Investigating the BOLD Spectral Power of the Intrinsic Connectivity Networks in Fibromyalgia Patients: A Resting-state fMRI Study	2017	Jarrahi, B, Martucci, KT, Nilakantan, AS, Mackey, S	Y	
Web Of Science	Is there any somatosensory amplification in patients with irritable bowel syndrome?	2017	Tugal, Ö, Özenli, Y, Cengisiz, C, Topal, K, Tasdogan, B, Kara, B, Can, C		NN
Web Of Science	Lower Functional Connectivity of the Periaqueductal Gray Is Related to Negative Affect and Clinical Manifestations of Fibromyalgia	2017	Coulombe, MA, St Lawrence, K, Moulin, DE, Morley-Forster, P, Shokouhi, M, Nielson, WR, Davis, KD	Y	
Web Of Science	Negative mood influences default mode network functional connectivity in patients with chronic low back pain: implications for functional neuroimaging biomarkers	2017	Letzen, JE, Robinson, ME		NN
Web Of Science	Occipital Nerve Field Transcranial Direct Current Stimulation Normalizes Imbalance Between Pain Detecting and Pain Inhibitory Pathways in Fibromyalgia	2017	De Ridder, D, Vanneste, S	Y	
Web Of Science	Painful After-Sensations in Fibromyalgia are Linked to Catastrophizing and Differences in Brain Response in the Medial Temporal Lobe	2017	Schreiber, KL, Loggia, ML, Kim, J, Cahalan, CM, Napadow, V, Edwards, RR	Y	
Web Of Science	Reduced laser-evoked potential habituation detects abnormal central pain processing in painful radiculopathy patients	2017	Hüllemann, P, von der Brelie, C, Manthey, G, Düsterhöft, J, Helmers, AK, Synowitz, M, Baron, R	Y	
Web Of Science	Reduced volume of gray matter in patients with trigeminal neuralgia	2017	Li, M, Yan, JH, Li, SM, Wang, TY, Zhan, WF, Wen, H, Ma, XF, Zhang, Y, Tian, JZ, Jiang, GH		NN

Web Of Science	Regional brain functions in the resting state indicative of potential differences between depression and chronic pain	2017	Yoshino, A, Okamoto, Y, Doi, M, Otsuru, N, Okada, G, Takamura, M, Ichikawa, N, Yokoyama, S, Yamashita, H, Yamawaki, S		YN	N
Web Of Science	Resting state electrical brain activity and connectivity in fibromyalgia	2017	Vanneste, S, Ost, J, Van Havenbergh, T, De Ridder, D	Y		
Web Of Science	Spatial-temporal signature of resting-state BOLD signals in classic trigeminal neuralgia	2017	Wang, YP, Xu, CY, Zhai, LP, Lu, XD, Wu, XQ, Yi, YH, Liu, ZY, Guan, QB, Zhang, XL		NN	
Web Of Science	Structural Brain Imaging in People With Low Back Pain	2017	Mansour, ZM, Lepping, RJ, Honea, RA, Brooks, WM, Yeh, HW, Burns, JM, Sharma, NK		NN	
Web Of Science	Structural Co-Variance Patterns in Migraine: A Cross-Sectional Study Exploring the Role of the Hippocampus	2017	Chong, CD, Dumkrieger, GM, Schwedt, TJ		NN	
Web Of Science	Suggestions to Reduce Clinical Fibromyalgia Pain and Experimentally Induced Pain Produce Parallel Effects on Perceived Pain but Divergent Functional MRI-Based Brain Activity	2017	Derbyshire, SWG, Whalley, MG, Seah, STH, Oakley, DA	Y		
Web Of Science	Towards a neurophysiological signature for fibromyalgia	2017	López-Solà, M, Woo, CW, Pujol, J, Deus, J, Harrison, BJ, Monfort, J, Wager, TD	Y		
Web Of Science	rTMS of the prefrontal cortex has analgesic effects on neuropathic pain in subjects with spinal cord injury	2017	Nardone, R, Höller, Y, Langthaler, PB, Lochner, P, Golaszewski, S, Schwenker, K, Brigo, F, Trinka, E	Y		
Web Of Science	A possible neural mechanism for photosensitivity in chronic pain	2016	Martenson, ME, Halawa, OI, Tonsfeldt, KJ, Maxwell, CA, Hammack, N, Mist, SD, Pennesi, ME, Bennett, RM, Mauer, KM, Jones, KD, Heinricher, MM	Y		
Web Of Science	Abnormal resting state functional connectivity of the periaqueductal grey in patients with fibromyalgia	2016	Truini, A, Tinelli, E, Gerardi, MC, Calistri, V, Iannuccelli, C, La Cesa, S, Tarsitani, L, Mainero, C, Sarzi-Puttini, P, Cruccu, G, Caramia, F, Di Franco, M	Y		

Web Of Science	Altered fMRI resting-state connectivity in individuals with fibromyalgia on acute pain stimulation	2016	Ichesco, E, Puiu, T, Hampson, JP, Kairys, AE, Clauw, DJ, Harte, SE, Peltier, SJ, Harris, RE, Schmidt-Wilcke, T	Y	
Web Of Science	Augmented Pain Processing in Primary and Secondary Somatosensory Cortex in Fibromyalgia: A Magnetoencephalography Study Using Intra-Epidermal Electrical Stimulation	2016	Lim, M, Roosink, M, Kim, JS, Kim, HW, Lee, EB, Son, KM, Kim, HA, Chung, CK	Y	
Web Of Science	Brain activations during pain: a neuroimaging meta-analysis of patients with pain and healthy controls	2016	Jensen, KB, Regenbogen, C, Ohse, MC, Frasnelli, J, Freiherr, J, Lundström, JN		NN
Web Of Science	CEREBRAL VASOMOTOR REACTIVITY IN FIBROMYALGIA PATIENTS AND ITS RELATIONSHIP TO CENTRAL NEUROPATHIC PAIN	2016	Guler, S, Kurtoglu, HS, Kehaya, S, Pamuk, N, Çelik, Y	Y	
Web Of Science	Characterizing "fibrofog": Subjective appraisal, objective performance, and task-related brain activity during a working memory task	2016	Walitt, B, Ceko, M, Khatiwada, M, Gracely, JL, Rayhan, R, VanMeter, JW, Gracely, RH	Y	
Web Of Science	Cognitive performance in women with fibromyalgia: A case-control study	2016	de Heredia-Torres, MP, Huertas-Hoyas, E, Máximo-Bocanegra, N, Palacios-Ceña, D, Fernández-De-Las-Peñas, C		NN
Web Of Science	Current treatments to counter sleep dysfunction as a pathogenic stimulus of fibromyalgia	2016	Choy, EH		NN
Web Of Science	Endogenous opioidergic dysregulation of pain in fibromyalgia: a PET and fMRI study	2016	Schrepf, A, Harper, DE, Harte, SE, Wang, H, Ichesco, E, Hampson, JP, Zubieta, JK, Clauw, DJ, Harris, RE	Y	
Web Of Science	Evaluation of cytokines, oxidative stress markers and brain-derived neurotrophic factor in patients with fibromyalgia - A controlled cross-sectional study	2016	Ranzolin, A, Duarte, ALBP, Bredemeier, M, Neto, CAD, Ascoli, BM, Wollenhaupt-Aguiar, B, Kapczinski, F, Xavier, RM	Y	
Web Of Science	Executive function in fibromyalgia: Comparing	2016	Gelonch, O, Garolera, M, Valls, J, Rosselló, L, Pifarré, J	Y	

	subjective and objective measures				
Web Of Science	Fibromyalgia Is Correlated with Retinal Nerve Fiber Layer Thinning	2016	Garcia-Martin, E, Garcia-Campayo, J, Puebla-Guedea, M, Ascaso, FJ, Roca, M, Gutierrez-Ruiz, F, Vilades, E, Polo, V, Larrosa, JM, Pablo, LE, Satue, M	Y	
Web Of Science	Functional Connectivity with the Default Mode Network Is Altered in Fibromyalgia Patients	2016	Fallon, N, Chiu, Y, Nurmikko, T, Stancak, A	Y	
Web Of Science	Habituation deficit of auditory N100m in patients with fibromyalgia	2016	Choi, W, Lim, M, Kim, JS, Chung, CK	Y	
Web Of Science	Impaired psychomotor ability and attention in patients with persistent pain: a cross-sectional comparative study	2016	Gunnarsson, H, Grahn, B, Agerström, J		NN
Web Of Science	Increased Low- and High-Frequency Oscillatory Activity in the Prefrontal Cortex of Fibromyalgia Patients	2016	Lim, M, Kim, JS, Kim, DJ, Chung, CK	Y	
Web Of Science	Intrinsic functional connectivity of insular cortex and symptoms of sickness during acute experimental inflammation	2016	Lekander, M, Karshikoff, B, Johansson, E, Soop, A, Fransson, P, Lundström, JN, Andreasson, A, Ingvar, M, Petrovic, P, Axelsson, J, Nilsonne, G		NN
Web Of Science	Is the volume of the caudate nuclei associated with area of secondary hyperalgesia? - Protocol for a 3-Tesla MRI study of healthy volunteers	2016	Hansen, MS, Asghar, MS, Wetterslev, J, Pipper, CB, Mårtensson, JJ, Becerra, L, Christensen, A, Nybing, JD, Havsteen, I, Boesen, M, Dahl, JB		NN
Web Of Science	Lower Resting State Heart Rate Variability Relates to High Pain Catastrophizing in Patients with Chronic Whiplash-Associated Disorders and Healthy Controls	2016	Koenig, J, De Kooning, M, Bernardi, A, Williams, DP, Nijs, J, Thayer, JF, Daenen, L		NN
Web Of Science	MRI based classification of chronic fatigue, fibromyalgia patients and healthy controls using machine learning algorithms: a comparison study	2016	Sevel, L, Letzen, J, Boissoneault, J, O'Shea, A, Robinson, M, Staud, R	Y	

Web Of Science	Microstructural Abnormalities in Gray Matter of Patients with Postherpetic Neuralgia: A Diffusional Kurtosis Imaging Study	2016	Zhang, Y, Yu, T, Qin, BY, Li, Y, Song, GJ, Yu, BW		NN
Web Of Science	Motor Cortex Excitability and BDNF Levels in Chronic Musculoskeletal Pain According to Structural Pathology	2016	Caumo, W, Deitos, A, Carvalho, S, Leite, J, Carvalho, F, Dussán-Sarria, JA, Tarragó, MDL, Souza, A, Torres, ILD, Fregni, F	Y	
Web Of Science	Nonpainful wide-area compression inhibits experimental pain	2016	Honigman, L, Bar-Bachar, O, Yarnitsky, D, Sprecher, E, Granovsky, Y		NN
Web Of Science	Pharmacologic attenuation of cross-modal sensory augmentation within the chronic pain insula	2016	Harte, SE, Ichesco, E, Hampson, JP, Peltier, SJ, Schmidt-Wilcke, T, Clauw, DJ, Harris, RE	Y	
Web Of Science	Relationship between FokI polymorphism in the vitamin D receptor gene and fibromyalgia syndrome	2016	Marasli, E, Ozdolap, S, Sarikaya, S		NN
Web Of Science	Transcranial Direct Current Stimulation (tDCS) Targeting Left Dorsolateral Prefrontal Cortex Modulates Task-Induced Acute Pain in Healthy Volunteers	2016	Mariano, TY, van't Wout, M, Garnaat, SL, Rasmussen, SA, Greenberg, BD		NN
Web Of Science	White matter microstructure alterations in primary dysmenorrhea assessed by diffusion tensor imaging	2016	Liu, P, Wang, GL, Liu, YF, Yu, QB, Yang, F, Jin, LM, Sun, JB, Yang, XJ, Qin, W, Calhoun, VD		NN
Web Of Science	Aberrant Cerebral Blood Flow Responses During Cognition: Implications for the Understanding of Cognitive Deficits in Fibromyalgia	2015	Montoro, CI, Duschek, S, de Guevara, CL, Fernández-Serrano, MJ, del Paso, GAR	Y	
Web Of Science	Affective Modulation of Brain and Autonomic Responses in Patients With Fibromyalgia	2015	Rosselló, F, Muñoz, MA, Duschek, S, Montoya, P	Y	
Web Of Science	Altered Cortical Processing of Observed Pain in Patients With Fibromyalgia Syndrome	2015	Fallon, N, Li, XY, Chiu, Y, Nurmikko, T, Stancak, A	Y	
Web Of Science	Brain Alterations and Neurocognitive Dysfunction in Patients With Complex Regional Pain Syndrome	2015	Lee, DH, Lee, KJ, Cho, KIK, Noh, EC, Jang, JH, Kim, YC, Kang, DH		NN

Web Of Science	Cerebral Lateralization, Depression and Serum S100B Levels in Patients with Fibromyalgia	2015	Baygutalp, NK, Yilmaz, N, Baygutalp, F, Öztürk, N, Gül, HI, Polat, H, Bakan, E, Dane, S		NN
Web Of Science	Classification of Fibromyalgia Syndrome by Using NIRS Signals	2015	Eken, A, Gökçay, D, Topçu, C, Baltaci, A, Baskak, B, Kara, M		NN
Web Of Science	Cognitive effects and autonomic responses to transcranial pulsed current stimulation	2015	Morales-Quezada, L, Cosmo, C, Carvalho, S, Leite, J, Castillo-Saavedra, L, Rozisky, JR, Fregni, F		NN
Web Of Science	Comparison of Machine Classification Algorithms for Fibromyalgia: Neuroimages Versus Self-Report	2015	Robinson, ME, O'Shea, AM, Craggs, JG, Price, DD, Letzen, JE, Staud, R	Y	
Web Of Science	Deficient habituation to repeated rectal distensions in irritable bowel syndrome patients with visceral hypersensitivity	2015	Lowén, MBO, Mayer, E, Tillisch, K, Labus, J, Naliboff, B, Lundberg, P, Thorell, LH, Ström, M, Engström, M, Walter, S		NN
Web Of Science	Disinhibition of the primary somatosensory cortex in patients with fibromyalgia	2015	Lim, M, Roosink, M, Kim, JS, Kim, DJ, Kim, HW, Lee, EB, Kim, HA, Chung, CK	Y	
Web Of Science	Dynamic Interactions Between Plasma IL-I Family Cytokines and Central Endogenous Opioid Neurotransmitter Function in Humans	2015	Prossin, AR, Zalcman, SS, Heitzeg, MM, Koch, AE, Campbell, PL, Phan, KL, Stohler, CS, Zubieta, JK		NN
Web Of Science	Early menopause and other gynecologic risk indicators for chronic fatigue syndrome in women	2015	Boneva, RS, Lin, JMS, Unger, ER		NN
Web Of Science	Effect of Milnacipran Treatment on Ventricular Lactate in Fibromyalgia: A Randomized, Double-Blind, Placebo-Controlled Trial	2015	Natelson, BH, Vu, D, Mao, XL, Weiduschat, N, Togo, F, Lange, G, Blate, M, Kang, GX, Coplan, JD, Shungu, DC	Y	
Web Of Science	Evidence of different mediators of central inflammation in dysfunctional and inflammatory pain - Interleukin-8 in fibromyalgia and interleukin-1 β in rheumatoid arthritis	2015	Kosek, E, Altawil, R, Kadetoff, D, Finn, A, Westman, M, Le Maître, E, Andersson, M, Jensen-Urstad, M, Lampa, J		NN
Web Of Science	Fibromyalgia is characterized by altered frontal and cerebellar	2015	Kim, H, Kim, J, Loggia, ML, Cahalan, C, Garcia, RG,	Y	

	structural covariance brain networks		Vangel, MG, Wasan, AD, Edwards, RR, Napadow, V			
Web Of Science	Fibromyalgia patients have reduced hippocampal volume compared with healthy controls	2015	McCrae, CS, O'Shea, AM, Boissoneault, J, Vatthauer, KE, Robinson, ME, Staud, R, Perlstein, WM, Craggs, JG	Y		
Web Of Science	Imaging techniques and pain	2015	Maihöfner, C, Bingel, U		YN	N
Web Of Science	Impaired pre-attentive auditory processing in fibromyalgia: A mismatch negativity (MMN) study	2015	Choi, W, Lim, M, Kim, JS, Kim, DJ, Chung, CK	Y		
Web Of Science	Increased Brain Gray Matter in the Primary Somatosensory Cortex is Associated with Increased Pain and Mood Disturbance in Patients with Interstitial Cystitis/Painful Bladder Syndrome	2015	Kairys, AE, Schmidt-Wilcke, T, Puiu, T, Ichesco, E, Labus, JS, Martucci, K, Farmer, MA, Ness, TJ, Deutsch, G, Mayer, EA, Mackey, S, Apkarian, AV, Maravilla, K, Clauw, DJ, Harris, RE	Y		
Web Of Science	Increased cortical activation upon painful stimulation in fibromyalgia syndrome	2015	Üçeyler, N, Zeller, J, Kewenig, S, Kittel-Schneider, S, Fallgatter, AJ, Sommer, C	Y		
Web Of Science	Investigating neural mechanisms of change of cognitive behavioural therapy for chronic fatigue syndrome: a randomized controlled trial	2015	van Der Schaaf, ME, Schmits, IC, Roerink, M, Geurts, DEM, Toni, I, Roelofs, K, De Lange, FP, Nater, UM, van der Meer, JWM, Knoop, H		NN	
Web Of Science	Investigation of Central Nervous System Dysfunction in Chronic Pelvic Pain Using Magnetic Resonance Spectroscopy and Noninvasive Brain Stimulation	2015	Simis, M, Reidler, JS, Macea, DD, Duarte, IM, Wang, XE, Lenkinski, R, Petrozza, JC, Fregni, F		NN	
Web Of Science	Normalization of aberrant resting state functional connectivity in fibromyalgia patients following a three month physical exercise therapy	2015	Flodin, P, Martinsen, S, Mannerkorpi, K, Löfgren, M, Bileviciute-Ljungar, I, Kosek, E, Fransson, P	Y		
Web Of Science	Reaction time, cerebral blood flow, and heart rate responses in fibromyalgia: Evidence of alterations in attentional control	2015	del Paso, GAR, Montoro, CI, Duschek, S	Y		
Web Of Science	Self-perspective leads to increased activation of pain processing brain regions in fibromyalgia	2015	Rahm, B, Lacour, M, Decety, J, Müller, J, Scheidt, CE, Bauer, J, König, R, Wirsching, M, Glauche, V, Ohlendorf, S,	Y		

			Unterbrink, T, Hartmann, A, Joos, AA		
Web Of Science	Significance of Non-phase Locked Oscillatory Brain Activity in Response to Noxious Stimuli	2015	Rouleau, RD, Lagrandeur, L, Daigle, K, Lorrain, D, Léonard, G, Whittingstall, K, Goffaux, P		NN
Web Of Science	The Somatosensory Link in Fibromyalgia Functional Connectivity of the Primary Somatosensory Cortex Is Altered by Sustained Pain and Is Associated With Clinical/Autonomic Dysfunction	2015	Kim, J, Loggia, ML, Cahalan, CM, Harris, RE, Beissner, F, Garcia, RG, Kim, H, Barbieri, R, Wasan, AD, Edwards, RR, Napadow, V	Y	
Web Of Science	Alterations in excitatory and inhibitory brainstem interneuronal circuits in fibromyalgia: Evidence of brainstem dysfunction	2014	Kofler, M, Halder, W	Y	
Web Of Science	Altered Resting State Connectivity of the Insular Cortex in Individuals With Fibromyalgia	2014	Ichesco, E, Schmidt-Wilcke, T, Bhavsar, R, Clauw, DJ, Peltier, SJ, Kim, J, Napadow, V, Hampson, JP, Kairys, AE, Williams, DA, Harris, RE	Y	
Web Of Science	Altered White Matter Integrity in the Corpus Callosum in Fibromyalgia Patients Identified by Tract-Based Spatial Statistical Analysis	2014	Kim, DJ, Lim, M, Kim, JS, Son, KM, Kim, HA, Chung, CK	Y	
Web Of Science	Basal μ-opioid receptor availability in the amygdala predicts the inhibition of pain-related brain activity during heterotopic noxious counter-stimulation	2014	Piché, M, Watanabe, N, Sakata, M, Oda, K, Toyohara, J, Ishii, K, Ishiwata, K, Hotta, H		NN
Web Of Science	Central neural mechanisms of interindividual difference in discomfort during sensorimotor incongruence in healthy volunteers: an experimental study	2014	Nishigami, T, Nakano, H, Osumi, M, Tsujishita, M, Mibu, A, Ushida, T		NN
Web Of Science	Changes in Clinical Pain in Fibromyalgia Patients Correlate with Changes in Brain Activation in the Cingulate Cortex in a Response Inhibition Task	2014	Schmidt-Wilcke, T, Kairys, A, Ichesco, E, Fernandez-Sanchez, ML, Barjola, P, Heitzeg, M, Harris, RE, Clauw, DJ, Glass, J, Williams, DA	Y	
Web Of Science	Classification of brain activity response to painful heat stimuli in	2014	Borja, M, Martucci, K, Nilakantan, A, Mackey, S	Y	

	fibromyalgia patients and healthy controls				
Web Of Science	Decreased activation of cingulo-frontal-parietal cognitive/attention network during an attention-demanding task in patients with chronic low back pain	2014	Mao, CP, Zhang, QL, Bao, FX, Liao, X, Yang, XL, Zhang, M		NN
Web Of Science	Disrupted Brain Circuitry for Pain-Related Reward/Punishment in Fibromyalgia	2014	Loggia, ML, Berna, C, Kim, J, Cahalan, CM, Gollub, RL, Wasan, AD, Harris, RE, Edwards, RR, Napadow, V	Y	
Web Of Science	Fibromyalgia Is Associated with Decreased Connectivity Between Pain- and Sensorimotor Brain Areas	2014	Flodin, P, Martinsen, S, Löfgren, M, Bileviciute-Ljungar, I, Kosek, E, Fransson, P	Y	
Web Of Science	Fibromyalgia Patients Had Normal Distraction Related Pain Inhibition but Cognitive Impairment Reflected in Caudate Nucleus and Hippocampus during the Stroop Color Word Test	2014	Martinsen, S, Flodin, P, Berrebi, J, Löfgren, M, Bileviciute-Ljungar, I, Ingvar, M, Fransson, P, Kosek, E	Y	
Web Of Science	Higher Glutamate plus Glutamine and Reduction of N-acetylaspartate in Posterior Cingulate According to Age Range in Patients with Cognitive Impairment and/or Pain	2014	Fayed, N, Andrés, E, Viguera, L, Modrego, PJ, Garcia-Campayo, J	Y	
Web Of Science	Impaired modulation of pain in patients with postherpetic neuralgia	2014	Pickering, G, Pereira, B, Dufour, E, Soule, S, Dubray, C		NN
Web Of Science	Is number sense impaired in chronic pain patients?	2014	Wolrich, J, Poots, AJ, Kuehler, BM, Rice, ASC, Rahman, A, Bantel, C		NN
Web Of Science	Preliminary structural MRI based brain classification of chronic pelvic pain: A MAPP network study	2014	Bagarinao, E, Johnson, KA, Martucci, KT, Ichesco, E, Farmer, MA, Labus, J, Ness, TJ, Harris, R, Deutsch, G, Apkarian, AV, Mayer, EA, Clauw, DJ, Mackey, S		NN
Web Of Science	Regional Neuroplastic Brain Changes in Patients with Chronic Inflammatory and Non-Inflammatory Visceral Pain	2014	Hong, JY, Labus, JS, Jiang, ZG, Ashe-Mcnalley, C, Dinov, I, Gupta, A, Shi, YG, Stains, J, Heendeniya, N, Smith, SR, Tillisch, K, Mayer, EA		NN

Web Of Science	The MAPP research network: design, patient characterization and operations	2014	Landis, JR, Williams, DA, Lucia, MS, Clauw, DJ, Naliboff, BD, Robinson, NA, van Bokhoven, A, Sutcliffe, S, Schaeffer, AJ, Rodriguez, LV, Mayer, EA, Lai, HH, Krieger, JN, Kreder, KJ, Afari, N, Andriole, GL, Bradley, CS, Griffith, JW, Klumpp, DJ, Hong, BA, Lutgendorf, SK, Buchwald, D, Yang, CC, Mackey, S, Pontari, MA, Hanno, P, Kusek, JW, Mullins, C, Clemens, JQ	Y		
Web Of Science	White Matter Involvement in Chronic Musculoskeletal Pain	2014	Lieberman, G, Shpaner, M, Watts, R, Andrews, T, Filippi, CG, Davis, M, Naylor, MR		YN	N
Web Of Science	fMRI pain activation in the periaqueductal gray in healthy volunteers cold pressor test	2014	La Cesa, S, Tinelli, E, Toschi, N, Di Stefano, G, Collorone, S, Aceti, A, Francia, A, Cruccu, G, Truini, A, Caramia, F	Y		
Web Of Science	A Chronic Fatigue Syndrome (CFS) severity score based on case designation criteria	2013	Baraniuk, JN, Adewuyi, O, Merck, SJ, Ali, M, Ravindran, MK, Timbol, CR, Ray-Han, R, Zheng, Y, Le, U, Esteitie, R, Petrie, KN		NN	
Web Of Science	Alterations in Endogenous Opioid Functional Measures in Chronic Back Pain	2013	Martikainen, IK, Peciña, M, Love, TM, Nuechterlein, EB, Cummiford, CM, Green, CR, Harris, RE, Stohler, CS, Zubieta, JK	Y		
Web Of Science	Augmented Central Pain Processing in Vulvodynia	2013	Hampson, JP, Reed, BD, Clauw, DJ, Bhavsar, R, Gracely, RH, Haefner, HK, Harris, RE	Y		
Web Of Science	Brain correlates of cognitive inhibition in fibromyalgia: Emotional intrusion of symptom-related words	2013	Mercado, F, González, JL, Barjola, P, Fernández-Sánchez, M, López-López, A, Alonso, M, Gómez-Esquer, F	Y		
Web Of Science	Cortical and white matter alterations in patients with neuropathic pain after spinal cord injury	2013	Yoon, EJ, Kim, YK, Shin, HI, Lee, Y, Kim, SE		NN	
Web Of Science	Deficient modulation of pain by a positive emotional context in fibromyalgia patients	2013	Kamping, S, Bomba, IC, Kanske, P, Diesch, E, Flor, H	Y		

Web Of Science	Do patients with fibromyalgia show abnormal neural responses to the observation of pain in others?	2013	Lee, SJ, Song, HJ, Decety, J, Seo, J, Kim, SH, Kim, SH, Nam, EJ, Kim, SK, Han, SW, Lee, HJ, Do, Y, Chang, Y	Y		
Web Of Science	Emotional modulation of muscle pain is associated with polymorphisms in the serotonin transporter gene	2013	Horjales-Araujo, E, Demontis, D, Lund, EK, Vase, L, Finnerup, NB, Borglum, AD, Jensen, TS, Svensson, P		NN	
Web Of Science	Evaluation of the Effectiveness of Pregabalin in Alleviating Pain Associated with Fibromyalgia: Using Functional Magnetic Resonance Imaging Study	2013	Kim, SH, Lee, Y, Lee, S, Mun, CW	Y		
Web Of Science	Experimental Placebo Analgesia Changes Resting-State Alpha Oscillations	2013	Huneke, NTM, Brown, CA, Burford, E, Watson, A, Trujillo-Barreto, NJ, El-Deredy, W, Jones, AKP		NN	
Web Of Science	Fear-learning deficits in subjects with fibromyalgia syndrome?	2013	Jenewein, J, Moergeli, H, Sprott, H, Honegger, D, Brunner, L, Ettlin, D, Grillon, C, Bloch, K, Brügger, M, Schwegler, K, Schumacher, S, Hasler, G		NN	
Web Of Science	Fibromyalgia interacts with age to change the brain	2013	Ceko, M, Bushnell, MC, Fitzcharles, MA, Schweinhardt, P	Y		
Web Of Science	Introducing the Event Related Fixed Interval Area (ERFIA) Multilevel Technique: a Method to Analyze the Complete Epoch of Event-Related Potentials at Single Trial Level	2013	Vossen, CJ, Vossen, HGM, Marcus, MAE, van Os, J, Lousberg, R		NN	
Web Of Science	Ipsilateral cortical activation in fibromyalgia patients during brushing correlates with symptom severity	2013	Fallon, N, Chiu, YH, Li, XY, Nurmikko, TJ, Stancak, A	Y		
Web Of Science	Muscle fatigue in fibromyalgia is in the brain, not in the muscles: a case-control study of perceived versus objective muscle fatigue	2013	Bandak, E, Amris, K, Bliddal, H, Danneskiold-Samsoe, B, Henriksen, M	Y		
Web Of Science	One night of total sleep deprivation promotes a state of generalized hyperalgesia: A surrogate pain model to study the relationship of insomnia and pain	2013	Schuh-Hofer, S, Wodarski, R, Pfau, DB, Caspani, O, Magerl, W, Kennedy, JD, Treede, RD		NN	

Web Of Science	Overlapping Structural and Functional Brain Changes in Patients With Long-Term Exposure to Fibromyalgia Pain	2013	Jensen, KB, Srinivasan, P, Spaeth, R, Tan, Y, Kosek, E, Petzke, F, Carville, S, Fransson, P, Marcus, H, Williams, SCR, Choy, E, Vitton, O, Gracely, R, Ingvar, M, Kong, J	Y	
Web Of Science	Structural alterations in brainstem of fibromyalgia syndrome patients correlate with sensitivity to mechanical pressure	2013	Fallon, N, Alghamdi, J, Chiu, Y, Sluming, V, Nurmikko, T, Stancak, A	Y	
Web Of Science	Altered Excitation-inhibition Balance in the Brain of Patients with Diabetic Neuropathy	2012	Petrou, M, Pop-Busui, R, Foerster, BR, Edden, RA, Callaghan, BC, Harte, SE, Harris, RE, Clauw, DJ, Feldman, EL		NN
Web Of Science	Behavioral and Neuronal Investigations of Hypervigilance in Patients with Fibromyalgia Syndrome	2012	Tiemann, L, Schulz, E, Winkelmann, A, Ronel, J, Henningsen, P, Ploner, M	Y	
Web Of Science	Brain dysfunction in fibromyalgia and somatization disorder using proton magnetic resonance spectroscopy: a controlled study	2012	Fayed, N, Andres, E, Rojas, G, Moreno, S, Serrano-Blanco, A, Roca, M, Garcia-Campayo, J	Y	
Web Of Science	Cerebral Blood Flow Dynamics During Pain Processing Investigated by Functional Transcranial Doppler Sonography	2012	Duschek, S, Hellmann, N, Merzoug, K, del Paso, GAR, Werner, NS		NN
Web Of Science	Cerebral mechanisms of experimental hyperalgesia in fibromyalgia	2012	Burgmer, M, Pfleiderer, B, Maihöfner, C, Gaubitz, M, Wessolleck, E, Heuft, G, Pogatzki-Zahn, E	Y	
Web Of Science	Changes in regional gray matter volume in women with chronic pelvic pain: A voxel-based morphometry study	2012	As-Sanie, S, Harris, RE, Napadow, V, Kim, J, Neshewat, G, Kairys, A, Williams, D, Clauw, DJ, Schmidt-Wilcke, T		NN
Web Of Science	Cognitive impairment in fibromyalgia syndrome: The impact of cardiovascular regulation, pain, emotional disorders and medication	2012	del Paso, GAR, Pulgar, A, Duschek, S, Garrido, S		NN
Web Of Science	Disrupted Functional Connectivity of the Pain Network in Fibromyalgia	2012	Cifre, I, Sitges, C, Fraiman, D, Muñoz, MA, Balenzuela, P, González-Roldán, A, Martínez-	Y	

			Jauand, M, Birbaumer, N, Chialvo, DR, Montoya, P		
Web Of Science	Effective Connectivity Among Brain Regions Associated With Slow Temporal Summation of C-Fiber-Evoked Pain in Fibromyalgia Patients and Healthy Controls	2012	Craggs, JG, Staud, R, Robinson, ME, Perlstein, WM, Price, DD	Y	
Web Of Science	Effects of Motor Cortex Modulation and Descending Inhibitory Systems on Pain Thresholds in Healthy Subjects	2012	Reidler, JS, Mendonca, ME, Santana, MB, Wang, XE, Lenkinski, R, Motta, AF, Marchand, S, Latif, L, Fregni, F		NN
Web Of Science	Intrinsic Brain Network Abnormalities in Migraines without Aura Revealed in Resting-State fMRI	2012	Xue, T, Yuan, K, Zhao, L, Yu, DH, Zhao, LM, Dong, T, Cheng, P, von Deneen, KM, Qin, W, Tian, J		NN
Web Of Science	Oxidative Stress Correlates with Headache Symptoms in Fibromyalgia: Coenzyme Q10 Effect on Clinical Improvement	2012	Cordero, MD, Cano-García, FJ, Alcocer-Gómez, E, De Miguel, M, Sánchez-Alcázar, JA		NN
Web Of Science	Patients with fibromyalgia display less functional connectivity in the brain's pain inhibitory network	2012	Jensen, KB, Loitoile, R, Kosek, E, Petzke, F, Carville, S, Fransson, P, Marcus, H, Williams, SCR, Choy, E, Mainguy, Y, Vitton, O, Gracely, RH, Gollub, R, Ingvar, M, Kong, J	Y	
Web Of Science	Self-ratings of higher olfactory acuity contrast with reduced olfactory test results of fibromyalgia patients	2012	Lötsch, J, Kraetsch, HG, Wendler, J, Hummel, T	Y	
Web Of Science	Structural changes of the brain in rheumatoid arthritis	2012	Wartolowska, K, Hough, MG, Jenkinson, M, Andersson, J, Wordsworth, BP, Tracey, I		NN
Web Of Science	The dopaminergic system in patients with functional dyspepsia analysed by single photon emission computed tomography (SPECT) and an alpha-methyl-para-tyrosine (AMPT) challenge test	2012	Braak, B, Booij, J, Klooker, TK, van den Wijngaard, RMJ, Boeckxstaens, GEE		NN
Web Of Science	Transcranial direct current stimulation does neither modulate results of a quantitative sensory testing protocol nor ratings of	2012	Jürgens, TP, Schulte, A, Klein, T, May, A	Y	

	suprathreshold heat stimuli in healthy volunteers				
Web Of Science	Working Memory Impairment in Fibromyalgia Patients Associated with Altered Frontoparietal Memory Network	2012	Seo, J, Kim, SH, Kim, YT, Song, HJ, Lee, JJ, Kim, SH, Han, SW, Nam, EJ, Kim, SK, Lee, HJ, Lee, SJ, Chang, Y	Y	
Web Of Science	Altered associative learning and emotional decision making in fibromyalgia	2011	Walteros, C, Sánchez-Navarro, JP, Muñoz, MA, Martínez-Selva, JM, Chialvo, D, Montoya, P		NN
Web Of Science	Brain Morphological Signatures for Chronic Pain	2011	Baliki, MN, Schnitzer, TJ, Bauer, WR, Apkarian, AV		NN
Web Of Science	CENTRAL MECHANISMS DURING FATIGUING MUSCLE EXERCISE IN MUSCULAR DYSTROPHY AND FIBROMYALGIA SYNDROME: A STUDY WITH TRANSCRANIAL MAGNETIC STIMULATION	2011	Schwenkreis, P, Voigt, M, Hasenbring, M, Tegenthoff, M, Vorgerd, M, Kley, RA	Y	
Web Of Science	Cerebral Activation and Catastrophizing During Pain Anticipation in Patients With Fibromyalgia	2011	Burgmer, M, Petzke, F, Giesecke, T, Gaubitz, M, Heuft, G, Pfleiderer, B	Y	
Web Of Science	Cerebral Blood Flow Alterations in Pain-Processing Regions of Patients with Fibromyalgia Using Perfusion MR Imaging	2011	Foerster, BR, Petrou, M, Harris, RE, Barker, PB, Hoeffner, EG, Clauw, DJ, Sundgren, PC	Y	
Web Of Science	Changes in Regional Gray and White Matter Volume in Patients with Myofascial-type Temporomandibular Disorders: A Voxel-based Morphometry Study	2011	Gerstner, G, Ichesco, E, Quintero, A, Schmidt-Wilcke, T		NN
Web Of Science	Different Pain, Different Brain: Thalamic Anatomy in Neuropathic and Non-Neuropathic Chronic Pain Syndromes	2011	Gustin, SM, Peck, CC, Wilcox, SL, Nash, PG, Murray, GM, Henderson, LA		NN
Web Of Science	Differential central pain processing following repetitive intramuscular proton/prostaglandin E2 injections in female fibromyalgia patients and healthy controls	2011	Diers, M, Schley, MT, Rance, M, Yilmaz, P, Lauer, L, Rukwied, R, Schmelz, M, Flor, H	Y	
Web Of Science	Effective Treatment of Chronic Low Back Pain in Humans	2011	Seminowicz, DA, Wideman, TH, Naso, L, Hatami-Khoroushahi, Z, Fallatah, S,		NN

	Reverses Abnormal Brain Anatomy and Function		Ware, MA, Jarzem, P, Bushnell, MC, Shir, Y, Ouellet, JA, Stone, LS		
Web Of Science	Executive Function in Chronic Pain Patients and Healthy Controls: Different Cortical Activation During Response Inhibition in Fibromyalgia	2011	Glass, JM, Williams, DA, Fernandez-Sanchez, ML, Kairys, A, Barjola, P, Heitzeg, MM, Clauw, DJ, Schmidt-Wilcke, T	Y	
Web Of Science	Feasibility, safety, and effectiveness of transcranial direct current stimulation for decreasing post-ERCP pain: a randomized, sham-controlled, pilot study	2011	Borckardt, JJ, Romagnuolo, J, Reeves, ST, Madan, A, Frohman, H, Beam, W, George, MS		NN
Web Of Science	Gray Matter Volumes of Pain-Related Brain Areas Are Decreased in Fibromyalgia Syndrome	2011	Robinson, ME, Craggs, JG, Price, DD, Perlstein, WM, Staud, R	Y	
Web Of Science	Hypothalamic Gray Matter Volume Loss in Hypnic Headache	2011	Holle, D, Naegel, S, Krebs, S, Gaul, C, Gizewski, E, Diener, HC, Katsarava, Z, Obermann, M		NN
Web Of Science	Insula-specific responses induced by dental pain. A proton magnetic resonance spectroscopy study	2011	Gutzeit, A, Meier, D, Meier, ML, von Weymarn, C, Ettlin, DA, Graf, N, Froehlich, JM, Binkert, CA, Brügger, M		NN
Web Of Science	Neuroimaging of fibromyalgia	2011	Gracely, RH, Ambrose, KR		NN
Web Of Science	Preattentive Processing Abnormalities in Chronic Pain: Neurophysiological Evidence from Mismatch Negativity	2011	Yao, SQ, Liu, XH, Yang, WH, Wang, X		NN
Web Of Science	Sleep architecture in patients with fibromyalgia	2011	González, JLB, Fernández, TVS, Rodríguez, LA, Muñiz, J, Giráldez, SL, Fernández, AA	Y	
Web Of Science	The Cortical Rhythms of Chronic Back Pain	2011	Baliki, MN, Baria, AT, Apkarian, AV		NN
Web Of Science	Pain inhibits pain' mechanisms: Is pain modulation simply due to distraction?	2010	Moont, R, Pud, D, Sprecher, E, Sharvit, G, Yarnitsky, D		NN
Web Of Science	Aberrances in Autonomic Cardiovascular Regulation in Fibromyalgia Syndrome and Their Relevance for Clinical Pain Reports	2010	del Paso, GAR, Garrido, S, Pulgar, A, Martín-Vazquez, M, Duschek, S		NN

Web Of Science	Attentional modulation fails to attenuate the subjective pain experience in chronic, unexplained pain	2010	Snijders, TJ, Ramsey, NF, Koerselman, F, van Gijn, J		NN	
Web Of Science	CNS processing of pain in functional somatic syndromes	2010	Petzke, F		YN	N
Web Of Science	Chronic myofascial temporomandibular pain is associated with neural abnormalities in the trigeminal and limbic systems	2010	Younger, JW, Shen, YF, Goddard, G, Mackey, SC		NN	
Web Of Science	Depression, anxiety, health-related quality of life and pain in patients with chronic. bromyalgia and neuropathic pain	2010	Gormsen, L, Rosenberg, R, Bach, FW, Jensen, TS	Y		
Web Of Science	Differential Effects of Painful and Non-Painful Stimulation on Tactile Processing in Fibromyalgia Syndrome and Subjects with Masochistic Behaviour	2010	Pollok, B, Krause, V, Legrain, V, Ploner, M, Freynhagen, R, Melchior, I, Schnitzler, A	Y		
Web Of Science	Dynamic assessment of the right lateral frontal cortex response to painful stimulation	2010	López-Solà, M, Pujol, J, Hernández-Ribas, R, Harrison, BJ, Ortiz, H, Soriano-Mas, C, Deus, J, Menchón, JM, Vallejo, J, Cardoner, N		NN	
Web Of Science	Increased Glutamate/Glutamine Compounds in the Brains of Patients With Fibromyalgia A Magnetic Resonance Spectroscopy Study	2010	Valdés, M, Collado, A, Bargalló, N, Vázquez, M, Rami, L, Gómez, E, Salamero, M	Y		
Web Of Science	Increased Sympathetic Activity Assessed by Spectral Analysis of Heart Rate Variability in Patients with CRPS I	2010	Schulze, J, Troeger, C		NN	
Web Of Science	Intrinsic Brain Connectivity in Fibromyalgia Is Associated With Chronic Pain Intensity	2010	Napadow, V, LaCount, L, Park, K, As-Sanie, S, Clauw, DJ, Harris, RE	Y		
Web Of Science	Linear and nonlinear analyses of EEG dynamics during non-painful somatosensory processing in chronic pain patients	2010	Sitges, C, Bornas, X, Llabrés, J, Noguera, M, Montoya, P		NN	
Web Of Science	Localized 1H-NMR spectroscopy in patients with fibromyalgia: a controlled study of changes in	2010	Fayed, N, Garcia-Campayo, J, Magallón, R, Andrés-	Y		

	cerebral glutamate/glutamine, inositol, choline, and N-acetylaspartate		Bergareche, H, Luciano, JV, Andres, E, Beltrán, J		
Web Of Science	MMPI-2 PROFILES: FIBROMYALGIA PATIENTS COMPARED TO EPILEPTIC AND NON-EPILEPTIC SEIZURE PATIENTS	2010	Johnson, AL, Storzbach, D, Binder, LM, Barkhuizen, A, Anger, WK, Salinsky, MC, Tun, SM, Rohlman, DS		NN
Web Of Science	Motor cortex rTMS reduces acute pain provoked by laser stimulation in patients with chronic neuropathic pain	2010	Lefaucheur, JP, Jarry, G, Drouot, X, Ménard-Lefaucheur, I, Keravel, Y, Nguyen, JP		NN
Web Of Science	Neuroimaging in Fibromyalgia Syndrome	2010	Wood, PB		NN
Web Of Science	Quantitative Electroencephalographic Abnormalities in Fibromyalgia Patients	2010	Hargrove, JB, Bennett, RM, Simons, DG, Smith, SJ, Nagpal, S, Deering, DE	Y	
Web Of Science	Rheumatologic Conditions: Sjogren's Syndrome, Fibromyalgia, and Chronic Fatigue Syndrome	2010	Glass, JM		NN
Web Of Science	Sensitisation of spinal cord pain processing in medication overuse headache involves supraspinal pain control	2010	Perrotta, A, Serrao, M, Sandrini, G, Burstein, R, Sances, G, Rossi, P, Bartolo, M, Pierelli, F, Nappi, G		NN
Web Of Science	The Anti-Fatigue Effect of Moderate Cooling: The Evidence, Physiological Mechanisms, and Possible Implications for the Prevention or Treatment of CFS	2010	Shevchuk, NA		NN
BVS	Assessment of retinal nerve fiber thickness and optic nerve head blood flow in female patients diagnosed with fibromyalgia syndrome	2022	Urfalıoglu, Selma, Berk, Ejder	Y	
BVS	Síndrome dolorosa disfuncional em doentes com sensibilidade exteroceptiva assimétrica: caracterização de uma entidade clínica	2014	Kaziyama, Helena Hideko Seguchi		NN
BVS	Brain structural differences between fibromyalgia patients and healthy control subjects: a	2025	Agoalikum, Elijah, Wu, Hongzhou, Klugah-Brown, Benjamin, Maes, Michael	Y	

	source-based morphometric study				
BVS	Altered Functional Networks during Gain Anticipation in Fibromyalgia	2023	Park, Su Hyoun, Michael, Andrew M, Baker, Anne K, Lei, Carina, Martucci, Katherine T	Y	
BVS	Abnormal Visual Evoked Responses to Emotional Cues Correspond to Diagnosis and Disease Severity in Fibromyalgia	2022	Goldway, Noam, Petro, Nathan M, Ablin, Jacob, Keil, Andreas, Ben Simon, Eti, Zamir, Yoav, Weizman, Libat, Greental, Ayam, Hendler, Talma, Sharon, Haggai	Y	
BVS	Altered Subprocesses of Working Memory in Patients with Fibromyalgia: An Event-Related Potential Study Using N-Back Task	2022	Mercado, Francisco, Ferrera, David, Fernandes-Magalhaes, Roberto, Peláez, Irene, Barjola, Paloma	Y	
BVS	Altered resting-state functional connectivity within corticostriatal and subcortical-striatal circuits in chronic pain	2022	Park, Su Hyoun, Baker, Anne K, Krishna, Vinit, Mackey, Sean C, Martucci, Katherine T	Y	
BVS	Assessment of retinal nerve fiber thickness and optic nerve head blood flow in female patients diagnosed with fibromyalgia syndrome	2022	Urfalıoglu, Selma, Berk, Ejder	Y	
BVS	Brain morphometric changes in patients with fibromyalgia	2022	Karayol, Kudret Cem, Karayol, Sunay Sibel	Y	
BVS	Electrophysiological indices of pain expectation abnormalities in fibromyalgia patients	2022	Barjola, Paloma, Peláez, Irene, Ferrera, David, González-Gutiérrez, José Luis, Velasco, Lilian, Peñacoba-Puente, Cecilia, López-López, Almudena, Fernandes-Magalhaes, Roberto, Mercado, Francisco	Y	
BVS	Neural correlates of the attentional bias towards pain-related faces in fibromyalgia patients: An ERP study using a dot-probe task	2022	Fernandes-Magalhaes, Roberto, Ferrera, David, Peláez, Irene, Martín-Buro, María Carmen, Carpio, Alberto, De Lahoz, María Eugenia, Barjola, Paloma, Mercado, Francisco	Y	
BVS	Plasma metabolomics reveals disrupted response and recovery following maximal exercise in	2022	Germain, Arnaud, Giloteaux, Ludovic, Moore, Geoffrey E, Levine, Susan M, Chia, John K,		NN

	myalgic encephalomyelitis/chronic fatigue syndrome		Keller, Betsy A, Stevens, Jared, Franconi, Carl J, Mao, Xiangling, Shungu, Dikoma C, Grimson, Andrew, Hanson, Maureen R		
BVS	The translocator protein gene is associated with endogenous pain modulation and the balance between glutamate and Î³-aminobutyric acid in fibromyalgia and healthy subjects: a multimodal neuroimaging study	2022	Fanton, Silvia, Sandström, Angelica, Tour, Jeanette, Kadetoff, Diana, Schalling, Martin, Jensen, Karin B, Sitnikov, Rouslan, Ellerbrock, Isabel, Kosek, Eva	Y	
BVS	Cerebral Perfusion and Sensory Testing Results Differ in Interstitial Cystitis/Bladder Pain Syndrome Patients with and without Fibromyalgia: A Site-Specific MAPP Network Study	2021	Deutsch, Georg, Deshpande, Hrishikesh, Lai, H Henry, Kutch, Jason J, Ness, Timothy J	Y	
BVS	Diffusion tensor imaging reveals neuronal microstructural changes in myalgic encephalomyelitis/chronic fatigue syndrome	2021	Thapaliya, Kiran, Marshall-Gradisnik, Sonya, Staines, Donald, Barnden, Leighton		NN
BVS	Dysfunctional energy metabolisms in fibromyalgia compared with healthy subjects	2021	Jung, Ye-Ha, Kim, Hyeonjin, Lee, Dasom, Lee, Jae-Yeon, Moon, Jee Youn, Choi, Soo-Hee, Kang, Do-Hyung	Y	
BVS	Impact of Fibromyalgia in the Hippocampal Subfields Volumes of Women-An MRI Study	2021	Leon-Llamas, Juan Luis, Villafaina, Santos, Murillo-Garcia, Alvaro, Gusi, Narcis	Y	
BVS	Relationship between pineal gland, sleep and melatonin in fibromyalgia women: a magnetic resonance imaging study	2021	Leon-Llamas, Juan Luis, Villafaina, Santos, Murillo-Garcia, Alvaro, Rohlfs Domínguez, Paloma, Gusi, Narcis	Y	
BVS	Neurophysiological Differences Between Women With Fibromyalgia and Healthy Controls During Dual Task: A Pilot Study	2020	Villafaina, Santos, Fuentes-García, Juan Pedro, Cano-Plasencia, Ricardo, Gusi, Narcis	Y	
BVS	Influence of pain anticipation on brain activity and pain perception in Gulf War Veterans with chronic musculoskeletal pain	2019	Lindheimer, Jacob B, Stegner, Aaron J, Ellingson-Sayen, Laura D, Van Riper, Stephanie M, Dougherty, Ryan J, Falvo, Michael J, Cook, Dane B	Y	

BVS	NYX-2925, A Novel N-methyl-D-aspartate Receptor Modulator: A First-in-Human, Randomized, Double-blind Study of Safety and Pharmacokinetics in Adults	2019	Houck, David R, Sindelar, Laurel, Sanabria, Carlos R, Stanworth, Stephanie H, Krueger, Maggie, Suh, Mary, Madsen, Torsten M	Y	
BVS	Nocturnal Gamma-Hydroxybutyrate Reduces Cortisol-Awakening Response and Morning Kynurenine Pathway Metabolites in Healthy Volunteers	2019	Dornbierer, D A, Boxler, M, Voegel, C D, Stucky, B, Steuer, A E, Binz, T M, Baumgartner, M R, Baur, D M, Quednow, B B, Kraemer, T, Seifritz, E, Landolt, H P, Bosch, O G	Y	
BVS	Subliminal emotional pictures are capable of modulating early cerebral responses to pain in fibromyalgia	2019	Peláez, Irene, Ferrera, David, Barjola, Paloma, Fernandes, Roberto, Mercado, Francisco	Y	
BVS	Altered theta oscillations in resting EEG of fibromyalgia syndrome patients	2018	Fallon, N, Chiu, Y, Nurmikko, T, Stancak, A	Y	
BVS	Fatigue Is Associated With Altered Monitoring and Preparation of Physical Effort in Patients With Chronic Fatigue Syndrome	2018	van der Schaaf, Marieke E, Roelofs, Karin, de Lange, Floris P, Geurts, Dirk E M, van der Meer, Jos W M, Knoop, Hans, Toni, Ivan		NN
BVS	Home-Based Transcranial Direct Current Stimulation Device Development: An Updated Protocol Used at Home in Healthy Subjects and Fibromyalgia Patients	2018	Carvalho, Fabiana, Brietzke, Aline Patrícia, Gasparin, Assunta, Dos Santos, Franciele Pereira, Vercelino, Rafael, Ballester, Rafael Firmino, Sanches, Paulo Roberto Stefani, da Silva, Danton Pereira, Torres, Iraci L S, Fregni, Felipe, Caumo, Wolnei	Y	
BVS	Hyperexcitability of the Central Nervous System in Children with Chronic Pain: A Systematic Review	2018	Pas, Roselien, Ickmans, Kelly, Van Oosterwijck, Sophie, Van der Cruyssen, Kelly, Foubert, Anthe, Leysen, Laurence, Nijs, Jo, Meeus, Mira	Y	
BVS	Neuroimagen de la fibromialgia: una enfermedad del cerebro	2018	Deus, J		NN
BVS	Task interference and distraction efficacy in patients with fibromyalgia: an experimental investigation	2018	Van Ryckeghem, Dimitri M L, Rost, Silke, Kissi, Ama, Vögele, Claus, Crombez, Geert		NN
BVS	Brain signature and functional impact of centralized pain: a	2017	Kutch, Jason J, Ichesco, Eric, Hampson, Johnson P, Labus,	Y	

	multidisciplinary approach to the study of chronic pelvic pain (MAPP) network study		Jennifer S, Farmer, Melissa A, Martucci, Katherine T, Ness, Timothy J, Deutsch, Georg, Apkarian, A Vania, Mackey, Sean C, Klumpp, David J, Schaeffer, Anthony J, Rodriguez, Larissa V, Kreder, Karl J, Buchwald, Dedra, Andriole, Gerald L, Lai, H Henry, Mullins, Chris, Kusek, John W, Landis, J Richard, Mayer, Emeran A, Clemens, J Quentin, Clauw, Daniel J, Harris, Richard E		
BVS	Classical Conditioning Differences Associated With Chronic Pain: A Systematic Review	2017	Harvie, Daniel S, Moseley, G Lorimer, Hillier, Susan L, Meulders, Ann	Y	
BVS	Diferencias en el volumen cerebral regional entre pacientes de fibromialgia y meditadores de larga duración	2017	Fayed, Nicolás, García-Martí, Gracián, Sanz-Requena, Roberto, Marti-Bonmati, Luis, Garcia-Campayo, Javier		NN
BVS	Difference in Regional Brain Volume between Fibromyalgia Patients and Long-Term Meditators	2017	Fayed, Nicolás, García-Martí, Gracián, Sanz-Requena, Roberto, Marti-Bonmatí, Luis, Garcia-Campayo, Javier	Y	
BVS	Elevations of Ventricular Lactate Levels Occur in Both Chronic Fatigue Syndrome and Fibromyalgia	2017	Natelson, Benjamin H, Vu, Diana, Coplan, Jeremy D, Mao, Xiangling, Blate, Michelle, Kang, Guoxin, Soto, Eli, Kapusuz, Tolga, Shungu, Dikoma C	Y	
BVS	Multimodal and simultaneous assessments of brain and spinal fluid abnormalities in chronic fatigue syndrome and the effects of psychiatric comorbidity	2017	Natelson, Benjamin H, Mao, Xiangling, Stegner, Aaron J, Lange, Gudrun, Vu, Diana, Blate, Michelle, Kang, Guoxin, Soto, Eli, Kapusuz, Tolga, Shungu, Dikoma C		NN
BVS	Prefrontal Structure Varies as a Function of Pain Symptoms in Chronic Fatigue Syndrome	2017	van der Schaaf, Marieke E, De Lange, Floris P, Schmits, Iris C, Geurts, Dirk E M, Roelofs, Karin, van der Meer, Jos W M, Toni, Ivan, Knoop, Hans		NN
BVS	A possible neural mechanism for photosensitivity in chronic pain	2016	Martenson, Melissa E, Halawa, Omar I, Tonsfeldt, Karen J,	Y	

			Maxwell, Charlene A, Hammack, Nora, Mist, Scott D, Pennesi, Mark E, Bennett, Robert M, Mauer, Kim M, Jones, Kim D, Heinricher, Mary M	
BVS	Functional Connectivity with the Default Mode Network Is Altered in Fibromyalgia Patients	2016	Fallon, Nicholas, Chiu, Yee, Nurmikko, Turo, Stancak, Andrej	Y
BVS	Habituation deficit of auditory N100m in patients with fibromyalgia	2016	Choi, W, Lim, M, Kim, J S, Chung, C K	Y
BVS	Increased Low- and High-Frequency Oscillatory Activity in the Prefrontal Cortex of Fibromyalgia Patients	2016	Lim, Manyoel, Kim, June Sic, Kim, Dajung J, Chung, Chun Kee	Y
BVS	Altered cortical processing of observed pain in patients with fibromyalgia syndrome	2015	Fallon, Nicholas, Li, Xiaoyun, Chiu, Yee, Nurmikko, Turo, Stancak, Andrej	Y
BVS	Fibromyalgia patients have reduced hippocampal volume compared with healthy controls	2015	McCrae, Christina S, O'Shea, Andrew M, Boissoneault, Jeff, Vatthauer, Karlyn E, Robinson, Michael E, Staud, Roland, Perlstein, William M, Craggs, Jason G	Y
BVS	Impaired pre-attentive auditory processing in fibromyalgia: A mismatch negativity (MMN) study	2015	Choi, Woojin, Lim, Manyoel, Kim, June Sic, Kim, Dajung J, Chung, Chun Kee	Y
BVS	Men and women with fibromyalgia: Relation between attentional function and clinical symptoms	2015	Miró, Elena, Martínez, María P, Sánchez, Ana I, Prados, Germán, Lupiáñez, Juan	Y
BVS	Reaction time, cerebral blood flow, and heart rate responses in fibromyalgia: Evidence of alterations in attentional control	2015	Reyes Del Paso, Gustavo A, Montoro, Casandra I, Duschek, Stefan	Y
BVS	Altered white matter integrity in the corpus callosum in fibromyalgia patients identified by tract-based spatial statistical analysis	2014	Kim, Dajung J, Lim, Manyoel, Kim, June Sic, Son, Kyeong Min, Kim, Hyun Ah, Chung, Chun Kee	Y
BVS	Fibromyalgia is associated with decreased connectivity between pain- and sensorimotor brain areas	2014	Flodin, Pär, Martinsen, Sofia, Löfgren, Monika, Bileviciute-Ljungar, Indre, Kosek, Eva, Fransson, Peter	Y

BVS	Síndrome dolorosa disfuncional em doentes com sensibilidade exteroceptiva assimétrica: caracterização de uma entidade clínica	2014	Kaziyama, Helena Hideko Seguchi	Y	
BVS	Alterations in endogenous opioid functional measures in chronic back pain	2013	Martikainen, Ilkka K, Peciña, Marta, Love, Tiffany M, Nuechterlein, Emily B, Cummiford, Chelsea M, Green, Carmen R, Harris, Richard E, Stohler, Christian S, Zubieta, Jon-Kar	Y	
BVS	Brain correlates of cognitive inhibition in fibromyalgia: emotional intrusion of symptom-related words	2013	Mercado, Francisco, González, José Luis, Barjola, Paloma, Fernández-Sánchez, Marisa, López-López, Almudena, Alonso, Miriam, Gómez-Esquer, Francisco	Y	
BVS	Deficient modulation of pain by a positive emotional context in fibromyalgia patients	2013	Kamping, Sandra, Bomba, Isabelle C, Kanske, Philipp, Diesch, Eugen, Flor, Herta	Y	
BVS	Ipsilateral cortical activation in fibromyalgia patients during brushing correlates with symptom severity	2013	Fallon, Nicholas, Chiu, Yee Ho, Li, Xiaoyun, Nurmikko, Turo J, Stancak, Andrej	Y	
BVS	Structural alterations in brainstem of fibromyalgia syndrome patients correlate with sensitivity to mechanical pressure	2013	Fallon, Nicholas, Alghamdi, Jamaan, Chiu, Yee, Sluming, Vanessa, Nurmikko, Turo, Stancak, Andrej	Y	
BVS	Differential central pain processing following repetitive intramuscular proton/prostaglandin E2 injections in female fibromyalgia patients and healthy controls	2011	Diers, Martin, Schley, Marcus T, Rance, Mariela, Yilmaz, Pinar, Lauer, Lydia, Rukwied, Roman, Schmelz, Martin, Flor, Herta	Y	
BVS	Distinct cerebrospinal fluid proteomes differentiate post-treatment lyme disease from chronic fatigue syndrome	2011	Schutzer, Steven E, Angel, Thomas E, Liu, Tao, Schepmoes, Athena A, Clauss, Therese R, Adkins, Joshua N, Camp, David G, Holland, Bart K, Bergquist, Jonas, Coyle, Patricia K, Smith, Richard D, Fallon, Brian A, Natelson, Benjamin H		NN

BVS	EEG spectral coherence data distinguish chronic fatigue syndrome patients from healthy controls and depressed patients--a case control study	2011	Duffy, Frank H, McAnulty, Gloria B, McCreary, Michelle C, Cuchural, George J, Komaroff, Anthony L		NN
BVS	Executive function in chronic pain patients and healthy controls: different cortical activation during response inhibition in fibromyalgia	2011	Glass, Jennifer M, Williams, David A, Fernandez-Sanchez, Maria-Luisa, Kairys, Anson, Barjola, Paloma, Heitzeg, Mary M, Clauw, Daniel J, Schmidt-Wilcke, Tobias	Y	
BVS	Increased ventricular lactate in chronic fatigue syndrome measured by 1H MRS imaging at 3.0 T. II: comparison with major depressive disorder	2010	Murrough, James W, Mao, Xiangling, Collins, Katherine A, Kelly, Chris, Andrade, Gizely, Nestadt, Paul, Levine, Susan M, Mathew, Sanjay J, Shungu, Dikoma C		NN
BVS	Intrinsic brain connectivity in fibromyalgia is associated with chronic pain intensity	2010	Napadow, Vitaly, LaCount, Lauren, Park, Kyungmo, As-Sanie, Sawsan, Clauw, Daniel J, Harris, Richard E	Y	
