# Supplementary material for: Structural and Functional Neuroimaging Findings in Fibromyalgia: A Systematic Review
Source: Eur J Pain. 2026 Jul 12;30(6):e70331. doi: 10.1002/ejp.70331 (PMC13356883; doi:10.1002/ejp.70331)
Supplement: Supplementary file 2 — Data S2: Search strategy. [file EJP-30-0-s003.pdf]

SEARCH STRATEGY – SYSTEMATIC REVIEW ON NEUROIMAGING IN FIBROMYALGIA

Inclusion criteria:

(1) Population: Studies including adults diagnosed with fibromyalgia according to the American College of Rheumatology (ACR) criteria.

(2) Comparison: Studies that included a comparison group composed of Healthy Controls (HCs).

(3) Outcome: Studies reporting quantitative results from brain neuroimaging techniques, including:

Structural Magnetic Resonance Imaging (e.g., Voxel-Based Morphometry – VBM, Surface-Based Morphometry – SBM, cortical volume/thickness analysis).

Diffusion Tensor Imaging (DTI, e.g., Fractional Anisotropy – FA, Mean Diffusivity – MD, tract-based analyses).

Functional Magnetic Resonance Imaging (fMRI), both at rest (resting-state fMRI) and during tasks (task-based fMRI).

(4) Study design: Observational case–control studies or baseline (pre-intervention) data from clinical trials or longitudinal studies that report a direct comparison between patients with fibromyalgia and healthy controls.

(5) Publication period: Studies published between 1 January 2010 and 16 October 2025.

(6) Language: Studies published in English.

Initial screening

PubMed: 254

Embase: 623

Web of Science: 414

BVS: 59

TOTAL: 1350

Duplicates removed (541): 809

PubMed

1# ("Fibromyalgia"[Mesh] OR fibromyalgia[tiab] OR fibrositis[tiab])

2# ("Neuroimaging"[Mesh] OR "Magnetic Resonance Imaging"[Mesh] OR "Functional Neuroimaging"[Mesh] OR "Diffusion Tensor Imaging"[Mesh] OR "Brain Mapping"[Mesh] OR "Brain"[Mesh] OR "Quantitative MRI"[tiab] OR "Diffusion tensor imaging"[tiab] OR "Structural analysis"[tiab] OR "Structural connectivity"[tiab] OR "Functional MRI"[tiab] OR neuroimag*[tiab] OR MRI[tiab] OR fMRI[tiab] OR DTI[tiab] OR VBM[tiab] OR "voxel-based morphometry"[tiab] OR SBM[tiab] OR "surface-based morphometry"[tiab] OR "functional connectivity"[tiab] OR "resting state"[tiab] OR brain[tiab] OR cerebral[tiab] OR cortical[tiab])

3# ("Healthy Volunteers"[Mesh] OR healthy control*[tiab] OR healthy subject*[tiab])

4# ("2010/01/01"[Date - Publication] : "2025/10/16"[Date - Publication])

5# (Review[pt] OR "Systematic Review"[pt] OR "Meta-Analysis"[pt])

Embase

1# ('fibromyalgia'/exp OR fibromyalgia:ti,ab OR fibrositis:ti,ab)

2# ('neuroimaging'/exp OR 'magnetic resonance imaging'/exp OR 'functional neuroimaging'/exp OR 'diffusion tensor imaging'/exp OR 'brain mapping'/exp OR 'brain'/exp OR 'quantitative mri':ti,ab OR 'diffusion tensor imaging':ti,ab OR 'structural analysis':ti,ab OR 'structural connectivity':ti,ab OR 'functional mri':ti,ab OR neuroimag*:ti,ab OR mri:ti,ab OR fmri:ti,ab OR dti:ti,ab OR vbm:ti,ab OR 'voxel based morphometry':ti,ab OR sbm:ti,ab OR 'surface based morphometry':ti,ab OR 'functional connectivity':ti,ab OR 'resting state':ti,ab OR brain:ti,ab OR cerebral:ti,ab OR cortical:ti,ab)

3# ('healthy volunteer'/exp OR 'healthy control*':ti,ab OR 'healthy subject*':ti,ab)

4# ([2010-2025]/py)

5# ('review':pt OR 'systematic review':pt OR 'meta analysis':pt)

Web of Science

1# TS=("Fibromyalgia" OR fibrositis)

2# TS=("Neuroimaging" OR "Magnetic Resonance Imaging" OR "Functional Neuroimaging" OR "Diffusion Tensor Imaging" OR "Brain Mapping" OR "Brain" OR "Quantitative MRI" OR "Diffusion tensor imaging" OR "Structural analysis" OR "Structural connectivity" OR "Functional MRI" OR neuroimag* OR MRI OR fMRI OR DTI OR VBM OR "voxel-based morphometry" OR SBM OR "surface-based morphometry" OR "functional connectivity" OR "resting state" OR brain OR cerebral OR cortical)

3# TS=("Healthy Volunteers" OR "healthy control*" OR "healthy subject*")

4# PY=(2010-2025)

5# DT=(Review OR "Systematic Review" OR "Meta-Analysis")

BVS (DeCS / LILACS)

1# (MH:"Fibromialgia") OR (TW:(Fibromialgia OR Fibromyalgia OR Fibrositis OR Fibrosite))

2# (MH:"Neuroimagem" OR MH:"Magnetic Resonance Imaging" OR MH:"Functional Neuroimaging" OR MH:"Diffusion Tensor Imaging" OR MH:"Brain Mapping" OR MH:"Brain") OR (TW:("Quantitative MRI" OR "Ressonância Magnética Quantitativa" OR "Diffusion tensor imaging" OR "Imagem de Tensor de Difusão" OR "Structural analysis" OR "Análise Estrutural" OR "Structural connectivity" OR "Conectividade Estrutural" OR "Functional MRI" OR fMRI OR "Ressonância Magnética funcional" OR neuroimag$ OR MRI OR DTI OR VBM OR "voxel-based morphometry" OR SBM OR "surface-based morphometry" OR "functional connectivity" OR "conectividade funcional" OR "resting state" OR "estado de repouso" OR brain OR cérebro OR encéfalo OR cerebro OR cerebral OR cortical))

3# (MH:"Healthy Volunteers") OR (TW:("Healthy control$" OR "healthy subject$" OR "controles saudáveis" OR "sujeitos saudáveis" OR "voluntários saudáveis" OR "controles sanos" OR "sujetos sanos" OR "voluntarios sanos"))

4# : (year_cluster:([2010 TO 2025]))

5# : (type:("Review" OR "Systematic Review" OR "Meta-Analysis" OR "Revisão" OR "Revisión sistemática" OR "Metaanálisis"))
