# Supplementary material for: Structural and Functional Neuroimaging Findings in Fibromyalgia: A Systematic Review
Source: Eur J Pain. 2026 Jul 12;30(6):e70331. doi: 10.1002/ejp.70331 (PMC13356883; doi:10.1002/ejp.70331)
Supplement: Supplementary file 4 — Table S1: Methodological quality assessment of included studies using the modified Newcastle–Ottawa Scale (NOS). Table S2: comorbidities and clinical characteristics. Table S3: Paradigm‐by‐region family counts underlying Figure 3. For each task paradigm and region‐of‐interest (ROI) family, the number of reports showing increased (hyperactivation) versus decreased (hypoactivation) task‐evoked activation in fibromyalgia versus healthy controls. Dominant direction reflects the more frequent direction (Tie/Mixed when equal). Normalized balance = (n hyperactivation − n hypoactivation)/7, where 7 is the largest absolute net difference across cells (range −1 to +1). Figure 3 score rescales this to 0–1 as (normalized balance + 1)/2 for the colour mapping (0 = decreased/blue, 1 = increased/red). ACC, anterior cingulate cortex; MCC, mid‐cingulate cortex; OFC, orbitofrontal cortex; PCC, posterior cingulate cortex. [file EJP-30-0-s004.pdf]

Supplementary Table S1. Methodological quality assessment of included studies using the modified Newcastle–Ottawa Scale (NOS)

Studies are listed in the order provided in the source worksheet. NOS score★ is shown as total stars awarded out of 9. Quality categories follow the source file.

Author / Year	Selection	Comparability	Outcome	NOS score★	Quality
Agoalikum et al., 2025	3	1	2	6/9 ★★★★★★☆☆☆	**Moderate**
Elkana et al., 2025	3	1	2	6/9 ★★★★★★☆☆☆	**Moderate**
Agoalikum et al., 2025	3	1	2	6/9 ★★★★★★☆☆☆	**Moderate**
Wu et al., 2025	3	2	2	7/9 ★★★★★★★☆☆	**High**
Renz et al., 2025	3	1	2	6/9 ★★★★★★☆☆☆	**Moderate**
Balducci et al., 2024	4	1	3	8/9 ★★★★★★★★☆	**High**
Liu et al., 2024	3	2	1	6/9 ★★★★★★☆☆☆	**Moderate**
Gurevitchet al., 2024	3	1	1	5/9 ★★★★★☆☆☆☆	**Moderate**
Neto et al., 2024	3	2	3	8/9 ★★★★★★★★☆	**High**
Flodin et al., 2014	3	2	1	6/9 ★★★★★★☆☆☆	**Moderate**
Martínez-López et al., 2023	3	2	1	6/9 ★★★★★★☆☆☆	**Moderate**
Manyoel Lim et al., 2021	3	2	2	7/9 ★★★★★★★☆☆	**High**
Mosch et al., 2023	3	1	3	7/9 ★★★★★★★☆☆	**High**
Izuno et al., 2023	3	1	3	7/9 ★★★★★★★☆☆	**High**
Sandström et al., 2024	3	1	3	7/9 ★★★★★★★☆☆	**High**
Löfgren et al., 2023	3	1	2	6/9 ★★★★★★☆☆☆	**Moderate**
Tu et al., 2023	3	1	2	6/9 ★★★★★★☆☆☆	**Moderate**
Balducci et al., 2022	3	1	3	7/9 ★★★★★★★☆☆	**High**
Ioachim et al., 2022	4	1	2	7/9 ★★★★★★★☆☆	**High**
Aster et al., 2022	4	1	2	7/9 ★★★★★★★☆☆	**High**
Oliva et al., 2022	4	1	3	8/9 ★★★★★★★★☆	**High**
Park et al., 2022	4	2	3	9/9 ★★★★★★★★★	**High**
Tu et al., 2022	3	1	3	7/9 ★★★★★★★☆☆	**High**
Tu et al., 2022	3	1	3	7/9 ★★★★★★★☆☆	**High**
Cheng et al., 2022	4	1	3	8/9 ★★★★★★★★☆	**High**

Author / Year	Selection	Comparability	Outcome	NOS score★	Quality
Park et al., 2022	4	1	3	8/9 ★★★★★★★★☆	**High**
Fanton et al., 2022	4	1	3	8/9 ★★★★★★★★☆	**High**
Kong et al., 2021	4	1	3	8/9 ★★★★★★★★☆	**High**
Larkin et al., 2021	3	1	3	7/9 ★★★★★★★☆☆	**High**
Warren et al., 2021	4	1	3	8/9 ★★★★★★★★☆	**High**
Leon-Llamas et al., 2021	4	2	2	8/9 ★★★★★★★★☆	**High**
Ellerbrock et al., 2021	4	2	3	9/9 ★★★★★★★★★	**High**
Kim et al., 2021	4	1	3	8/9 ★★★★★★★★☆	**High**
Hubbard et al., 2020	4	1	3	8/9 ★★★★★★★★☆	**High**
van Ettinger-Veenstra et al., 2020	4	2	3	9/9 ★★★★★★★★★	**High**
Jung et al., 2020	3	1	4	8/9 ★★★★★★★★☆	**High**
Sandström et al., 2020	4	2	3	9/9 ★★★★★★★★★	**High**
Feraco et al., 2020	3	1	2	6/9 ★★★★★★☆☆☆	**Moderate**
Kim et al., 2020	4	1	3	8/9 ★★★★★★★★☆	**High**
Kong et al., 2020	4	2	3	9/9 ★★★★★★★★★	**High**
Martucci et al., 2019	4	1	3	8/9 ★★★★★★★★☆	**High**
Sundermann et al., 2019	4	1	3	8/9 ★★★★★★★★☆	**High**
Pando-Naude et al., 2019	4	2	3	9/9 ★★★★★★★★★	**High**
Ellingson et al., 2018	4	2	3	9/9 ★★★★★★★★★	**High**
Jarrahi et al., 2018	4	1	3	8/9 ★★★★★★★★☆	**High**
Harper et al., 2018	4	2	3	9/9 ★★★★★★★★★	**High**
Martinsen et al., 2018	4	1	2	7/9 ★★★★★★★☆☆	**High**
Kutch et al., 2017	2	1	3	6/9 ★★★★★★☆☆☆	**Moderate**
Fayed et al., 2017	4	1	3	8/9 ★★★★★★★★☆	**High**
Fayed et al., 2017	4	1	3	8/9 ★★★★★★★★☆	**High**
Jarrahi et al., 2017	3	1	3	7/9 ★★★★★★★☆☆	**High**
Coulombe et al., 2017	4	1	3	8/9 ★★★★★★★★☆	**High**
Schreiber et al., 2017	4	1	3	8/9 ★★★★★★★★☆	**High**

Author / Year	Selection	Comparability	Outcome	NOS score★	Quality
Derbyshire et al., 2017	3	0	2	5/9 ★★★★★☆☆☆☆	**Moderate**
López-Solà et al., 2017	4	2	3	9/9 ★★★★★★★★★	**High**
Truini et al., 2016	4	1	3	8/9 ★★★★★★★★☆	**High**
Ichesco et al., 2016	4	1	3	8/9 ★★★★★★★★☆	**High**
Fallon et al., 2016	4	1	3	8/9 ★★★★★★★★☆	**High**
Harte et al., 2016	4	2	3	9/9 ★★★★★★★★★	**High**
Robinson et al., 2015	3	1	3	7/9 ★★★★★★★☆☆	**High**
McCrae et al., 2015	4	2	2	8/9 ★★★★★★★★☆	**High**
Flodin et al., 2015	4	1	3	8/9 ★★★★★★★★☆	**High**
Rahm et al., 2015	3	1	3	7/9 ★★★★★★★☆☆	**High**
Kim et al., 2015	4	1	3	8/9 ★★★★★★★★☆	**High**
Ichesco et al., 2014	4	1	3	8/9 ★★★★★★★★☆	**High**
Kim et al., 2014	3	1	3	7/9 ★★★★★★★☆☆	**High**
Schmidt-Wilcke et al., 2014	3	1	2	6/9 ★★★★★★☆☆☆	**Moderate**
Loggia et al., 2014	3	1	2	6/9 ★★★★★★☆☆☆	**Moderate**
Martinsen et al., 2014	4	1	3	8/9 ★★★★★★★★☆	**High**
Kamping et al., 2013	4	2	3	9/9 ★★★★★★★★★	**High**
Flodin et al., 2014	3	2	3	8/9 ★★★★★★★★☆	**High**
Kim et al., 2013	2	1	2	5/9 ★★★★★☆☆☆☆	**Moderate**
Ceko et al., 2013	4	2	3	9/9 ★★★★★★★★★	**High**
Lee et al., 2013	4	1	3	8/9 ★★★★★★★★☆	**High**
Burgmer et al., 2012	4	2	3	9/9 ★★★★★★★★★	**High**
Cifre et al., 2012	4	1	3	8/9 ★★★★★★★★☆	**High**
Craggs et al., 2012	4	1	3	8/9 ★★★★★★★★☆	**High**
Jensen et al., 2013	4	2	3	9/9 ★★★★★★★★★	**High**
Fallon et al., 2013	4	2	3	9/9 ★★★★★★★★★	**High**
Jensen et al., 2012	4	1	3	8/9 ★★★★★★★★☆	**High**
Seo et al., 2012	4	2	3	9/9 ★★★★★★★★★	**High**

Author / Year	Selection	Comparability	Outcome	NOS score★	Quality
Burgmer et al., 2011	3	2	3	8/9 ★★★★★★★★☆	**High**
Diers et al., 2011	3	1	2	6/9 ★★★★★★☆☆☆	**Moderate**
Glass et al., 2011	3	2	3	8/9 ★★★★★★★★☆	**High**
Robinson et al., 2011	4	1	3	8/9 ★★★★★★★★☆	**High**
Burgmer et al., 2010	4	2	3	9/9 ★★★★★★★★★	**High**
Napadow et al., 2010	4	1	3	8/9 ★★★★★★★★☆	**High**
Fayed et al., 2010	3	1	2	6/9 ★★★★★★☆☆☆	**Moderate**
Pomares et al., 2017	4	2	3	9/9 ★★★★★★★★★	**High**
Park et al., 2022	3	1	3	7/9 ★★★★★★★☆☆	**High**
Baker et. al. 2022	3	1	3	7/9 ★★★★★★★☆☆	**High**
Kaplan et al. 2019	4	1	3	8/9 ★★★★★★★★☆	**High**
Kim et al. 2015	4	1	3	8/9 ★★★★★★★★☆	**High**

Abbreviations: NOS, Newcastle–Ottawa Scale.

Supplementary Table 2 – Comorbidities and Clinical Characteristics

Tables extracted from the uploaded workbook and arranged sequentially for supplementary material.

Tasked-Based fMRI

Author / Year	Age (mean ± SD)	Pain duration	Pain intensity at baseline	FM severity / impact	Mood burden	Morphometry scope	Modality	Psychiatric comorbidities reported	Other comorbidities reported	Medication use reported / restricted	Clinical scales reported	Confounders controlled or matched?	Notes / NR
Balducci et al. / 2024	41.9 (6.3) (HC: 41.2 [6.1])	NR	VAS for pain during the interview: 45.7 (19.8) (HC: 1.2 [3.5])	Widespread pain index: 11.8 (4.3); Symptom severity scale: 8.3 (2.5)	HAMD total score: 15.2 (6.5); HAMA total score: 21.2 (7.0)	NR	Task-based fMRI	Current psychiatric disorder in 63%; major depressive disorder most common (46.7%); MINI-Plus used to document depression and anxiety; major psychiatric disorders excluded in HC	NR	56.67% used medication daily; pregabalin was the most prescribed	Edinburgh Handedness Inventory Short Form; MINI-Plus; HAMD; HAMA; Emotional Regulation Questionnaire; Toronto Alexithymia Scale; Positive and Negative Affect Schedule; AMAI NSE 8×7; Fibromyalgia Impact Questionnaire; McGill Pain Questionnaire; VAS ratings	Matched for age and years of education; all participants right-handed; age and education entered as covariates; partial correlations controlled for depression and anxiety	30 female FM and 31 female HC analyzed after fMRI quality control
Park et al. / 2024	M = 37.87, SD = 13.89 (HC: M = 41.79, SD = 12.36)	9 months to 20 years (M = 8.58, SD = 8.50)	Pain Severity (BPI): 5.3±1.3	NR	Trait Anxiety (STAI): 45.9±10.5; State Anxiety (STAI): 42.0±9.4; Depression (BDI): 19.0±8.9	NR	Task-based fMRI	Major and/or uncontrolled depression or anxiety excluded; uncontrolled psychiatric disorders excluded	NR	Regular medications allowed/continued; no opioid use at time of study, none in prior 90 days, and no prior use >1 month lifetime; medication classes included NSAIDs, acetaminophen, other pain medicine, SNRIs, SSRIs, other anxiolytics, triptans, trazodone, antiepileptic, muscle relaxants, GABA analogs, benzodiazepines, and NDRI	BDI; STAI-State; STAI-Trait; BIS/BAS; Brief Pain Inventory-Short Form (BPI-sf); Fibromyalgia Assessment Form (FAF)	All participants were female; no significant age difference between groups; participants with excessive head motion were excluded; motion parameters were regressed out during denoising	26 FM and 28 HC enrolled; 24 FM and 24 HC analyzed after excessive head motion exclusions
Park et al. / 2023	37.87 ± 13.89 (controls: 41.79 ± 12.36)	9 months to 20 years (M = 8.58, SD = 8.50)	NR	NR	NR	NR	Task-based fMRI	No patients reported uncontrolled depression or anxiety.	NR	Regular medications allowed; no current opioids, no opioid use >90 days prior, and no prior opioid use >1 month lifetime; 25/26 patients used NSAIDs, acetaminophen, other pain medicine, SNRIs, SSRIs, anxiolytics, triptans, trazodone, antiepileptics, muscle relaxants, GABA analogs, benzodiazepines, or NDRI; controls reported no pain- or mood-altering medications except 2 single doses.	Average pain score (0-10 verbal scale); fibromyalgia-related pain symptom duration	All female; head-motion outliers excluded; motion parameters regressed out; sensitivity check excluding 2 medicated controls did not significantly change results.	Preprint; final analysis included 24 patients with fibromyalgia and 24 healthy controls after motion exclusions. Only uploaded file considered.
Sandström et al. / 2024	48 ± 8 years (controls: 48 ± 8 years)	119 (90, 11, 408) months	VAS pain current: 52 (23, 6, 99)	FIQ: 62 (16, 12, 92)	BDI: 15 (8, 1, 34); STAI-State: 42 (12, 24, 75)	NR	Task-based fMRI	Psychiatric disorders including ongoing treatment for depression or anxiety were excluded.	Other dominant pain conditions than FM; rheumatic or autoimmune diseases; other severe somatic diseases (neurological, cardiovascular, cancer, etc.); hypertension (>160/90 mmHg) were excluded.	Ongoing medication with antidepressants or anticonvulsants excluded; inability to refrain from NSAIDs, analgesics, or hypnotics for at least 48 hours prior to participation (72 hours prior to fMRI) excluded; healthy controls had no regular NSAIDs, analgesics, sleep medication, antidepressants, or anticonvulsants.	VAS pain current; VAS pain last week; tender points; FIQ; PCS; BDI; STAI-State; pressure pain thresholds (PPTs); conditioned pain modulation (CPM); 0-100 pain ratings during conditioning	Age- and sex-balanced healthy controls; right-handed only; female only; working age 20-60 years; participants with excessive head motion or structural brain anomalies excluded; 6 motion parameters added as regressors of no interest.	MRS data were not included in the current study; functional whole-brain activation and PPI analyses were reported, but no morphometric analysis was reported. Only uploaded file considered.
Mosch et al. / 2023	50.48 ± 9.89 years (HC: 46.62 ± 13.08 years)	14.88 years (SD = 11.82; range 2 to 44 years)	NR	NR	NR	Both (whole-brain VBM + predefined bilateral ROIs: VLPFC, DLPFC, OFC, dACC)	Task-based fMRI; Structural MRI	Acute major depression or bipolar disorder excluded; psychotropic medication users and psychotic patients excluded; 16 FM reported previous major depressive episodes; 4 had a history of generalized anxiety disorder and/or PTSD; no HC current or past psychopathological symptoms	NR	No pain medication on the examination day; opioid use suspended no later than 3 days before MRI (1x fentanyl patches, 1x tramadol); psychotropic medication users excluded	VAS for intensity and unpleasantness; pain threshold; pain tolerance; perceived control rating (0-10); pain duration	Female-only sample; left-handed persons excluded; age not significantly different between groups; TIV used as a covariate in VBM; FC denoising included WM/CSF confounds, realignment, and scrubbing of head motion-induced artifacts	Methods report 22 FM / 21 HC, while the abstract states HC n = 21 and FM n = 23
Oliva et al. / 2022	mean age 43, range 25-60 (HC: mean age 35, range 20-59)	NR	BPI pain on average 6.4 ± 1.7; BPI pain now 5.3 ± 1.6	Widespread Pain Index 13.5 ± 2.6; Symptom Severity 10 ± 1.5	Hospital Anxiety (HADS) 12.2 ± 3.6; Hospital Depression (HADS) 10.5 ± 4.7	NR	Task-based fMRI	History of major psychiatric illness excluded	Other chronic painful conditions excluded; significant medical disorder precluded controls; pregnancy excluded	Regular medications not altered; included non-opioid analgesics (n = 13), opioids (n = 9), tricyclic antidepressants/serotonin and noradrenaline reuptake inhibitors (n = 11), and gabapentinoids (n = 7); medications taken in the previous 72 hours were recorded	Widespread Pain and Symptom Severity Index; Edinburgh Handedness Inventory; PainDETECT; Brief Pain Inventory (pain now, pain on average); HADS; Pain Anxiety Symptom Scales; QST (warm detection, heat pain, cold	Sex-matched healthy controls; right-handed only / left-handed excluded; stimulus temperature individually calibrated to a 6/10 pain score; RSVP task individually titrated to 70% performance; standard MRI screening and safety exclusions applied	Healthy controls were 8 years younger on average (P = 0.03); inclusion required fibromyalgia diagnosis for at least 6 months before entry

Author / Year	Age (mean ± SD)	Pain duration	Pain intensity at baseline	FM severity / impact	Mood burden	Morphometry scope	Modality	Psychiatric comorbidities reported	Other comorbidities reported	Medication use reported / restricted	Clinical scales reported	Confounders controlled or matched?	Notes / NR
											detection, cold pain, pressure pain threshold)		
Cheng et al. / 2022	39.8 ± 12.3 (controls: 38.8 ± 12.9)	NR	NR	NR	NR	NR	Resting-state fMRI; Task-based fMRI	NR	NR	NR	0–100 pain rating scale; temporal summation of pain (TSP)	Age- and sex-matched healthy controls; all participants female; physiologic data used to correct cardiorespiratory artifacts; subjects excluded after quality control of psychophysical/image-acquisition data or healthy subject inclusion criteria	Resting-state and sustained cuff pressure–pain fMRI runs; data collected prior to any intervention
Park et al. / 2022	35.9 ± 12.3 (controls: 44.2 ± 12.1)	6.03 ± 5.33 years (range: 9 months to 20 years)	Pain Severity (BPI): 4.5 ± 1.9 (5.25)	Number of Pain Areas (FAF): 12.0 ± 3.8 (12)	BDI: 16.7 ± 8.7 (15); STAI-Trait: 48.2 ± 10.4 (51); STAI-State: 38.7 ± 9.3 (37.5)	NR	Task-based fMRI	No major and/or uncontrolled depression or anxiety; uncontrolled psychiatric disorder excluded	NR	Opioid-restricted/opioid-naïve (no opioid use within previous 90 days; no prior use >30 days lifetime); controls reported no pain or mood-altering medications; patients reported NSAIDs, acetaminophen, SNRIs, SSRIs, trazodone, TCAs, other anxiolytics, triptans, antiepileptics, muscle relaxants, GABA analogs, benzodiazepines, and NDRIs	BDI; STAI-State; STAI-Trait; BIS/BAS; POMS; PANAS; BPI; PROMIS Fatigue; BSI (anxiety, depression); FAF	All participants female; age included as a continuous covariate (ANCOVA) in primary analyses; motion censoring (>0.5 Euclidean norm); participants with missing behavioral/clinical data excluded from relevant correlation analyses	MID task; exploratory task-based network connectivity implied enhanced connectivity within the default mode network
Ellerbrock et al. / 2021	47.4 ± 7.9 (HC 47.9 ± 7.9)	AA: 184 (±112, 24, 492) mo; */G: 189 (±89.2, 60, 408) mo	NR	FIQ — AA: 63.6 (±16.2); */G: 61.5 (±18.3)	BDI — AA: 16 (±8); */G: 15.1 (±7.8); STAI-S — AA: 43.3 (±11.8); */G: 44.5 (±12.3)	NR	Task-based fMRI	Psychiatric disorders incl. ongoing treatment for depression or anxiety excluded	Other dominant pain conditions, painful osteoarthritis, rheumatic/autoimmune diseases, severe somatic diseases, hypertension excluded	Antidepressants/anticonvulsants excluded; analgesics/NSAIDs/hypnotics restricted before visits; no strong opioids	PCS, BDI, STAI-State, SF-36 bodily pain, FIQ, PPT, VAS pain ratings	HC age-balanced; right-handed women, working age 20–60; excessive-motion exclusion (FD > 0.5 in >15% images); mixed models adjusted for pressure level/time/PPT where specified	Behavioral n=118; final fMRI n=105; OPRM1 grouped as AA vs AG/GG
Hubbard et al. / 2020	46.13 ± 13.44 (CTL 45.53 ± 12.40)	NR	NRS ratings: 44.84 ± 7.70	NR	NR	NR	Task-based fMRI	History of psychiatric disorders excluded	Neurological or autoimmune disorders, cardiac events/head injury excluded; MRI contraindication, recreational drug use incl. opioids, pregnancy/plans excluded	Patients continued usual medications, including antidepressants, gabapentin, NSAIDs, and acetaminophen	NRS, Brief Pain Inventory, Neuropathic Pain Questionnaire, Widespread Pain Inventory, Symptom Severity Index, PCS, BDI, verbal anxiety NRS, QST, cuff pain threshold	Healthy controls frequency matched for age and gender; cuff pressure individually calibrated to ~40/100 NRS; motion regressors/outlier scrubbing; no significant group differences in head motion	Final sample 38 FM / 15 CTL after exclusions; onset and offset modeled as stick functions
Sandström et al. / 2020	47 (8) (HC: 48 (8))	119 (87, 11-408) months	VAS pain current: 52 (22, 6-99)	FIQ: 63 (17, 13-95)	BDI: 16 (8, 1-36); STAI-Trait: 43 (12, 24-75); STAI-State: 48 (8, 34-70)	NR	Task-based fMRI	Psychiatric disorders including ongoing treatment for depression or anxiety were excluded	Other dominant pain conditions than FM; rheumatic or autoimmune diseases; other severe somatic diseases (neurological, cardiovascular, cancer, etc.) were excluded	Antidepressants or anticonvulsants excluded; had to refrain from NSAIDs, analgesics, or hypnotics before participation; HC without regular NSAIDs, analgesics, or sleep medication	VAS pain current; VAS pain last week; tender points; disease duration; FIQ; PCS; BDI; STAI-Trait; STAI-State; P10/P50/P30 VAS	HC age-balanced; right-handed women; excessive head motion excluded; button presses and 6 motion parameters were regressors of no interest	Final fMRI sample: 67 FMS and 34 HC; only the experimental test phase was analyzed
Ellingson et al. / 2018	42.3 (11.3) (CO: 40.7 (9.3))	NR	SF-MPQ VAS: 38.3 (17.0)	FIQ: 51.2 (15.2)	BDI: 9.2 (8.1); STAI Trait Anxiety: 38.2 (10.6); POMS Total Mood Disturbance: 133.7 (30.4)	NR	Task-based fMRI	Current diagnosis of Axis I psychiatric disorders excluded	NR	Regular opioids, cardiovascular medications, anticonvulsants, and high-dose antidepressants excluded; low-dose antidepressants permitted (4 FM patients) and maintained; abstain from pain medications for 24 hours before testing	BDI; STAI; POMS; SF-MPQ; FIQ; PCS; Gracely Box Scales pain intensity/unpleasantness	Age- and sex-matched female controls; individualized perceptually relative pain stimulus; BDI controlled in partial correlations/brain analyses; excessive head movement >2 mm excluded	Twenty FM patients and 20 age-matched female controls were enrolled; 4 FM and 2 controls were excluded from imaging analyses for excessive head motion
Schreiber et al. / 2017	46.3 ± 11.4 (controls 44.1 ± 14.8)	NR	5.4 ± 2.1 (Pain severity (BPI; NRS 0–10))	Widespread pain index 10.9 ± 2.6; Symptom severity sum 9.3 ± 1.9	Depression (BDI) 15.2 ± 8.2	NR	Task-based fMRI	Anxiety disorders or significant anxiety symptoms interfering with MRI procedures excluded	History of significant neurologic disorder, significant cardiac events, significant head injury, pregnancy excluded	Opioids excluded; gabapentin, antidepressants, nonsteroidal anti-inflammatory drugs, and acetaminophen allowed/continued	Clinical pain NRS; Brief Pain Inventory; neuropathic pain questionnaire; Widespread Pain Inventory; Symptom Severity index; Beck Depression Inventory; Pain Catastrophizing Scale; anxiety NRS; Situational PCS; QST NRS	Healthy controls recruited to achieve a balance of age and sex; 6 head motion parameters and motion outlier regressors included; no significant group differences in motion parameters	Final imaging sample: FM n = 38, controls n = 15; PAS rating 15 seconds after cuff deflation 13.6 ± 20.4
Derbyshire et al. / 2017	51.4 years (controls 25.3 years)	NR	4.1 (SE 0.6) prescan pain	NR	HADS-A 9.5 (1.1); HADS-D 7.7 (1.3)	NR	Task-based fMRI	NR	NR	NR	Hospital Anxiety and Depression Scale (HADS); Harvard Group Scale of Hypnotic Susceptibility (HGSHS:A objective and subjective); pain dial rating (0–10); hypnotic depth (0–10); control over pain (0–10)	Participants selected for high hypnotic suggestibility (>8/12 on HGSHS:A) and ability to respond to pain-control suggestions; hypnosis/no-hypnosis order reversed across participants	All FM participants were women; suggestions altered clinical FM pain, whereas controls altered experimental heat pain

Author / Year	Age (mean ± SD)	Pain duration	Pain intensity at baseline	FM severity / impact	Mood burden	Morphometry scope	Modality	Psychiatric comorbidities reported	Other comorbidities reported	Medication use reported / restricted	Clinical scales reported	Confounders controlled or matched?	Notes / NR
López-Solà et al. / 2017	46.27 ± 7.72 (controls 43.86 ± 6.05)	80.41 ± 52.05 months	Clinical Pain (0–100 NRS) 72.03 ± 14.82	FIQ (Total Score) 66.86 ± 15.79	HADS-Depression 8.89 ± 4.72; HADS-Anxiety 11.54 ± 4.15	NR	Task-based fMRI	History of psychiatric illness excluded in controls; NR for FM psychiatric comorbidity diagnoses	Relevant medical and neurological disorders, chronic or acute pain, and substance abuse excluded in controls	Stable medical treatment allowed; rescue analgesic drugs withheld 72 h before scanning; antidepressants, anxiolytics, hypnotics, gabapentin, ibuprofen, paracetamol, tramadol reported	FIQ; SF-36 General Perception of Health; HADS; Clinical Pain (0–100 NRS); post-scan pain intensity and unpleasantness ratings	Matched for age, education status, and handedness (all right-handed); head motion quantified and entered in logistic regression; controls excluded for relevant medical/neurological disorders, chronic/acute pain, substance abuse, or psychiatric history	37 female FM patients and 35 female matched healthy controls; combined classifier: 92% sensitivity and 94% specificity
Walitt et al. / 2016	44.9 ± 10.2 (controls 44.2 ± 11.2)	NR	BPI severity 4.59 ± 2.26	FIQ impact 53.5 ± 12.8	NR	NR	Task-based fMRI	Healthy controls did not have any psychiatric diagnoses; NR for FM psychiatric comorbidity diagnoses	FM patients did not have concomitant medical diagnoses; healthy controls did not have any medical diagnoses	Healthy controls were not taking any medications; FM medication use NR	FIQ; BPI; MFI (general fatigue, mental fatigue); MASQ (verbal memory, attention/concentration, language); N-back accuracy; N-back reaction time	Age-matched, female, right-handed groups; left-handedness, pregnancy, body metals/pacemakers, and claustrophobia excluded; six motion parameters included as regressors of no interest; no significant group differences in head motion	One FM excluded for pregnancy; one FM and three controls excluded for not understanding the N-back; no group differences in working-memory performance or task-related BOLD response
Rahm et al. / 2015	48.5 (6.5) (controls 46.4 (8.4))	6.7 (6.9) years	NR	NR	BDI 20.0 (6.8)	NR	Task-based fMRI	Depressive comorbidity reported; psychosis excluded; HC screened for lifetime psychiatric disorder	Rheumatic, oncological or neurological disease excluded; primary FM only (secondary FM due to inflammatory disease excluded)	Concurrent analgesic or psychotropic medication allowed; participants asked not to take medication in the morning of scanning; tramadol (3), hydromorphone (1), antidepressants (6: mirtazapine, sertraline, doxepin, amitriptyline, clomipramine, duloxetine)	Symptom Checklist-27 (SCL-27/GSI); Beck Depression Inventory (BDI); post-scan pain ratings (0–10); Duration of illness (years)	All participants right-handed women; exclusions by rheumatic/oncological/neurological disease, psychosis, claustrophobia, cognitive deficits and pregnancy; six realignment parameters modeled; analyses repeated with medication and BDI as covariates	Pilot study; all 22 fMRI datasets available; post-scan pain ratings available for only 7 FM and 8 HC due to data loss
Schmidt-Wilcke et al. / 2014	44.1 (9.9) (controls 42.7 (12.2))	NR	PED 54.4 (16.4) (0–100)	NR	CES-D 15.9 (9.4); STPI 18.6 (5.8)	NR	Task-based fMRI	Present psychiatric disorder involving psychosis, current suicide risk/attempt within 2 years, or substance abuse within 2 years excluded	Severe physical impairment/coexisting physical injury; morbid obesity; autoimmune, cardiopulmonary, uncontrolled endocrine or allergic disorders; malignancy within 2 years excluded	NR	Electronic pain diary / PED (0–100); percentage body area in pain (%BP); CES-D; STPI; Go/No-Go false alarms; reaction time	All participants female; extensive medical/psychiatric/MRI exclusions; poor-quality images/motion >2 mm translation or >5° rotation excluded; motion parameters modeled as regressors of no interest	Longitudinal 12-week follow-up; final analysis included 17 FM and 12 HC due to missing data/poor image quality
Loggia et al. / 2014	44.0 ± 11.9 (controls 44.2 ± 14.3)	12.5 ± 12.2 years	Clinical pain intensity (0–100) 34.3 ± 25.19	BPI, Pain Interference (0–10) 5.5 ± 2.0	BDI (0–63) 17.0 ± 13.6	NR	Task-based fMRI	Current or past history of significant psychiatric disorders excluded	Healthy controls free from chronic pain and rheumatic disease; significant neurological or cardiovascular disorders, significant head injury, implanted medical or metallic objects, and pregnancy excluded	Current opioid use excluded	Beck Depression Inventory; Fatigue VAS; Widespread Pain Index; Short Form 36 Health Survey; Brief Pain Inventory; clinical pain VAS intensity/unpleasantness; cuff pain intensity/unpleasantness ratings	Exclusion criteria handled by design; pain stimuli calibrated to elicit ~50/100 pain rating; gender distribution compared across groups	ROI analyses focused on nucleus accumbens and ventral tegmental area
Martinsen et al. / 2014	49.8 years (range 25–64) (controls 46.3 years, range 20–63)	8.9 years (0.5–19)	Pain VAS (mm) 45.3 (5–92)	FIQ 63.1 (42.5–85.0)	HADS-D 7.3 (3.0–16.3); HADS-A 8.8 (0–18)	NR	Task-based fMRI	Other severe psychiatric disorders excluded	High blood pressure (>160/90 mmHg), osteoarthritis in hip or knee, and other primary causes of pain than FM excluded	One patient on anticonvulsants; 11 on antidepressants (4 TCA, 4 SSRI, 3 SNRI); daily NSAIDs 2, acetaminophen 4, tramadol 1; refrained from hypnotics, NSAIDs, acetaminophen and tramadol/other analgesics 48 h before study participation and 72 h before fMRI	FIQ; HADS; SF-36; Pain VAS; pressure pain thresholds; blood pressure; heart rate; reaction times during SCWT	All participants were women; movement parameters entered as covariates of no interest; congruent/incongruent order and thigh side were counterbalanced	fMRI study 2 final sample: 23 FM and 28 healthy controls
Burgmer et al. / 2012	52.59 ± 7.95 (controls 49.53 ± 8.87)	At least 2 years duration	NRS 1 4.71 ± 9.43 (pre-incision)	PDI 26.35 ± 13.45; FFbH 70.23 ± 17.29	HADS 21.24 ± 7.87; Preanxiety 32.47 ± 25.47	NR	Task-based fMRI	Current axis I mental disorder excluded	Rheumatic or endocrinologic diseases excluded; controls free of ongoing pain symptoms	10 patients used no pain medication; remaining patients used steroids, non-steroid anti-rheumatics, and tricyclic antidepressants; medications that may alter pain perception or brain activation were discontinued 48 h prior to scanning	PDI; short-form MPQ; FFbH; HADS; anxiousness VAS; NRS; primary hyperalgesia (pH); secondary hyperalgesia (sH)	Female-only sample; HADS, pre-anxiety, and experimental pain included as nuisance variables; motion-specific realignment parameters added; exclusion if movement >4 mm translation or >4° rotation	Experimental skin incision in the right volar forearm to induce primary and secondary hyperalgesia
Craggs et al. / 2012	NR	NR	NR	NR	NR	NR	Task-based fMRI	NR	NR	NR	NRS/NPS pain ratings; clinical pain and anxiety ratings	Stimuli were tailored to produce comparable, robust TSSP ratings of moderate pain across subjects; head positions were fixed with foam pillows to minimize motion artifacts	Study extended a previous dataset with 11 NC participants and 13 FM participants; no significant group differences in pain-related brain activity were found when TSSP was equivalent across subjects
Jensen et al. / 2012	37.8 (6.8) (controls 33.6 (8.6))	123.8 (76.8) months	Average clinical pain during	FIQ 71.0 (12.6)	NR	NR	Task-based fMRI	Severe psychiatric illness excluded (including severe melancholic depressive	Significant cardiovascular, pulmonary, gastrointestinal, hepatic,	CNS-acting therapies washed out; antidepressants, anticonvulsants, mood stabilizers, opioids, narcotic	VAS (average clinical pain previous week; current pain); FIQ	Age-matched (2 FM per HC); all female; right-handed; subjectively calibrated pressure pain (50 mm	Seed-based pain-evoked connectivity analysis using rACC and thalamus seeds

Author / Year	Age (mean ± SD)	Pain duration	Pain intensity at baseline	FM severity / impact	Mood burden	Morphometry scope	Modality	Psychiatric comorbidities reported	Other comorbidities reported	Medication use reported / restricted	Clinical scales reported	Confounders controlled or matched?	Notes / NR
			previous week 72.3 (13.3) VAS mm					episode); serious suicide risk; substance/drug/alcohol abuse excluded	or renal disease; autoimmune disease; systemic infection; active cancer; unstable endocrine disease; severe sleep apnea excluded; controls excluded for any clinical pain problem	patches, TENS, biofeedback, tender/trigger point injections, acupuncture, and anesthetics withdrawn; analgesics prohibited except paracetamol, dipyrone, and NSAIDs as rescue; analgesic/narcotic drugs stopped 48 h; zolpidem allowed		VAS); movement, whole-brain, ventricular, and white-matter signals included as nuisance regressors	
Seo et al. / 2012	38.73±7.65 (controls 38.27±8.48)	39.41±43.90 months	NR	FIQ 59.37±19.89	BDI 23.21±10.59; BAI 29.79±8.45	NR	Task-based fMRI	NR	NR	Allowed / not controlled; 7 patients took antidepressants; 6 took pregabalin (75 mg) once daily; 1 took pregabalin (75 mg) plus milnacipran (25 mg) once daily; medication considered as possible confound and subgroup analyses found no activation differences at P<0.01 uncorrected	FIQ; BFI; BDI; BAI; tender point count; pressure-pain intensity (mild, moderate); task accuracy; response time; IQ	Age-matched; all female; right-handed; BDI and BAI entered as covariates; movement parameters entered as covariates of no interest; medication subgroup analyses performed	No between-group difference in deactivation network during the n-back task
Burgmer et al. / 2011	50.1 (7.3) years (controls 46.9 (6.8))	10.50 (6.26) years of widespread FMS pain (range 3–26)	NR	Pain Disability Index 47.7 (18.1); Hannover Functional Capacity Questionnaire 72.0 (15.0)	HADS total score 18.1 (7.1)	NR	Task-based fMRI	Free of any current Axis I psychiatric diagnosis	Other origins of pain excluded (e.g., rheumatic or endocrinologic diseases); controls free of any pain syndrome	Wide range of medication including steroids, opioids, nonsteroidal antirheumatics, tricyclic antidepressants, and SSRIs; medication altering pain perception or brain activation discontinued 48 h before scanning	CSQ (trait/state), HADS, Pain Disability Index, Hannover Functional Capacity Questionnaire, pressure pain NRS, tender point score	All participants female; HADS scores entered as control variable; trait catastrophizing added as control variable in repeated analysis	Pain anticipation paradigm with prior notification vs no prior notification; PAG also analyzed with ROI analysis
Diers et al. / 2011	54.67 (4.50) years (controls 50.33 (8.78))	21.67 (15.49) years (range 6–45)	Habitual pain intensity 3.8 (2.8) on 0–10 NRS before measurement	FIQ total 44.43 (5.87)	CESD 20.5 (11.48)	NR	Task-based fMRI	No current major depression or any other Axis I or II mental disorder; HC did not fulfill any DSM-IV Axis I or II disorder	Neurological complications, pregnancy, cardiac pacemaker, and allergy to plaster excluded	No opioid medication; one NSAID; three antidepressants (1 SSRI, 1 tricyclic, 1 tetracyclic); patients asked not to take pain medication the day before and if possible 3 days before measurement; antidepressants not interrupted	WHYMPI, Pain-related self-statements scale, FIQ, grade of chronicity screening, CESD, continuous VAS, 11-point NRS, tender points	All participants female; no significant age difference between groups; exclusion criteria applied	Final analyzed sample was 6 FMS and 6 HC because 2 FMS and 4 HC felt no sensation and could not be analyzed; sustained pain was prolonged in FMS
Glass et al. / 2011	43.6 (9.79) (controls 41.13 (11.91))	9.1 years	Average Pain Intensity (PED) 55.4 (15.5)	NR	Depressive Symptoms (CES-D) 16.28 (9.31); Anxiety (STPI) 20.1 (5.9)	NR	Task-based fMRI	Psychosis, current suicide risk/attempt within 2 years, and substance abuse within 2 years excluded	Severe physical impairment/co-existing physical injury; morbid obesity, autoimmune diseases, cardiopulmonary disorders, uncontrolled endocrine or allergic disorders, malignancy within 2 years	NR	PED; BPI body map (% Body Distribution); CES-D; STPI; CMSI; MFI (mental fatigue); MOS Sleep Scale (somnolence); MASQ; Go/NoGo reaction time and false alarms	Age-matched HCs; all participants female; age as covariate; age and anxiety as covariates; motion parameters modeled as regressors of no interest	Performance was not different between FM and HC in reaction time or false alarms on the Go/NoGo task
Burgmer et al. / 2010	52.6 ± 7.9 (controls 49.5 ± 8.9; RA 47.7 ± 6.5)	NR	Clinical pain pre (VAS) 31.2 ± 24.4	PDI score 26.4 ± 13.4; FFbH (% of functioning) 70.2 ± 17.3	HADS 21.2 ± 7.9	NR	Task-based fMRI	HADS measured; healthy controls were free of any current Axis I psychiatric diagnosis	Other origins of pain excluded (e.g., rheumatic or endocrinologic diseases)	Medication that may alter pain perception or brain activation (e.g., pain medication, anxiolytics, antidepressants) discontinued 48 h prior to functional scanning	PDI; FFbH; HADS; anxiousness VAS; clinical pain VAS; experimental pain NRS; SF-MPQ	HADS, pre-anxiety, and clinical pain included as nuisance variables	Included a rheumatoid arthritis comparator group; reported FMS-unique temporal brain activation of the frontal cortex
Renz et al. / 2025	52.07 ± 8.82	NR	61.67 ± 16.23 (Pain intensity [CPG])	NR	BDI-II 21.64 ± 8.89; HADS-A 9.52 ± 3.47	NR	Task-based fMRI	MDD allowed/not excluded; N = 12 of 46 (26.1%) of FMS additionally fulfilled diagnostic criteria of MDD	NR	NR	Beck Depression Inventory-II (BDI-II); Hospital Anxiety and Depression Scale (HADS); Cognitive Emotion Regulation Questionnaire (CERQ); Perceived Stress Scale (PSS); West-Haven Yale Multidimensional Pain Inventory; Chronic Pain Grade Scale; Widespread Pain Index (WPI); Somatic Symptom Disorder B Criteria Scale 12 (SSD-12); Whiteley Index; WHODAS 2.0; Pain Catastrophizing Scale (PCS); Toronto Alexithymia Scale (TAS-20); STAI-X2; PANAS; EMA pain/mood measures	Age and sex used as covariates in psychometric ANCOVAs, multilevel models, and fMRI ANCOVA	Transdiagnostic FMS/MDD study; FMS showed unique right amygdala regulation deficits (P = 0.004)
Park et al. / 2022	35.9 ± 12.37 (controls 44.25 ± 12.1)	6.03 years (SD = 5.33); range 9	4.5 ± 1.9 (Pain Severity [BPI])	12.05 ± 3.8 (Number of	BDI 16.7 ± 8.7; STAI-Trait 48.2 ± 10.4;	NR	Task-based fMRI	Uncontrolled depression or anxiety excluded; mood	NR	Patients were opioid-naïve/no opioids before study >90 days; non-opioid and mood-altering medications allowed	BDI; STAI-State; STAI-Trait; BIS/BAS; POMS; PANAS; BPI;	All participants female; primary analyses used one-way ANCOVA including age as a continuous	Replication MID task study; MPFC reduced during gain anticipation and increased

Author / Year	Age (mean ± SD)	Pain duration	Pain intensity at baseline	FM severity / impact	Mood burden	Morphometry scope	Modality	Psychiatric comorbidities reported	Other comorbidities reported	Medication use reported / restricted	Clinical scales reported	Confounders controlled or matched?	Notes / NR
		months to 20 years		Pain Areas [FAF])	STAI-State 38.7 ± 9.3			symptoms measured with BDI/STAI		and listed (NSAIDs, acetaminophen, SNRIs, SSRIs, TCAs, anxiolytics, triptans, trazodone, antiepileptic, muscle relaxants, GABA analogs, benzodiazepine, NDRI); controls took no pain or mood-altering medications	PROMIS Fatigue; BSI; cue arousal/valence ratings	covariate; motion censoring applied (>0.5 euclidean norm); target duration adjusted to ~66% hit rate	during no-loss outcome; NAcc gain anticipation activity did not differ
Lofgren et al. / 2023	51 y (median, 25–75%; EIH cohort); fMRI subsample 50 y (median, 25–75%) (HC 56 y; fMRI HC 55 y)	9 years (median; EIH cohort); fMRI subsample 8 years (median)	VAS pain 54.0 (33.7–70.2) (EIH cohort); fMRI subsample 38.0 (28.5–54.8)	FIQ total 59.8 (50.1–73.6) (EIH cohort); fMRI subsample 63.9 (52.8–73.8)	HADS-D 7.0 (5.0–8.2); HADS-A 8.0 (4.0–11.0) (EIH cohort); fMRI subsample HADS-D 6.5 (5.0–10.2), HADS-A 9.0 (6.2–10.0)	NR	Task-based fMRI	Severe psychiatric disorders excluded	High blood pressure, osteoarthritis hip/knee, other severe somatic disorders, other primary causes of pain excluded	Needed to refrain from analgesics/NSAIDs/hypnotics; declared refraining from hypnotics, NSAIDs, acetaminophen and tramadol/other analgesics 48 h before EIH and 72 h before fMRI	VAS pain; FIQ; HADS; PPTs; EIH; P50	Women only; age included as covariate of no interest across all analyses; head motion assessed with FD >0.5 in >15% images; none excluded for excessive movement in both runs	Resistance exercise arm only; fMRI performed only in Stockholm subsample; resting state data collected but published elsewhere
Balducci et al. / 2022	41.7 (6.1) (HC 41.5 (6.0))	NR	NR	NR	NR	NR	Structural MRI; Resting-state fMRI; Task-based fMRI	Major psychiatric disorders excluded; depression and anxiety allowed	Cardiovascular disease, neurological illness, other pain conditions if worse than FM pain excluded; migraine, tension-type headache, systemic lupus erythematosus, systemic hypertension excluded; neuropathic pain and irritable bowel syndrome allowed	Opioids excluded; participants needed to stop rescue-dose analgesic or benzodiazepine for at least 24 h before MRI; current medication assessed but not reported in detail	Tender points assessment; WPI; Symptom Severity Scale; MINI-Plus; Hamilton Depression Rating Scale; Hamilton Anxiety Rating Scale; Emotion Regulation Questionnaire; Toronto Alexithymia Scale; PANAS; Inventory of Personality Organization; FIQ; McGill Pain Questionnaire; Fibromyalgia general questionnaire; task VAS ratings (affective state, valence, arousal, pain); fatigue/performance VAS	All participants were women and right-handed; groups matched for age and education; motion/outlier thresholds and motion regression applied in preprocessing/denoising	Open dataset paper; MRI data presented in BIDS/OpenNeuro format; emotion processing and regulation task with Attend/Reappraise/Suppress conditions
Ioachim et al. / 2022	46 ±13 years (controls 39 ±10 years)	NR	Initial pain score 33.9 (23.7) (controls 2.3 (5.62))	FIQR total 50.26 (3.66)	BDI 16.26 (2.77)	NR	Task-based fMRI	Severe psychiatric illness excluded	NR	Not taking centrally-acting medications; other medications allowed if taken for at least 3 months prior; participants were not asked to stop ongoing non-centrally-acting medication	2016 FSQ; STAI; BDI; SDS; PCS; COMPASS-31; FIQR; SF-MPQ-2; tender point test; NPS 0–100	All participants were women; MRI contraindications excluded; centrally-acting medications excluded; ANCOVA included normalized pain scores as a continuous variable	Brainstem and spinal cord fMRI at 3 tesla; SEM connectivity analysis
Fanton et al. / 2022	47.3 (7.8) (controls 48.1 (7.6))	FM duration 121.3 (87.6, 11, 408)	VAS current 53.5 (22.1, 6, 99)	FIQ 63.5 (16.4, 13, 95)	HAD-A 7.8 (4.3, 0, 21); HAD-D 7.4 (4.1, 0, 18)	NR	Task-based fMRI	Severe psychiatric disorders requiring treatments for depression or anxiety excluded	Rheumatic or autoimmune diseases; severe somatic diseases; other dominant pain syndromes than FM; previous heart or brain surgery; hypertension	Anticonvulsants or antidepressants excluded; inability to refrain from hypnotics, NSAIDs, or analgesics before participation excluded; 48 h before first visit and 72 h before neuroimaging	FIQ; VAS current; VAS past week; SF-36BP; HADS; PCS; PPTmean; CPM; P10/P50/P30 pain ratings	Age-balanced healthy controls; all women/right-handed/working age; motion exclusions (FD > 0.5 in ≥15% of images); six motion parameters as regressors of no interest	Multimodal neuroimaging: single-voxel 1H-MRS in rACC and bilateral thalamus plus task-based fMRI
Kim et al. / 2020	NR	NR	NR	NR	NR	NR	Task-based fMRI	NR	NR	Restricted: routine moderate-to-high opioid use (>60 mg morphine equivalents); benzodiazepines excluded except alprazolam, lorazepam, and diazepam	Visual analog scale for pain; Beck Depression Inventory (BDI-1A); Snaith-Hamilton Pleasure Scale (SHAPS)	Age and sex used as covariates of no interest; six head motion parameters modeled as regressors of no interest	Mixed chronic musculoskeletal pain sample; primary analyses combined CLBP and FM into a single "Pain" group (CLBP n=15; FM n=13)
Martucci et al. / 2019	Non-opioid FM: 48.1 ± 9.6; opioid FM: 52.8 ± 6.9 (controls 48.1 ± 10.2)	Non-opioid FM: 11.5 ± 7.7 years; opioid FM: 10.0 ± 7.0 years	Pain Severity (BPI): non-opioid FM 5.7 ± 2.1; opioid FM 6.0 ± 1.5	Fibromyalgia Assessment Form / Number of Pain Areas (FAF): non-opioid FM 13.9 ± 3.9; opioid FM 12.6 ± 3.7	Trait Anxiety (STAI): 49.7 ± 8.5 / 51.9 ± 12.4; State Anxiety (STAI): 41.4 ± 7.0 / 41.9 ± 12.8; Depression (BDI): 15.8 ± 8.9 / 15.2 ± 9.3	NR	Task-based fMRI	Depression/anxiety not uncontrolled; controls required to have no depression or anxiety	NR	Allowed: normal medication use continued; opioid group required opioid treatment ≥3 months; non-opioid group no opioids in prior 90 days and never >30 days; classes reported included opioids, NSAIDs, acetaminophen, SNRIs, SSRIs, tricyclic antidepressants, other anxiolytics, antiepileptics, triptans, benzodiazepines, benzodiazepine-like drugs, muscle relaxants, GABA analogues, low-dose naltrexone, medical cannabis, SARI, NDRI, ondansetron	PANAS; BAS/BIS; POMS; PROMIS Fatigue; STAI; BDI; Fibromyalgia Assessment Form (FAF); Brief Pain Inventory (BPI); post-scan arousal and valence ratings	All participants female; participants with incomplete scans, artifacts, or excessive head motion were excluded	Two fibromyalgia subgroups were analyzed separately by opioid status (17 taking opioids; 17 not taking opioids)
Martinsen et al. / 2018	49.6 years (range 25–64) (controls 47.2 years,	8 years (range 1–16)	44.6 ± 17.5 (Pain VAS)	59.9 ± 16.0 (FIQ total)	HAD-D 11.3 ± 11.1; HAD-A 8.63 ± 4.4	NR	Task-based fMRI	Other severe psychiatric disorders excluded; HADS measured	High blood pressure (>160/90 mmHg), osteoarthritis in hip or knee, other primary causes of pain than FM,	Analgesics/NSAIDs/hypnotics restricted; patients refrained from hypnotics, NSAIDs, acetaminophen, and tramadol/other analgesics at least	SF-36; HADS; 100-mm VAS; FIQ; PPT; SCWT reaction times	All participants were women; movement parameters entered as covariates of no interest; exclusion criteria handled psychiatric/somatic conditions and medication use	15-week resistance exercise intervention; increased bilateral amygdala activation post-intervention; no effect on DIA

Author / Year	Age (mean ± SD)	Pain duration	Pain intensity at baseline	FM severity / impact	Mood burden	Morphometry scope	Modality	Psychiatric comorbidities reported	Other comorbidities reported	Medication use reported / restricted	Clinical scales reported	Confounders controlled or matched?	Notes / NR
	range 20–63)								other severe somatic disorders excluded	48 h before study participation and 72 h before fMRI			
Harte et al. / 2016	40.9 ± 10.7 (controls 41.4 ± 11.9)	>6 months (disease duration criterion)	48.2 ± 23.3 (VAS at baseline)	NR	NR	NR	Task-based fMRI	Psychiatric illness excluded (eg, current schizophrenia, major depression with suicidal ideation, or substance abuse within the past 2 years)	Concurrent autoimmune or inflammatory disease causing pain (eg, rheumatoid arthritis, systemic lupus erythematosus, inflammatory bowel disease), chronic medical illness, chronic pain disorder, migraine headaches, pregnancy/currently nursing excluded	Current use/history of opioid or narcotic analgesics, sedatives, hypnotics, unstable doses of antidepressants, NSAIDs, or muscle relaxants excluded; pregabalin nonresponders to ≥300 mg/d excluded for pharmacologic subset	100-mm VAS; Gracely Box scale (GBS); Pain50; PPT; pressure pain tolerance; pain sensitivity range (PSR)	Healthy controls were age- and sex-matched; BOLD activation responses were corrected for age and scanner; motion thresholds set at ±2 mm translation/±1° rotation; participants exceeding thresholds were excluded	Pregabalin vs placebo crossover subset (n=17); visual-evoked brain activity classified FM vs HC and pregabalin vs placebo with 82% accuracy
Kamping et al. / 2013	53.00 years, SD 7.10 years (controls 51.25 years, SD 8.51 years)	23.21 years (SD 15.20 years, range 5 to 60 years)	NR	FIQ sum score 43.82 (SD 12.91, range 21.55 to 64.34)	CESD 16.75 (SD 8.46, range 4.00–30.00)	NR	Task-based fMRI	Current MDE and any other current mental disorder excluded; lifetime MDE present in 8 FMS patients (2 controls)	No control subject reported recurring pain episodes (e.g., migraine, back pain)	All FM patients except 2 discontinued usual pain medication and were at least 3 days without medication; 2 continued duloxetine or citalopram	CESD; MPI; FIQ; Pain-Related Self-Statements Scale; tender point examination; WPI; Symptom Severity Scale; picture valence/arousal ratings; pain intensity and unpleasantness VAS; laser pain rating scale (0–10)	Matched for age, gender, and educational status; movement parameters included as parameters of no interest; no group differences in movement	All participants female; FM patients showed deficient modulation of pain in a positive emotional context
Lee et al. / 2013	38.0 ± 7.3 years (controls 37.3 ± 8.1 years)	27.7 ± 36.7 months (median 12.0 month)	NR	KFIQ 56.4 ± 19.5	BDI 21.6 ± 9.9; BAI 29.4 ± 8.1	NR	Task-based fMRI	NR for FM psychiatric diagnoses/comorbidities; controls had no current psychiatric diagnosis	History of head injury or other neurological condition, medical condition associated with cognitive dysfunction, and substance abuse excluded	Patients were on stable doses of medication and instructed not to take any medication on the test day; controls took no psychoactive medication	KFIQ; tender points; BDI; BAI; BFI-K; pressure pain threshold by thumbnail pressure; Wong-Baker FACES Pain Rating Scale; Verbal-VAS adapted from Gracely Box scale	Right-handed female participants; menstrual cycle timing controlled; depression and anxiety scores controlled as covariates in within- and between-group analyses	Patients with FM did not show enhanced neural responses to observation of pain in others
Kim et al. / 2013	51.3 ± 8.4 (controls 46.5 ± 12.0)	NR	NR	FIQ 65.98 ± 18.11	BDI 41.10 ± 12.17; STAI1 44.10 ± 8.46; STAI2 47.05 ± 7.58	NR	Task-based fMRI	NR	NR	Pregabalin pharmacological therapy; other medication restrictions NR	FIQ; BFI; BDI; WPI; SSS; STAI1; STAI2; VAS; GBS	Age- and gender-matched healthy controls	Only responders underwent follow-up fMRI; 7 of 9 responders had post-treatment MRI

Diffusion / Structural Connectivity (additional)

Author / Year	Modality	Psychiatric comorbidities reported	Other comorbidities reported	Medication use reported / restricted	Clinical scales reported	Confounders controlled or matched?	Age (mean ± SD)	Pain duration	Pain intensity at baseline	FM severity / impact	Mood burden	Brain morphometry scope	Notes / NR
Mosch / 2023	Structural MRI; DTI	Previous major depressive episodes reported (16); history of generalized anxiety disorder and/or PTSD in 4; acute major depression, bipolar disorder, psychosis, and psychotropic medication excluded; depression symptoms assessed with CES-D	Other current autoimmune or inflammatory diseases causing pain excluded (rheumatoid arthritis, systemic lupus erythematosus, chronic inflammatory bowel disease)	No pain medication on examination day; opioid use suspended no later than 3 days prior to MRI; users of psychotropic medication excluded	MPI; FIQ-G; FSQ; CES-D; EHI; SKID-I; heat pain threshold	Female-only sample; left-handed persons excluded; age included as covariate; TIV included as covariate in VBM	50.5 ± 9.9 (HC 46.6 ± 13.1)	14.9 ± 11.8 years	Pain severity (MPI) 4.0 ± 15.8 (as reported)	FIQ total 60.2 ± 17.6	CES-D 22.1 ± 6.5	Both (whole-brain VBM + thalamus, amygdala, putamen, pallidum, caudate nucleus, SMA, MTG, cerebellum, insular, parahippocampal, prefrontal, orbitofrontal, cingulate, SI, SII cortices)	One FM patient excluded due to insufficient DTI data quality; structured clinical interview used to rule out acute mental illness
Aster / 2022	Structural MRI; DTI; Resting-state fMRI	Psychiatric diseases, such as major depression, excluded (past and present); depressiveness assessed with ADS; anxiety assessed with STAI	Other current autoimmune or inflammatory diseases that can cause pain excluded (rheumatoid arthritis, systemic lupus erythematosus, chronic inflammatory bowel disease)	Off pain medication for 3 days before examination; no anticonvulsants, antihistamines, muscle relaxants or benzodiazepines within 4 weeks before examination	GCPS; NPSI; ADS; PCS; STAI; FIQ; SSS; O'Leary-Sant Symptom and Problem Index; WPI; tender points; time since diagnosis; duration of pain due to the disease	Healthy controls age- and sex-matched; women only; age and pain intensity (GCPS) used as covariates in structural analyses; pain intensity (GCPS) and ADS used as second-level covariates in resting-state analyses; motion thresholds applied	53.5 ± 6.5 (controls 52.6 ± 6.7)	PNS: 16.8 ± 10.8 years; noPNS: 18.8 ± 12.7 years	GCPS pain intensity — PNS: 73.6 ± 10.8; noPNS: 64 ± 15.1	FIQ — PNS: 51.9 ± 12; noPNS: 42.3 ± 13.2	ADS — PNS: 27.8 ± 11.8; noPNS: 21.2 ± 11.4; STAI — PNS: 47.1 ± 11.6; noPNS: 44.3 ± 13.3	Both (whole-brain approach + left medial frontal cortex, right posterior cingulate cortex)	Subgrouping by intraepidermal nerve fiber density (PNS vs noPNS)
Fayed / 2010	DTI	Axis I psychiatric disorders excluded (depressive disorder, bipolar disorder, dementia, schizophrenia, paranoid disorder, alcohol/drug-abuse disorders); neither group had any psychiatric disorder by SPPI; anxiety and	NR	Pharmacologic treatment discontinued 1 week before study or modified not to affect brain imaging; all FM patients took tramadol, pregabalin, or paracetamol; some took duloxetine and benzodiazepines; during washout	PCS; HADS; PVAS; pain threshold assessed by sphygmomanometer; MMSE; FIQ; duration of disorder	Controls matched for gender and age (± 3 years) by 'head-by-head' method; Axis I psychiatric disorders excluded; no significant age difference because of matching process	40.0 (SD = 6.2) years (controls 37.8 (SD = 8.7) years)	1.6 (SD = 0.3) years	PVAS 63.0 (SD, 17.0)	FIQ 70.20 ± 10.50	HADS-Anx 7.40 ± 0.69; HADS-Dep 6.90 ± 0.87	NR	Controlled cross-sectional study; study also performed proton MRS, DWI, and task-based fMRI for motor cortex localization; narrative text

Author / Year	Modality	Psychiatric comorbidities reported	Other comorbidities reported	Medication use reported / restricted	Clinical scales reported	Confounders controlled or matched?	Age (mean ± SD)	Pain duration	Pain intensity at baseline	FM severity / impact	Mood burden	Brain morphometry scope	Notes / NR
		depression symptoms assessed with HADS		week only occasional tramadol or paracetamol allowed									reports HADS means that differ from Table 1
Tu / 2023	DTI	Anxiety and depression measured with STAI and CES-D; no psychiatric exclusion reported for FM in Methods	Unmanaged endocrine issues, neurological diseases, auditory diseases, and pregnancy excluded	On the day of the scan, individuals with FM were instructed not to use painkillers	VRS; PCS; PSP; STAI; CES-D; disease duration	Healthy controls age-matched; age included as nuisance covariate and regressed out in network analyses; HCs checked to verify that none reported any form of pain	46.4 ± 12.4 years (HC 42.1 ± 12.5)	5.2 ± 5.1 years	VRS 7.2 ± 1.6	NR	STAI 52.8 ± 20.1; CES-D 31 ± 13.7	NR	Whole-brain graph-theoretical probabilistic diffusion tractography study; female-only sample; public OpenNeuro dataset ds001928

Resting-state fMRI

Author / Year	Age (mean ± SD)	Pain duration	Pain intensity at baseline	FM severity / impact	Mood burden	Morphometry scope	Modality	Psychiatric comorbidities reported	Other comorbidities reported	Medication use reported / restricted	Clinical scales reported	Confounders controlled or matched?	Notes / NR
Liu et al., 2024	38.97±11.68	1.75 [0.5-5]	4.5 [3–6] (Current VAS, 0–10)	WPI 9.17±2.12; SSS 7.31±1.69	HADS-anxiety 9.14±3.56; HADS-depression 8.26±3.12	Whole-brain ("voxel-based morphometric analysis using FSL's FSL-VBM"; "voxel-wise GLM")	Structural MRI; Resting-state fMRI	Anxiety and depression measured with HADS; psychiatric diagnostic comorbidities/exclusions NR	NR	Stable preventative or as-needed medication allowed (NSAIDs); previous medication recorded, including NSAIDs, duloxetine, pregabalin, and sedatives	VAS (current pain), global pain, WPI, SSS, PSQI, HADS, FSS, disease duration	Age and sex included as regressors/covariates of no interest; TIV additionally included in VBM; HC pain-related conditions/pain medication excluded	FM n=38 in final analysis; FM, AS, and HC cohorts; 3.0T MRI
Gurevitch et al., 2024	NR	NR	NR	NR	NR	NR	Structural MRI; Task-based fMRI	NR	NR	NR	TAS-20, STAI, BDI	Possible protocol/training-length effects handled with LME model; group as fixed effect; subject and study as random effects; first-level fMRI models included motion, WM/CSF, and scrub regressors	Mixed sample (HC, PTSD, FM); FM n=19; Amyg-EFP neurofeedback study with post-training fMRI-NF
Lim et al., 2023	49.3 ± 9.0	at least one year	5.1 ± 2.3 (VAS)	NR	NR	NR	Resting-state fMRI	History of psychiatric disease excluded (e.g., "major depressive disorders"); substance abuse excluded	Co-existing autoimmune or chronic inflammatory disease that causes pain excluded (e.g., rheumatoid arthritis)	No new medication during study; currently taking opiates excluded	VAS, McGill Pain Questionnaire	Female HC comparison; no significant group age difference; age and mean FD included as covariates of no interest	Within-subject cross-over sham-controlled tDCS study; 12 female FM completed; baseline comparison with 15 female HC; 3.0 T MRI
Aster et al., 2022	PNS 53.5 ± 6.7; noPNS 53.4 ± 6.5 (patients all: 53.5 ± 6.5)	PNS 16.8 ± 10.8; noPNS 18.8 ± 12.7	GCPS pain intensity: PNS 73.6 ± 10.8; noPNS 64 ± 15.1	FIQ: PNS 51.9 ± 12; noPNS 42.3 ± 13.2	ADS: PNS 27.8 ± 11.8; noPNS 21.2 ± 11.4; STAI: PNS 47.1 ± 11.6; noPNS 44.3 ± 13.3	Both (exploratory whole-brain approach + left medial frontal cortex and right posterior cingulate cortex)	Structural MRI; DTI; Resting-state fMRI	Psychiatric diseases excluded (e.g., major depression); depressiveness assessed with ADS; anxiety assessed with STAI	Current autoimmune/inflammatory pain diseases excluded (rheumatoid arthritis, systemic lupus erythematosus, chronic inflammatory bowel disease); neurological and cardiovascular diseases excluded; bladder dysfunction symptoms assessed with O'Leary-Sant	Off pain medication for 3 days; no anticonvulsants, antihistamines, muscle relaxants, or benzodiazepines within 4 weeks	GCPS, NPSI, ADS, PCS, STAI, FIQ, SSS, O'Leary-Sant Symptom and Problem Index, WPI, tender points, time since diagnosis, duration of pain, HbA1c, sural SNAP, sural nerve conduction velocity, vitamin D	Healthy controls age- and sex-matched; age and GCPS pain intensity used as covariates in structural analyses; GCPS pain intensity and ADS used as second-level covariates in FC; participant motion parameters included as first-level covariates; motion-threshold exclusion	Prospective case-control study; female-only sample; subgrouped by IENFD into PNS/noPNS; 43 FMS and 40 HC; 3 T MRI
Kim et al., 2021	44.9 (8.3)	35.6 (31.1) months	52.8 (20.3) (VAS—last week)	NR	BDI 19.0 (6.8)	Both (region-specific left posterior thalamus [pulvinar and VPL nuclei] + whole-brain cortical thickness/structural covariance/seed-based functional connectivity)	Structural MRI; Resting-state fMRI	Psychiatric disorders excluded (major depressive disorder, schizophrenia, substance abuse); depressive symptoms measured with BDI	Secondary FM associated with inflammatory arthritis excluded; CNS disorders excluded (cerebrovascular accident, multiple sclerosis, Parkinson's disease); peripheral neuropathy and acute upper-extremity pain excluded	Medications influencing the somatosensory system stopped at least 3 days before assessment (analgesics, antidepressants, anticonvulsants); baseline medication classes reported: analgesics/muscle relaxants/NSAIDs, antidepressants, anticonvulsants	BDI, VAS, SF-MPQ, PPT, EPT, pain duration, tender point count	HCs age-, sex-, and education level-matched; age and intracranial volume used as covariates in thalamic volume analyses; age and average thickness used in cortical analyses; age used as a covariate in FC analyses	Right-handed female primary FM sample; 19 FM and 21 HC overall; rs-fMRI subset 12 FM and 20 HC; 3T MRI
van Ettinger-Veenstra et al., 2020	39.23 11.44	NR	5.68 1.83 (current pain intensity, NRS 0–10)	NR	HADS-depression 6.00 3.61; HADS-anxiety 7.81 4.04	NR	Resting-state fMRI	Severe psychiatric condition excluded; anxiety and depressive symptoms measured with HADS-A/HADS-D	Rheumatoid arthritis, metabolic disease, neurologic disease, malignancy, cardiovascular disease, unregulated thyroid disease, and lung disease excluded	NSAID, pain, and sleep medication washed out for 48 h before visits; patients continued standard therapy; no experimental treatment	Numeric rating scale (pain intensity), PSQ, PPT, HADS-D, HADS-A, HADS total, PCS total/subscales, PDI	Female age-matched HC; seed-target ANCOVA included HADS and PPT as regressors; within-FM regression included HADS, PPT, and pain intensity; motion, scrubbing, white matter, and CSF handled in preprocessing	Female-only sample; final sample 31 FM and 28 HC; 3T Prisma scanner
Jung et al., 2020	41.07 ± 11.64	diagnosis for at least one year	Pain severity (BPI) 5.37 (1.50)	FIQ 50.18 (18.55)	Depression (HADS) 7.26 (3.55); Anxiety (HADS) 7.68 (4.26)	NR	Resting-state fMRI	Severe psychiatric illnesses excluded ("current schizophrenia, major depression with suicidal ideation"); depression and	Concurrent autoimmune or inflammatory disease and peripheral neuropathy excluded; history of head injury with substantial loss of consciousness excluded	NSAIDs and FDA-approved FM medications (pregabalin, duloxetine) allowed; OTC pain medications on scan day and narcotic pain medications within 48 h excluded; routine daily narcotic	BPI (pain severity, pain interference), FIQ, HADS	Female age-matched HC; all subjects right-handed; analyses controlled for age and pregabalin status; pregabalin/gabapentin treatment controlled in analyses	Female-only sample; 43 FM and 16 HC enrolled, final rs-fMRI analysis 38 FM and 16 HC after motion exclusion; multimodal study also included 1H-MRS

Author / Year	Age (mean ± SD)	Pain duration	Pain intensity at baseline	FM severity / impact	Mood burden	Morphometry scope	Modality	Psychiatric comorbidities reported	Other comorbidities reported	Medication use reported / restricted	Clinical scales reported	Confounders controlled or matched?	Notes / NR
								anxiety severity assessed with HADS		analgesics, marijuana, and stimulant medications excluded; pregabalin/gabapentin use reported			
Pando-Naude et al., 2019	46.4 ± 12.4	NR	NR	NR	STAI 52.8 ± 20.1; CES-D 31 ± 13.7	NR	Resting-state fMRI	NR for psychiatric diagnoses/comorbidities; anxiety and depression symptoms measured with STAI and CES-D ("not used for diagnosis")	NR	FM patients were asked not to intake painkillers on the day of testing only	PCS, STAI, PSP, CES-D, VRS pain intensity, VRS pain unpleasantness	Age-matched HC; all participants women and right-handed; baseline GLM included age, years with FM diagnosis, and anxiety and depression symptoms as covariates	20 FM and 20 HC; women-only sample; 3T MRI; music vs pink-noise design with four rs-fMRI acquisitions
Jarrahi et al., 2018	40.36 ± 9.91 years	NR	NR	NR	NR	NR	Resting-state fMRI	NR	NR	NR	0-10 pain intensity scale (average pain >2), VAS (target pain rating 3 for cold pressor calibration), pain duration >6 months	Right-handed female FM and HC groups; no explicit matching or covariate adjustment reported	8 FM and 11 HC; cold water pressor test during resting-state scan; 3T MRI
Elkana and Beheshti, 2025	43.00 [37.00, 47.00]	Disease duration, years: 2.00 [1.00, 6.00]	Pain intensity during interview (range 0–100): 47.00 [38.00, 66.00]	FIQ total score (range 0–100): 35.47 [29.99, 39.97]	HAM-D total score (range 0–51): 16.00 [11.00, 21.00]; HAM-A total score (range 0–56): 23.00 [17.00, 27.00]	Both (whole-brain VBM/DBM + cortical thickness measurements from the DKT atlas)	Structural MRI; Resting-state fMRI	Psychological/psychiatric assessments collected: MINI-Plus, Hamilton Depression Rating Scale, Hamilton Anxiety Rating Scale; depression and anxiety symptoms compared	NR	Number of medications currently taken daily and during a crisis recorded; no restrictions/washout reported; medication effects not accounted for	WPI, SSS, Fibromyalgia General Questionnaire, FIQ, MINI-Plus, Hamilton Depression Rating Scale, Hamilton Anxiety Rating Scale, Toronto Alexithymia Scale, Emotional Regulation Questionnaire, PANAS, McGill Pain Questionnaire	33 matched healthy female controls; age and TIV covariates in VBM; age covariate in cortical thickness and FC analyses; partial correlations adjusted for age; age regressed out during feature selection	Secondary analysis of Mexican dataset; 33 female FM and 33 matched female HC
Jarrahi et al., 2017	40.36 ± 9.91 years	NR	NR	NR	NR	NR	Resting-state fMRI	Severe psychiatric conditions excluded	NR	Opioid medications excluded	Average pain intensity (0-10), CPT pain rating (0-10), symptom duration >6 months	Right-handed female sample; frame-wise displacement included as a nuisance regressor; previous head injury and MRI incompatibility excluded	8 FM and 11 HC after exclusion for motion/image artifacts; cold pressor paradigm; 3.0T MRI
Coulombe et al., 2017	50.6 ± 8.1 years	NR	Pain severity (BPI, 0–40): 22.3 ± 7.5	FIQ total score (Normalized, 0–100): 60.3 ± 15.8	HADS Anxiety: 10.4 ± 3.8; HADS Depression: 7.3 ± 3.4	NR	Resting-state fMRI	Active psychosis excluded; concurrent antidepressant treatment excluded; depressive mood and anxiety assessed with HADS	HC with chronic illness excluded (including neurological disorders); FM-specific non-psychiatric comorbidities NR	Concurrent antidepressant treatment excluded; recent change in pain medication (dose alternation within preceding month) excluded	BPI, FIQ, HADS, PCS, PDI, DN4, EQ-5D	Age- and sex-matched HC; first five WM/CSF components and six motion parameters regressed out as nuisance components	23 female FM and 16 HC; 3T MRI; PAG seed-based rs-fMRI study
Truini et al., 2016	28-67 years	NR	NR	NR	NR	NR	Resting-state fMRI	Psychiatric diseases excluded, including major depression; depressive/anxiety symptoms assessed with ZSDS and ZSAS	Autoimmune and rheumatic diseases excluded; other/additional pain sources excluded, including osteoarthritis; neurological diseases excluded	No pain medications potentially affecting PAG connectivity (antidepressants, opioids, antiepileptics); rescue analgesics avoided 72 h before fMRI	Manual Tender Point Survey, ZSDS, ZSAS, VAS, disease duration	Healthy subjects matched ("15 healthy, matched subjects"; figure: "15 age and gender matched healthy controls"); pregnancy excluded for patients and controls	20 FM (19F/1M) and 15 HC (13F/2M); PAG seed-based study; 3 Tesla Siemens-Verio scanner
Ichesco et al., 2016	38.5 ± 12.1 years	NR	VAS Pre Scan 52.6 ± 20.5	NR	HADS Anxiety 10.0 ± 4.1; HADS Depression 6.9 ± 3.9; PANAS Positive Affect 28.6 ± 8.9; PANAS Negative Affect 23.2 ± 7.9	NR	Resting-state fMRI	Psychiatric illness excluded (schizophrenia, major depression with suicidal ideation, substance abuse within the past 2 years); anxiety and depression measured with HADS	Concurrent autoimmune/inflammatory disease contributing to pain excluded (rheumatoid arthritis, systemic lupus erythematosus, inflammatory bowel disease); HC with chronic medical illness/chronic pain excluded	New medications/treatment introductions limited during study; current or history of opioid/narcotic analgesics excluded; over-the-counter analgesics withheld at least 8 h before study visit	VAS, PANAS, HADS, NRS, Pain40	Age-matched healthy women; all participants right-handed and female; motion, white matter, and CSF were first-level regressors of no interest; age and scanner were second-level covariates	12 FM and 15 HC; resting-state scans before and after experimental pressure pain; 3.0T GE Discovery and GE Signa scanners
Agoalikum et al., 2025	46.4±12.5 (HC: 42.1±12.5)	5.2±5.0 years	PI 7.2±1.6 (verbal rating scale)	NR	STAI 52.8±20.0; CESD 30.6±13.7	Whole-brain ("whole-brain voxel-based morphometric comparison")	Structural MRI; Resting-state fMRI	Anxiety and depression symptoms measured with STAI and CESD; psychiatric diagnoses/comorbidities NR	Uncontrolled endocrine problems, neurological diseases (e.g., stroke, epilepsy, traumatic brain injury), and auditory problems excluded; HC acute/chronic pain excluded (e.g., osteoarthritis)	Painkillers restricted on the day of testing only	Pain intensity (verbal rating scale), PCS, STAI, CESD, helplessness, pain magnification, rumination, PSP, FM years	20 female age-matched HC; all participants female and right-handed; age and TIV used as covariates in VBM; age regressed out in partial correlations; age and mean FD regressed out in GCA; motion-threshold exclusion (>2 mm or >2°)	Public post hoc analysis of OpenNeuro dataset; 20 female FM and 20 female HC; Rest and T1 scans before music/pink-noise condition; 3T MRI
Flodin et al., 2015	48.4 (range 25–64) years (HC: 41.8	7.3 years (SD = 4.0)	SF36BP 37.00 ± 9.70 (pre-treatment)	FIQ 60.8 ± 11.8 (pre-treatment)	NR	NR	Resting-state fMRI	NR	NR	NR	FIQ, SF36BP, FM duration	All-female FM and HC groups; analyses controlled for age and inter-individual differences in mean frame-wise displacement;	Longitudinal pre/post 15-week supervised exercise study; 14 FM and 11 HC completed; 3T MRI; resting-

Author / Year	Age (mean ± SD)	Pain duration	Pain intensity at baseline	FM severity / impact	Mood burden	Morphometry scope	Modality	Psychiatric comorbidities reported	Other comorbidities reported	Medication use reported / restricted	Clinical scales reported	Confounders controlled or matched?	Notes / NR
	years, range 20–63)											movement outliers regressed out (FD >0.5 mm or signal intensity changes >3 SD); one HC excluded for excessive head-motion	state scan acquired after task-fMRI sessions
Kim et al. / 2015	44.94±12.02 (HC: 44.21±14.26)	9.76±8.56 years (based on date of diagnosis)	Clinical pain at MRI scan (0-100) 29.9±22.6	NR	BDI 13.5±8.2; STPI Form Y measured (value NR)	NR	Resting-state fMRI; Task-based fMRI	Anxiety disorders/significant anxiety symptoms excluded; depression measured (BDI); anxiety measured (STPI Form Y)	Significant neurologic disorders, significant cardiac events, and head injury excluded	Current treatment with opioids excluded; current use of recreational drugs excluded; 49% on antidepressants (most commonly SNRIs or tricyclic antidepressants); muscle relaxants 16%; benzodiazepines 9%	PCS; BDI; BPI; 0-100 numeric pain rating scale; Gracely Box Scale (GBS); MRS pressure thresholds; temporal summation index; disease duration	HC in same age range; neither sex nor age distribution differed; PCS controlled for depression (BDI); white matter/CSF and realignment parameters as covariates of no interest; exclusions for neurologic, anxiety, cardiac, head injury, opioids, recreational drugs, chronic/acute pain in HC	Electrocardiography/HRV collected; block-design pain fMRI used as functional localizer for S1leg seed
Ichesco et al. / 2014	35.8 ± 12.0 (HC: 32.3 ± 11.3)	3.9 ± 3.7 yrs	SF-MPQ VAS 4.4 ± 2.3	NR	HADS (n=7) or CES-D (n=11) assessed; STPI Form Y assessed (quantitative values NR)	NR	Resting-state fMRI	Major depression excluded; psychiatric illness excluded (current schizophrenia, major depression with suicidal ideation, substance abuse within the past 2 years); depression measured (HADS or CES-D); anxiety measured (STPI Form Y)	Concurrent autoimmune or inflammatory disease causing pain excluded (rheumatoid arthritis, systemic lupus erythematosus, inflammatory bowel disease); co-morbid symptoms assessed with CMSI	Current use or history of opioid/narcotic analgesics excluded; participants excluded if new treatments were introduced between consenting and imaging time points	CMSI; VAS; SF-MPQ (PRI sensory/affective/total and VAS); HADS or CES-D; STPI Form Y; GBS; MRS pressure thresholds; disease duration	Age- and sex-matched HC; age controlled as covariate in two-sample t-tests and regression analyses; white matter, CSF signal, and realignment parameters as covariates of no interest; exclusions for opioid use, autoimmune/inflammatory disease, psychiatric illness, chronic medical illness in HC	All female; subset had been part of previous study using different methodologies
Flodin et al. / 2014	48.3 (range 25–64 years) (HC: 45.7, range 20–63 years)	7.6 years (SD = 3.8)	NR	FIQ 61.2 (SD = 13.3)	NR	NR	Resting-state fMRI	Depression mentioned as comorbidity; other severe psychiatric disorders excluded	High blood pressure, osteoarthritis in hip or knee, other primary causes of pain excluded	Analgesics, NSAID, sedatives/hypnotics restricted before assessment/scanning; 1 on anticonvulsants; 11 on antidepressants (4 tricyclic, 4 SSRI, 3 SNRI)	FIQ; SF-36; VAS; P50	Age- and sex-matched healthy controls; second-level analyses controlled for mean FD and age; exclusion criteria used	Baseline Stockholm cohort from multicenter study; ICA and fALFF also analyzed
Cifre et al. / 2012	52.3 (8.9) years (HC: 49.0 [12.1] years)	26.8 (17.4) y	WHYMPI pain intensity 4.5 (0.9)	NR	BDI 29.78 (12.07)	NR	Resting-state fMRI	Psychiatric diagnosis excluded; depression measured (BDI)	Neurological disease excluded; other origins of pain excluded	Reported: antidepressants (n=8), analgesics/relaxants/NSAIDs (n=4), anxiolytics (n=8)	BDI; WHYMPI (pain intensity, pain interference, affective distress, social support, life control, distracting responses, solicitous responses, punishing responses, household chores, activities away from home, outdoor work, social activities); pain beginning; pain duration	Right-handed patients and controls; external rheumatologist reviewed charts to exclude other origins of pain; neurological disease or psychiatric diagnosis excluded	Pain as major complaint required
Napadow et al. / 2010	38.9 ± 10.8 (HC: 36.1 ± 15.3)	Disease duration ≥ 1 year	Spontaneous pain at time of scan 4.8 ± 2.4 (0-10 verbal analog scale)	NR	NR	NR	Resting-state fMRI	Current major depression and severe psychiatric illnesses excluded; depression assessed with HADS or CES-D	Concurrent autoimmune or inflammatory disease causing pain excluded (e.g., rheumatoid arthritis, systemic lupus erythematosus, inflammatory bowel disease)	Current use or history of opioid/narcotic analgesics excluded; willing to limit new medications or treatment modalities	0-10 verbal analog scale (spontaneous pain); HADS or CES-D	Age-matched healthy controls; white matter/ventricular and cardiac/respiratory regressors included	Female, right-handed participants; DMN, EAN, and MVN evaluated with dual regression ICA
Elkana et al. / 2024	41.73 (6.09) (HC: 41.52 (6.04))	Disease duration, years (SD) 4.31 (4.95)	Pain intensity during interview (range 0-100) 47.70 (20.04)	FIQ total score, (range 0-100) 33.45 (9.79)	HAM-D total score 15.58 (6.37); HAM-A total score 21.54 (6.32); general negative affect 25.27 (8.72); general positive affect 28.61 (8.00)	Whole-brain (VBM on GM and WM images, DBM, and cortical thickness)	Structural MRI; Resting-state fMRI	Depression symptoms and anxiety symptoms measured; MINI-Plus administered; diagnosis status NR	NR	Number of medications currently taken daily and during a crisis collected; medication effects not accounted for; antidepressants and anticonvulsants mentioned	WPI; SSS; Fibromyalgia General Questionnaire; FIQ; MINI-Plus; HAM-D; HAM-A; TAS; ERQ; PANAS; McGill Pain Questionnaire; pain intensity during interview; disease duration; symptom duration; time to diagnosis	33 matched healthy female controls; age and TIV used as covariates in VBM; age used as covariate/controlled in GLMs, FC t-tests, partial correlations, and ML feature regression	Mexican population; medRxiv preprint
Balducci et al. / 2022	41.7 (6.1) (HC: 41.5 (6.0))	NR	NR	NR	NR	NR	Structural MRI; Resting-state fMRI; Task-based fMRI	Major psychiatric disorder excluded (psychosis, bipolar disorder, obsessive compulsive disorder); depression and anxiety allowed; MINI-Plus, HAM-D, HAM-A collected	Cardiovascular disease and neurological illness excluded; migraine, tension-type headache, systemic lupus erythematosus, systemic hypertension excluded; neuropathic pain and irritable bowel syndrome allowed	Opioids excluded; analgesic or benzodiazepine rescue-doses stopped >=24 h before MRI; current medication received assessed	Tender points; WPI; SSS; MINI-Plus; HAM-D; HAM-A; Emotional Regulation Questionnaire; TAS; PANAS; Inventory of Personality Organization; FIQ; McGill Pain Questionnaire;	33 paired healthy controls; matched for age and years of education; all participants right-handed; exclusion criteria applied	Mexican population; unprocessed BIDS dataset; emotion processing and regulation task

Author / Year	Age (mean ± SD)	Pain duration	Pain intensity at baseline	FM severity / impact	Mood burden	Morphometry scope	Modality	Psychiatric comorbidities reported	Other comorbidities reported	Medication use reported / restricted	Clinical scales reported	Confounders controlled or matched?	Notes / NR
											Fibromyalgia general questionnaire		
Park et al. / 2022	Stanford: 48.11 (7.47); Duke: 34.86 (11.65)	9 months to 28 years (M=9.08 years, SD=7.35 years)	NR	NR	NR	NR	Resting-state fMRI	No uncontrolled anxiety or depression; BDI, STAI-State, STAI-Trait, POMS, PANAS measured; healthy controls did not have depression or anxiety	NR	Opioid-naive; no opioid medications within 90 days before study and lifetime use <1 month; other mood-altering/pain medications reported (NSAIDs, gabapentin/pregabalin, SNRIs, TCAs, SSRIs, LDN, anticonvulsants, muscle relaxants, anxiolytics, medical cannabis, triptans, trazodone, benzodiazepine, topical lidocaine patches)	WPI; SS; BDI; STAI-State; STAI-Trait; BIS/BAS; POMS; PANAS; BPI; PROMIS Fatigue	Pain duration, study site, and age included as covariates; healthy controls reported no chronic pain, no pain medications, and no depression/anxiety/major ongoing health conditions	All female; combined Stanford and Duke cohorts; pooled FM age not reported in main text; inclusion criterion average pain score >=2 (0-10 verbal scale) over the previous month
Kong et al. / 2021	51.6±11.6 (controls: 52.3±10.4)	NR	NR	FIQR 45.9±17.6	BDI-II 17.7±9.3	NR	Resting-state fMRI	NR (psychiatric comorbid diagnoses not reported)	NR (specific non-psychiatric comorbidities not reported)	Regular medications allowed; medication use during Tai Chi/end of study not tracked; 3/20 FM patients maintained regular pharmacological medications	FIQR (Function, Overall Impact, Symptom Severity); BDI-II; Mini-Mental State Examination; Physical Activity Readiness Questionnaire	Controls matched for age, gender, and BMI; exclusion criteria included medical conditions contributing to FM symptomatology, failure to pass the Physical Activity Readiness Questionnaire, MMSE <24, fMRI contraindications, prior Tai Chi training, and similar CAM in the past year; one HC with brain atrophy, four HCs with pain in the past week, and one FM patient with excessive head movement were excluded; age and gender were controlled for in BMA if needed	Pre/post 12-week Tai Chi design; final analysis: 20 FM and 19 pain-free controls
Larkin et al. / 2021	NR (groups age-matched within 2 years; exact mean ± SD not reported in uploaded article)	≥6 months of self-reported chronic widespread pain (discovery); at least one year of disease duration (replication)	VAS (0–100) prior to scan; overall FM baseline mean NR; combined FM tertiles: low 31.04 ± 12.92, medium 58.48 ± 5.99, high 76.87 ± 7.32	NR	NR (baseline numeric mood values not reported; HADS/CES-D/STAI used)	NR	Resting-state fMRI	Severe psychiatric illness excluded; depression and anxiety assessed (HADS in discovery; CES-D and STAI in subset replication); cutoffs used to classify possibly depressed or anxious vs not	Concurrent autoimmune or inflammatory disease causing pain excluded; systemic malignancy or infection such as HIV or hepatitis excluded; pregnant or nursing mothers excluded; BMI >36 excluded	Replication dataset required willingness to forgo new treatments and medication use for FM during the study; current or past opioid or narcotic analgesics excluded	VAS (0–100); HADS; CES-D; STAI	Age matched within 2 years; all participants female and right-handed; age and motion (meanFD and maxFD) included as nuisance covariates; multiple exclusion criteria including substance abuse and severe psychiatric illness	Two independent datasets; final analysis: discovery 38 FM/17 HC, replication 32 FM/20 HC; 1 FM patient missing clinical variables for low/medium/high pain analysis
Kong et al. / 2019	53.10 ± 11.58 (HC: 52.90 ± 11.12)	NR	NR	FIQR: 45.1 ± 18.6	BDI-II: 19.71 ± 11.12	NR	Resting-state fMRI	NR	Excluded medical conditions known to contribute to FM symptomatology (thyroid disease, inflammatory arthritis, systemic lupus erythematosus, rheumatoid arthritis, myositis, vasculitis, Sjogren's syndrome); serious medical conditions limiting participation excluded (dementia, neurological disease, cancer, cardiovascular disease, pulmonary disease, metabolic disease, renal disease, liver disease).	Allowed regular medications; participants could maintain routine physician visits.	FIQR (Function, Overall Impact, Symptom Severity); BDI-II	Matched for age, gender, and BMI; age, gender, and BDI scores included as covariates; exclusion criteria applied.	21 FM and 20 healthy controls completed the study; 12-week Tai Chi intervention.
Harper et al. / 2018	40.7 (10.2) (HC: 40.7 (11.5))	At least 6 months of chronic widespread pain	VAS: 68.3 (13.4)	NR	HADS-Dep: 4.9 (3.3); HADS-Anx: 6.5 (3.5)	Whole-brain (small volume correction for PAG)	Structural MRI; Resting-state fMRI	Depression and anxiety assessed with HADS; significant risk of suicide and severe acute/chronic psychiatric conditions excluded; healthy controls excluded if psychiatric disorder.	Excluded: cardiac diseases, glaucoma, autoimmune disease, systemic infections (eg, HIV, hepatitis), active cancer, pulmonary disease/dysfunction, unstable endocrine disease, unstable diabetes, unstable thyroid disease; healthy controls excluded for chronic medical illness.	Required withdrawal from CNS-active therapies marketed as antidepressants, stimulants, anorectic agents, or anticonvulsants; subjects abstained from over-the-counter analgesics for at least 8 hours before a study visit.	VAS, Brief Pain Inventory (severity, interference), HADS, NRS during CPM	Age- and sex-matched; VBM used age and total intracranial volume as regressors of no interest; rsFC entered white matter, CSF, and realignment parameters as covariates of no interest.	Cross-sectional baseline analysis from a larger milnacipran study; VBM n=15 FM/14 HC, resting-state analyses n=15 FM/13 HC, psychophysical CPM data n=13 FM/12 HC.

Author / Year	Age (mean ± SD)	Pain duration	Pain intensity at baseline	FM severity / impact	Mood burden	Morphometry scope	Modality	Psychiatric comorbidities reported	Other comorbidities reported	Medication use reported / restricted	Clinical scales reported	Confounders controlled or matched?	Notes / NR
Kutch et al. / 2017	38.4 ± 14.5 (HC: 33.6 ± 9.2)	NR	BPI Severity 4.5 ± 2.0	NR	HADS-A 7.1 ± 4.9; HADS-D 6.7 ± 3.7	Both (whole-brain gray matter tissue + right supplementary motor area/mid cingulate cortex)	Structural MRI; Resting-state fMRI	NR	NR	NR	HADS (Anxiety, Depression); BPI (Severity, Interference, body map); SF-12 (Physical Function, Mental Function)	Sex-matched female validation cohort; controlled for study site, total intracranial volume (structural only), and age	Fibromyalgia validation cohort only (n=23); pain reported at many body locations except the pelvis
Fallon et al. / 2016	38.5 ± 8.45 (HC: 39.40 ± 8.65)	9.13 ± 6.80 years	NR	FIQ 62.37 ± 15.84	BDI 19.5 ± 11.19	NR	Resting-state fMRI	NR	NR	Five patients used no medications; 11 used permissible doses with minimal central nervous efficacy or withdrew from non-permitted medications for at least 3 days	FIQ; MTPS; BDI; years of symptoms	Age-matched female controls; motion artefact threshold for exclusion (>3 mm translation, >1 degree rotation)	Mean time since diagnosis: 2.88 ± 1.34 years; no participants exceeded motion threshold
Ceko et al. / 2013	48.7 ± 7.8 years (HC: 48.8 ± 7.7 years)	11.5 (8.7) years	2.6 (2.7), range 0–9 (Clinical pain Intensity [VAS] / 11-point numerical pain intensity rating scale)	NR	HADS Anxiety: 10.3 (4.5); HADS Depression: 5.7 (4.3)	Whole-brain	Structural MRI; DTI; Resting-state fMRI	Current psychiatric conditions excluded, including severe depression and generalized anxiety disorder; depressive symptoms and anxiety measured with HADS	NR	Stable medication; opioid medications excluded. Reported medication classes: NSAIDs, antidepressants, muscle relaxants, anticonvulsants, cannabinoids, triptans	Clinical pain intensity rating scale; HADS; MFI-20; PCS; Edinburgh Handedness Inventory; IPAQ; VAS ratings for pressure stimuli	Individually age-matched (±3 years); matched for handedness, education, income, physical activity, menstrual phase, menopausal status; analyses controlled for age, pain duration, and/or menopausal status	Split into younger (<50) and older (>50) groups based on interaction with age
Jensen et al. / 2013	38 (7) years (HC: 34 [9] years)	11 (6) years	72 (14) VAS mm (weekly pain intensity)	NR	BDI score: 21 (11)	Both (whole-brain cortical thickness + region-specific volumes: rACC, amygdala, lateral orbitofrontal cortex)	Structural MRI; Task-based fMRI	Severe psychiatric disorder, suicide risk, and history of substance, drug, or alcohol abuse excluded; comorbid depressive symptoms assessed with BDI	NR	Treatments strictly limited; discontinuation required for antidepressants, mood stabilisers, tramadol, codeine, dextropropoxyphene, strong opioids/patches, anesthetic patches, anticonvulsants, centrally acting relaxants, joint injections, trigger/tender point injections, biofeedback, and TENS	VAS; BDI; pressure at VAS 50 (P50)	Age- and gender-matched healthy controls; site-matched across three sites; analyses controlled for age and, for some correlations, depression or duration	Present study included 26 FM patients and 13 matched healthy controls from a larger screened sample
Kaplan et al. / 2019	39.03 ± 11.04 (HC: 38.83 ± 12.18)	At least 6 months of self-reported chronic widespread pain	VAS: 4.88 ± 2.24 (0–10, immediately before fMRI)	NR	HADS-D: 5.1 ± 3.5; HADS-A: 7.3 ± 4.3 (subset: FM n=28; HC n=32)	NR	Resting-state fMRI	Severe psychiatric illness excluded; depression and anxiety assessed with HADS	Concurrent autoimmune or inflammatory disease causing pain; systemic malignancy or infection such as HIV or hepatitis excluded	Current medications collected; list in Supplementary Table 4. Validation cohort required willingness to forgo new medications/treatments; opioid or narcotic analgesics excluded in validation cohort	VAS; HADS	Age- and sex-matched HCs; analyses controlled for age; subset analyses also controlled for depression and anxiety	40 female FM patients and 46 HCs in discovery cohort; separate validation cohort of 11 female FM patients; right posterior insula 1H-MRS acquired in 40 FM and 27 HC

Diffusion MRI and Structural Connectivity

Year	Modality	Psychiatric comorbidities reported	Other comorbidities reported	Medication use reported / restricted	Clinical scales reported	Confounders controlled or matched?	Age (mean ± SD)	Pain duration	Pain intensity at baseline	FM severity / impact	Mood burden	Brain morphometry scope	[illegible]
22	DTI	Depression and anxiety symptoms measured (CES-D, STAI); questionnaires assessed symptoms, not diagnosis	NR	Pain medication not taken on day of scan	VAS; Pain Self-Perception Scale; Pain Catastrophizing Scale; CES-D; STAI; disease duration	Age entered as covariate; anxiety, depression, and age included as confounding factors; HC screened to ensure no pain conditions	46.4 ± 12.4 (HC 42.1 ± 12.5)	5.2 ± 5.1 years	VAS 7.2 ± 1.6	NR	STAI 52.8 ± 20.1; CES-D 31.0 ± 13.7	NR	[illegible]
014	DTI; Structural MRI	Depression and anxiety measured (BDI, BAI); HC excluded axis I psychiatric illness ('major depressive disorder, schizophrenia') and substance abuse	Secondary FM associated with inflammatory arthritis excluded; peripheral neuropathy excluded; concomitant acute pain in the upper extremities excluded	Analgesics, antidepressants, and anticonvulsants stopped 3 days before experiment	VAS; BDI; BAI; PSQI; FIQ; SF-MPQ; pain duration	HC age-, sex-, and education-matched; matched on handedness; ancillary analyses considered age and mean FA of the whole FA skeleton	44.9 ± 8.3 (HC 44.7 ± 8.8)	35.6 ± 31.1 months	Past week pain, VAS 51.8 ± 19.3 mm	FIQ 62.5 ± 13.2	BDI 19.0 ± 6.8; BAI 23.3 ± 10.8	NR	[illegible]
013	Structural MRI; DTI; Resting-state fMRI	Current psychiatric conditions excluded, including severe depression and generalized anxiety disorder; anxiety and depressive symptoms measured with HADS	Chronic pain conditions other than fibromyalgia excluded; major medical and neurological conditions excluded	Stable medication; NSAIDs, antidepressants, muscle relaxants, anticonvulsants, cannabinoids, triptans reported	Clinical pain intensity scale/VAS; pain duration; MFI-20; PCS; HADS	Individually age-matched (±3 years); groups further matched for handedness, education, income, physical activity, menstrual phase, menopausal status; analyses controlled for age, pain duration, and menopausal status as specified	48.7 ± 7.8 overall (younger 42.4 ± 5.9; older 55.0 ± 2.9)	11.5 (8.7) years overall (younger 8.8 (7.1); older 12.1 (9.0))	2.6 (2.7) overall (younger 2.8 (3.0); older 2.41 (2.3))	NR	HADS-A 10.3 (4.5); HADS-D 5.7 (4.3)	Whole-brain (voxel-based morphometry; cortical thickness analysis)	[illegible]

Morphometry

Author / Year	Age (mean ± SD)	Pain duration	Pain intensity at baseline	FM severity / impact	Mood burden	Morphometry scope	Modality	Psychiatric comorbidities reported	Other comorbidities reported	Medication use reported / restricted	Clinical scales reported	Confounders controlled or matched?	Notes / NR
Agoalikum et al., 2025	46.4±12.5 (HC: 42.1±12.5)	5.2±5.0 years	7.2±1.6 (PI)	NR	STAI: 52.8±20.0; CESD: 30.6±13.7	Whole brain	Structural MRI	Anxiety and depressive symptoms measured (STAI, CESD); no psychiatric diagnosis/comorbidity prevalence explicitly reported	Uncontrolled endocrine problems and neurological diseases excluded in FM; HC excluded for acute or chronic pain	FM patients were asked not to use painkillers on the day of testing	VRS pain intensity, PCS, STAI, CESD, helplessness, pain magnification, rumination, pain self-perception, FM years	Age- and sex-matched; age and TIV regressed out as covariates; partial correlations used age as covariate	All FM participants were female and right-handed; public dataset; HC screened to ensure no pain on testing day
Wu et al., 2025	Subtype 1: 43.3 (10.5); Subtype 2: 48.4 (10.1)	Subtype 1: 79.7 (69.5) months; Subtype 2: 51.9 (48.9) months	Subtype 1: 6.1 (1.3) (Pain VAS); Subtype 2: 6.2 (1.8) (Pain VAS)	Subtype 1: 43.4 (17.2) (FIQR); Subtype 2: 35.2 (20.1) (FIQR)	Subtype 1: BDI-II 9.6 (6.8), PSS 30.7 (9.0); Subtype 2: BDI-II 7.9 (7.1), PSS 25.0 (9.3)	Whole brain and Regional analysis	Structural MRI	Severe depression or anxiety excluded using BDI-II/HAMA cutoffs; depression also measured with BDI-II	Healthy controls required to have no chronic pain; history of brain tumor, head injury, or cerebral vascular accidents excluded	Patients were required to not receive any treatments for fibromyalgia for at least 4 weeks before MRI; later randomized to Ba-Duan-Jin or pregabalin intervention	Pain VAS, WPI, MFI-20, PSQI, BDI-II, PSS, FIQR	HC matched by age and educational level; HYDRA used age and total intracranial volume as covariates; partial correlations adjusted for age, symptom duration, baseline pain VAS and PSS	Female-only sample; single-center cohort; only 24 completed 12-week intervention
Liu et al., 2024	38.97±11.68	1.75 [0.5-5] years	4.5 [3–6] (Current VAS, 0–10)	WPI: 9.17±2.12; SSS: 7.31±1.69	HADS-anxiety: 9.14±3.56; HADS-depression: 8.26±3.12	Whole-brain (voxel-based morphometric analysis/FSL-VBM)	Resting-state fMRI; Structural MRI	Anxiety and depression evaluated with HADS; no psychiatric diagnosis/comorbidity prevalence explicitly reported	HC excluded for pain-associated conditions (migraine, dysmenorrhea); no FM non-psychiatric comorbidity prevalence explicitly reported	FM/AS patients were allowed to remain on stable preventative or as-needed medication (NSAIDs); previous medication including NSAIDs, bDMARDs, corticosteroids, duloxetine, pregabalin and sedatives was recorded	VAS (current pain), global pain, WPI, SSS, PSQI, HADS-Anxiety, HADS-Depression	Age and sex included as regressors of no interest; age, sex and TIV used as covariates in VBM; head-motion exclusions applied	Multigroup design (FM vs AS vs HC); structural and functional MRI acquired in one session
Oliveria Neto et al., 2024	41.73 (6.09) years	5 years (1° quartile = 2.0; 3° quartile = 8.0; range = 0.4-48.0)	NR	NR	Hamilton depression: 16.0 (1° = 11.0; 3° = 21.0); Hamilton anxiety: 23.0 (1° = 16.0; 3° = 27.5)	Whole-brain (cortical thickness across the entire cortical surface)	Structural MRI	History of psychiatric disorders excluded; anxiety and depression symptoms measured with Hamilton scales	Neurological diseases and cardiovascular conditions excluded; FM excluded for other pain conditions of intensity higher than FM pain; controls excluded for any pain and inflammatory/autoimmune/rheumatologic illness	Use of opioids excluded in the FM group	Toronto Alexithymia Scale (TAS), Positive and Negative Affect Scale, ERQ, Hamilton Depression Rating Scale, Hamilton Anxiety Rating Scale	HC paired by age and school degree; groups did not differ in age, sex, school degree, or socioeconomic status; age treated as a nuisance factor in group and correlation analyses	Female-only Mexican OpenNeuro dataset; 33 FM and 33 HC participants
Mosch et al., 2023	50.48±9.89 years (HC: 46.62±13.08 years)	14.9±11.8 years (range 2–44 years)	Pain severity 4.0 (MPI)	FIQ total: 60.2±17.6	CES-D: 22.1±6.5	Both (whole-brain + thalamus, amygdala, putamen, pallidum, caudate nucleus, supplementary motor area, middle temporal gyrus, cerebellum, insular, parahippocampal, prefrontal, orbitofrontal, cingulate, and primary and secondary somatosensory cortices)	Structural MRI; DTI	Psychotropic medication users excluded; psychotic patients, acute major depression, and bipolar disorder excluded; 16 FM patients reported previous major depressive episodes, 4 with generalized anxiety disorder and/or PTSD	NR	No pain medication on the examination day; opioid use suspended no later than 3 days prior to MRI (2 cases: fentanyl patches, tramadol)	MPI, FIQ-G, FSQ, CES-D, EHI, heat pain threshold, pain duration	Female-only sample; left-handed persons excluded; age included as covariate; TIV used as covariate in VBM; age used as covariate in DTI connectometry	One FM patient was excluded from DTI due to insufficient data quality
Izuno et al., 2023	41.6±7.4	NR	NR	NR	STAI state: 55.7±10.8; STAI trait: 59.6±12.8; HADS depression: 11.0±4.4; HADS anxiety: 10.5±4.2	Both (whole-brain + cingulate cortex, cerebellum, amygdala, temporal pole, insula, postcentral gyrus, and brainstem)	Structural MRI	HC had no history of psychiatric disorder; depression, anxiety, and psychological distress were assessed with CES-D, STAI, and HADS; no psychiatric diagnosis prevalence in FM explicitly reported	NR	NR	CES-D, STAI, HADS, PCS	Female-only and right-handed sample; age and total brain volume included as covariates; age-matched FM subgroup analysis (25 FM vs 25 HC)	Five FM patients were excluded due to white matter lesions
Aster et al., 2022	Patients all: 53.5±6.5; PNS: 53.5±6.7;	PNS: 16.8±10.8 years; noPNS:	PNS: 73.6±10.8 (GCPS pain intensity); noPNS:	PNS: 51.9±12 (FIQ); noPNS:	PNS: ADS 27.8±11.8, STAI 47.1±11.6; noPNS: ADS	Both (whole-brain + left medial frontal cortex, right posterior cingulate cortex)	Structural MRI; DTI; Resting-state fMRI	Psychiatric diseases such as major depression excluded (past and present); depressiveness assessed	Other current autoimmune or inflammatory diseases that can cause pain excluded (e.g., rheumatoid arthritis, systemic lupus erythematosus, chronic inflammatory bowel	All patients were off pain medication for 3 days before examination; no anticonvulsants, antihistamines, muscle	GCPS, NPSI, ADS, PCS, STAI, FIQ, SSS, O'Leary-Sant Symptom and Problem Index, WPI, tender points, time since diagnosis, duration of pain	Healthy controls were age- and sex-matched; age and GCPS pain intensity included as covariates in structural	Female-only sample; FM subgrouping based on intraepidermal nerve

Author / Year	Age (mean ± SD)	Pain duration	Pain intensity at baseline	FM severity / impact	Mood burden	Morphometry scope	Modality	Psychiatric comorbidities reported	Other comorbidities reported	Medication use reported / restricted	Clinical scales reported	Confounders controlled or matched?	Notes / NR
	noPNS: 53.4±6.5	18.8±12.7 years	64±15.1 (GCPS pain intensity)	42.3±13.2 (FIQ)	21.2±11.4, STAI 44.3±13.3			with ADS; anxiety assessed with STAI	disease); neurological and cardiovascular diseases excluded	relaxants, or benzodiazepines within 4 weeks before examination		analyses; participant motion parameters included as first-level covariates; pain intensity (GCPS) and ADS included as second-level covariates in resting-state fMRI; motion thresholds used for exclusion	fiber density (PNS vs noPNS)
Tu et al., 2022	46.4±12.4	5.2±5.1 years	7.2±1.6 (VAS)	NR	STAI 52.8±20.1; CES-D 31.0±13.7	Whole-brain	Structural MRI	Anxious and depressive symptoms assessed with STAI and CES-D; scales were used for symptom assessment, not for diagnosis	Uncontrolled endocrine problems and neurological diseases excluded; HC excluded for acute or chronic pain (e.g., osteoarthritis)	FM patients were asked not to use painkiller on the day of testing	VRS, PCS, pain self-perception scale, STAI, CES-D, disease duration	HC subjects were age-matched; between-group analyses used age as covariate; analyses were additionally controlled for comorbid affective measures (depression and anxiety)	Female-only, right-handed sample; public OpenNeuro dataset (ds001928)
Leon-Llamas et al., 2021	54.18 (10.12) (HC: 53.37 (4.47))	Years with FM Symptoms: 19.38 (12.70)	NR	FIQ-100: 58.05 (17.85)	GDS-15: 6.88 (4.07)	Region-specific (hippocampal tail, parasubiculum, presubiculum, subiculum, CA1, CA2/3, CA4, HATA, GC-DG, molecular layer, fimbria, hippocampal fissure; whole hippocampus)	Structural MRI	Depressive symptoms measured with GDS-15; HC inclusion required GDS score 0 to 5; no psychiatric diagnosis/comorbidity prevalence explicitly reported	Cerebral injury/pathology excluded in FM (traumatic brain disease, cerebral stroke, brain tumor, other diagnosed pathology)	NR	GDS-15, MMSE, FIQ	HC sample filtered to obtain healthy subjects of similar age and gender; regression models included age, eTIV, group, GDS-15 score, and MMSE score as independent variables	Female-only sample; HC data from OASIS-3; one FM subject removed because an illegible MRI sequence was obtained
Kim et al., 2021	44.9 (8.3) (HC: 45.0 (8.4))	35.6 (31.1) months	Pain intensity VAS—last week: 52.8 (20.3)	NR	Beck Depression Inventory Score: 19.0 (6.8)	Both (whole-cortex cortical thickness + region-specific thalamic volume/vertex-wise shape [left posterior thalamus encompassing the ventral posterior lateral and pulvinar nuclei])	Structural MRI; Resting-state fMRI	Psychiatric disorders excluded (major depressive disorder, schizophrenia, substance abuse); depressive symptoms measured with BDI	Secondary FM associated with inflammatory arthritis excluded; CNS disorders, peripheral neuropathy, acute upper-extremity pain, and pregnancy excluded	Stopped medications influencing the somatosensory system at least 3 days before assessment (e.g., analgesics, antidepressants, anticonvulsants); medication classes recorded in Table 1 (analgesics/muscle relaxants/NSAIDs, antidepressants, anticonvulsant)	BDI, VAS, SF-MPQ, PPT, EPT, tender point count, pain duration	Age-, sex-, and education level-matched HC; age and intracranial volume used as covariates in thalamic volume analysis; age and average thickness as covariates in cortical thickness/SC; age as covariate of no interest in functional connectivity	Right-handed female primary FM sample; 1 HC excluded from structural MRI due to claustrophobia; only 12 FM had resting-state fMRI
Feraco et al., 2020	mean age 43.2 years (HC: mean age 41.3 years)	NR	NR	NR	NR	Region-specific (ventrolateral prefrontal cortices (VLPFC), thalami, cingulate cortex subparts, insular cortex, temporal cortex, occipital cortex, bilateral caudate, putamen, thalamus, brainstem)	12 FM and 12 matched healthy controls; combined structural MRI and single-voxel MR spectroscopy	Depression and anxiety symptoms were not collected; no psychiatric comorbidities reported	No comorbidity (other rheumatologic diseases and/or central nervous system pathologies)	All patients suspended pharmacologic therapies known to affect brain function for 1 week before the study; acetaminophen was permitted to control pain; controls took no drugs or illicit substances affecting cognitive functions or brain metabolites	Tender point count, VAS, FIQ, onset, duration	HC matched for sex and age; analyses were controlled for age and intracranial volume	Retrospective study; combined structural MRI and single-voxel MR spectroscopy; FM sample listed as 11 F and 1 M in Methods
Sundermann et al., 2019	52.60 ± 11.10 (HC: 52.24 ± 9.79)	15.76 ± 10.58 years	3.80 ± 2.40 (clinical pain, 0–10)	FS: 16.68 ± 4.35 (WPI: 9.96 ± 3.84; SSS: 8.72 ± 1.57)	PHQ-9: 11.60 ± 4.26	Both (whole-brain VBM + ACC, frontal gyrus (BA 10), insula, parahippocampal gyrus, amygdala)	25 female FMS; compared with healthy controls and osteoarthritis patients	Major psychiatric disorders excluded (major depression, anxiety disorder, schizophrenia, or addiction); depressive symptoms measured with PHQ-9	Major medical disorders excluded (e.g., diabetes, cancer) and neurological disorders excluded (e.g., epilepsy, dementia)	Not taking any pain or central-acting medication on a regular basis, or able to discontinue such medication 72 hr prior to the study	Numeric rating scale pain intensity, pain duration, SF-36 (PCS/MCS), PHQ-9, CTQ, WPI, SSS, FS	Female healthy controls were age-matched; age and TIV were entered as covariates in the VBM analysis	Comparison included OA and HC groups; one HC was excluded due to substantial movement artefacts
Fayed et al., 2017	41.7; SD=7.3	NR	NR	NR	HADS-Anx 6.20; SD=2.8; HADS-Dep 5.90; SD=3.8	Whole-brain (VBM8 whole-brain gray matter maps)	Structural MRI	Other Axis I psychiatric disorders excluded; anxiety and depression assessed with HADS	NR	No pharmacological treatment 1 week before the study began	HADS-Anx, HADS-Dep, MMSE	Controls adjusted for gender, age, years of education, and ethnic group; statistical model included age, sex, and total intracranial volume as nuisance variables	Three-group design (FM, long-term meditators, healthy controls); no images were discarded after quality check
Pomares et al., 2017	61 ± 5.4	16 ± 9 yr (symptom duration)	4.8 ± 2.2 (current	FIQ 51 ± 18	BDI 16 ± 10; HADS 16 ± 6	Both (whole-brain VBM + ACC MRS voxel; region-focused analyses of	Structural MRI	Psychiatric disorders excluded; depression and anxiety measured with Beck	Pain conditions other than fibromyalgia excluded; uncontrolled medical conditions and	Participants using benzodiazepine medication more than once a week were	Current pain level (11-point numerical rating scale), Beck Depression Inventory, Hospital	Healthy controls matched for age, body mass index, education	Postmenopausal women only; multimodal study

Author / Year	Age (mean ± SD)	Pain duration	Pain intensity at baseline	FM severity / impact	Mood burden	Morphometry scope	Modality	Psychiatric comorbidities reported	Other comorbidities reported	Medication use reported / restricted	Clinical scales reported	Confounders controlled or matched?	Notes / NR
			pain level, 1-10)			VLPFC/inferior frontal gyrus, thalami, cingulate cortex subparts, insular, temporal and occipital cortices, bilateral caudate, putamen, thalamus, brainstem)		Depression Inventory and Hospital Anxiety and Depression Scale; none had clinically significant levels by questionnaire cutoffs	neurological disorders excluded; body mass index ≥30 kg/m2 excluded	excluded; occasional benzodiazepine users were off medication for at least 48 h before the PET scan	Anxiety and Depression Scale, Pain Catastrophizing Scale, Fibromyalgia Impact Questionnaire, time since diagnosis, symptom duration	level, income, and physical activity level; age used as a covariate of no interest in VBM analysis and in spectroscopy analyses	including [18F]flumazenil PET, proton MR spectroscopy, and T1 relaxometry; final sample 26 FM and 25 controls
Robinson et al., 2015	44.1 years (HC: 42.2 years)	NR	NR	NR	NR	Region-specific (55 neuroanatomical region volumes from FreeSurfer)	Structural MRI	Mood variables including depression and anxiety were assessed by VAS; no psychiatric diagnoses/comorbidity prevalence explicitly reported	Healthy controls were pain-free; no other comorbidities explicitly reported	NR	VAS pain intensity; VAS mood ratings for depression, anxiety, frustration, anger, and fear	Age- and sex-matched healthy pain-free controls	Female-only sample; machine-learning classification study; structural MRI volumes from 55 neuroanatomical regions processed in FreeSurfer
Agoalikum et al., 2025	46.4±12.5 (HC: 42.1±12.5)	5.2±5.0 years	7.2±1.6 (PI)	NR	STAI 52.8±20.0; CESD 30.6±13.7	Whole-brain	Structural MRI; Resting-state fMRI	Anxiety and depressive symptoms assessed with STAI and CESD; no psychiatric diagnoses/comorbidity prevalence explicitly reported	Uncontrolled endocrine problems and neurological diseases excluded in FM; HC excluded for acute or chronic pain	FM patients were asked not to use painkillers on the day of testing	VRS pain intensity, PCS, STAI, CESD, helplessness, pain magnification, rumination, pain self-perception, FM years	Age-matched female groups; age and TIV used as covariates in VBM; age used as covariate in partial correlations; age and mean framewise displacement regressed out in GCA	Public OpenNeuro dataset; all participants were female and right-handed; study combined VBM and Granger causality analysis
McCrae et al., 2015	51.0 (13.5) (HC: 43.4 (11.3))	11.9 (9.9) years	NR	PDI 30.8 (15.7)	BDI 14.5 (10.2)	Region-specific (hippocampus)	Structural MRI	Bipolar disorder and severe untreated psychopathology (e.g., schizophrenia, substance abuse) excluded; depressive symptoms measured with BDI-II	Sleep disorder other than insomnia excluded; seizure disorder, significant medical (e.g., cancer) or neurological (e.g., dementia) disorder excluded; HC excluded for painful neuropathies, diabetes, or cancer	HC regularly taking NSAIDs, antidepressants, antihistamines, analgesics, anticonvulsants, muscle relaxants, or benzodiazepines were excluded; FM required no prescribed or OTC sleep medication for ≥1 month or stable for ≥6 months	BDI-II, MMSE, sleep efficiency, Pain Disability Index, symptom duration	ANCOVA controlled for age, whole brain gray matter volume, anatomic SNR, and BDI; semipartial correlations controlled for age, total gray matter volume, SNR, and BDI	Female-only sample; FM and HC were scanned on two different 3T scanners
Robinson et al., 2011	43.1 (6.9) (HC: 42.4 (9.8))	NR	2.9 (1.2) VAS units	NR	BDI 13.2 (9.5); STAI-State 33.9 (3.4); STAI-Trait 43.1 (3.4)	Region-specific (left and right THAL, THAL Medial Dorsal Nucleus, S1, S2, inferior parietal lobule, post INS, mid INS, dorsal ACC, rostral ACC, mid ACC, precentral gyrus, inferior frontal gyrus, medial frontal gyrus, superior temporal gyrus, cerebellum)	Structural MRI	Depression and anxiety measured with BDI and STAI; frustration, anger, and fear also assessed; BDI scores in FM were below the cut-off for major depressive episodes	Healthy controls were pain-free; no other non-psychiatric comorbidities explicitly reported	NR	Medical College of Virginia Pain Questionnaire (pain, negative emotions, impact on life), BDI, STAI, baseline somatic pain VAS	Total cortical gray matter volume was calculated and used as a covariate during subsequent analyses	Female-only sample; VBM focused on 19 pain-related VOIs defined from a prior fMRI study
Baker et al., 2022	48.12 ± 9.6 (HC: 48.42 ± 10.3)	NR	BPI Severity 5.70 ± 2.1	BPI Interference 5.32 ± 2.78	BDI 15.76 ± 8.88	Whole-brain	Structural MRI	Depression and anxiety measured with BDI and STAI; HC participants were required to have no depression or anxiety	FM participants required no other disorder explaining symptoms/pain; HC participants required no chronic pain; overall exclusions included MRI contraindications, pregnancy or nursing, and claustrophobia	FM participants were not taking any opioid medications, had not taken opioids within 90 days prior to study visits, and had not taken opioids for longer than 30 days in their lifetime	BDI, STAI State, STAI Trait, BIS/BAS Scales, POMS, PANAS, Fibromyalgia Assessment Form, BPI, PROMIS Fatigue	Sex-matched healthy controls; two-sample t-tests controlled for total intracranial volume as a covariate of no interest	All participants were female; one participant was excluded because of scan artifacts; medRxiv preprint
Harper et al., 2018	40.7 (10.2) (HC: 40.7 (11.5))	NR	VAS 68.3 (13.4)	BPI Int 5.0 (2.5)	HADS Dep 4.9 (3.3); HADS Anx 6.5 (3.5)	Both (whole-brain + PAG)	Structural MRI; Resting-state fMRI	Hospital Anxiety and Depression Scale (HADS) administered; FM excluded severe acute or chronic psychiatric conditions that could increase risk or interfere with results; HC excluded psychiatric disorder	FM exclusions included cardiac diseases, glaucoma, autoimmune disease, systemic infections, active cancer, pulmonary disease/dysfunction, unstable endocrine disease, unstable diabetes, unstable thyroid disease, BMI >36; HC excluded chronic medical illness and chronic pain disorder	FM participants had to be willing to withdraw from CNS-active therapies marketed as antidepressants, stimulants, anorectic agents, or anticonvulsants; subjects abstained from over-the-counter analgesics at least 8 hours before a study visit	Brief Pain Inventory (BPI severity/interference), 100-mm clinical pain VAS, HADS, CPM 0–100 NRS ratings	Age- and sex-matched healthy controls; VBM used age and total intracranial volume as regressors of no interest; resting-state analyses excluded excessive head motion and included white matter, cerebrospinal fluid, and realignment parameters as covariates of no interest	Right-handed female sample; baseline data from a larger milnacipran treatment study; one HC excluded from resting-state analyses for excessive head motion

Author / Year	Age (mean ± SD)	Pain duration	Pain intensity at baseline	FM severity / impact	Mood burden	Morphometry scope	Modality	Psychiatric comorbidities reported	Other comorbidities reported	Medication use reported / restricted	Clinical scales reported	Confounders controlled or matched?	Notes / NR
Kutch et al., 2017	38.4 ± 14.5	NR	4.5 ± 2.0 (BPI Severity)	NR	HADS Anxiety 7.1 ± 4.9; HADS Depression 6.7 ± 3.7	Whole-brain	Structural MRI; Resting-state fMRI	Anxiety and depression measured with HADS in the neuroimaging validation sample; FM-specific psychiatric diagnostic exclusions NR	NR for the fibromyalgia validation subgroup	NR for the fibromyalgia validation subgroup; medication comparison reported only for the UCPPS discovery cohort	HADS (anxiety, depression), BPI (body map, severity, interference), SF-12 (physical function, mental function)	Sex-matched female validation cohort; GLMs controlled for study site, total intracranial volume (structural analyses only), and age	Fibromyalgia participants were a validation cohort within a larger UCPPS study (N=23); women only; pelvic pain absent
Ceko et al., 2013	48.7 ± 7.8 (younger: 42.4 ± 5.9; older: 55.0 ± 2.9)	11.5 (8.7) years (younger: 8.8 (7.1); older: 12.1 (9.0))	2.6 (2.7) (clinical pain intensity, 0–10) (younger: 2.8 (3.0); older: 2.41 (2.3))	NR	HADS Anxiety 10.3 (4.5); HADS Depression 5.7 (4.3) (younger: 9.8 (5.4)/5.6 (4.1); older: 10.8 (3.3)/5.8 (4.7))	Whole-brain (VBM; cortical thickness analysis)	Structural MRI; DTI; Resting-state fMRI	Current psychiatric conditions excluded, including severe depression and generalized anxiety disorder; depressive symptoms and anxiety measured with HADS	Chronic pain conditions other than fibromyalgia excluded; major medical and neurological conditions excluded	Patients were on stable medication; opioid medications excluded; Table 1 reports NSAIDs, antidepressants, muscle relaxants, anticonvulsants, cannabinoids, and triptans	Clinical pain intensity NRS (0-10), pain duration, HADS, MFI-20, PCS, pressure-stimulus intensity/unpleasantness VAS	Healthy controls individually age-matched (±3 years); groups further matched for handedness, education, income, physical activity, menstrual phase, and menopausal status; imaging analyses controlled for age, with some models additionally controlling for pain duration and/or menopausal status	Female-only sample
Jensen et al., 2013	38 (7) (HC: 34 (9))	11 (6) years	72 (14) (Weekly pain intensity, VAS mm)	NR	BDI score 21 (11)	Both (whole-brain + rostral anterior cingulate cortex, amygdala, lateral orbitofrontal cortex)	Structural MRI; Task-based fMRI	Severe psychiatric disorder, suicide risk, and history of substance/drug/alcohol abuse excluded; depressive symptoms measured with BDI and analyzed as comorbid depressive symptoms	NR	Medications/treatments strictly limited; discontinuation required for antidepressants, mood stabilisers, selected analgesics, strong opioids, anesthetic patches, anticonvulsants, centrally acting relaxants, joint/trigger/tender point injections, biofeedback, and TENS	VAS (weekly pain intensity; experimental pain ratings), BDI, pressure at VAS 50, duration of FM symptoms	Age- and gender-matched controls; site-matched across 3 sites; cortical thickness analyses covaried for age; correlations controlled for age and depression or duration; site-factor tested statistically	Female-only, multi-site baseline sample from a pharmacological fMRI clinical trial; 1.5 T scanners
Fallon et al., 2013	38.5 ± 8.45 years (HC: 39.4 ± 8.7 years)	9.1 ± 6.8 years	NR	FIQ 63.37 ± 15.83	BDI 19.50 ± 11.19	Both (whole-brain + brainstem, bilateral thalami, hippocampi, amygdalae, putamen, caudate nucleus, accumbens nucleus, and pallidum)	Structural MRI	BDI measured; alcohol/drug abuse excluded; medications with central nervous system effects (e.g., tricyclic antidepressants) not suitable for withdrawal excluded	Additional disease/disorders not commonly comorbid with FMS (e.g., diabetes, hypertension) excluded	Analgesics withdrawn 3-5 days; paracetamol permitted; central nervous system medications generally excluded unless stable low-dose pregabalin, gabapentin, or amitriptyline was considered acceptable	MTPS, BDI, FIQ, duration of symptoms, time since diagnosis	Age- and sex-matched female controls; brainstem volume analyses adjusted for total intracranial volume and total grey matter volume; Chiari I measure entered as covariate	Female-only homogeneous sample; shape analysis of 15 subcortical regions plus VBM

Supplementary Table S3 (figure 3)

Paradigm	ROI family	Hyperactivation (n)	Hypoactivation (n)	Total reports (n)	Dominant direction	Normalized balance	Figure 3 score (0–1)
Cognitive / control	Amygdala	1	0	1	Activation	0,14	0,57
Cognitive / control	Basal ganglia / striatum	3	9	12	Deactivation	-0,86	0,07
Cognitive / control	Brainstem / midbrain	1	1	2	Tie/Mixed	0,00	0,50
Cognitive / control	Cerebellum	0	2	2	Deactivation	-0,29	0,36
Cognitive / control	Cingulate cortex (ACC/MCC/PCC)	2	6	8	Deactivation	-0,57	0,21
Cognitive / control	Insula	4	1	5	Activation	0,43	0,71
Cognitive / control	Lateral prefrontal cortex	1	7	8	Deactivation	-0,86	0,07
Cognitive / control	Lateral temporal / fusiform	3	4	7	Deactivation	-0,14	0,43
Cognitive / control	Medial prefrontal cortex / OFC	1	0	1	Activation	0,14	0,57
Cognitive / control	Medial temporal / limbic	1	3	4	Deactivation	-0,29	0,36
Cognitive / control	Parietal association cortex	2	4	6	Deactivation	-0,29	0,36
Cognitive / control	Sensorimotor / premotor cortex	4	6	10	Deactivation	-0,29	0,36
Cognitive / control	Thalamus	1	2	3	Deactivation	-0,14	0,43
Cognitive / control	Visual / occipital cortex	2	1	3	Activation	0,14	0,57
Pain anticipation	Basal ganglia / striatum	0	2	2	Deactivation	-0,29	0,36
Pain anticipation	Brainstem / midbrain	1	4	5	Deactivation	-0,43	0,29
Pain anticipation	Cingulate cortex (ACC/MCC/PCC)	0	2	2	Deactivation	-0,29	0,36
Pain anticipation	Insula	0	1	1	Deactivation	-0,14	0,43
Pain anticipation	Lateral prefrontal cortex	1	4	5	Deactivation	-0,43	0,29
Pain anticipation	Medial prefrontal cortex / OFC	0	1	1	Deactivation	-0,14	0,43
Pain anticipation	Parietal association cortex	1	1	2	Tie/Mixed	0,00	0,50
Pain anticipation	Sensorimotor / premotor cortex	0	4	4	Deactivation	-0,57	0,21
Pain anticipation	Visual / occipital cortex	0	3	3	Deactivation	-0,43	0,29
Pain empathy	Cingulate cortex (ACC/MCC/PCC)	1	1	2	Tie/Mixed	0,00	0,50
Pain empathy	Insula	1	0	1	Activation	0,14	0,57
Pain empathy	Lateral prefrontal cortex	0	1	1	Deactivation	-0,14	0,43
Pain empathy	Sensorimotor / premotor cortex	2	5	7	Deactivation	-0,43	0,29
Pain empathy	Thalamus	0	1	1	Deactivation	-0,14	0,43
Pain empathy	Visual / occipital cortex	0	1	1	Deactivation	-0,14	0,43

Pain-evoking	Amygdala	1	1	2	Tie/Mixed	0,00	0,50
Pain-evoking	Basal ganglia / striatum	1	0	1	Activation	0,14	0,57
Pain-evoking	Brainstem / midbrain	0	1	1	Deactivation	-0,14	0,43
Pain-evoking	Cerebellum	3	0	3	Activation	0,43	0,71
Pain-evoking	Cingulate cortex (ACC/MCC/PCC)	6	1	7	Activation	0,71	0,86
Pain-evoking	Insula	3	2	5	Activation	0,14	0,57
Pain-evoking	Lateral prefrontal cortex	8	3	11	Activation	0,71	0,86
Pain-evoking	Lateral temporal / fusiform	3	2	5	Activation	0,14	0,57
Pain-evoking	Medial prefrontal cortex / OFC	4	1	5	Activation	0,43	0,71
Pain-evoking	Medial temporal / limbic	3	3	6	Tie/Mixed	0,00	0,50
Pain-evoking	Parietal association cortex	2	0	2	Activation	0,29	0,64
Pain-evoking	Sensorimotor / premotor cortex	8	1	9	Activation	1,00	1,00
Pain-evoking	Thalamus	2	1	3	Activation	0,14	0,57
Pain-evoking	Visual / occipital cortex	1	0	1	Activation	0,14	0,57
Reward / motivational	Basal ganglia / striatum	0	1	1	Deactivation	-0,14	0,43
Reward / motivational	Insula	0	2	2	Deactivation	-0,29	0,36
Reward / motivational	Lateral prefrontal cortex	4	0	4	Activation	0,57	0,79
Reward / motivational	Medial prefrontal cortex / OFC	0	3	3	Deactivation	-0,43	0,29
Reward / motivational	Sensorimotor / premotor cortex	4	0	4	Activation	0,57	0,79
